# Interleukin-34 orchestrates bone formation through its binding to bone morphogenic proteins

**DOI:** 10.7150/thno.107340

**Published:** 2025-02-11

**Authors:** Javier Muñoz-Garcia, Jorge W. Vargas-Franco, Kristina Schiavone, Marcus T. Keatinge, Robin Young, Jérôme Amiaud, Laurie Fradet, Jean-François Jégou, Hideo Yagita, Claudine Blin-Wakkach, Abdelilah Wakkach, Denis Cochonneau, Emilie Ollivier, Martine Pugière, Corinne Henriquet, Marie Legendre, Irina Giurgea, Serge Amselem, Marie-Françoise Heymann, Stéphane Télétchéa, Frédéric Lézot, Dominique Heymann

**Affiliations:** 1 Nantes University, CNRS, US2B, UMR 6286, Nantes, France, 44300.; 2 Institut de Cancérologie de l'Ouest, Saint-Herblain, France, 44805.; 3 University of Antioquia, Department of Basic Studies, Faculty of Odontology, Medellin, Colombia, 1225.; 4 Université of Sheffield, School of Medicine and Population Health, Sheffield, UK, S10 2TN.; 5 University of Edinburgh, Centre for Discovery Brain Sciences, Edinburgh, UK, EH8 9XD.; 6 Sheffield Teaching Hospitals NHS Foundation Trust, Sheffield, UK, S10 2JF.; 7 Nantes University, Department of Histology and Embryology, Medical School, Nantes, France, 44000.; 8 University of Poitiers, LITEC, UR15560, Poitiers, France, 86073.; 9 Juntendo University, Department of Immunology, School of Medicine, Tokyo, Japan, 113-8421.; 10 University Côte d'Azur, CNRS, UMR7370, LP2M, Nice, France, 06107.; 11 University of Montpellier, INSERM, UMR1194, IRCM, Montpellier, France, 34298.; 12 Sorbonne University, INSERM, UMR933, Hospital Armand-Trousseau (AP-HP), Paris, France, 75012.

**Keywords:** development, bone homeostasis, osteoclastogenesis, osteoblastogenesis, protein docking

## Abstract

**Rationale:** During development, the contribution of IL34, a ligand of macrophage colony stimulating factor receptor (MCSFR), has not been fully defined. Together with its twin cytokine MCSF, they display an essential role in macrophage differentiation and activation, including tissue specialized macrophages. The mechanism of action of each molecule involves the phosphorylation of MCSFR in varying intensity and kinetics. Furthermore, IL34 can interact with other receptors and cofactors, opening a wide range of modulations during development. The aim of this work was to investigate these effects through the suppression of IL34 in different animal models and study molecular interactions, with a particular focus on osteoclast / osteoblast regulation.

**Methods:** Two different and unique models of *IL34^-/-^* were generated in zebrafish and mouse. The skeleton of both species was analyzed and compared by histological and morphometric (Micro-CT) approaches. The role of IL34 and new partners in osteoclast and osteoblast differentiation was analyzed by multiple techniques including mineralization assays, tartrate resistant acid phosphatase (TRAP) staining, receptor phosphorylation and activation assays, and gene expression (real-time quantitative PCR) studies. Furthermore, protein interactions were studied by surface plasmon resonance approach and protein-protein docking ClusPro analysis.

**Results:** Significant growth delay and hypo-mineralization of skeletal elements were observed in both *IL34^-/-^* models, as well as craniofacial dysmorphoses in mice. With regard to bone cells, an unexpected increase in the number of osteoclasts and an accumulation of pre-osteoblasts were observed in mice lacking IL34. For the first time, *in vitro* analyses complemented by protein binding and molecular docking studies established that IL34 interacts directly with certain Bone Morphogenetic Proteins (BMPs), modulating their various activities such as the stimulation of osteoblast differentiation.

**Conclusions:** A new mechanism of action for IL34 through BMPs has been characterized. IL34 interactions with MCSFR and BMPs appear crucial for both osteoclastogenesis and osteoblastogenesis, impacting bone tissue homeostasis and development. The potential interaction of IL34 with different members of the BMP family and their functional impact, including pathological situations such as cancer, should be further explored, opening new therapeutic perspectives.

## Introduction

Interleukin-34 (IL34) is a soluble cytokine discovered in 2008 by its ability to bind to macrophage colony-stimulating factor receptor (MCSFR), also known as c-FMS/CSF1R/CD115 1. This work has rekindled interest in the MCSFR signaling pathway and in the roles of the twin cytokines MCSF/IL34 in the differentiation and activation of myeloid cell lineage, such as macrophages, Langerhans cells, microglia cells and osteoclasts 2-5. IL34 binding to MCSFR can occur as a homodimer or heterodimer with MCSF/CSF1, depending on the relative amounts of the two cytokines 6. The twin cytokines induce similar patterns of phosphorylation of MCSFR but with variable intensity and kinetics, raising the question of their functional redundancy and specific functions. Their functional redundancy is confirmed by the greater severity of the bone phenotype associated with *MCSFR* versus *MCSF* invalidation in mice 7,8. As far as the implications of IL34 during bone development are concerned, the data currently available are scarce, and focus mainly on its pro-osteoclastic action via its binding to the M-CSFR receptor (the binding that led to its identification 1). Similarly to MCSF, IL34, by binding to MCSFR on the surface of osteoclastic precursors of myeloid origin, induces their engagement in the osteoclastic differentiation pathway which will then be completed by RANKL stimulation 2,9-14. Regarding the source of IL34, osteoblastic expression 15,16 and chondroblastic expression 17 have been reported, suggesting that IL34 may be involved in communications between bone forming cells and osteoclasts. In the absence of a detailed description of the skeletal phenotype associated with IL34 invalidation during growth, the roles of IL34 in this growth process are still unclear. Additional receptors of IL34 have been identified and include Protein-Tyrosine Phosphatase β/ζ receptor (PTPβ/ζ) 18*,* Triggering Receptor Expressed on Myeloid cells-2 (TREM2) 19 and syndecan-1 20. PTPβ/ζ is mainly expressed by neuronal progenitors and glial cells and known as pleiotrophin/heparin-binding growth-associated molecule receptor 21. TREM2 is a lipid-binding receptor 22, carried by myeloid lineage cells, whose differentiation and migratory capacities it modulates 23. Finally, IL34 binds to Syndecan-1 (CD138) and this binding modulates IL34-mediated activation of MCSFR 20. The diversity of IL34 receptors and co-ligands suggests that this cytokine plays an important role in the differentiation and activation of myeloid, neural and glial cells. In this context, the existence of other partners for IL34 must not be excluded. To analyze these functions, IL34 was suppressed in zebrafish and mouse and the phenotypes of these mutants was fully deciphered during development. New partners have been identified and their functional and biological implications have been analyzed.

## Results

### Zebrafish and mouse IL34 null models show significant alterations of the skeleton during development

*IL34* invalidation was genetically achieved in zebrafish and mouse using respectively CrispR/Cas9 technology on one-cell stage embryos and conventional homologous recombination in embryonic stem cells.

**Figure 1:**
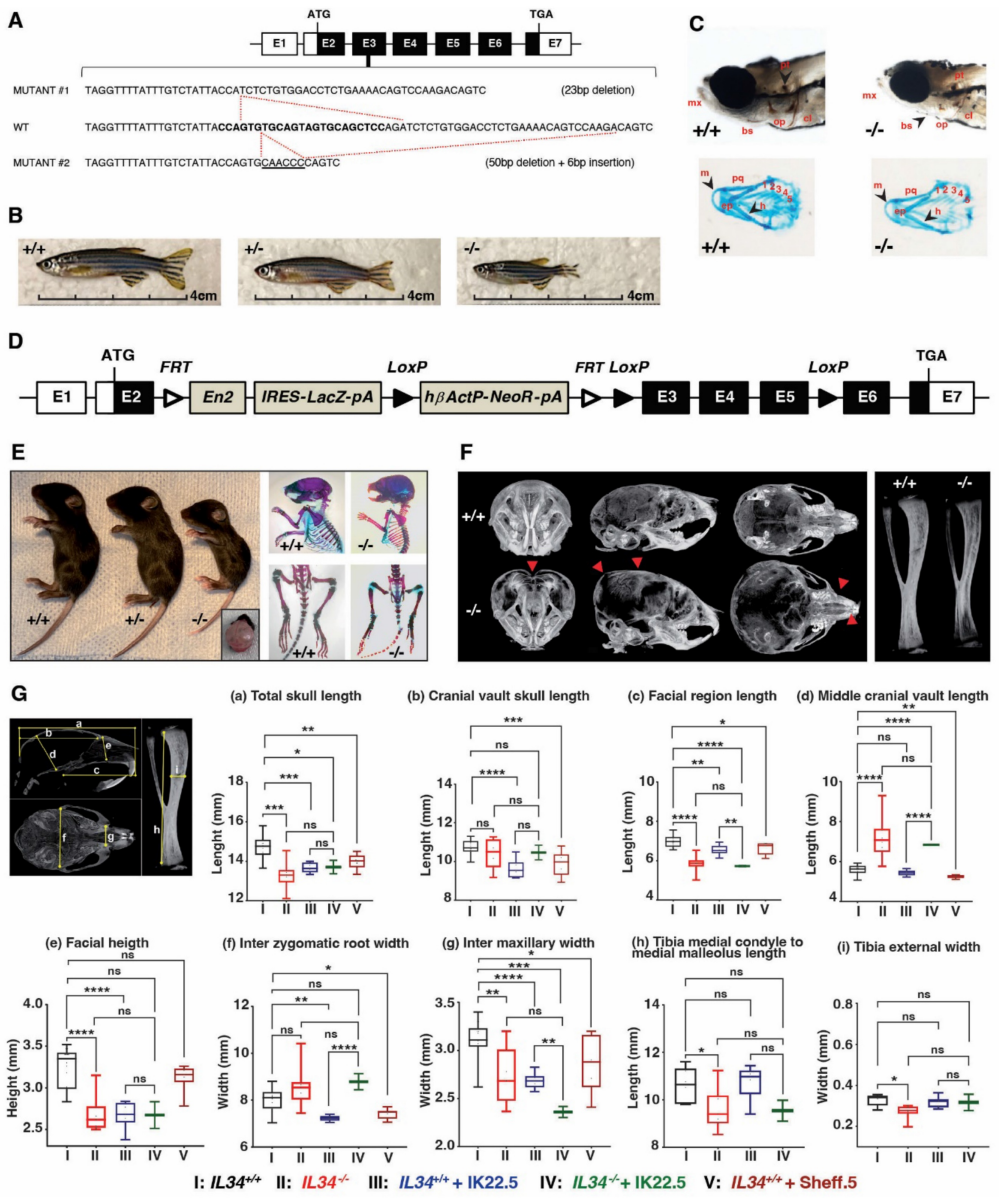
** Growth alterations associated with *IL34* genetic invalidations in zebrafish and mouse**. (**A**) Scheme of IL34 Exon3 genetic alterations induced by CrispR/Cas9 technology in zebrafish. (**B**) Images of zebrafish mutants compared to the control at age of 3 months. (**C**) Mineralization of craniofacial skeleton by Von Kossa and Alcian Blue staining of embryos at 5 days post fecundation. Abbreviations: mx - branchio maxilla, bs - branchistegal ray, op - opercle, cl - cleithrum, pt - pharyngeal teeth, m - Meckel's cartilage, pq - palatoquadrate, ch - ceratohyal, ep - ethmoid plate, marked 1-5 - different arches. (**D**) Scheme of *Il34* floxed allele used to obtain constitutive invalidation of *IL34* in mouse by removing exons 3 to 5 under CRE recombinase activity. (**E**) Images at 15 days after birth of consequences of the constitutive invalidation of *IL34* with detail of hydrocephaly in *Il34^-/-^* mouse (left panel). And comparative of skeletons at 15 postnatal days visualized by Alizarin red / Alcian blue double staining (right panel). (**F**) MicroCT scan 3D reconstructions of skull and tibia enable to visualize growth defects (red arrowheads). (**G**) Quantification of growth defects in the different morphometric planes (a to i) in wile type (black box) *vs. Il34^-/-^* mice (red box), both treated with IK22.5 RANKL blocking antibody (blue and green boxes), or wile type mice with Sheff.5 IL34 blocking antibody (brown box). *p<0.05, **p<0.01, ***p<0.001, ****p<0.0001. The differences between the experimental conditions were assessed one-way ANOVA test. n=8 except for *Il34^-/-^* + IK22.5 (n=4).

Two zebrafish loss of function lines were generated for the single *Il34* allele, corresponding to a 23 bp deletion (mutant #1) and a 50 bp deletion with a 6 bp insertion (mutant 2) in exon 3 (**Figure 1A**; **Figure S1B-E**). In both zebrafish mutant lines, individuals at homozygous (-/-) status presented a severe growth alteration as shown in adult fish (**Figure 1B**). At 5 days post fertilization, both null mutations resulted in a poorer craniofacial skeletal mineralization (Von Kossa and Alcian blue staining) comparatively to the control (+/+) but no evident dysmorphosis (**Figure 1C**, showed example for mutation #1).

**Figure 2:**
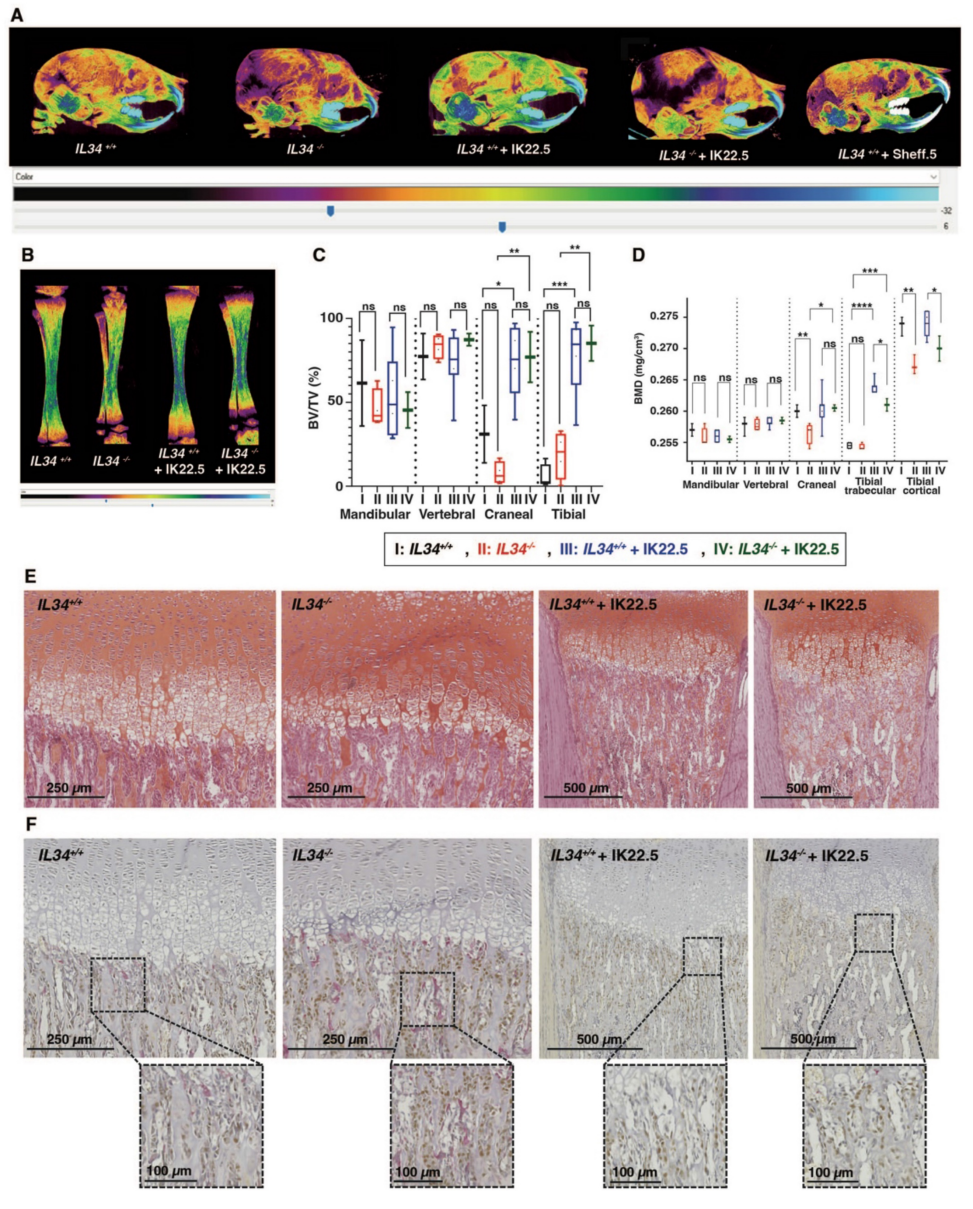
** Bone mineral and histologic alterations associated with *IL34* genetic invalidation in mouse.** (**A**) Comparative analyses of skull bones mineralization levels between *Il34^+/+^*, *Il34^-/-^*, *Il34^+/+^* injected with IK22.5 antibody, *Il34^-/-^* injected with IK22.5 antibody and *Il34^+/+^* injected with Sheff.5 antibody mice at age of 15 days, using profile views of the microCT scan 3D reconstructions. The color density ranges from black (lower mineralization) to clear blue (higher mineralization). (**B**) Comparative analyses of tibias mineralization levels between *Il34^+/+^*, *Il34^-/-^*, *Il34^+/+^* treated with IK22.5 antibody and *Il34^-/-^* treated with IK22.5 antibody mice at age of 15 days, using longitudinal views of the microCT scan 3D reconstructions. (**C**) Comparative analysis of the bone volume (BV)/total volume (TV) ratio between *Il34^+/+^*, *Il34^-/-^*, *Il34^+/+^* treated with IK22.5 antibody and *Il34^-/-^* treated with IK22.5 antibody in bone of different anatomical sites: the mandible, the vertebra, the skull and the tibia. *p<0.05, **p<0.01, ***p<0.001, ****p<0.0001, ns: not significant. n=8 except for *Il34^-/-^* + IK22.5 (n=4). (**D**) Comparative analysis of the bone mineral density (BMD) between *Il34^+/+^*, *Il34^-/-^*, *Il34^+/+^* treated with IK22.5 antibody and *Il34^-/-^* treated with IK22.5 antibody in bone of different anatomical sites: the mandible, the vertebra, the skull and the tibia. Two areas were considered for the tibia, the trabecular and the cortical. *p<0.05, **p<0.01, ***p<0.001, ****p<0.0001, ns: not significant. n=8 except for *Il34^-/-^* + IK22.5 (n=4). (**E**) Chondrocytes stained by safranin-O staining of tibia longitudinal sections at the level of the proximal epiphysis performed for *Il34^-/-^* and *Il34^+/+^* mice injected or not with the IK22.5 antibody. (**F**) Tartrate resistant acid phosphatase (TRAP) and Osterix (Osx/SP7) dual-staining of tibia longitudinal sections at the level of the proximal epiphysis performed for *Il34^-/-^* and *Il34^+/+^* mice injected or not with the IK22.5 antibody. TRAP red staining for osteoclast cells. OSX brown staining for pre-osteoblasts cells. The scales are given as bars with the corresponding values in the lower part of each histological view.

The IL34 invalidated mouse line was obtained by CRE-recombinase activation on genetically modified *Il34* gene (**Figure 1D** and **Figure S2**) with LoxP sites in introns 2 and 5 enabling to remove exons 3 to 5 while maintaining a lacZ reporter sequence located in the 5' part of intron 2 (*Il34^LacZ^* allele in **Figure S2**). The functionality of the generated *Il34^LacZ^* allele was validated in the skin (**Figure S3**), a well-known site of IL34 expression (for instance 24,25), with in mice homozygous for this allele (thereafter called *Il34^-/-^* mice), the expected absence of IL34 expression (**Figure S3A**) associated to a significant reduction of CD207^+^ Langerhans cells (**Figures S3B-D**). The LacZ reporter was also functional as attested by the ß-galactosidase staining on skin section of mice heterozygous for the *Il34^LacZ^* allele (**Figure S3E**). *Il34^-/-^* mice were phenotypically altered. Indeed, 15 days-old IL34 invalidated mice exhibited a severe growth delay and dysmorphoses in whole skeleton elements, specifically in the craniofacial skeleton associated with hydrocephaly (**Figure 1E**). MicroCT scan 3D reconstructions of skull and tibia enabled visualization of these growth defects in *Il34^-/-^* mice (**Figure 1F,** red arrowheads). Morphometric analysis evidenced significant reduction in the skull growth in all planes (sagittal, vertical and transversal) and of the long bone growth in the length and width dimensions in *Il34^-/-^* mice compared to wild type (WT) (red vs black values in **Figure 1H** and **Figure S4A**). However, a significant augmentation was observed for the middle cranial vault and no impact was reported on the cranial vault length and the inter-zygomatic root width. Interestingly, the use of a murine IL34 blocking antibody (Sheff.5 clone) during the first post-natal week in WT mouse pups (protocol described in **Figure S4B**) similarly induced skull growth alterations in all planes but to a lower extent when compared to WT mice (brown vs black values in **Figure 1H**). MicroCT scans were also used to determine bone structure parameters and bone mineral density (BMD) in various anatomical sites, namely the mandibular, the vertebral, the cranial and the tibial bones. No significant difference in the trabecular thickness (Tb.Th), the trabecular space (Tr. Sp) or the percentage of bone volume (BV/TV) was observed between *Il34^+/+^* and *Il34^-/-^* mice whichever bone was considered (**Figure S4C** and **Figure 2C**, red vs black values). On the contrary, the trabecular number (Tb.N) was significantly increased only for the vertebral bone in *Il34^-/-^* (**Figure S4C**). Injections of the Sheff.5 blocking antibody had no impact on the bone structure parameters (**Figure S5**). Regarding the bone mineral density, a significant reduction in bone mineralization was observed in the cranial and the tibial bones of *Il34^-/-^* mice compared to *Il34^+/+^* mice (**Figures 2A-B and D**). The Sheff.5 antibody transitory treatment was insufficient to induce a similar bone mineral reduction in WT mice (**Figure 2A**, **Figure S5**).

Taken together, all those data demonstrated that IL34-invalidaton during development induces important bone modifications.

### The absence of IL34 alters the osteoclast-osteoblast balance and bone homeostasis

Histological analyses on tibia sections performed at the level of the proximal epiphysis (Safranin-O staining **Figure 2E**; Masson's trichrome staining **Figure S6**) revealed an important reduction in the growth plate hypertrophic chondrocytes area (*Il34^+/+^* 0.277 ± 0.021 mm^2^ and *Il34^-/-^* 0.146 ± 0.094 mm^2^). Tartrate resistant acid phosphatase (TRAP) and Osterix (Osx/SP7) dual staining carried out by histoenzymology and immunohistochemistry respectively (**Figure 2F** and top panel **Figure S7** for higher magnification) outlined an increase of both staining corresponding to osteoclastic (red stain) and pre-osteoblastic cells (brown stain) in the null mutant comparatively to the wild-type (*Il34^+/+^*) littermate (**Figure 2F**). Interestingly, the RUNX2 immunohistochemistry staining, which enables identification of cells of the osteoblastic lineage (**Figure S8,** top panels), showed no difference in the number of stained cells between *Il34^-/-^* and *Il34^+/+^* mice, suggesting a slowdown of the osteoblast differentiation process with an accumulation of Osterix-positive pre-osteoblasts in the null mutant and without reduction of the total number of cells committed in this process.

To identify the part of the *Il34^-/-^* mouse skeleton phenotype linked to the increased number of osteoclastic cells, a RANKL blocking antibody (IK22.5) was injected during the first postnatal week to totally block the osteoclastogenesis (protocol described in **Figure S4B**). Such blockade had no consequence on the morphometric parameters in the null mutant (green vs red in **Figure 1G**) but impacted the trabecular parameters, the BV/TV and the BMD with significant differences for cranial and tibial bones (green vs red in **Figure S4C** and **Figures 2C-D**). Histological analyses on tibia sections performed at the level of the proximal epiphysis enabled visualization in the null mutant treated with the IK22.5 blocking antibody of a massive reduction of the TRAP positive cells (**Figure 2F** and bottom panels in **Figure S7**) associated with an apparent normalization of the growth plate hypertrophic chondrocytes area from 0.146 ± 0.094 mm^2^ to 0.250 ± 0.033 mm^2^ (**Figure 2E**) whereas no impact was noticed on the number of Osterix-positive (**Figure 2F** and **Figure S7**) and RUNX2-positive (**Figure S8**) cells.

Overall, these results suggested that IL34 may directly impact osteoblastic differentiation during development.

### IL34 improves BMP2 activity in osteoblastic differentiation

BMPs and TGFβs proteins are direct involved in skeletal development and bone homeostasis (nicely reviewed in 26). Interestingly, Interestingly, conditional knockout BMP2 and BMP4 mice described in the literature have a phenotype similar to our *Il34^-/-^* mouse model, with small body size and cranial and growth plate defects 27. BMPs and TGFβs signaling pathways are crucial for proper osteoclast and osteoblast differentiation and maturation by regulating key transcriptional factors as NF-kβ in bone marrow monocytes and RUNX2 in mesenchymal stem cells. In order to see if those phenotypic and histological defects observed in *Il34^-/-^* models could be due to unknown interactions between IL34 and BMPs / TGFβs members we performed *in vitro* studies to evaluate the impact of IL34 in BMPs / TGFβs signaling during osteoblastogenesis and osteoclastogenesis.

**Figure 3:**
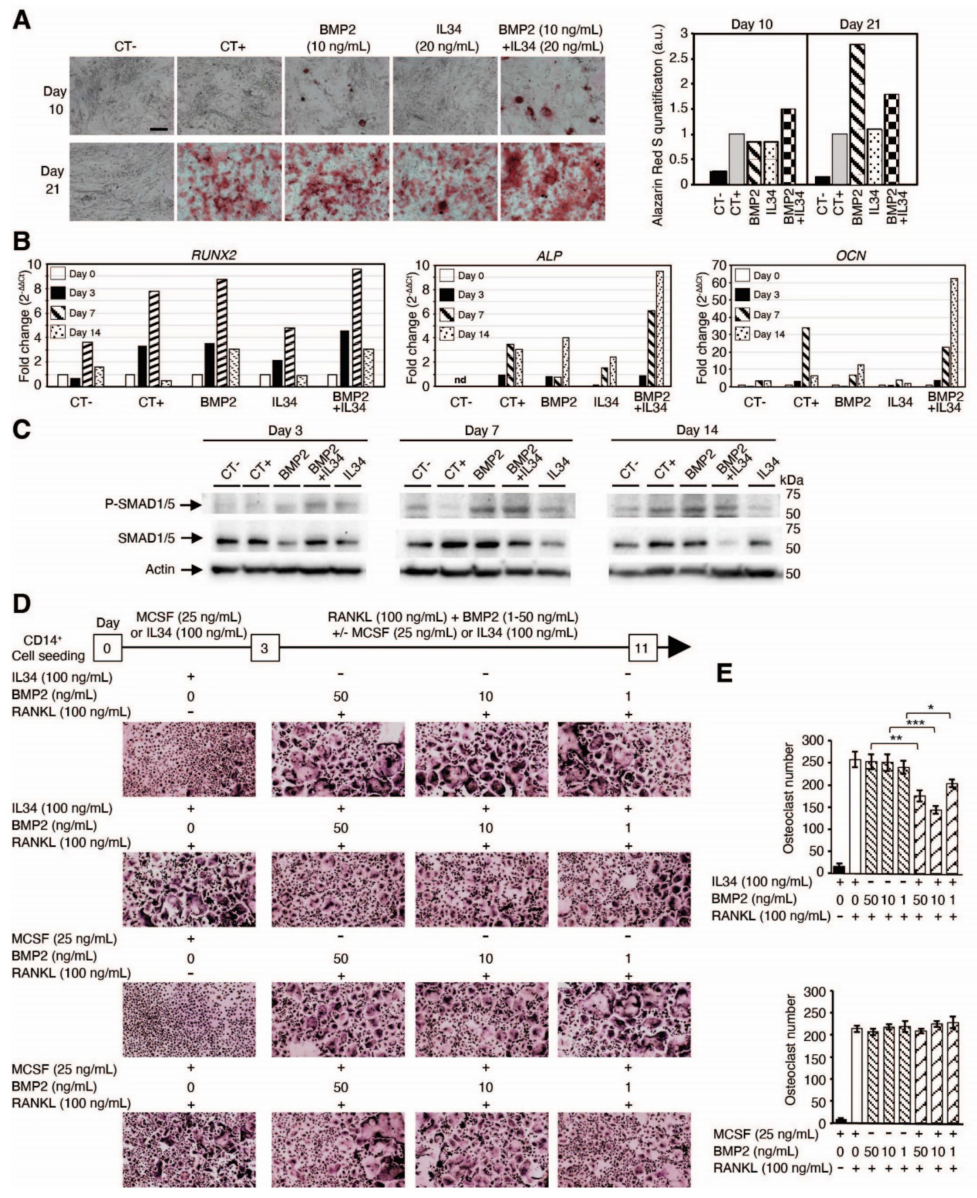
** IL34 regulates BMP2-associated osteoblastic and osteoclastic differentiation.** (**A**) Images of human mesenchymal stem cells differentiated into osteoblasts cultured in basic culture medium (CT-) or in osteogenic culture medium (CT+) in the absence or presence of BMP2 (10 ng/mL), IL34 (20 ng/mL) or combination of both at 10 and 21 days. Right panel: quantification of alizarin red staining. Magnification was similar for all views and the bar in CT- view at day 10 corresponds to 500 µm. (**B**) Real-time PCR quantification of early (*RUNX2*) and late (*ALP* and *OCN*) markers of osteoblastogenesis at days 0, 3 7 and 14. Data correspond to fold increase by 2^-ΔΔCt^ (cycle threshold) method. A representative experiment is shown. nd: non detected. (**C**) Western blot analysis of SMDA1/5 phosphorylation at different times of human mesenchymal stem cells differentiated into osteoblasts in basic culture medium (CT-) and in osteogenic culture medium (CT+) in the absence or presence of BMP2 (10 ng/mL), IL34 (20 ng/mL) or combination of both. (**D**) Differentiation of human CD14^+^ cells into osteoclastic cells analyzed by Tartrate Resistant Acid phosphatase activity (TRAP histoenzymology: purple staining) after 3-day culture period in the presence of MCSF (25 ng/mL) or IL34 (100 ng/mL), followed by an 8-day period of maturation with the addition of RANKL (100 ng/mL) and /or BMP2 addition (concentrations from 1 to 50 ng/mL) (**E**) Quantification of the different experiments repeated in triplicate and presented in **D**. At least two independent experiments have been carried in triplicate. *p<0.05, **p<0.01, ***p<0.001.

*In vitro* human mesenchymal stem cell differentiation into osteoblasts was induced by a standard osteoblastic differentiation medium (composition described in the Methods section). This differentiation, quantified by the phosphocalcic mineral deposition (alizarin red staining). and the expression levels of differentiation markers (*RUNX2*, *ALP* and *OCN*), was accelerated by addition of the bone morphogenic protein 2 (BMP2) at 10 ng/mL as shown in **Figure 3A-B**, **Figure S9** and **Figure S10**. The addition of IL34 alone (20 ng/mL) to the differentiation medium had no impact on the rate of osteoblastic differentiation, but interestingly it was able to potentiate the effect of BMP2 when added in combination with an optimal IL34/BMP2 concentration ratio (ng/mL) of two (**Figure 3A**). This concentration ratio corresponded to an equal amount in molarity of the two cytokines (**Figure S11A**). The combination of both molecules resulted in an earlier formation of calcium phosphate crystals (identifiable by alizarin red staining) (**Figure 3A** and **Figure S9**). No mineralization was observed in osteoblasts cultured in basic culture medium (CT-) neither in the presence alone or in combination of BMP2 and IL34 (data not shown). The expression levels of osteoblast differentiation markers were in agreement with the alizarin red staining. Indeed, the combination of both molecules impacted the expression of early (*RUNX2*) and late (*ALP* and *OCN*) markers of osteoblastogenesis in the way of a more rapid differentiation clearly visible from day 3 for *RUNX2* and at day 14 for *ALP* and *OCN* (**Figure 3B** and **Figure S10**). These data suggest a potentiation of BMP2 functions induced by IL34.

**Figure 4:**
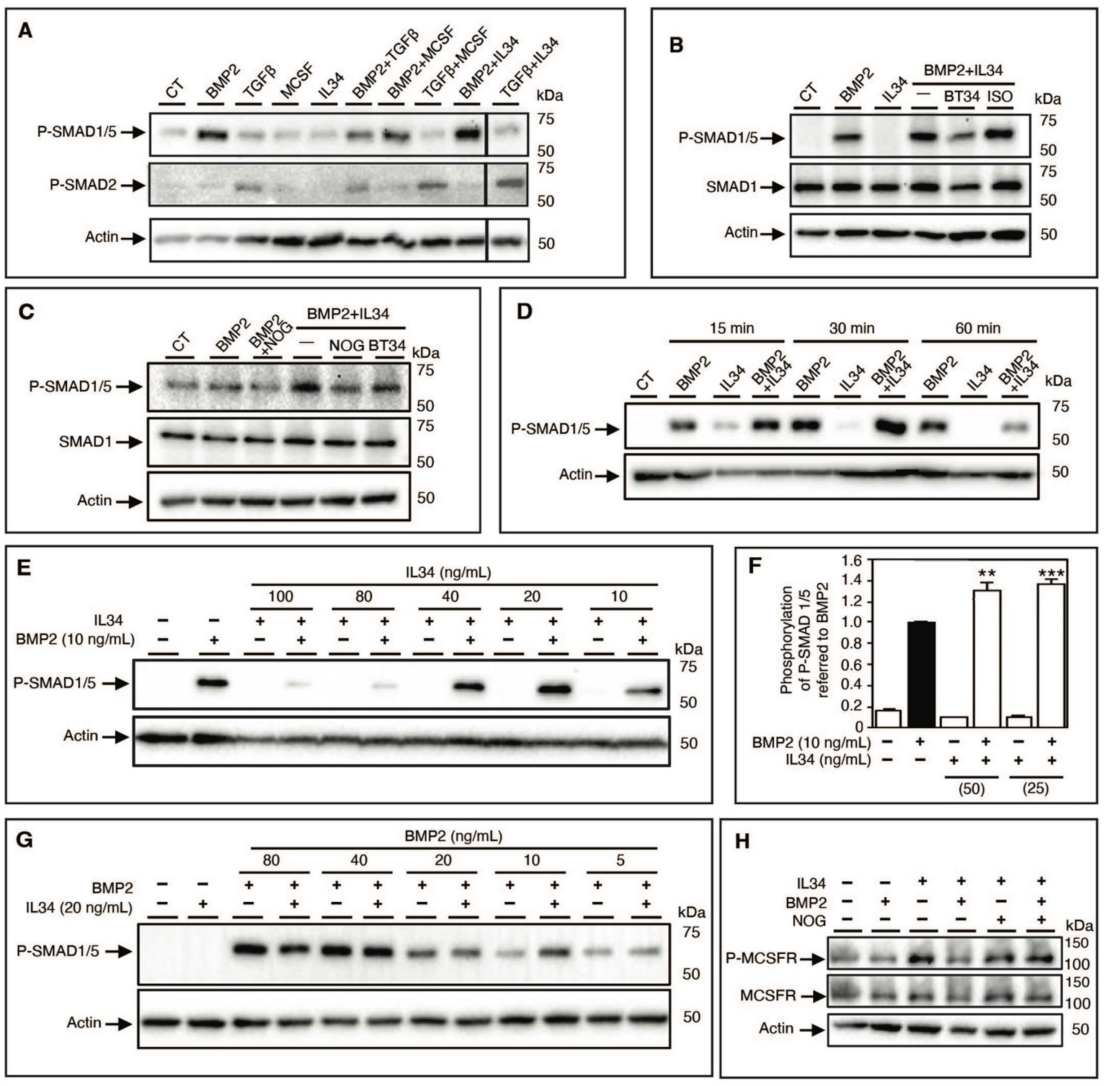
** The interaction IL34-BMP2 modulates SMAD1/5 as well as MCSF receptor (MCSFR) phosphorylation and related signaling.** (A) Western blot analysis of SMDA1/5 and SMAD2 phosphorylations of human MNNG-HOS osteosarcoma cells in the presence of BMP2 (10 ng/mL), TGFß (10 ng/mL), MCSF (20 ng/mL), IL34 (20 ng/mL) alone or in corresponding combination. A representative experiment is shown. CT: basic culture medium. (B) Western blot analysis of SMDA1/5 phosphorylation of human MNNG-HOS osteosarcoma cells in the presence of BMP2 (10 ng/mL), IL34 (20 ng/mL) alone or in combination (BMP2+IL34, -) plus the human IL34 blocking antibody (BT34) (100 µg/mL) or its irrelevant isotypic control antibody (ISO) (100 µg/mL). CT: basic culture medium. (C) Western blot analysis of SMDA1/5, similar conditions used in B in the presence of the human IL34 blocking antibody (BT34) (100 µg/mL) or the natural inhibitor of BMP2 called NOGGIN (NOG) (200 ng/mL). (D) Kinetic analysis by Western blot of the potentiating effect of IL34 on BMP2-induced SMAD1/5 phosphorylation at 15 min, 30 min and 60 min with similar corresponding molecules concentrations described in B. (E) Western blot analysis of SMDA1/5 as described in B in the presence of a single concentration of BMP2 (10 ng/mL) in combination with gradual quantities of IL34 (10, 20, 40, 80 and 100 ng/mL). (F) The Alpha SureFire technology (Revvity) was used to quantitatively validate the potentiation effect of IL34 on BMP2 activation of SMAD1/5 phosphorylation. Co-additions of 25 or 50 ng/mL of IL34 increased significantly the phosphorylation of SMAD1/5 induced by the addition of BMP2 at 10 ng/mL. **p<0.01, ***p<0.001. (G) Western blot analysis of SMDA1/5 as described in B with a single concentration of IL34 (20 ng/mL) in combination with gradual quantities of IL34 (5, 10, 20, 40 and 80 ng/mL). (H) Western blot analysis of MCSFR phosphorylation expressed in HEK293 transfected cells in the presence or absence of BMP2 (10 ng/mL), IL34 (20 ng/mL) or in combination (BMP2+IL34, -) plus NOGGIN (NOG) (200 ng/mL). Quantifications of all the Western blots presented in this figure are shown in Figure S12. All experiments have done at least three times independently.

To validate this hypothesis, the canonical BMP signaling pathway was analyzed by Western blots in human mesenchymal stem cells. IL34 treatment resulted in an increased and earlier phosphorylation of the SMAD1/5 proteins observed in the presence of BMP2 compared to each molecule alone (Figure 3C). Due to the amount of human mesenchymal stem cells required for Western blot analysis, we decided to use an osteoblastic human osteosarcoma cell line (MNNG-HOS). This cell line recapitulated the same effect in SMAD1/5 phosphorylation of IL34 treatment in the presence of BMP2 (**Figure 4A**), BMP4 and BMP7 (**Figure S11B-E**), whereas IL34 has no similar impact on the phosphorylation of the SMAD2 protein induced by the TGFβ (**Figure 4A**, **Figure S12**). Interestingly, the potentiation effect of IL34 was blocked by the use of a specific human-IL34 blocking antibody named BT34 (**Figure 4B**, **Figure S12**), and the blocking of BMP2 pro-differentiation signaling with its natural inhibitor NOGGIN was annihilated by the presence of IL34 (**Figure 4C**, **Figure S12**). The potentiation effect of IL34 was moreover rapid (**Figure 4D**, **Figure S12**) and as previously mentioned sensitive to the ratio between the two cytokines (**Figure 4E-G, Figure S12**). The combination of BMP2 at 10 ng/mL to IL34 at 20 or 40 ng/mL induced higher SMAD1/5 phosphorylation than those observed with BMP2 alone, while the combination of BMP2 at 10 ng/mL and IL34 at 80 or 100 ng/mL significantly reduced SMAD1/5 phosphorylation (**Figure 4E**, **Figure S12**). The addition of IL34 at 20 ng/mL to BMP2 at 5, 10 or 20 ng/mL induced higher SMAD1/5 phosphorylation than those observed with BMP2 alone, while the combination of IL34 at 20 ng/mL and BMP2 at 40 or 80 ng/mL decreased SMAD1/5 phosphorylation (**Figure 4G**, **Figure S12**). This observation supported the existence of a physical and strong functional interaction between IL34 and some proteins of the BMP family.

To complete the evidence on a physical interaction between IL34 and members of the BMP protein family, the potential impact of BMP2 addition on the IL34-induced osteoclastogenesis was evaluated *in vitro*. Differentiation of human CD14^+^ cells into osteoclasts can be achieved by a two-step protocol corresponding to a 3-days culture period in the presence of MCSF (25 ng/mL) or IL34 (100 ng/mL) to the culture medium, followed by an 8-days period in presence of MCSF or IL34 combined to RANKL (100 ng/mL). Osteoclasts were identified in the culture by their expression of the TRAP activity (TRAP histoenzymology: purple staining). As previously shown 28, BMP2 addition to the cell cultures (concentrations from 1 to 50 ng/mL) may replace either MCSF or IL34 during the second period. However, while the combined addition of BMP2 (concentrations from 1 to 50 ng/mL) and MCSF to RANKL had no impact on the osteoclastogenesis, the combined addition of BMP2 and IL34 to RANKL induced a reduction of the number of osteoclasts formed (**Figure 3D-E**). Furthermore, the phosphorylation of MCSFR in response to IL34 was inhibited in presence of BMP2 and this inhibition was reversed by addition of NOGGIN supporting the existence of a functional physical link between IL34 and some members of the BMP protein family (**Figure 4H**, **Figure S12**).

### Physical interactions between IL34 and some members of BMPs and receptors

**Figure 5:**
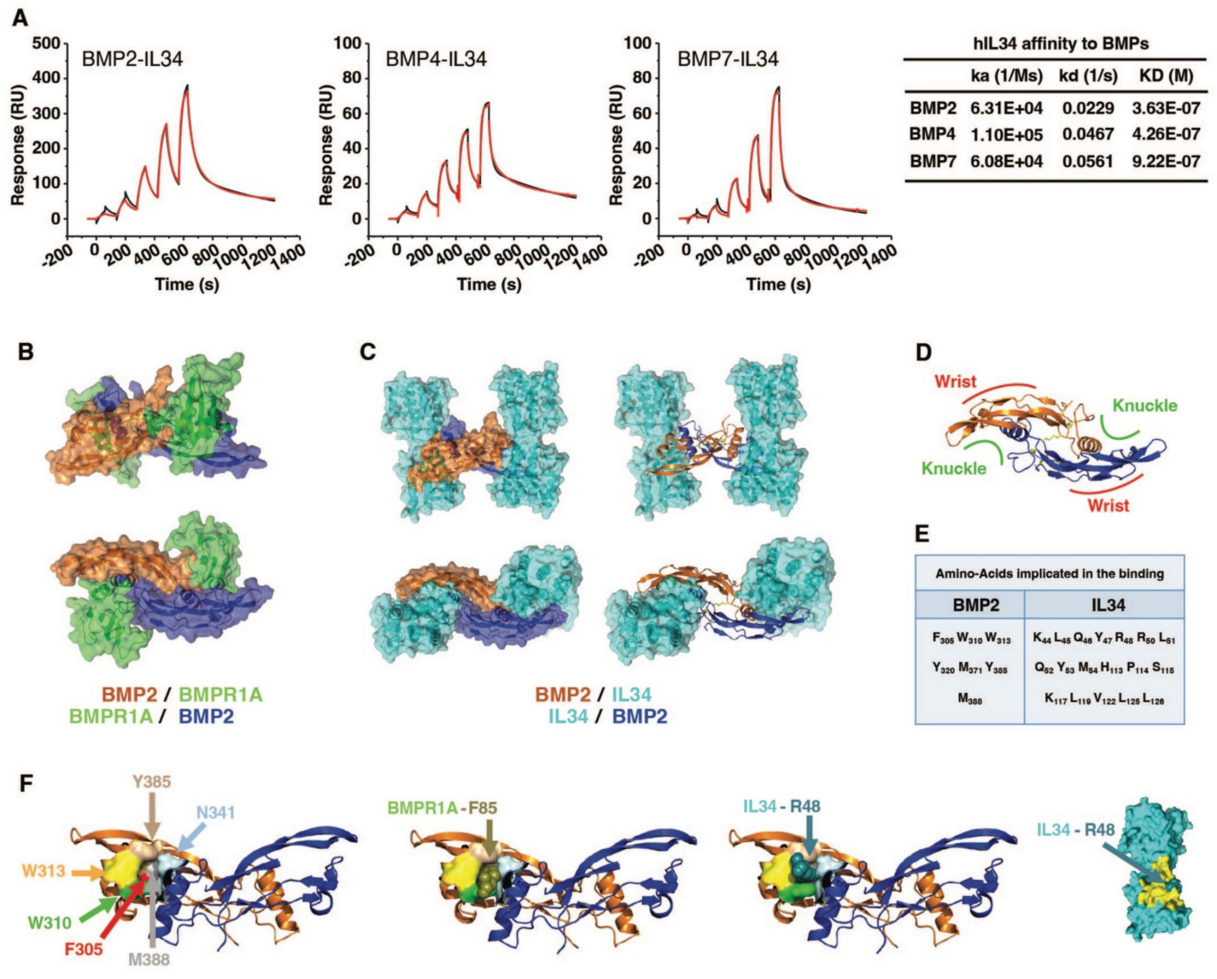
** Demonstration and deciphering at the molecular level of the physical interaction between the IL34 protein and proteins of the BMP family.** (**A**) Surface plasmon resonance experiments (described in Materials and Methods section) and values of proteins interaction parameters between IL34 and BMPs. ka: association rate constant, kd: dissociation rate constant, KD: affinity constant. (**B**) Molecular modelling of the binding of two BMPR1A receptors (green) to a BMP2 dimer (brown and dark blue) by using PyMOL. (**C**) Molecular modelling of the binding of two IL34 proteins (cyan) to a BMP2 dimer (brown and dark blue) seen in profile (top) and from above (bottom) with a representation of the BMP2 proteins in surface (left) and in structure (right) by using PyMOL. (**D**) Structural representation of a BMP2 dimer seen from above with the location of the “Knuckle” and “Wrist” binding sites to the type 1 and type 2 receptors respectively as described by Sebald and collaborators 76,77. (**E**) Main amino acid of BMP2 and IL34 identified as being involved in binding. In addition, hydrogen bonds and salt bridges were found between BMP2 and IL34, more specifically between residues K383-D190, D312-K55 and E376-R73. (**F**) Localization on the representation of the BMP2 protein in structure of amino acids important for partner binding: F305 in red, W310 in bright green, W313 in yellow, Y385 in light brown and M388 in grey. These amino acids delineate the pocket in which residues F85 of BMPR1A and R48 of IL34 are positioned during their interaction with BMP2. The amino acid N341 presented in light blue, despite is localization in the most outside part of the pocket, was not identified as important for the binding to IL34. IL34 is displayed in surface representation with the entire binding region colored in yellow, and the important intercalating residue R48 is indicated in duck blue.

To definitively establish the physical interaction between IL34 and BMPs, surface plasmon resonance experiments were performed and demonstrated effective binding of IL34 to BMP2, BMP4 and BMP7 with KD values of 3.63E-07 M, 4.26E-07 M and 9.22E-07 M respectively (**Figure 5A** and **Figure S13**).

**Figure 6:**
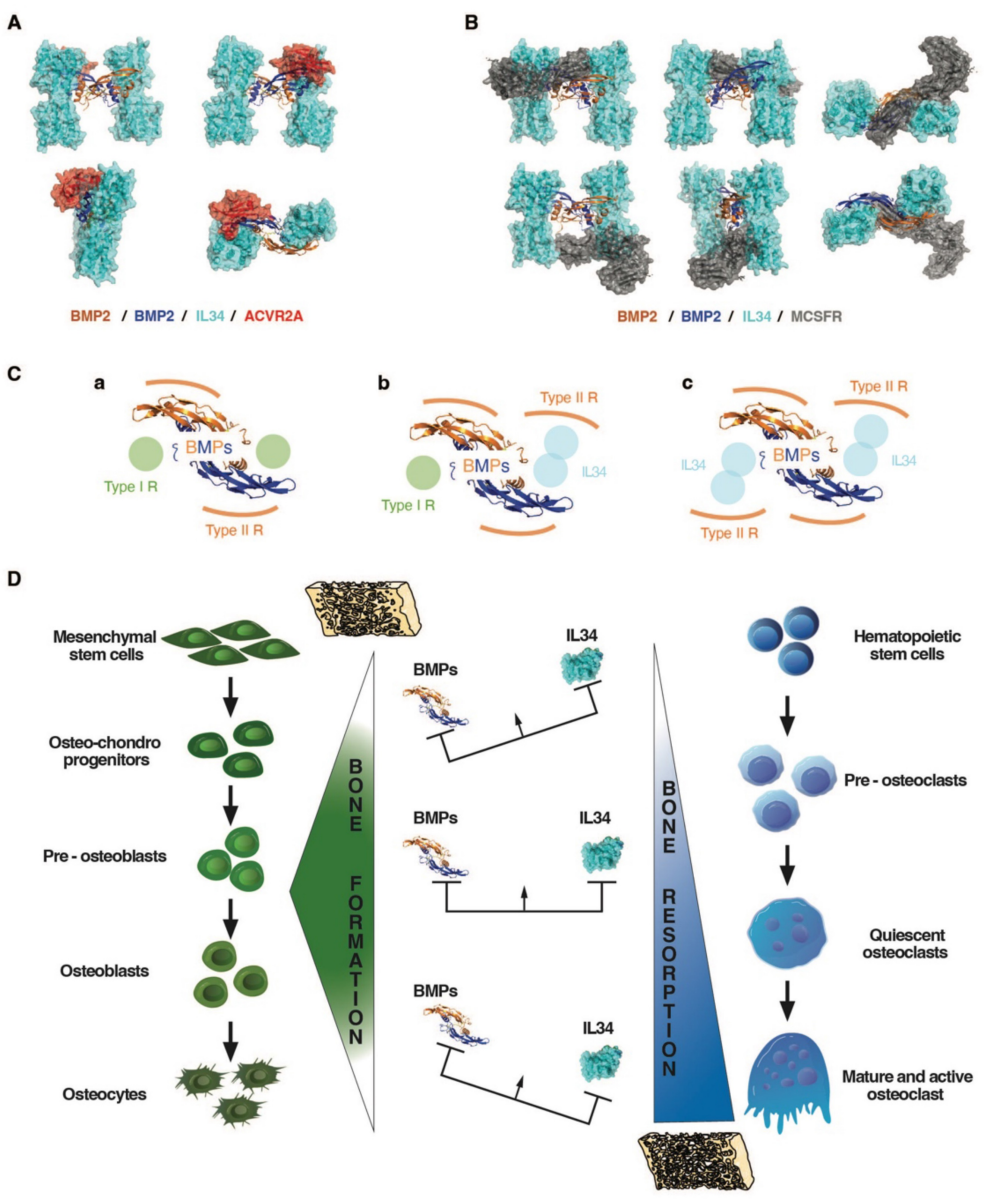
** Impacts of the binding between BMP2 and IL34 on the ability of BMP2 and IL34 to bind to ACVR2A and MCSFR receptors respectively: importance of stoichiometry and functional consequences on bone formation and resorption.** (**A**) ACVR2A receptor binding to BMP2 (“Wrist” site) does not appear to be affected by IL34 binding to the “Knuckle” sites of a BMP2 dimer. (**B**) MCSFR receptor binding to IL34 occurs at a site overlapping the BMP2 “Knuckle” site binding site. Simultaneous binding of BMP2 and MCSFR to IL34 is therefore impossible. (**C**) BMP receptor binding stoichiometry to a BMP2 dimer. The standard binding of two type 1 and two type 2 receptors per dimer (a), is gradually modified by the amount of IL34 present with potential transformation of a “Knuckle” site into a “Wrist”-like site at intermediate concentrations (b), then a second at high IL34 concentrations (c), bearing in mind that IL34 can bind type 2 BMP receptors. (**D**) Schematic representation of the impact of different ratios of BMPs and IL34 on bone formation and resorption.

A molecular modelling approach (see Materials and Methods), corresponding to a protein-protein docking study, established that IL34 binding to BMP2 occurred at the “Knuckle” sites of the BMP2 dimers known to correspond to the binding sites of BMP type 1 receptors and did not impinge on the “Wrist” sites that correspond to the binding sites of BMP type 2 receptors (**Figure 5B-D**; **Figure S14**). The amino acids of BMP2 and IL34 involved in binding were identified (**Figure 5E** and **Figure S15**), with BMP2 involving a pocket (formed by F305, W310, W313, Y385 and M388) in which the phenylalanine in position 85 for BMPR1A (F85) or the arginine in position 48 for IL34 (R48) are positioned during their respective interactions (Figure 5F). It is important to note that the amino acids involved are phylogenetically highly conserved in both BMP2 and IL34 and that, in addition, the amino acids of BMP2 implicated are also found conserved in several members of the BMP family (**Figure S16**). It was therefore possible to model the binding to IL34 of certain BMPs for which crystallographic structures were available, such as BMP3, BMP6 and BMP7 (**Figure S17**). The existence of direct physical links between BMP proteins and IL34 having been established, the question of the consequences of these links on the binding of BMPs to their receptors on the one hand and the binding of IL34 to MCSFR on the other was raised. Binding of BMP2 to the type 1 BMP receptor is hindered by IL34, which binds to the same site as shown above. It should be noted that this “Knuckle” site is also the binding site for co-receptor proteins of the RGM family (**Figure S14C**) and that it is partially masked by NOGGIN binding (**Figure S14D**). With regard to the binding of BMP2 to type 2 BMP receptors, modelling shows that binding of the ACVR2A receptor, for example, is entirely possible on a BMP2 dimer with two IL34 binders (**Figure 6A**), the “Wrist” sites not being masked by the presence of IL34. These different possibilities for binding type 1 and 2 receptors and IL34 to a BMP2 dimer are shown in 3D in **Movie S1**. Concerning the binding of IL34 to MCSFR, the binding of IL34 to BMP2 occurs at a site that overlaps with the binding site of MCSFR to IL34 (**Figure 6B**), preventing the simultaneous binding of MCSFR and BMP2 to IL34.

## Discussion

IL34, one of the latest cytokines identified 29, has been shown to bind to a variety of receptors with consequences for the differentiation and activation of myeloid, neural and glial cells (For review 4,5). Surprisingly, the implications of this cytokine during development and growth had not been addressed in detail, unlike those of its receptor MCSFR (for review 30), although its ability to stimulate differentiation of osteoclasts, cells important for skeletal growth, had been established via binding and activation of this receptor 13,14,31. The primary aim of the work presented here was to determine these implications by generating two *in vivo* models of IL34 invalidation, one in zebrafish and the other in mice, and to characterize the associated skeletal phenotypes. Both models showed significant growth retardation, with reductions in cartilage mineralization in zebrafish and bone mineralization in mice. Concerning bone mineral density (BMD), the observation in *Il34^-/-^* mice of a significant reduction of this parameter only in certain bones raises questions. Interestingly, a dichotomy was observed between bones with endochondral mineralization (mandible, vertebrae, tibia trabecular zone (metaphyseal)) and those with intramembranous mineralization (cranial bone, tibia periosteal zone (diaphyseal)), the latter being the only ones to show a reduction in BMD. To decipher the molecular basis of such a difference, further studies will be required, focusing on the expression of all the factors involved in these two types of mineralization (matrix proteins, nucleation factors, enzymes, etc.).

In mice invalidated for IL34, obtained at an expected frequency (Mendelian inheritance) but with a reduced life expectancy (3 weeks), significant craniofacial dysmorphoses were observed with the presence of hydrocephalus. Such defects in craniofacial development are consistent with the previously established implications of IL34 in neural and microglial cells differentiation and activation 24,32-38. This model should therefore provide a useful additional tool for deciphering the precise functions of IL34 during normal and pathological development of the central nervous system.

Histological study of bone tissue from *IL34*-invalidated mice revealed a marked increase in the number of osteoclasts in the growth plate, in contrast to the phenotype envisaged for the loss of a factor known to stimulate osteoclastogenesis 10,12,13. As this increase in osteoclast numbers was associated with an accumulation of pre-osteoblasts (OSX-positive) and a reduction in the hypertrophic zone of the growth plate, the question of a role for IL34 in the differentiation of osteoblasts and chondroblasts was raised. To check whether these two points were simply not secondary to the increase in osteoclastogenesis, the consequences of inhibiting RANKL (a factor essential to osteoclastogenesis) during the first week of life in *Il34^-/-^* mice were analyzed. No impact was observed on the accumulation of OSX-positive cells, while normalization of the size of the hypertrophic zone was observed. These results suggest that IL34 may directly regulate osteoblastic differentiation and probably indirectly that of chondroblasts via osteoclasts, bearing in mind that in inflammatory situations, both mature osteoblasts and hypertrophic chondrocytes can become important sources of pro-osteoclastic IL34 16,39,40. Interestingly, a relationship has already been observed between the level of osteoclastic activity and the size of the hypertrophic zone of the growth plate, and vice versa. Thus, a decrease in the hypertrophic zone goes hand in hand with an increase in osteoclastic activity 41,42 and an increase in this zone with a decrease in osteoclastic activity 43-47. It should then be noted that the disruption of one or other of the elements in this relationship, over and above the repercussions on the other, induces growth retardation in all cases, as has been reported in patients with disorders of osteoclastogenesis (for example, in patients suffering from juvenile osteoporosis 48 or osteopetrosis 49) as well as in those with chondrodysplasia (for review 50). The increase in osteoclasts observed in *Il34^-/-^* mice, appropriately associated with a reduction in the hypertrophic zone of the growth plate, could therefore explain the growth retardation. Establishing the origin of the increase in osteoclasts in relation to the absence of IL34 was not immediately obvious. Osterix expression marks pre-osteoblasts, which are known as an important source of RANKL during growth 51,52. The accumulation of OSX-positive cells at the subchondral level in *Il34^-/-^* mice could explain the increased number of osteoclasts, taking into account that an analysis of the number CD11b-positive cells (osteoclast precursors) in the bone marrow and spleen of *Il34^-/-^* mice reveled a marked increase (**Figure S18**). The question then arose as to the origin of the accumulation of these OSX-positive pre-osteoblasts in *Il34^-/-^* mice, given that the total number of cells committed to osteoblastic lineage according to RUNX2 labeling did not appear to be affected. An impact of IL34 absence on osteoblastogenesis was therefore strongly suspected.

Members of the TGFβ-BMP family, in particular BMP2, are major stimulators of osteoblastogenesis (for review 26). The co-addition of IL34 with BMP2 in the culture medium of mesenchymal stem cells undergoing osteoblastic differentiation has shown, for certain ratios, a potentiation of the effect of BMP2 on this differentiation. Protein binding studies showed that IL34 could bind directly to BMP2, and 3D modeling identified the amino acids involved in this binding in the sequences of IL34 and BMP2. With regard to the BMP family, the amino acids involved in IL34 binding were found to be highly conserved, and the veracity of the direct binding of BMP4 and BMP7 to IL34 was established, suggesting that IL34 may potentiate the effects of several family members. IL34 binds to the "Knuckle" site of BMP2, which is also the binding site for type 1 receptors to BMPs, without obscuring the "Wrist" binding site for type 2 receptors. Protein binding studies have also shown that IL34 can directly bind type 2 receptors to BMPs (**Figure S19**), enabling it to occupy the "Knuckle" site of a BMP and transform it into a "Wrist"-like site. A biphasic mechanism of action associated with IL34 binding to BMP dimers can then be proposed (**Figure 6C-D**), corresponding to the progressive modification of the ratio between type 1 and 2 BMP receptors. Thus, in the absence of IL34, basal activity is observed with a receptor ratio of 2/2. Then with an amount of IL34 equivalent to that of BMP2, maximum activity is observed corresponding to a receptor ratio of 1/3. Finally, with an excess of IL34, zero activity is observed with a receptor ratio of 0/4. Interestingly, several studies have reported that in an inflammatory context, BMP2 could inhibit IL34 expression 53-55, suggesting the possible existence of a feedback loop of IL34 potentiation of BMP2 activity. The ratio of IL34 to BMP2 has also been shown to impact IL34 binding to the MCSFR so the osteoclastogenesis in bone. IL34 thus appears to play a key role in bone formation, modulating both osteoclastogenesis via its direct binding to the MCSFR and osteoblastogenesis via its binding to BMPs.

In a more general context, IL34's ability to directly control MCSF receptor activation and indirectly BMP receptors activation defines it as a major player in the development, growth, homeostasis and function of most organs. Further studies will obviously be needed to determine which members of the BMP family are IL34 partners in each organ, in normal physiology and pathological situations (for review 56) including cancers for which IL34 is already presented as a therapeutic target of major interest 57-59.

## Materials and Methods

### *In vivo* experiments

All zebrafish (*Danio rerio*) used for this project were located in the aquaria at the Bateson Centre, at the University of Sheffield (UK). Zebrafish were present in tanks at a density of no more than four zebrafish per liter, with 14 hours light and 10 hours dark cycle, at a temperature of 28 °C. All experimental procedures were carried out in accordance with the UK Home Office Project License PPL70/8178 and personal license IO6008638. All transgenic mice (*Mus Musculus*) used for this project were housed under pathogen-free conditions at the Experimental Therapy Unit at the Faculty of Medicine of the University of Nantes, France (Agreement D44015 and DUO 6781). All protocols applied in the present study were first validated by the French Ethical Committee of the “Pays de la Loire” (CEEA-PdL-06) and authorized by the French Ministry of Agriculture and Fisheries (authorization # 18415-201901101823350 v2).

### Generation of IL34 mutant zebrafish

The zebrafish *Il34* gene (ENSDARG00000091003.2 or ZDB-GENE-050419-150) contains seven exons as human and mouse genes (**Figure 1**). IL34 mutant zebrafish was generated using the CRISPR-Cas9 technology as previously described 60,61. Exon 3 was targeted using the sequence shown in **Figure S1A** and the corresponding 20 bp spacer region was placed into a guide RNA template for in *vitro* transcription. The gRNA was then transcribed using the MEGAshortscript T7 kit (Life Technologies, UK) and microinjected with Cas9 protein (NEB, UK) into the yolk of zebrafish embryos the one cell stage. F0 adult fish were crossed with wild-type fish to identify founder with germline transmission. Primers used for genotyping were (Fw 5'-TCA GCC AAT AAA TAT CAG ATC CA-3' and Rv 5'-CGT CTC CTG GTT GCA TTT-3') which amplify a 300 bp fragment of the WT sequence of zebrafish IL34 exon3 covering the chosen CRISPR target sequence. Obtained fragments of shorter sizes were sequenced to identify mutations induced in the different founders. Two mutations corresponding to a 23 bp deletion (mutant #1 in **Figure 1**) and a 50 bp deletion combined to a 6bp insertion (mutant #2 in **Figure 1**) were obtained. Phenotypes of zebrafishes homozygous for each of these mutations (*Il34^-/-^* from F3 or following generations) were compared to ensure for link to *Il34* deficiency and not from potential background mutations. Genotyping was performed on DNA extracted from the caudal fins by PCR using same primers as those used to identify founders. Fragments of 300 bp, 277 bp and 256 bp were amplified respectively for *Il34* exon 3 WT, mutant #1 and mutant #2 sequences. Animals were studied at 5 days post fertilization or at 3 months after birth.

### Van Kossa and Alcian Blue staining of zebrafish skeleton

For Von Kossa staining, samples were fixed in 4% PFA for 2 h at room temperature, rinsed in water containing 0.01% tween 20, and left to incubate in a solution of silver nitrate under a 60 W light bulb for 1 h. After rinsing with water containing 0.01% tween 20, samples were fixed in 2.5% sodium-thiosulfate for 10 min, rinsed and again fixed in 4% PFA for 30 min at room temperature. Preservation was done in glycerol, and samples were kept at room temperature in dark until images were taken.

For Alcian Blue Staining, samples were fixed overnight in 4% PFA at 4 ºC. After several washes in a phosphate buffer solution containing 0.1% tween 20 (PBS-T) and dehydration using methanol, samples were transferred into Alcian blue staining solution (0.1% Alcian Blue, 70% ethanol, 1% concentrated hydrochloric acid) and left to stain overnight at room temperature. Samples where then rinsed in PBS-T and bleached in 30% hydrogen peroxide for 10 min at 37 ºC. A 30% saturated borate solution was then used to eliminate all residues of bleaching solution before putting the samples into a trypsin digestion solution for 30 min at 37 ºC until brains and eyes appeared translucent. A rehydration was performed, and samples were put in glycerol for preservation until images were taken.

Zebrafish were imaged for both stains using the SMZ1500 stereomicroscope, with a DS-Fi1 camera (both Nikon, Japan), at 20 X magnification and Nikon Elements software.

### Generation of *Il34* mutant mouse

The *Il34* mutant mouse was generated at the Mouse Clinical Institute (IGBMC, Illkirch, France; Project IR00004258 / K4258) by classical embryonic stem cells (ES) injection in blastocyst stage embryo. Three JM8.N4 ES cell clones carrying the targeted Il34^tm1a(EUCOMM)Wtsi^ allele were purchased at the European Conditional Mouse Mutagenesis Consortium (EUCOMM) and the clone EPD0146_4_F02 (embryonic stem line JM8.N4; C57BL/6) that was confirmed by PCR and Sanger sequencing (**Figure S2**) as being correctly targeted was used to generate the *Il34* conditional mutant mouse line. Breeding with ERT2-Cre mice (B6.Cg-Tg(UBC-cre/ERT2)1Ejb/J, JR#8085, Jackson Laboratory, Bar Harbor, Maine, USA) enabled to (Tamoxifen dependently) delete exons 3-5 of *Il34* and the neomycin-resistance cassette generating the Il34^+/LacZ^ mice (**Figure S2**). Breeding with CAG-FLPe mice (C57BL/6-Tg(CAG-flpe)16Ito, RBRC10707, RIKEN BRC, Tsukuba, Ibaraki 305-0074, Japan) allowed to delete the whole LacZ-NeoR cassette and generate mice carrying a loxP-flanked *Il34* allele (*Il34^+/f^*). Homozygous *Il34^LacZ/LacZ^* mice (called *Il34^-/-^* in the manuscript) were used for analysis. Mice were genotyped by PCR (**Figure S2**) with the primers Il34-S2: 5'-GTC AGT ATC GGC GGA ATT-3', Il34-S3: 5'-GTT TGG CCG ATG CTG GCA AAG G-3' and Il34-AS2: 5'-CTG TCT TAT GAA GAT GGC ATG CC-3'. Il34-S2 and Il34-AS2 primers enable to amplify a 440 bp fragment in presence of *Il34^LacZ^* allele, and Il34-S3 and Il34-AS2 primers fragments of 240 bp and 290 bp respectively in presence of wild type (WT) and *Il34^f^* alleles (**Figure S2**).

### Alizarin Red and Alcian Blue double staining of mouse skeleton

The whole-mount skeletal staining protocol used is derived from the protocol of Rigueur and Lyons 62. Briefly, after euthanasia, all skin, internal organs, adipose tissue and as much as possible muscle were removed before fixation in a PBS 1X pH 7.4 solution containing 2% of paraformaldehyde and 0.2% glutaraldehyde. Skeletons were then dehydrated in ethanol and placed in acetone for permeabilization. Cartilage staining was then realized by submerging the skeletons in the Alcian blue stain (Alcian blue 8GX 0.03% (w/v), 80% EtOH, 20% glacial acetic acid). After washes in 70% and 95% ethanol, a pre-clear of the tissue was realized in a 1% KOH solution. Bone staining was then carried out in Alizarin red stain (Alizarin red 0.005% (w/v) in 1% (w/v) KOH). The Alizarin red solution was then replaced with a v/v mix of glycerol and 1% KOH to remove the excess red color. Skeleton were transferred to 100% glycerol for long-term storage and imaging.

### New-born mice treatment with blocking antibodies

The protocol used to treat newborn mice with blocking antibodies was previously described 46. Briefly, newborn C57BL/6 mice from naïve and transgenic *IL34^+/LacZ^* mothers received four subcutaneous injections (25 mg/kg of body weight) of respectively Sheff-5 (rat anti-mouse IL34 blocking IgG1 antibody, Diaclone, Besançon, France) and IK22-5 rat anti-mouse RANKL blocking IgG2a antibody 63 or isotopic corresponding control every 2 days beginning at day 1 after birth (**Figure S4B**). The mice were finally sacrificed at postnatal day 15 for phenotyping.

### Micro-CT analysis

A Skyscan 1076 micro-CT scanner (Skyscan, Kontich, Belgium) was used to analyze and compare between the different groups of mice (at 15 days postnatal and n=8 for each group except for *Il34^-/-^* + IK22, n=4) the bone morphometric, structural and mineral parameters at different anatomical sites namely the tibia, the mandible, the vertebra and the cranium. All samples were scanned using the same parameters (pixel size 9 μm, 50 kV, 0.5 mm Aluminum filter, 20 min of scanning). The scanner reconstruction was carried out using the NRecon software and the analyses were performed using CTAn, CTVox, and DataViewer software (Skyscan). In order to obtain the different measurements, the IMAGE-J software (National Institutes of Health, Bethesda, MD, USA) was used. In this way, the acquisition of the image in CTVox was systematically calibrated with a phantom of 5 mm (known size) and all measurements were finally sized using the analysis scale in the IMAGE-J software.

Bone morphometric parameters including tibia total length and width were sized using specific reference marks (**Figure 1C** and **Figure S4A**), and for the cranium measurements were made using the method previously described 64. Briefly, seven measurements regarding the sagittal, vertical and transversal planes of craniofacial growth were made (**Figure 1C** and **Figure S4A**).

Bone mineral and structural parameters including the bone mineral density (BMD), the percentage of bone volume (BV/TV), the trabecula thickness (Tb.Th), the trabecula separation (Tb.Sp) and the trabecula number (Tb.N) were analyzed for each bone at different anatomical sites using a volume of interest (VOI) measuring 2.0 mm x 1.1 mm × 1.1 mm. The VOI was sectioned using the Data Viewer software and analyzed using the CTAn software. The different points chosen for the analysis are presented in **Figure S4**. To facilitate the identification of changes in the different structures, a “color density range” was used in the CTAn software that made it possible to adjust the correspondence of color and brightness values using image gray scales. For tibia and head images, a brightness level of -32 and a contrast level of 6 from the color density range of the CTAn software were systematically used.

### Histology, histoenzymology and immunohistochemistry

Histology, histoenzymology and immunohistochemistry were performed on 3 µm thickness paraffin embedded sections of the different samples prepared as previously described 65. Masson's trichrome and Safranin-O stains were performed following classical protocols and tartrate-resistant acid phosphatase (TRAP) histoenzymology was carried out as previously described 66. Immunohistochemistry was performed by using the protocol as previously described 67 and the following antibodies: rabbit monoclonal anti-RUNX2 (Abcam, ref#ab192256, 1/1000), rabbit polyclonal anti- osterix (OSX) (Abcam ref#ab22552, 1/1000), anti-CD207 (eBioscience, ref# 14-2073-80, 1/100).

### LacZ staining

Sections (12μm) of *IL34^+/LacZ^* mice epidermis embedded in OCT were cut using Cryostat Leica CM3050S. Slices were fixed with PFA 1% 5 min, rinsed with PBS 1x and incubated in Xgal (5-bromo-4-chloro-3-indolyl-beta-D-galactopyranoside) solution overnight at 37 °C. Sections were rinsed with PBS 1X, left to dry and mounted with EUKITT® medium.

### *In vitro* experiments

#### Reagents

Recombinant human Macrophage-Colony Stimulating Factor (MCSF), human interleukin-34 (IL34), human M-CSF receptor (MCSFR/CD115), human TGF-β1, human bone morphogenetic protein 2 (BMP2), human bone morphogenetic protein 4 (BMP4), human bone morphogenetic protein 7 (BMP7), human Noggin, Activin RIIA receptor (ActRIIA), human Activin RIIB receptor (ActRIIB), human TRANCE (RANKL) and antibody anti-human M-CSFR, Anti-Phosho-M-CSFR (Y723) were obtained from R&D Systems (Abingdon, UK). Anti-human IL34 (BT-34) mouse IgG1 monoclonal antibody was produced by Diaclone (Besançon, France) under patent (Heymann D, Ségaliny A, Brion R. University of Nantes /Nantes Hospital/INSERM, “Anti-IL-34 antibodies”. WO/2016/097420 A1, 2016). Antibodies directed against human Smad1 (D59D7), human Smad2 (D43B4), anti Phospho-Smad1/5 (ser463/465) (41D10), anti-phospho-Smad2 (Ser465/467) (138D), β-Actin (8H10D10) and HRP-conjugated secondary antibodies were purchased from Cell Signalling (Ozyme, Saint Quentin Yvelines, France). AlphaLISA® SureFire® Ultra Total SMAD1 and p-SMAD1 (Ser463/465) Assay kits were purchase from PerkinElmer (Villebon-sur-Yvette, France).

#### Cell cultures

The cell lines used in the present study were purchased from the American Tissue Cell Collection (ATCC, Molsheim, France). HEK293 (HEK) transfected with the pCDNA3 empty plasmid or the pCDNA3 plasmid containing the MCSFR gene as described by Segaliny *et al.*, 6. Human Mesenchymal Stem Cells-Bone Marrow (HMSC-BM) (CLS catalog number 300665, Lot.071222P2) and human MNNG/HOS osteosarcoma cell line (ATCC, catalog number CRL-1547) were cultured in Dulbecco's Modified Eagle's Medium (DMEM, Lonza, Levallois-Perret, France) supplemented with 10% fetal bovine serum (FBS; Hyclone Perbio, Bezons, France) and 2 mmol/L of L-glutamine. All the experiments using HMSC-BM cells were done at passage 2. For the Human MNNG/HOS osteosarcoma cell line experiments were performed between passage 2 and 4. All cell lines were regularly tested for the absence of mycoplasma.

#### Human osteoclast differentiation

CD14^+^ monocytes were isolated from peripheral blood of 3 healthy donors CD14^+^ cells were initially isolated from human peripheral blood donors provided by the French blood bank institute (Etablissement Français du Sang, Nantes, France, authorization number: NTS 2000-24), by using MACS microbeads (MiltenyiBiotec, Bergisch Gladbach, Germany) as previously described 68. For osteoclast differentiation, CD14^+^ cells were cultured in alpha-MEM (Lonza) supplemented with 10% human serum (Invitrogen, France) and in the presence of human MCSF (25 ng/mL) or human IL34 (100 ng/mL) +/- human BMP2 (40 or 100 ng/mL) for 3 days. Then cells were treated with same molecules in the presence of human RANKL (100 ng/mL) for 11 days. Medium was renewed every 3 days. After 11 days of treatment, osteoclasts were analyzed by Acid Phosphatase (TRAP) staining kits (Sigma Aldrich, Saint-Quentin Fallavier, France). TRAP^+^ multinucleated cells with 3 nuclei and more were considered as osteoclasts and were manually enumerated.

#### Human osteoblastic differentiation

Human Mesenchymal Stem Cells-Bone Marrow (HMSC-BM) (CLS catalog number 300665) were purchased from CLS (Germany). Osteoblast differentiation assays were performed as previously described 67,69. Briefly, HMSC-BM were cultured in DMEM was supplemented 10% of FBS, vitamin D3 (10^-8^ M; Sigma) and dexamethasone (10^-7^ M; Sigma). After 3 days, ascorbic acid (50 ng/mL; Sigma) and β-glycerophosphate (10 mM; Sigma) were added to allow mineralization detected by alizarin red-S staining for three weeks. Images were captured using a stereomicroscope (Nikon), and mineralized surfaces were quantified using Image J software. Mineralization process was carried out in the presence or absence of human cytokine IL34 (25 ng/mL), BMP2 (10 ng/mL) or combination of both molecules for 3 weeks. RNA samples were collected at days 3, 4, 14 and 21 after the induction of differentiation.

#### Flow cytometry

FACS analysis of CD11b monocytic bone marrow and spleen cells were performed as previously described 70. Briefly, after red blood cell lysis (Sigma-Aldrich), bone marrow and spleen cells were labelled with anti-CD11b (clone M1/70; BD Bioscience, Le Pont de Claix, France). Data were acquired using a FACS Canto-II (BD Biosciences).

#### Western blot

The cells were collected in a RIPA buffer (10 mM Tris pH 8, 1 mM EDTA, 150 mM NaCl, 1% NP40, 0.1% SDS containing a cocktail of protease and phosphatase inhibitors Halt™ (Thermo Fisher, Waltham, MA, USA). The protein concentration was determined using a BCA (bicinchoninic acid) method by BC Assay Protein Quantitation Kit (Interchim, Montluçon, France). 50 μg of protein extracts were prepared in a Laëmmli buffer (62.5 mM Tris-HCl, pH 6.8, 2% SDS, 10% glycerol, 5% 2-mercaptoethanol, 0.001% bromophenol blue) and then separated by SDS-polyacrylamide gel electrophoresis. After electrophoretic transfer, the immobilon-P membranes (Millipore, Molsheim, France) were blotted with the antibodies referenced in the “Reagents” section. The membranes were then probed with secondary antibodies coupled with horseradish peroxidase. Antibody binding was visualized with an enhanced chemiluminescence (ECL) kit Clarity™ Western ECL Substrate (Bio-Rad, Marnes-la-Coquette, France). The luminescence was detected with a ChemiDoc MP Imaging System (Bio-Rad). Blots images and semi-quantitative analysis were done using ImageJ software (USA). Each experiment was repeated at least 3 times.

#### SMAD1/5 signaling measured by Alpha SureFire® Technology

Direct quantification analysis of cell signaling was performed by using Alpha SureFire® Technology from PerkinElmer in a Victor® Nivo™ multimode microplate reader (ALSU-PSM1; PerkinElmer, Villebon-sur-Yvette, France).

#### RNA isolation and real-time PCR

Total RNA was extracted using NucleoSpin® RNA Plus (Macherey-Nagel, Duren, Germany). 1 μg of total RNA was used for first strand cDNA synthesis using the OneScript® RT Mix (Ozyme). Real-time PCR was performed on 20 ng of reverse transcribed total RNA (cDNA), 300 nM of primers (QuantiTect Primer® Assays, Qiagen) and PowerUp™ SYBR™ Master Mix from Applied Biosystems™ (Thermo Fisher) in a CFX96 Touch Deep Well Real-Time PCR Detection system from Bio-Rad. Thermal cycle conditions were perform by following manufacture protocol. The analysis was performed with CFX Manager Software (Bio-Rad) using human glyceraldehyde 3-phosphate dehydrogenase (GAPDH), Hypoxanthine Phosphoribosyl transferase 1 (HPRT1) and TATA box binding protein (TBP) as invariant controls (QuantiTect Primer® Assays, Qiagen). Oligonucleotides were designed with Primer-Blast software (NCBI) and purchased from Eurogentec (Eurogentec, Angers, France). The 2^-ΔΔCt^ (cycle threshold) method was used to calculate expression levels. List of primers and gene name symbols with corresponding full names are indicated in Tables S1 and S2.

#### Surface plasmon resonance (SPR) assays

All SPR experiments were performed on a T200 apparatus (Cytiva) at 25 °C in PBS pH 7.4 containing 0.05% of surfactant P20. Human recombinant BMP2, BMP4 and BMP7 proteins were immobilized (1500- 2300 RU) at pH 4.5 on CM5-S sensor chip by amine coupling following the manufacturer's instructions (Cytiva, Velizy-Villacoublay, France). IL34 kinetics were measured using one cycle titration, for these five increasing concentrations of recombinant human IL34 (12.5, 25, 50, 100, 200 nM) were injected during 60 s at 100 µL/min on coated BMPs. The last injection was followed by a 600 s dissociation time in running buffer. The KD values were evaluated using a bivalent fitting model (T200 Evaluation software 3.2.1, Cytiva). All sensorgrams were corrected by subtracting the low signal from the control reference surface (without any immobilized protein) and blank buffer injections before fitting. For KD evaluation of IL34 on human recombinant receptors BMPRIIA, Act RIIA and Act RIIB, these receptors were captured on immobilized anti-human Fc (Cytiva), four increasing concentrations of IL34 (18.75, 37.5, 75, 150, 300 nM) were injected. The KD values were evaluated by using a steady-state fitting model. The binding responses of IL34 (50 nM) alone, Nogging (50 nM) alone and a mixing of IL34 and Noggin were measured by 180 s injection on different coated BMP proteins (BMP2, BMP4, BMP7) at a flow rate of 30 µL/min followed by a dissociation time of 400 s in running buffer.

#### Protein-protein docking and analysis

Structures of M-CSFR, BMPR1 and IL-34 were extracted from their bound crystallographic forms (1REW for BMPR1A + BMP2 71, 4WRL for M-CSF:M-CSFR1 72 and 4DKD for IL-34:M-CSFR1 73). Docking experiments were performed using either BMP-2 fixed and the partner protein mobile, or the reverse, as previously published 71. ClusPro analysis 74 was performed in balanced mode, only the first 10 binding modes clusters were considered for analysis, the best modes were selected by visual inspection. Interface analysis was performed using the PISA web server 75. Visualization and superimposition of docking poses and crystallographic structures were done using PyMOL (The PyMOL Molecular Graphics System, Version 2.5 Schrödinger, LLC; Schrödinger, LLC 2015).

### Statistical analysis

All experiments were repeated at least three times in independent experiments. The differences between the experimental conditions were assessed with Student's t test or a one-way ANOVA followed by the Mann-Whitney test or Kruskal-Wallis test (in the case of more than two independent samples of equal or different sample size). The results are given as a mean ± SD. Results were considered significant at p-values of ≤ 0.05, p-values of ≤ 0.01 and p-values of ≤ 0.001. GraphPad Prism 6 software (GraphPad Software, San Diego, CA, USA) and Real Statistics Resource Pack Software (Release 8.91), copyright (2013-2023) Charles Zaiontz (www.real-statistics.com) were used for statistical analyses.

## Supplementary Material

Supplementary figures.

Supplementary movie.

Supplementary tables.
