# Supplementary material for: Interleukin-34 orchestrates bone formation through its binding to bone morphogenic proteins
Source: Theranostics. 2025 Feb 11;15(7):3185–202. doi: 10.7150/thno.107340 (PMC11898274; doi:10.7150/thno.107340)
Supplement: Supplementary file 1 — Supplementary figures. [file thnov15p3185s1.zip › Munoz-Garcia et al _Supplemenary Figures revised.docx]

**Supplementary figure 1**


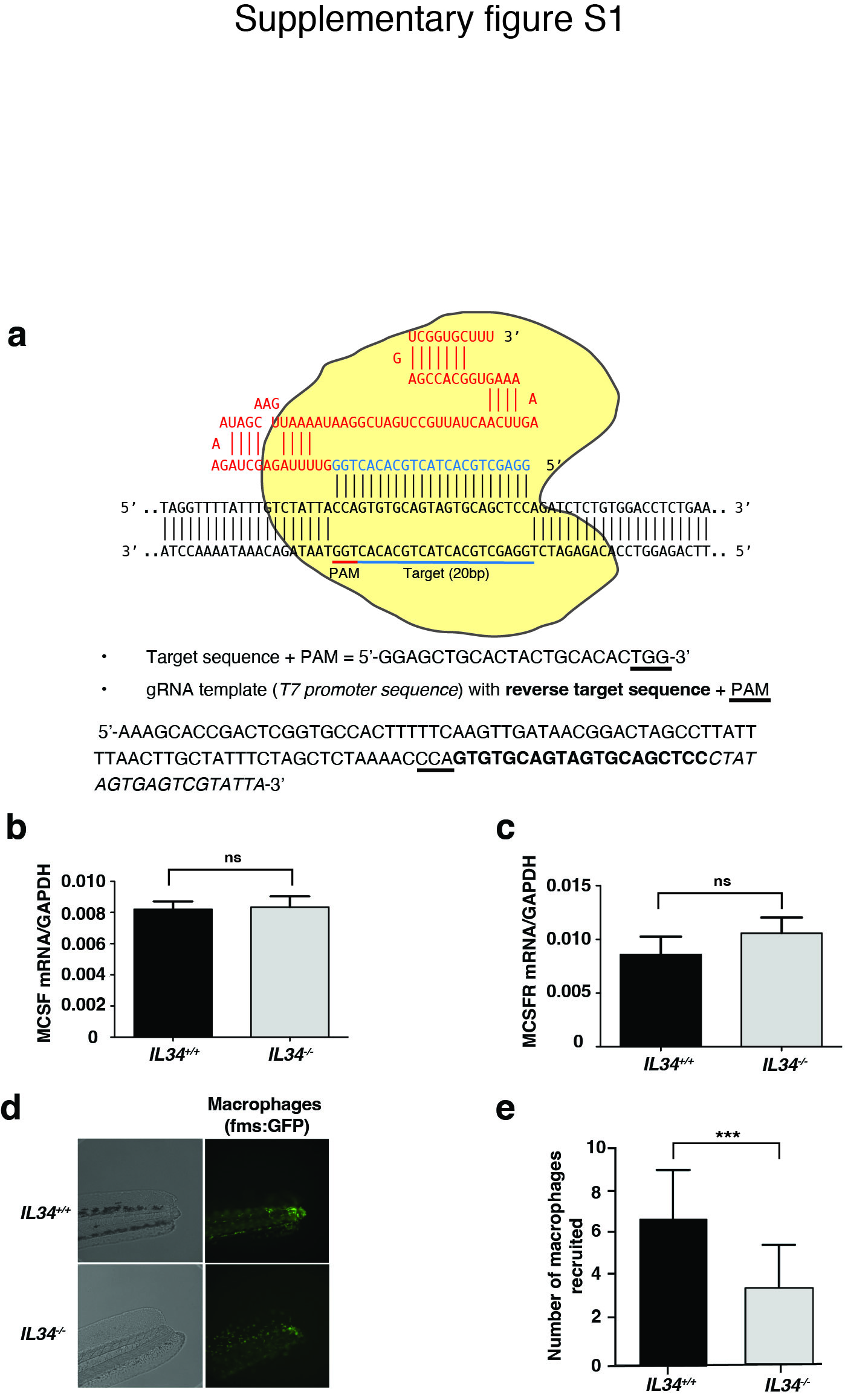


**Figure S1. Tools to *IL34* genetic invalidation in zebrafish using CrispR/Cas9 technology and functional confirmation.** (**A**) Schematic representation of the targeted sequence in *il34* exon 3 including the protospacer adjacent motif (PAM) and the use guide RNA template sequence containing a T7 promoter sequence and the reverse targeted sequence and PAM. (**B-E)** Functional confirmation of *il34* invalidation was achieved showing the loss of the known IL34 effects on the differentiation and survival of monocytes and macrophages, [6,74] using the caudal fin amputations strategy on 3 day embryos from a heterozygous (+/-) in-cross of the double transgenic line *tg*(fms: GFP). This line contained the heterozygous mutant *il34* allele, and GFP expression of macrophages was driven by the *MCSFR* promoter. To visualize these inflammatory cells throughout injury, live images of the amputated caudal fins were taken at 8 hours post fin injuries. The 8 hours-time-point was chosen as this is the optimal time for recruitment of macrophages to the site of injury. At this time, despite no variation of *MCSF* and *MCSFR* transcript expression levels induced by *IL34* invalidation **B-C**, an important reduction of the number of macrophages recruited **D-E** was observed in the *il34-/-* comparatively to the control (*il34+/+*).

**Supplementary figure 2**


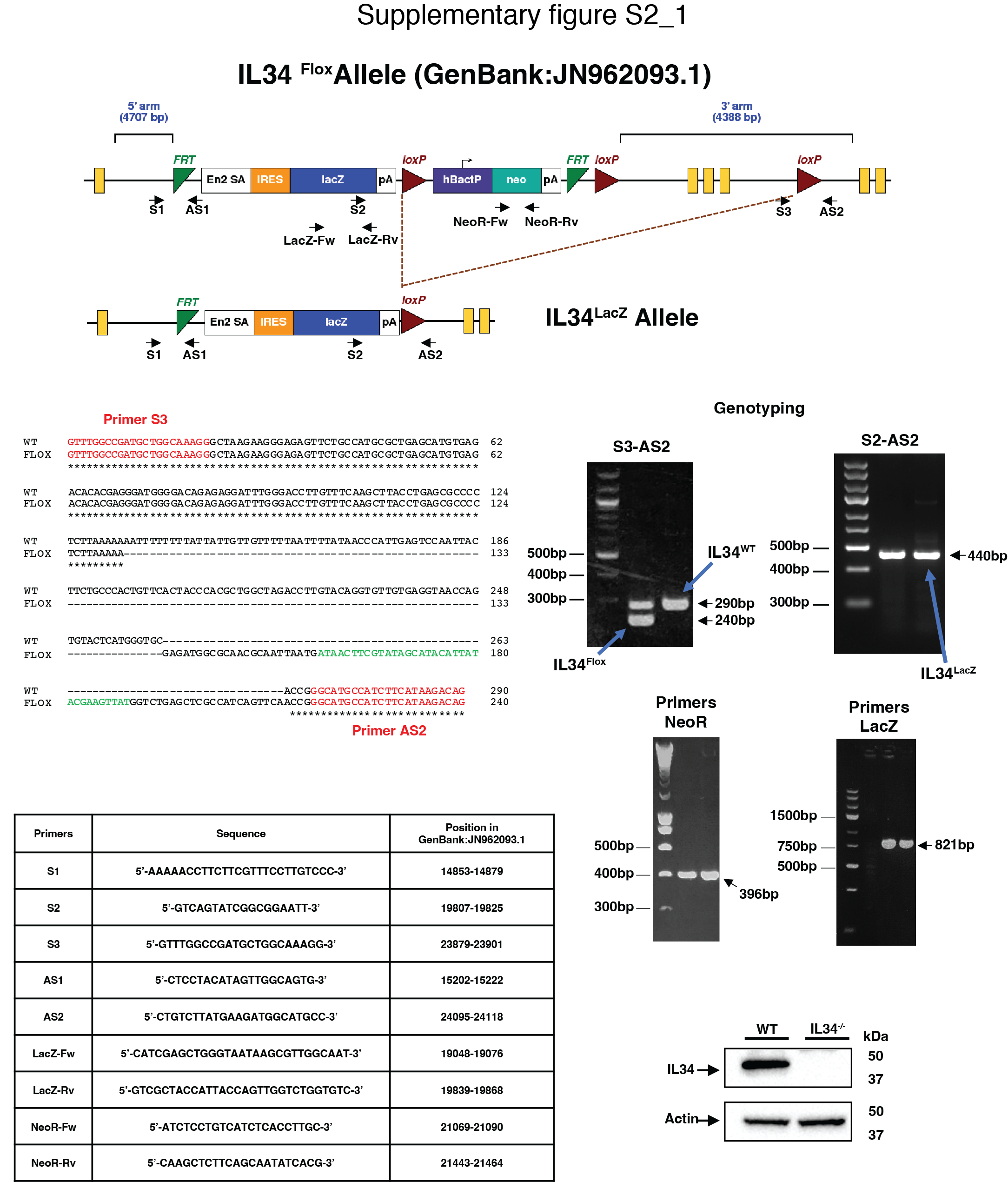


**
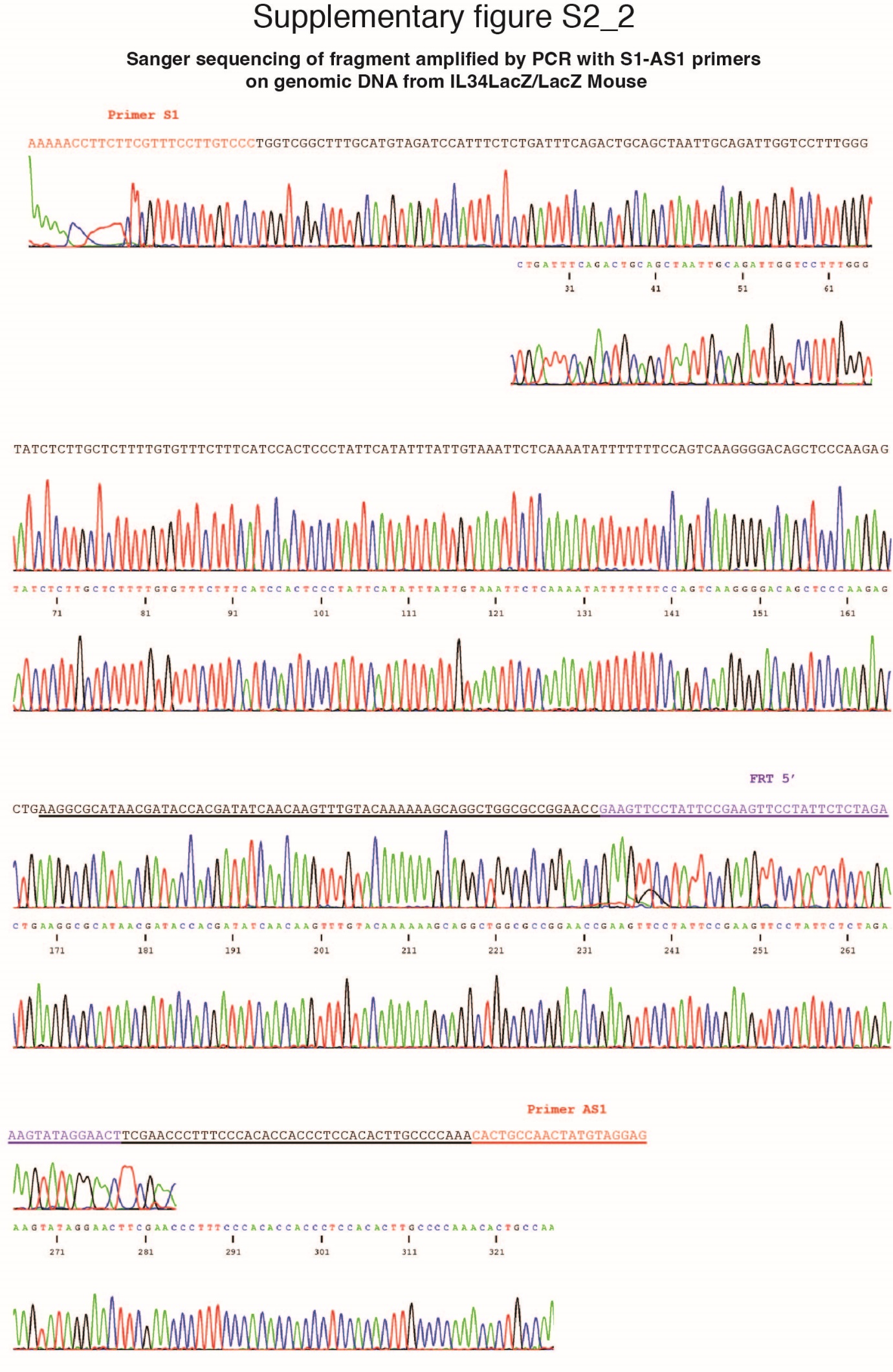
**

**
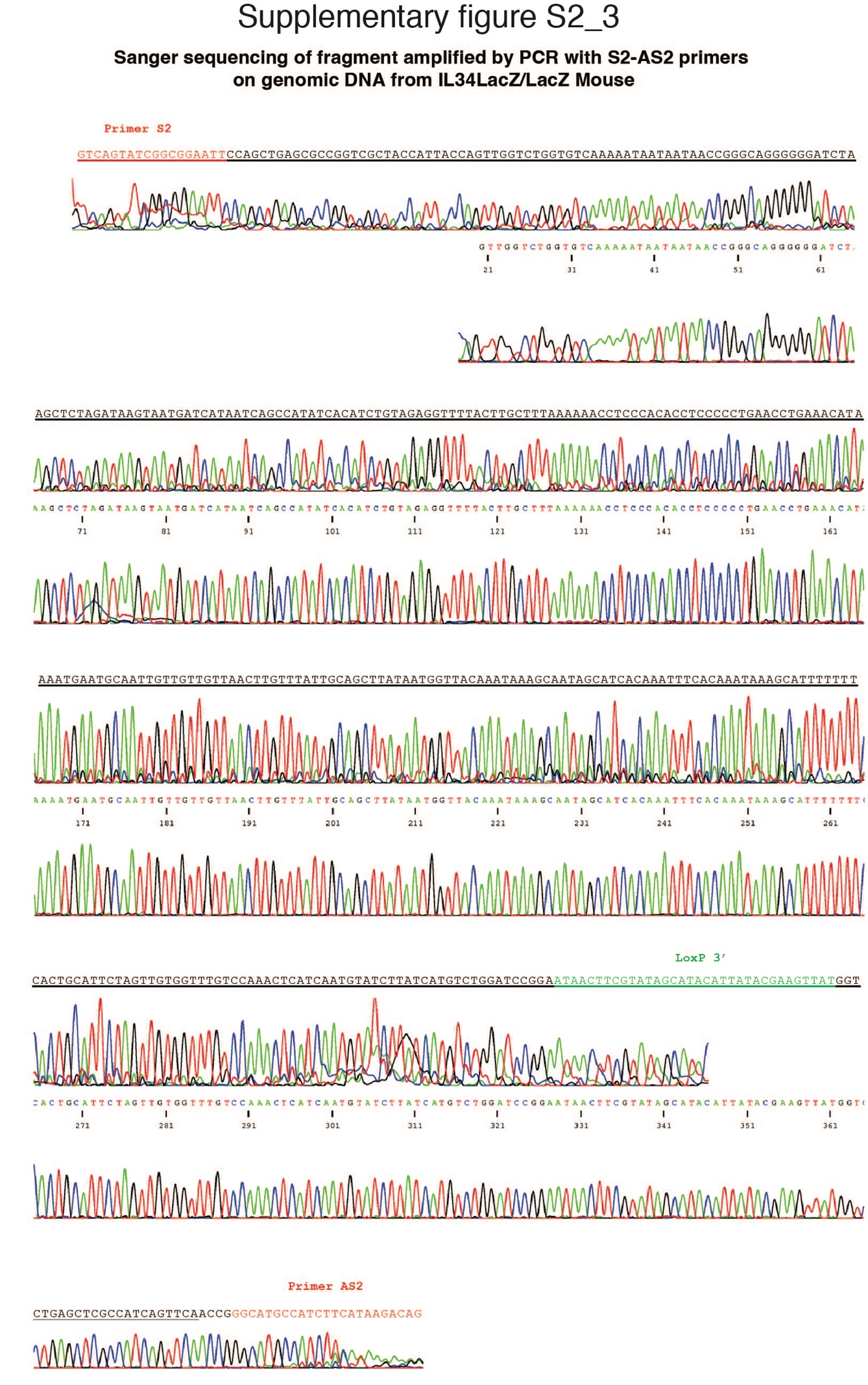
**

**Figure S2. *IL34* genetic invalidation in mouse.** *Il34Flox* allele (Genbank: JN962093.1) and the derived *Il34LacZ* allele obtained after recombination between its 5’ and 3’ LoxP sites are graphically represented. Primers used to genotype the different alleles are given in the table and positioned on the different graphical representations. Primers S3 and AS2 enable to discriminate the *Il34Flox* allele from the WT allele with respectively amplification of 240 bp and 290 bp fragments. Primers S2 and AS2 enable to identify the *Il34LacZ* allele corresponding to the amplification of a 440 bp fragment. LacZ and NeoR primer pairs were used to check the integrity of the *Il34Flox* allele. Fragments amplified with S1-AS1 and S2-AS2 primer pairs from genomic DNA extracted from *Il34LacZ/LacZ* mouse tail were Sanger sequenced to confirm recombination terminals (sequences corresponding to the construct cassette are underlined). The disruption of IL34 has been also analyzed by western blot. Briefly, whole protein extracts were obtained from frozen spleens from WT and IL34 null mice and specific antibody targeting IL34 was used. As showed, IL34 was only detected in WT and not in IL34 null sample.

**Supplementary figure 3**

**
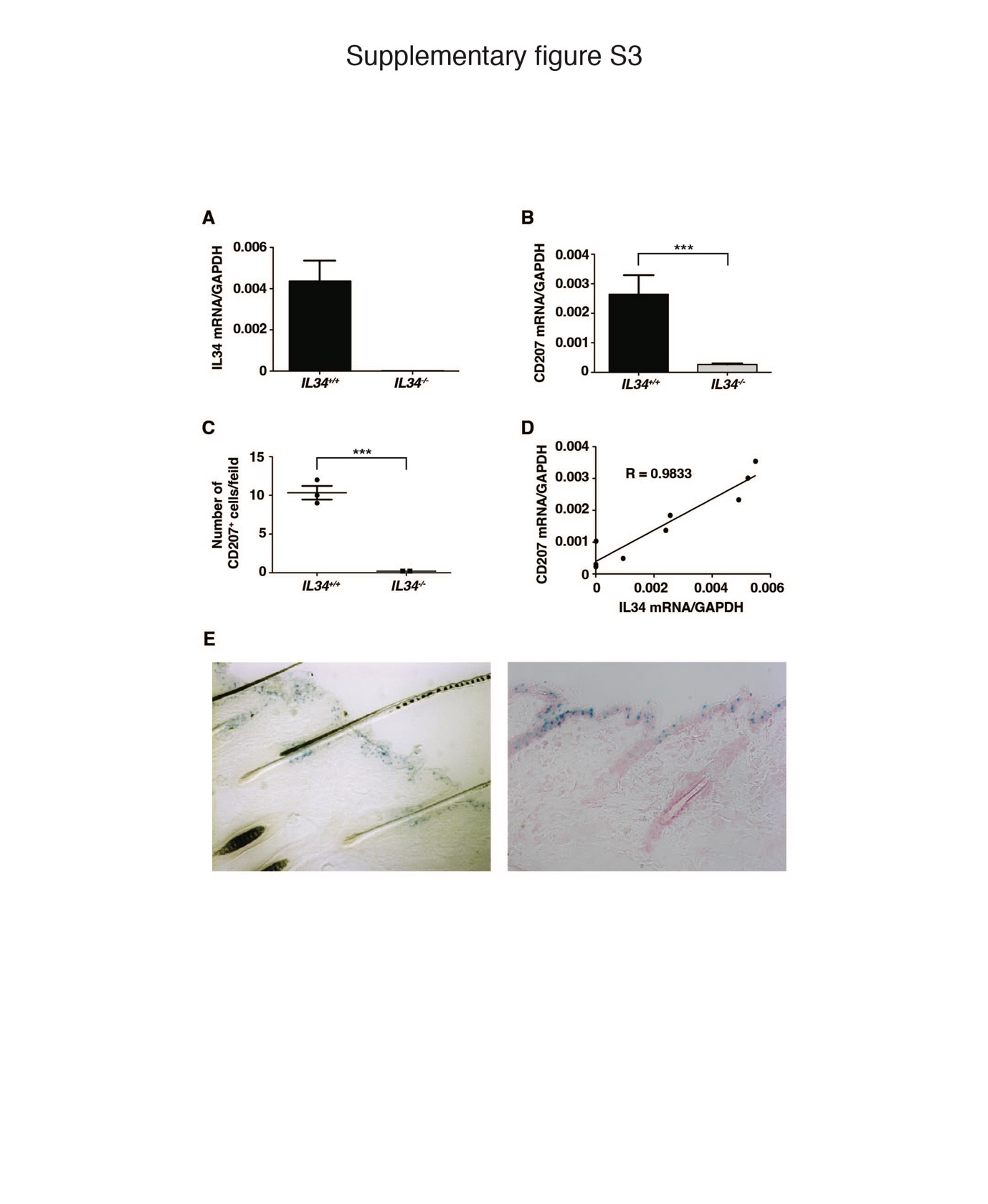
**

**Figure S3. Functional confirmation of *IL34* invalidation and *IL34 LacZ* reporter in mouse.** (**A-D**) In order to confirm the effective loss of IL34 function in *Il34-/-* mouse, skin well-known as an IL34 expression site [16] was used. Correlated to IL34invalidation, a significant reduction of CD207 expressing cell (Langerhans cells) was observed. (**E**) ß-galactosidase staining (blue) performed on section of *Il34+/LacZ* mouse enable to confirm previously described expression of IL34 [15,16,75] in some keratinocytes and cells from the hair follicle.

**Supplementary figure 4**

**
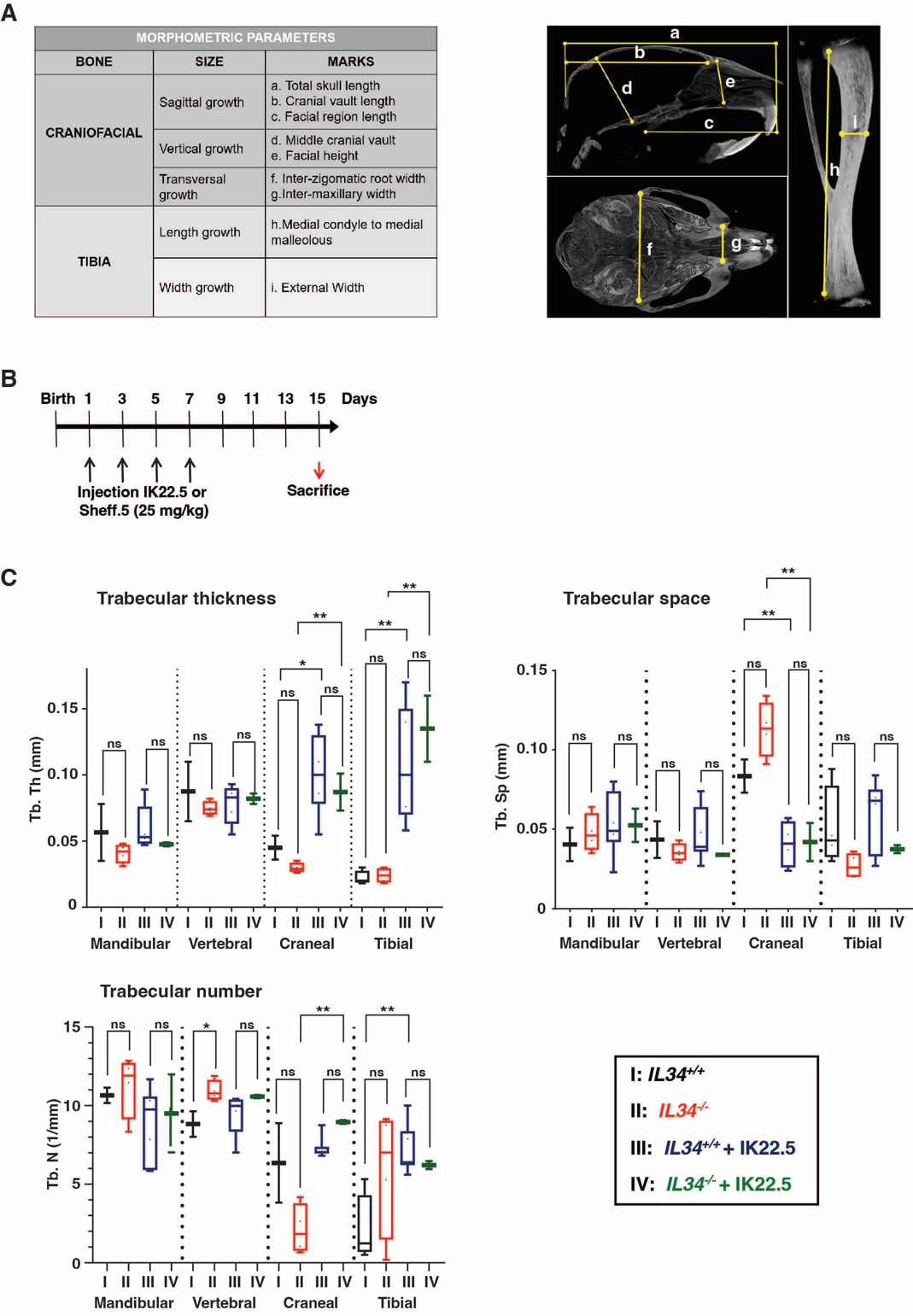
**

**Figure S4. Graphical representation of morphometric parameters used for mouse head and tibia analyses, blocking antibodies injections protocol and results of bone trabecular structure analyses at different anatomical sites.** (**A**) The seven morphometric parameters (a-g) measured for the head (craniofacial skeleton) and the two parameters (h-i) measured for tibia (appendicular skeleton) are listed and represented on 2D microCT scan views. (**B**) The blocking antibodies (IK22.5 and Sheff5) injections protocol was characterized by four subcutaneous injections at postnatal days 1, 3, 5 and 7 of 25 mg/kg of antibody following by a sacrifice at day 15. (**C**) The trabecular thickness (Tb. Th), the trabecular space (Tb. Sp) and the trabecular number (Tb. N) were determined at four anatomical sites, the mandible, the vertebra (C2) the skull and the tibia. *Il34*+/+ are represented in black, *Il34*-/- in red, *Il34*+/+ treated with IK22.5 in blue, and *Il34*-/- treated with IK22.5 in green. *p<0.05, **p<0.01, ***p<0.001, ****p<0.0001, ns: not significant. n=8 except for *Il34-/-* + IK22.5 (n=4).

**Supplementary figure 5**

**
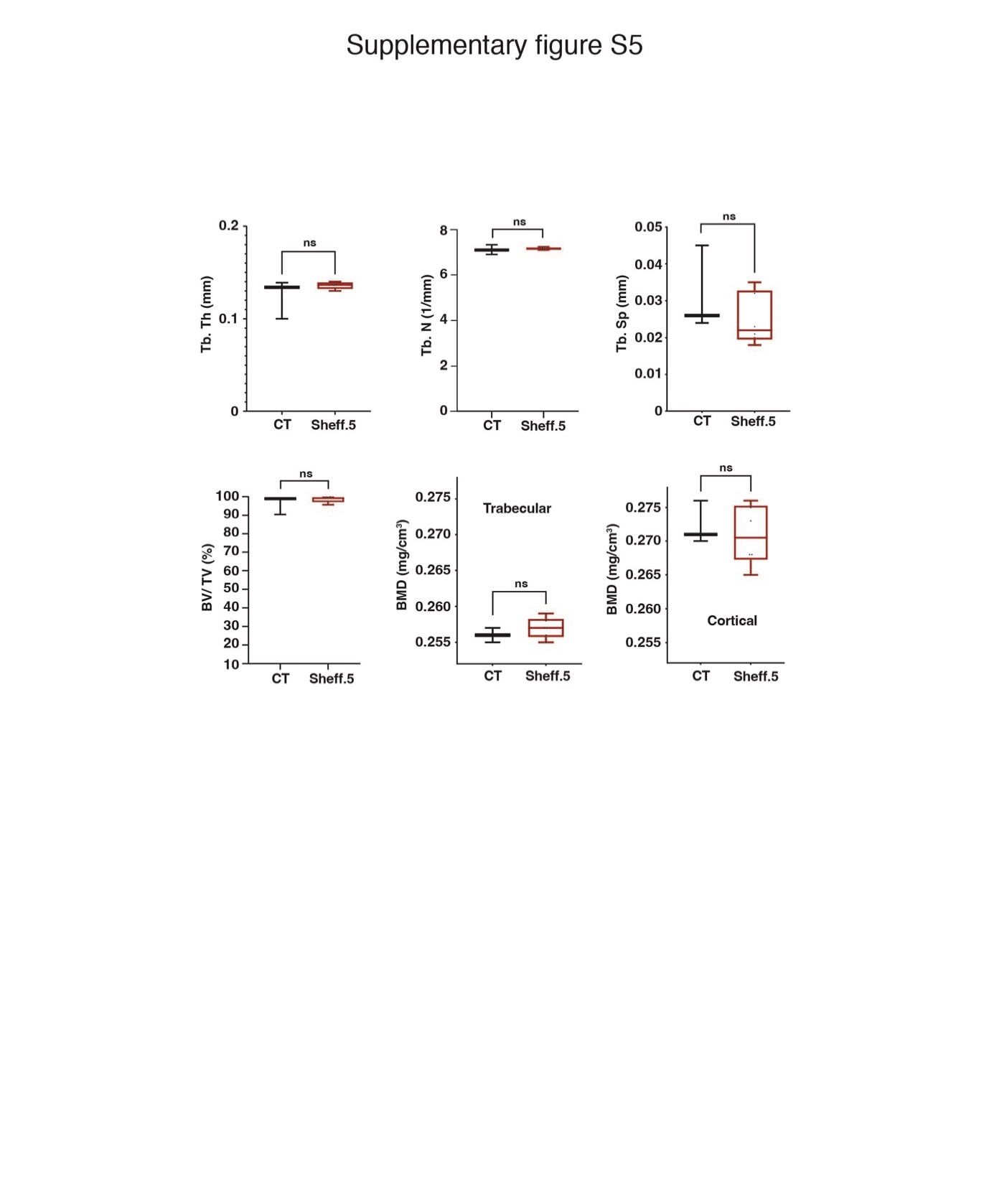
**

**Figure S5. Analyses of the impact of Sheff.5 antibody injections in WT pups on the bone trabecular structure (Tb. Th, Tb. Sp and Tb. N), the percentage of BV/TV and BMD.** No significant variation was observed comparatively to pups injected with a control irrelevant antibody from the same idiotype whatever the parameter considered. ns: not significant. CT: control group. n=8.

**Supplementary figure 6**


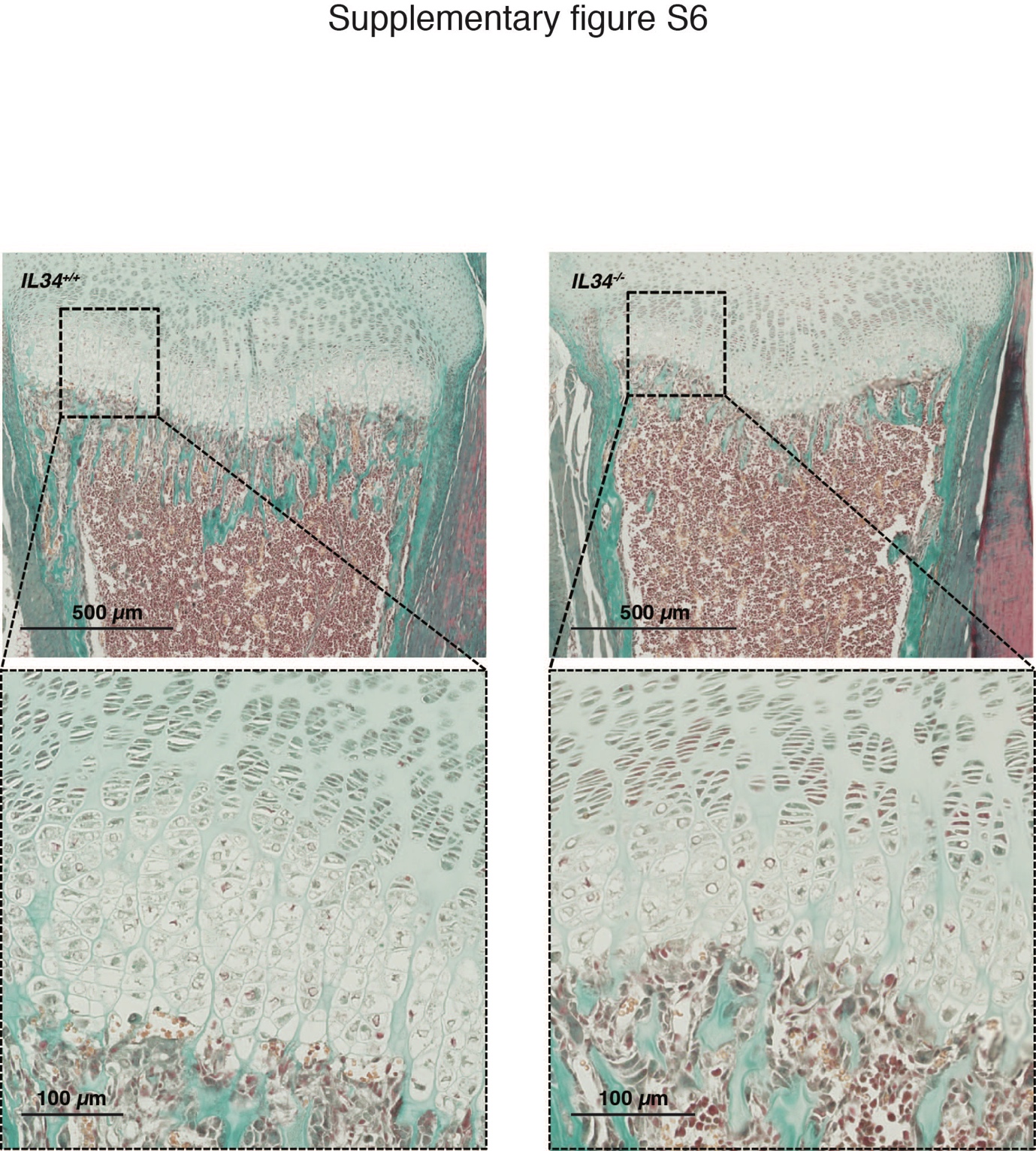


**Figure S6. Histological comparative analysis of the tibia proximal epiphysis area of 15-day-old *Il34-/-* and *Il34+/+* mice using Masson’s trichome stained longitudinal sections.** An important reduction of the thickness of the hypertrophic chondrocytes area was observed in the null mutant. The scales are given as bars with the corresponding values in the lower part of each histological view.

**Supplementary figure 7**


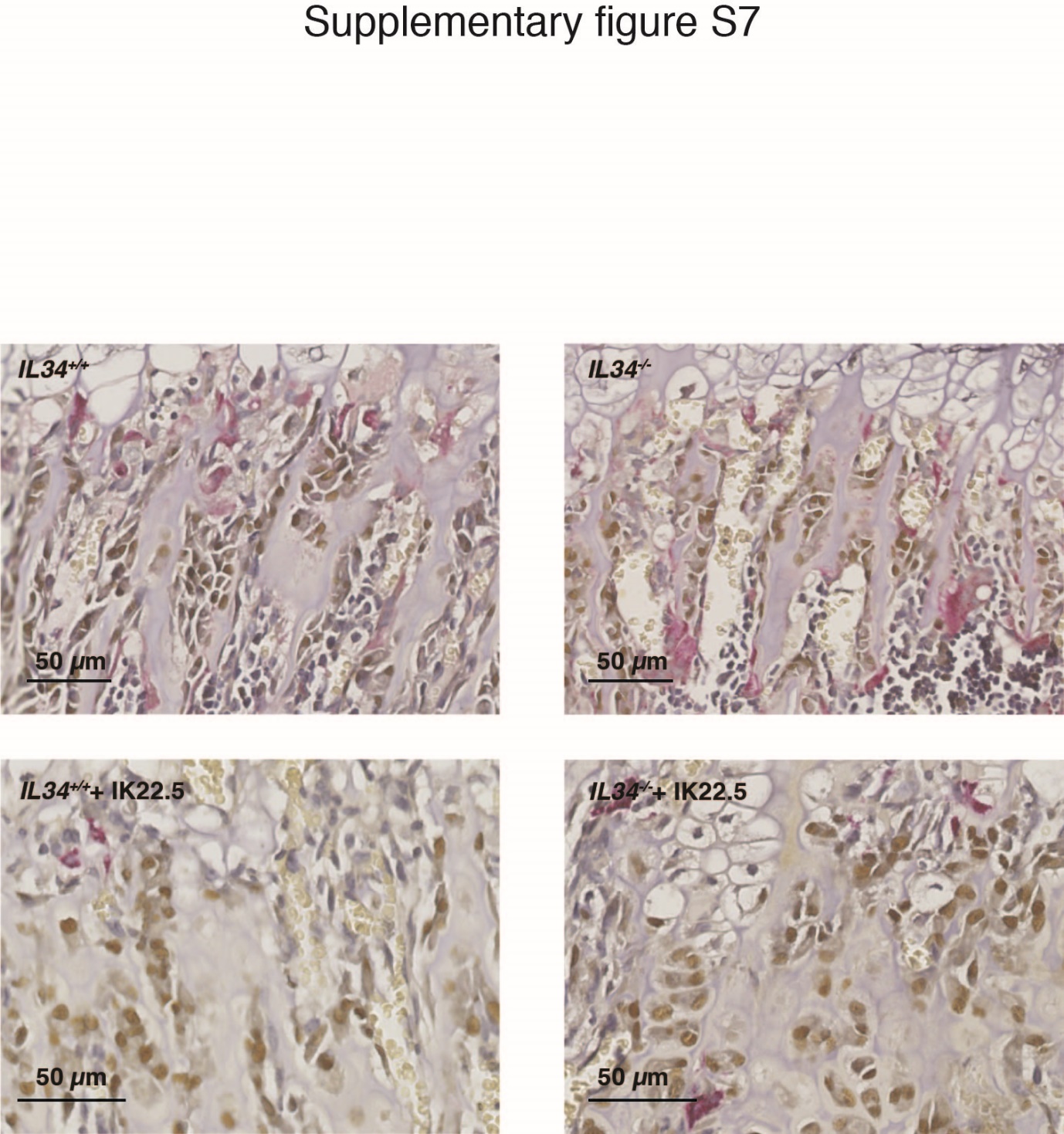


**Figure S7. High magnification views of tartrate resistant acid phosphatase (TRAP) and Osterix dual-staining of tibia longitudinal sections at the level of the proximal epiphysis performed for 15-day-old *Il34-/-* and *Il34+/+* mice treated or not with the IK22.5 antibody.** An important increase of both staining (TRAP in red and OSX in brown) was observed in the null mutant mouse. The IK22.5 injections drastically reduced the number of TRAP-positive cells in both *Il34-/-* and *Il34+/+* mice, whereas no evident variation of the number of cells stained for OSX was evidenced. The scale is given as a bar corresponding to 50 μm in the lower part of each histological view.

**Supplementary figure 8**


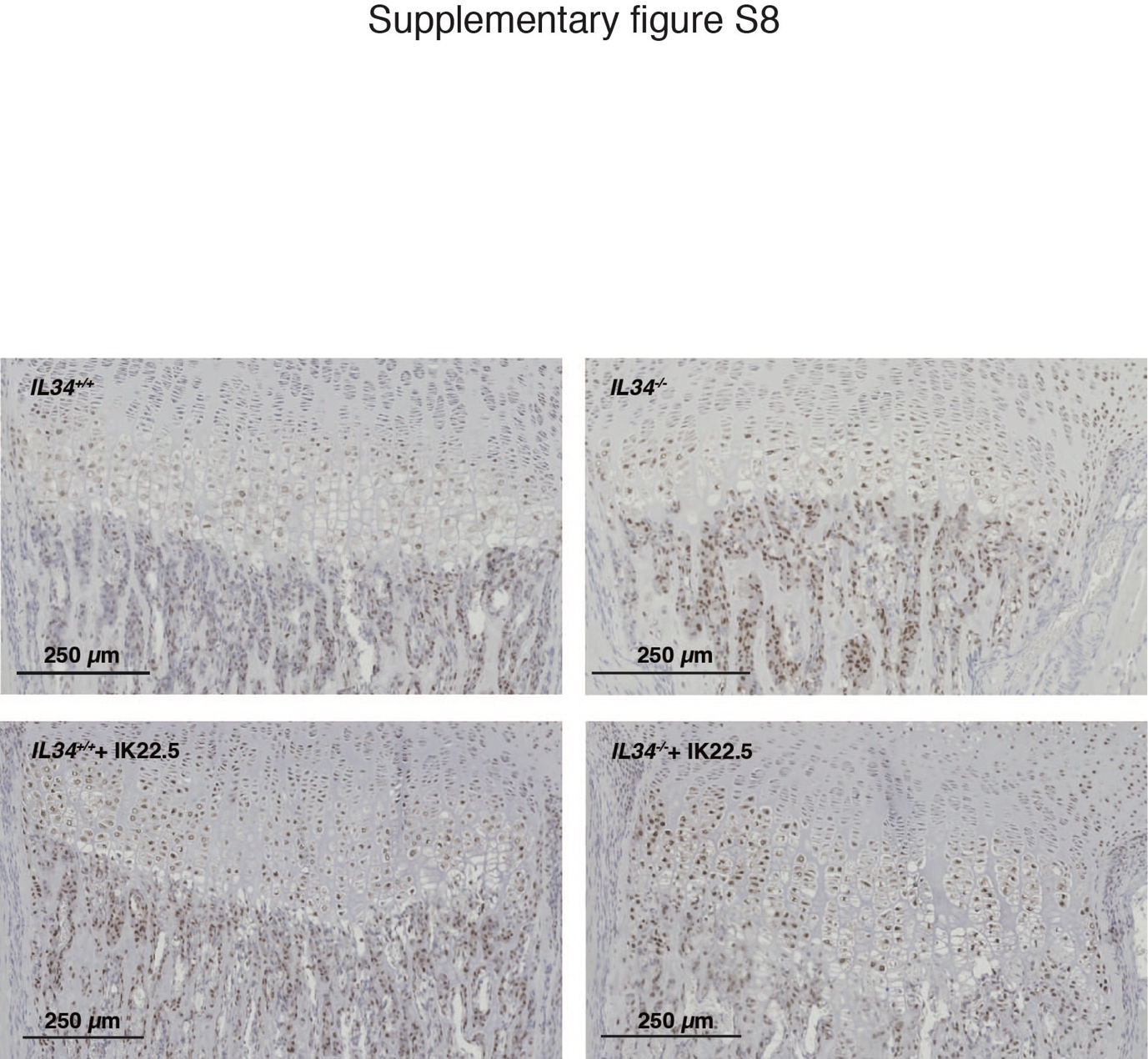


**Figure S8. Comparative analysis of RUNX2 expression in the tibia proximal epiphysis area of 15-day-old *Il34-/-* and *Il34+/+* mice treated or not with the IK22.5 antibody using immunohistochemistry applied to longitudinal sections.** No variation of the number of stained cells was evidenced between *Il34-/-* and *Il34+/+* mice as between *Il34-/-* and *Il34+/+* injected with IK22.5 antibody. The scale is given as a bar corresponding to 250 μm in the lower part of each histological view.

**Supplementary figure 9**


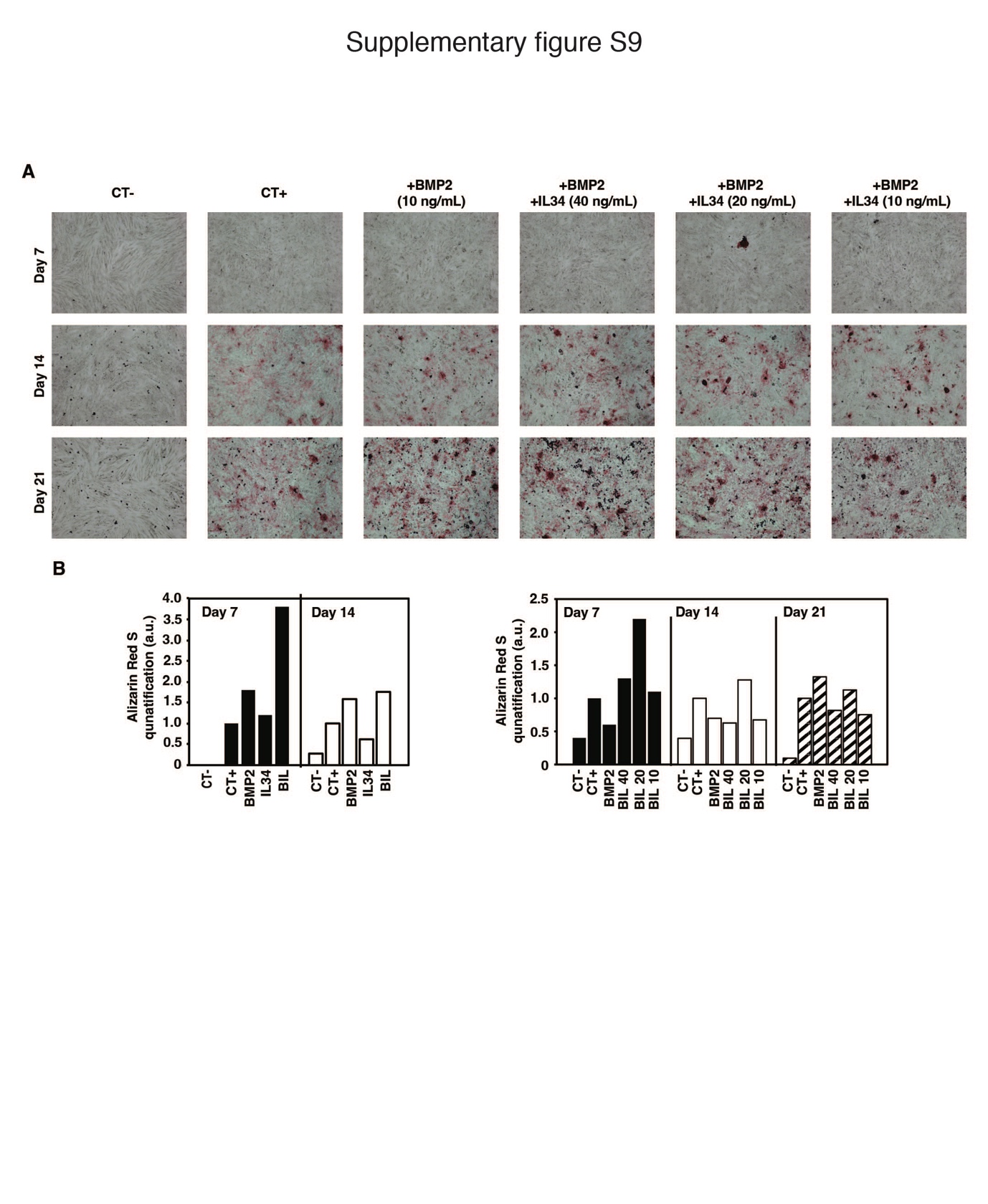


**Figure S9. Functional impact of BMP2, IL34 or combined BMP2+IL34 addition in the culture medium onto osteoblastic differentiations.** (**A**) BMP2 addition (10 ng/mL) to the osteogenic differentiation medium (CT+) induced an acceleration of the differentiation as evidenced by the higher alizarin red staining corresponding to phosphocalcic crystal deposition at 7, 14 and 21 days. IL34 addition (20 ng/mL) to the osteogenic differentiation medium had no effect on the differentiation (view not shown but the quantification is presented in **B**, right panel). Co-addition of IL34 (10, 20 or 40 ng/mL) with BMP2 (10 ng/mL) potentialized the acceleration of the osteoblastic differentiation observed with BMP2 alone with an optimal concentration ratio (ng/mL) of 2 between BMP2 and IL34. Magnification is similar for all views and the bar in CT- view at day 7 correspond to 500 µm. (**B**) Quantification of the alizarin red staining corresponding to independent experiments from different donors. BIL: BMP2+IL34 addition; Numbers (10, 20 or 40) correspond to the used concentrations of IL34 in ng/mL.

**Supplementary figure 10**

**
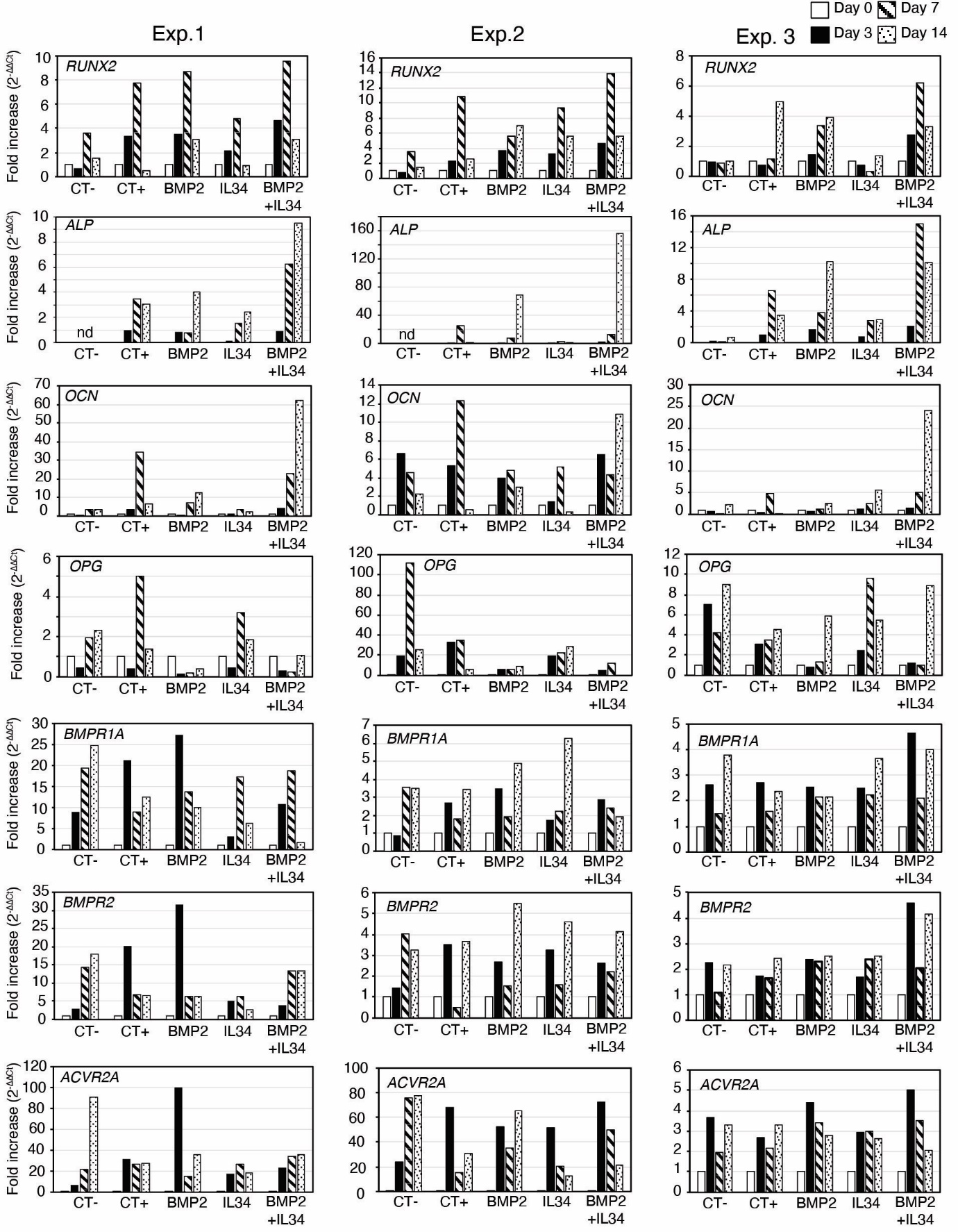
**

**Figure S10. Impacts of the additions of BMP2 (10 ng/mL), IL34 (20 ng/mL) or BMP2+IL34 to the CT+ medium onto the expression of early (*RUNX2*) and late (*ALP* and *OCN*) markers of osteoblast differentiation, onto osteoprotegerin (*OPG*, a major regulatory factor of osteoclastogenesis), and onto type 1 and type 2 receptors of the BMPs (*BMPR1A, BMPR2* and *ACVR2A*).** Results of three different experiments (Exp. 1 to 3) carried out with three different batches of mesenchymal stem cells (obtained from different donors) are presented in order to deal with the inter-batches variabilities considering only results similarly observed in the three experiments. BMP2 addition accelerated the osteoblastic differentiation and the co-addition of IL34 potentialized this effect as evidenced for *RUNX2* expression at days 3 and 7, and for *ALP* and *OCN* at day 14. BMP2 addition alone or in combination with IL34 reduced the *OPG* expression at days 3 and 7. Regarding the different receptors of the BMPs, no reproducible effects were observed between experiments, but all three receptors are expressed at all stages (days) of differentiation whatever the culture conditions used. nd: not detected.

**Supplementary figure 11**

**
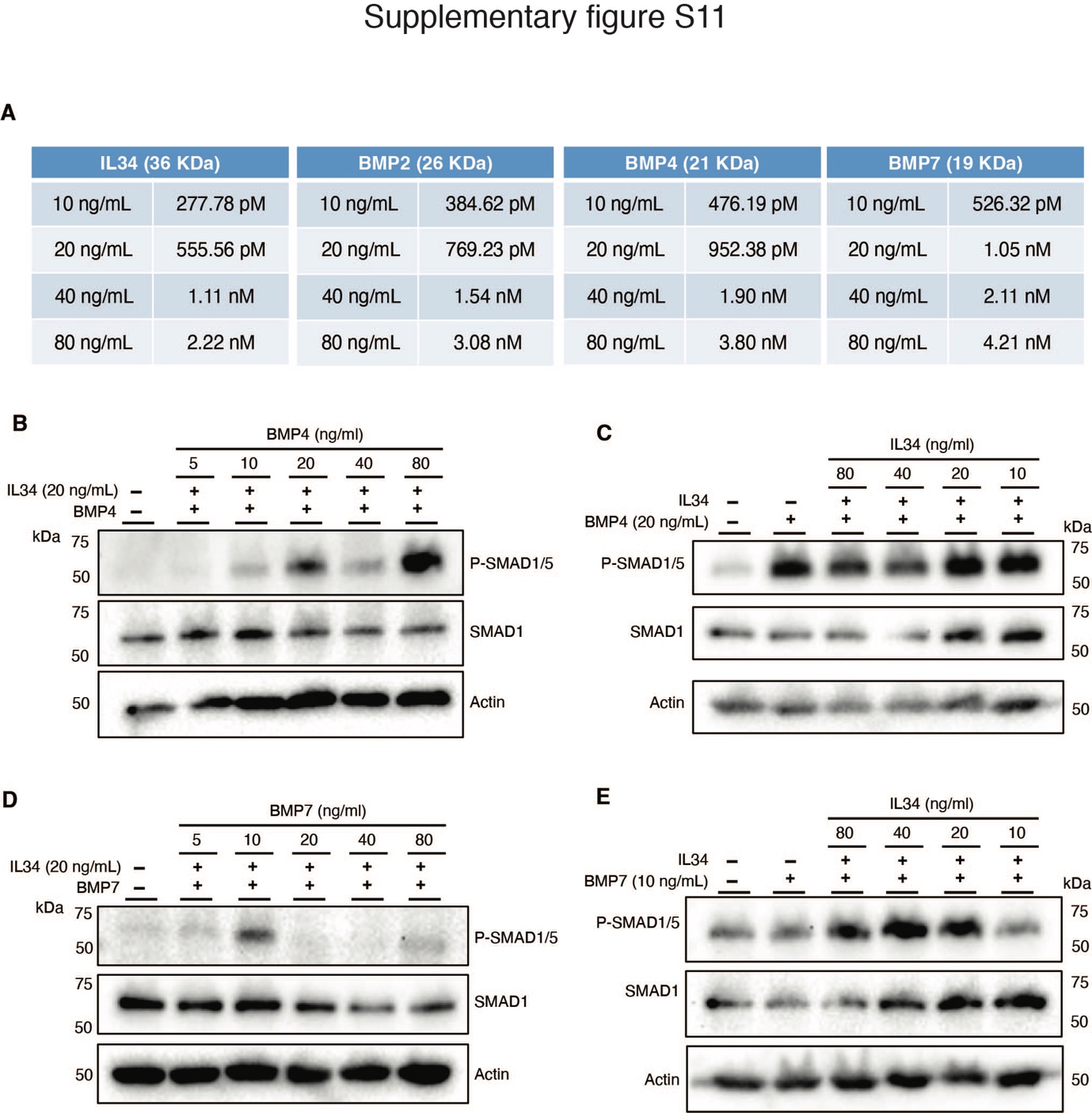
**

**Figure S11. Impacts of addition of different combinations of BMP4+IL34 or BMP7+IL34 onto the activation of BMP receptors evidenced by SMAD1-5** **phosphorylation in human MNNG-HOS osteosarcoma cell line.** (**A**) Correspondence table between csoncentrations used in ng/mL and in pM or nM for IL34, BMP2, BMP4 and BMP7. (**B**) Joint addition of BMP4 (graded concentrations from 5 to 80 ng/mL) and IL34 (20 ng/mL) induced SMAD1-5 phosphorylation, with greater effects observed with 20 and 80 ng/mL BMP4. (**C**) Joint addition of BMP4 (20 ng/mL) and IL34 (graded concentration from 10 to 80 ng/mL) induced SMAD1-5 phosphorylation, with an optimal effect observed with 20 ng/mL IL34. (**D**) Joint addition of BMP7 (graded concentrations from 5 to 80 ng/mL) and IL34 (20 ng/mL) induced SMAD1-5 phosphorylation, with greater effects observed with 10 and 80 ng/mL BMP7. (**E**) Joint addition of BMP7 (10 ng/mL) and IL34 (graded concentration from 10 to 80 ng/mL) induces SMAD1-5 phosphorylation, with greater effects observed with 20 and 40 ng/mL IL34.

**Supplementary figure 12**


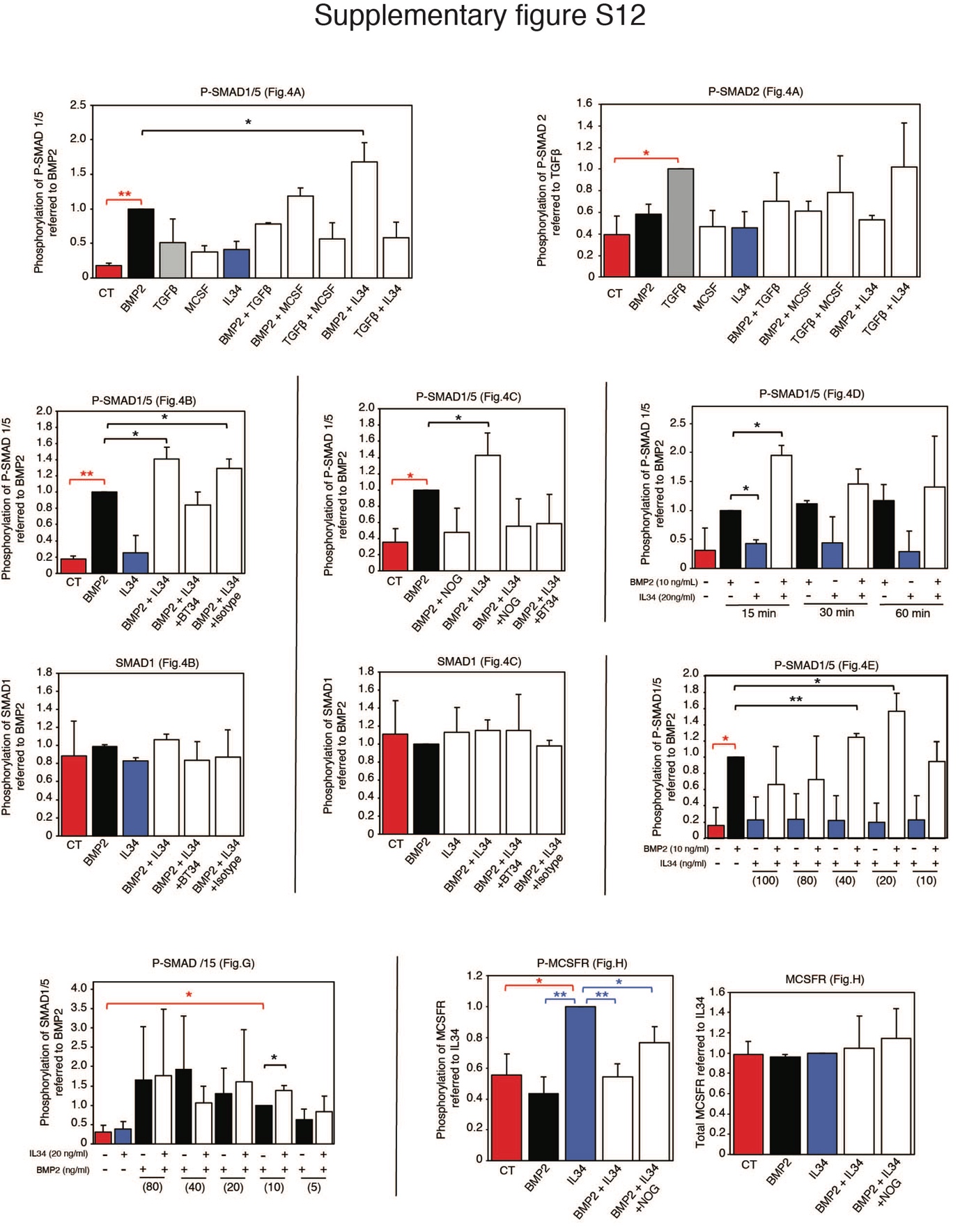


**Figure S12. Quantitative analyses of Western blots presented in Figure 4.** Histograms corresponding to the control condition, the BMP2-only condition and the IL34-only condition appear in red, black and blue respectively. *p<0.05, **p<0.01.

**Supplementary figure 13**


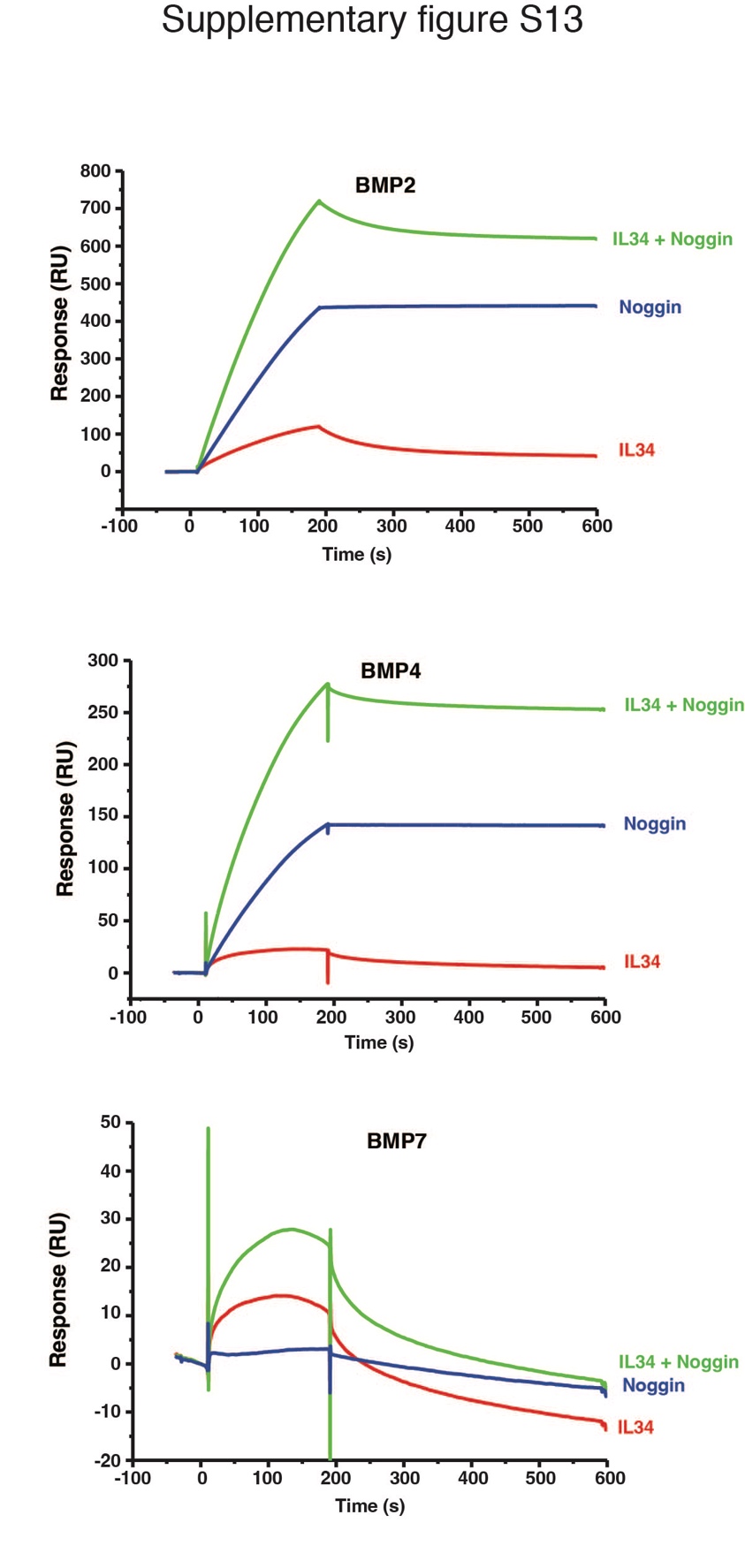


**Figure S13. Curves obtained during the surface plasmon resonance experiments with immobilization of the BMP proteins (2, 4 and 7) and additions of IL34 and NOGGIN alone or in combination.** Whatever the BMP protein considered an effective binding of IL34 as NOGGIN was observed with an additive effect of the two factors.

**Supplementary figure 14**


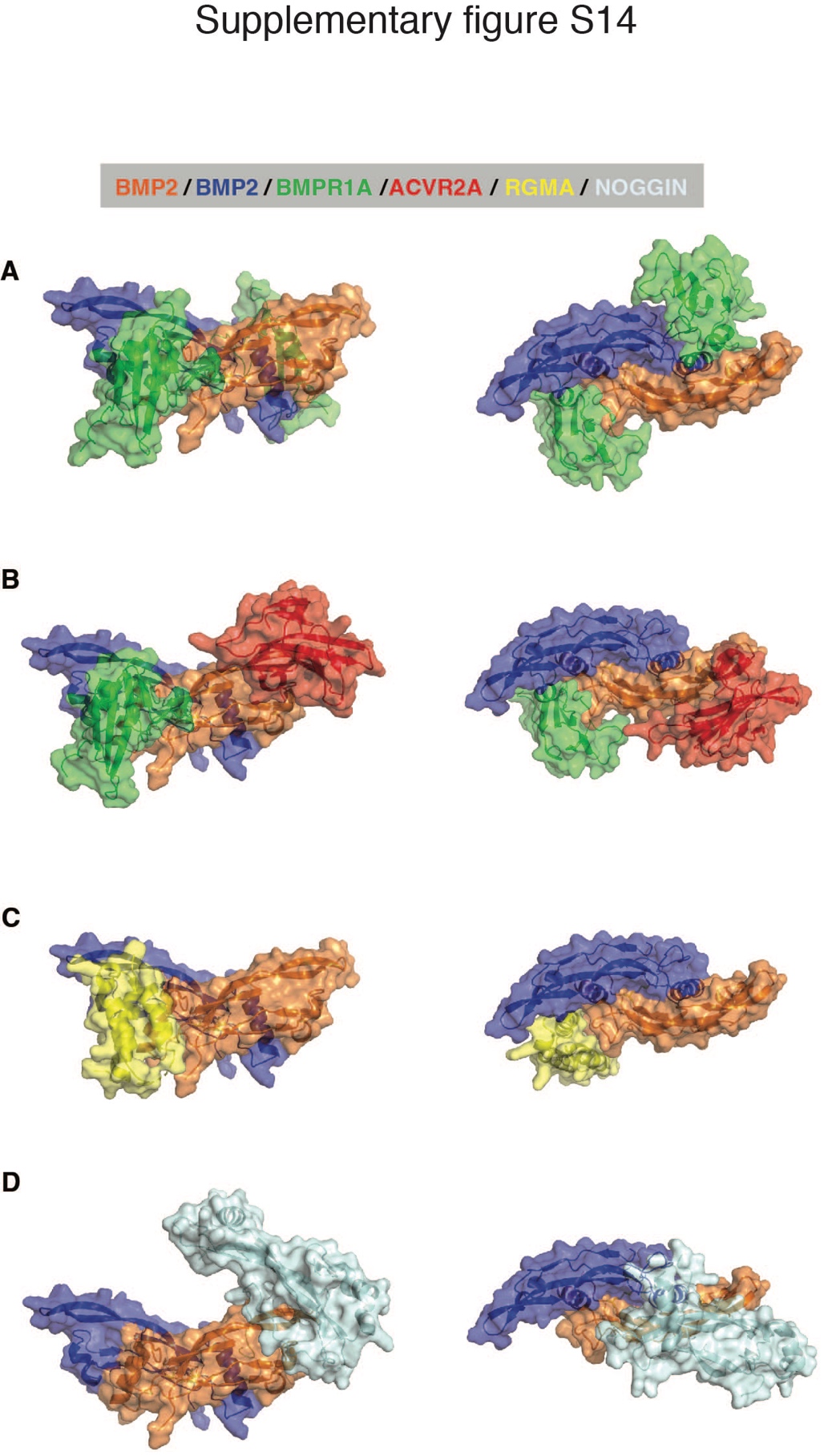


**Figure S14.** **Representative structures of BMP2 dimer bound proteins resolved experimentally.** BMP-2 dimers are displayed in blue and orange surface. (**A**) BMPR1A structure (green) as found in PDB ID 1ES7 [76]. State that binding the knuckle site. (**B**) BMPR1A (green) and Activin Receptor IIA (red) occupy each wrist and knuckle epitopes (PDB ID 2GOO [77]). (**C**) RGMA (yellow) as found in PDB ID 4UHY [78]. State that binding the knuckle site. (**D**) NOGGIN was determined to occupy both epitopes in structure PDB ID 7AG0 [79]. Left: side view of protein complexes, right: top view of protein complexes.

**Supplementary figure 15**

**
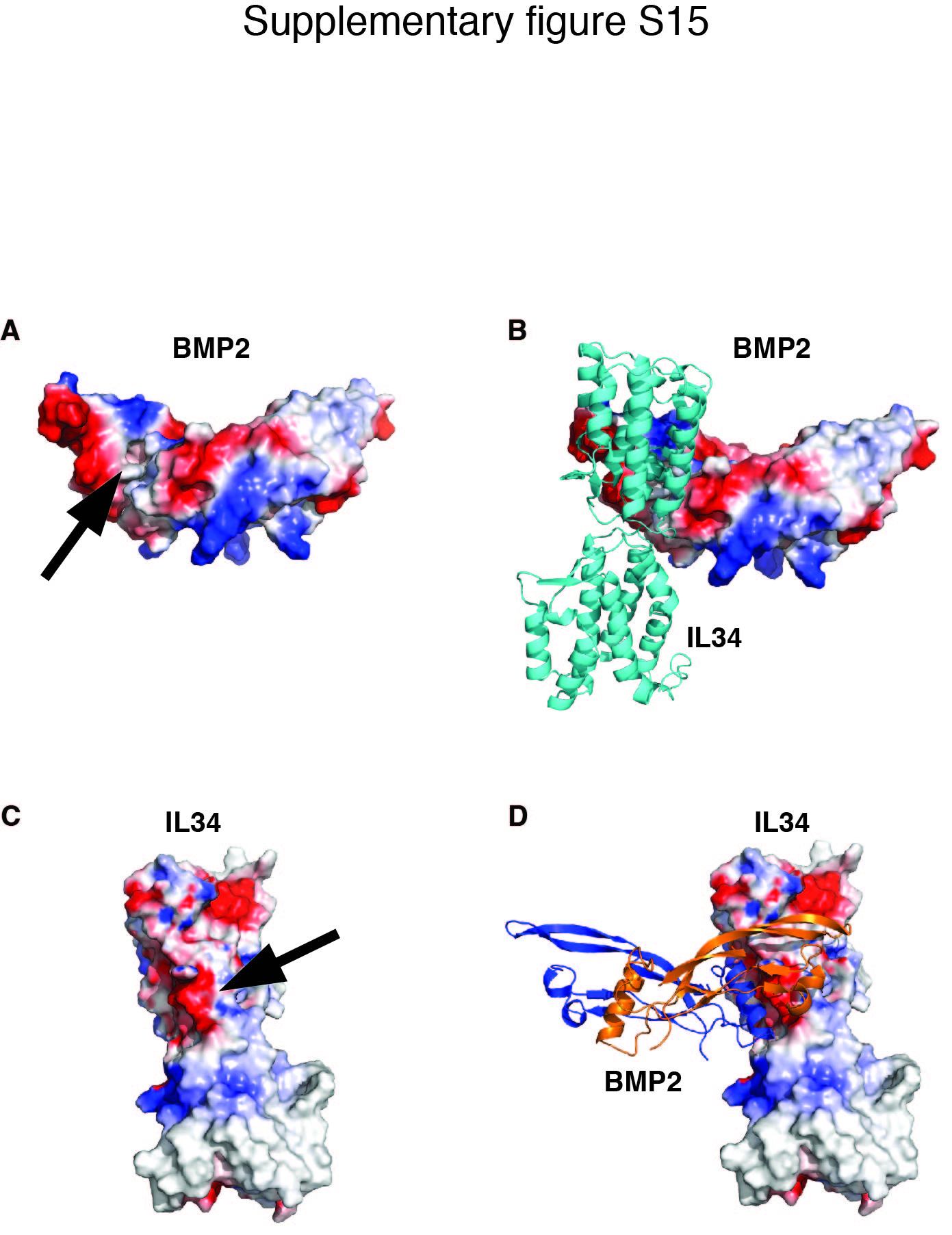
**

**Figure S15. Representative structures of the BMP2 dimer and IL34 in electrostatics surface view with the hydrophobic (white) and hydrophilic sites (red for negative patches, blue for positive patches) and identification of their respective binding sites.** (**A**) Representation of the BMP2 dimer with the knuckle site pocket underlined (arrow). (**B**) BMP2 dimer and IL34 position in cartoon representation. (**C**) IL34 with the BMP2 binding site indicated by the arrow. (**D**) IL34 and BMP2 position in cartoon representation.

**Supplementary figure 16**

All BMP/TGFß family members

ALIGN TOTAL

[NP_001191.1](https://www.ncbi.nlm.nih.gov/protein/NP_001191.1?report=genbank&log$=protalign&blast_rank=0&RID=0) BMP2 295 -SCKRHPLYVDFS-DVGW-NDWIVAPPGYHAFYCHGECPFPLADHLNSTNH AIVQTLVNSVN--SKIPKACCVPTELSAISMLYLD---ENEKVVLKNYQDMVVEGCGCR 396

[NP_001193.2](https://www.ncbi.nlm.nih.gov/protein/NP_001193.2?report=genbank&log$=protalign&blast_rank=1&RID=0) BMP4 306 KNCRRHSLYVDFS-DVGW-NDWIVAPPGYQAFYCHGDCPFPLADHLNSTNH AIVQTLVNSVN--SSIPKACCVPTELSAISMLYLD---EYDKVVLKNYQEMVVEGCGCR 408

[NP_001710.1](https://www.ncbi.nlm.nih.gov/protein/NP_001710.1?report=genbank&log$=protalign&blast_rank=2&RID=0) BMP7 328 QACKKHELYVSFR-DLGW-QDWIIAPEGYAAYYCEGECAFPLNSYMNATNH AIVQTLVHFINP-ETVPKPCCAPTQLNAISVLYFD---DSSNVILKKYRNMVVRACGCH 431

[NP_066551.1](https://www.ncbi.nlm.nih.gov/protein/NP_066551.1?report=genbank&log$=protalign&blast_rank=3&RID=0) BMP5 351 QACKKHELYVSFR-DLGW-QDWIIAPEGYAAFYCDGECSFPLNAHMNATNH AIVQTLVHLMFP-DHVPKPCCAPTKLNAISVLYFD---DSSNVILKKYRNMVVRSCGCH 454

[NP_001709.1](https://www.ncbi.nlm.nih.gov/protein/NP_001709.1?report=genbank&log$=protalign&blast_rank=4&RID=0) BMP6 410 TACRKHELYVSFQ-DLGW-QDWIIAPKGYAANYCDGECSFPLNAHMNATNH AIVQTLVHLMNP-EYVPKPCCAPTKLNAISVLYFD---DNSNVILKKYRNMVVRACGCH 513

[NP_057288.1](https://www.ncbi.nlm.nih.gov/protein/NP_057288.1?report=genbank&log$=protalign&blast_rank=5&RID=0) GDF2 325 SHCQKTSLRVNFE-DIGW-DSWIIAPKEYEAYECKGGCFFPLADDVTPTKH AIVQTLVHLKFP-TKVGKACCVPTKLSPISVLYKD--dMGVPTLKYHYEGMSVAECGCR 429

[NP_005251.1](https://www.ncbi.nlm.nih.gov/protein/NP_005251.1?report=genbank&log$=protalign&blast_rank=6&RID=0) GDF9 351 NECELHDFRLSFS-QLKW-DNWIVAPHRYNPRYCKGDCPRAVGHRYGSPV-[1]TMVQNIIYEKLD-SSVPRPSCVPAKYSPLSVLTIE---PDGSIAYKEYEDMIATKCTCR 454

[NP_000548.2](https://www.ncbi.nlm.nih.gov/protein/NP_000548.2?report=genbank&log$=protalign&blast_rank=7&RID=0) GDF5 398 ARCSRKALHVNFK-DMGW-DDWIIAPLEYEAFHCEGLCEFPLRSHLEPTNH AVIQTLMNSMDP-ESTPPTCCVPTRLSPISILFID---SANNVVYKQYEDMVVESCGCR 501

[NP_001001557.1](https://www.ncbi.nlm.nih.gov/protein/NP_001001557.1?report=genbank&log$=protalign&blast_rank=8&RID=0)GDF6 352 LRCSKKPLHVNFK-ELGW-DDWIIAPLEYEAYHCEGVCDFPLRSHLEPTNH AIIQTLMNSMDP-GSTPPSCCVPTKLTPISILYID---AGNNVVYKQYEDMVVESCGCR 455

[NP_878248.2](https://www.ncbi.nlm.nih.gov/protein/NP_878248.2?report=genbank&log$=protalign&blast_rank=9&RID=0) GDF7 347 SRCSRKPLHVDFK-ELGW-DDWIIAPLDYEAYHCEGLCDFPLRSHLEPTNH AIIQTLLNSMAP-DAAPASCCVPARLSPISILYID---AANNVVYKQYEDMVVEACGCR 450

[NP_055297.1](https://www.ncbi.nlm.nih.gov/protein/NP_055297.1?report=genbank&log$=protalign&blast_rank=11&RID=0) BMP10 321 NYCKRTPLYIDFK-EIGW-DSWIIAPPGYEAYECRGVCNYPLAEHLTPTKH AIIQALVHLKNS-QKASKACCVPTKLEPISILYLDk--GVV-TYKFKYEGMAVSECGCR 424

[NP_002183.1](https://www.ncbi.nlm.nih.gov/protein/NP_002183.1?report=genbank&log$=protalign&blast_rank=14&RID=0) INHBA 319 NICCKKQFFVSFK-DIGW-NDWIIAPSGYHANYCEGECPSHIAGTSGSSLS[4]VINHYRMRGHSP-FANLKSCCVPTKLRPMSMLYYD---DGQNIIKKDIQNMIVEECGCS 426

[NP_002184.2](https://www.ncbi.nlm.nih.gov/protein/NP_002184.2?report=genbank&log$=protalign&blast_rank=15&RID=0) INHBB 301 NLCCRQQFFIDFR-LIGW-NDWIIAPTGYYGNYCEGSCPAYLAGVPGSASS[4]VVNQYRMRGLNP--GTVNSCCIPTKLSTMSMLYFD---DEYNIVKRDVPNMIVEECGCA 407

[NP_005250.1](https://www.ncbi.nlm.nih.gov/protein/NP_005250.1?report=genbank&log$=protalign&blast_rank=19&RID=0) GDF8 281 --CCRYPLTVDFE-AFGW-D-WIIAPKRYKANYCSGECEFVFLQKYPHTH- -----LVHQANP-RGSAGPCCTPTKMSPINMLYF---nGKEQIIYGKIPAMVVDRCGCS 375

[NP_005802.1](https://www.ncbi.nlm.nih.gov/protein/NP_005802.1?report=genbank&log$=protalign&blast_rank=20&RID=0) GDF11 313 --CCRYPLTVDFE-AFGW-D-WIIAPKRYKANYCSGQCEYMFMQKYPHTH- -----LVQQANP-RGSAGPCCTPTKMSPINMLYF---nDKQQIIYGKIPGMVVDRCGCS 407

[NP_005529.1](https://www.ncbi.nlm.nih.gov/protein/NP_005529.1?report=genbank&log$=protalign&blast_rank=16&RID=0) INHBC 245 RMCCRQEFFVDFR-EIGW-HDWIIQPEGYAMNFCIGQCPLHIAGMPGIAAS[4]-VLNLLKANTAAgTTGGGSCCVPTARRPLSLLYYDr--DSN-IVKTDIPDMVVEACGCS 352

[NP_113667.1](https://www.ncbi.nlm.nih.gov/protein/NP_113667.1?report=genbank&log$=protalign&blast_rank=17&RID=0) INHBE 245 PLCCRRDHYVDFQ-ELGW-RDWILQPEGYQLNYCSGQCPPHLAGSPGIAAS[4]-VFS-LLKANNP-WPASTSCCVPTARRPLSLLYLDh-nGN--VVKTDVPDMVVEACGCS 350

[NP_000651.3](https://www.ncbi.nlm.nih.gov/protein/NP_000651.3?report=genbank&log$=protalign&blast_rank=21&RID=0) TGFB1 291 KNCCVRQLYIDFRkDLGW-K-WIHEPKGYHANFCLGPCPYIWSLD---TQY SKVLALYNQHNP-GASAAPCCVPQALEPLPIVYYV---GRK-PKVEQLSNMIVRSCKCS 390

[NP_001129071.1](https://www.ncbi.nlm.nih.gov/protein/NP_001129071.1?report=genbank&log$=protalign&blast_rank=22&RID=0)TGFB2 343 DNCCLRPLYIDFKrDLGW-K-WIHEPKGYNANFCAGACPYLWSSD---TQH SRVLSLYNTINP-EASASPCCVSQDLEPLTILYYI---GKT-PKIEQLSNMIVKSCKCS 442

[NP_003230.1](https://www.ncbi.nlm.nih.gov/protein/NP_003230.1?report=genbank&log$=protalign&blast_rank=23&RID=0) TGFB3 313 ENCCVRPLYIDFRqDLGW-K-WVHEPKGYYANFCSGPCPYLRSAD---TTH STVLGLYNTLNP-EASASPCCVPQDLEPLTILYYV---GRT-PKVEQLSNMVVKSCKCS 412

[NP_861525.2](https://www.ncbi.nlm.nih.gov/protein/NP_861525.2?report=genbank&log$=protalign&blast_rank=12&RID=0) BMP8A 299 QVCRRHELYVSFQ-DLGW-LDWVIAPQGYSAYYCEGECSFPLDSCMNATNH AILQSLVHLMKP-NAVPKACCAPTKLSATSVLYYD---SSNNVILRKHRNMVVKACGCH 402

[NP_001711.2](https://www.ncbi.nlm.nih.gov/protein/NP_001711.2?report=genbank&log$=protalign&blast_rank=13&RID=0) BMP8B 299 QVCRRHELYVSFQ-DLGW-LDWVIAPQGYSAYYCEGECSFPLDSCMNATNH AILQSLVHLMMP-DAVPKACCAPTKLSATSVLYYD---SSNNVILRKHRNMVVKACGCH 402

[NP_060525.3](https://www.ncbi.nlm.nih.gov/protein/NP_060525.3?report=genbank&log$=protalign&blast_rank=18&RID=0) NODAL 245 QLCRKVKFQVDFN-LIGW-GSWIIYPKQYNAYRCEGECPNPVGEEFHPTNH AYIQSLLKRYQP-HRVPSTCCAPVKTKPLSMLYVD--nGR--VLLDHHKDMIVEECGCL 347

[NP_004953.1](https://www.ncbi.nlm.nih.gov/protein/NP_004953.1?report=genbank&log$=protalign&blast_rank=10&RID=0) GDF10 374 RVCSRRYLKVDFA-DIGW-NEWIISPKSFDAYYCAGACEFPMPKIVRPS--[2]ATIQSIVRAVGIiPGIPEPCCVPDKMNSLGVLFLD---ENRNVVLKVYPNMSVDTCACR 478

[NP_065685.1](https://www.ncbi.nlm.nih.gov/protein/NP_065685.1?report=genbank&log$=protalign&blast_rank=24&RID=0) GDF3 262 NLCHRHQLFINFR-DLGW-HKWIIAPKGFMANYCHGECPFSLTISLNSS--[2]AFMQALMHAVDP--EIPQAVCIPTKLSPISMLYQD---NNDNVILRHYEDMVVDECGCG 364

[NP_001483.3](https://www.ncbi.nlm.nih.gov/protein/NP_001483.3?report=genbank&log$=protalign&blast_rank=25&RID=0) GDF1 265 GACRARRLYVSFR-EVGW-HRWVIAPRGFLANYCQGQCALPVALSGSGGPP[4]AVLRALMHAAAP-GAADLPCCVPARLSPISVLFFD---NSDNVVLRQYEDMVVDECGCR 372

[NP_001192.4](https://www.ncbi.nlm.nih.gov/protein/NP_001192.4?report=genbank&log$=protalign&blast_rank=26&RID=0) BMP3 368 RNCARRYLKVDFA-DIGW-SEWIISPKSFDAYYCSGACQFPMPKSLKPS--[2]ATIQSIVRAVGVvPGIPEPCCVPEKMSSLSILFFD---ENKNVVLKVYPNMTVESCACR 472

[NP_066277.1](https://www.ncbi.nlm.nih.gov/protein/NP_066277.1?report=genbank&log$=protalign&blast_rank=27&RID=0) LEFTY1 261 TRCCRQEMYIDLQ-GMKWaENWVLEPPGFLAYECVGTCRQPPEALAFKWPF[3]------------------RQCIASETDSLPMIVSIkegGRTRPQVVSLPNMRVQKCSCA[12] 366

[NP_003231.2](https://www.ncbi.nlm.nih.gov/protein/NP_003231.2?report=genbank&log$=protalign&blast_rank=28&RID=0) LEFTY2 261 TRCCRQEMYIDLQ-GMKWaKNWVLEPPGFLAYECVGTCQQPPEALAFNWPF[3]------------------RQCIASETASLPMIVSIkegGRTRPQVVSLPNMRVQKCSCA[12] 366

BMP2

NP_001191.1 bone morphogenetic protein 2 preproprotein [Homo sapiens]

NP_031579.2 bone morphogenetic protein 2 preproprotein [Mus musculus]

NP_058874.2 bone morphogenetic protein 2 precursor [Rattus norvegicus]

NP_001092611.1 bone morphogenetic protein 2 precursor [Bos taurus]

NP_001182328.1 bone morphogenetic protein 2 precursor [Sus scrofa]

XP_038289102.1 bone morphogenetic protein 2 isoform X1 [Canis lupus familiaris]

XP_023481904.1 bone morphogenetic protein 2 isoform X1 [Equus caballus]

XP_004014402.1 bone morphogenetic protein 2 [Ovis aries]

XP_514508.2 bone morphogenetic protein 2 [Pan troglodytes]

XP_001115987.1 bone morphogenetic protein 2 [Macaca mulatta]

XP_003983818.1 bone morphogenetic protein 2 isoform X2 [Felis catus]

NP_001274493.1 bone morphogenetic protein 2 precursor [Capra hircus]

XP_004061840.1 bone morphogenetic protein 2 [Gorilla gorilla gorilla]

XP_002829993.1 bone morphogenetic protein 2 [Pongo abelii]

XP_031998602.1 bone morphogenetic protein 2 [Hylobates moloch]

[NP_001191.1](https://www.ncbi.nlm.nih.gov/protein/NP_001191.1?report=genbank&log$=protalign&blast_rank=0&RID=0) 1 ------------------------------------MVAGTRCLLALLLPQVLLGGAAGLVPELGRRKFAAAS--SGRPS 42

[NP_031579.2](https://www.ncbi.nlm.nih.gov/protein/NP_031579.2?report=genbank&log$=protalign&blast_rank=1&RID=0) 1 ------------------------------------MVAGTRCLLVLLLPQVLLGGAAGLIPELGRKKFAAAS---SRPL 41

[NP_058874.2](https://www.ncbi.nlm.nih.gov/protein/NP_058874.2?report=genbank&log$=protalign&blast_rank=2&RID=0) 1 ------------------------------------MVAGTRCLLVLLLPQVLLGGAAGLIPELGRKKFAGAS---GRPL 41

[NP_001092611.1](https://www.ncbi.nlm.nih.gov/protein/NP_001092611.1?report=genbank&log$=protalign&blast_rank=3&RID=0) 1 ------------------------------------MVAGTRCLLALLLPQVLLGGAAGLIPELGRRKFAAS---AGRSS 41

[NP_001182328.1](https://www.ncbi.nlm.nih.gov/protein/NP_001182328.1?report=genbank&log$=protalign&blast_rank=4&RID=0) 1 ------------------------------------MVAGTRCLLALLLPQVLLGGAADLIPELGRRKFAAS---TGLSS 41

[XP_038289102.1](https://www.ncbi.nlm.nih.gov/protein/XP_038289102.1?report=genbank&log$=protalign&blast_rank=5&RID=0) 1 mclglfsdpggvrpsggfprfparavsasvteerstMVAGTRCLLALLLPQVLLGGAAGLITELGRRKFAAS---PGRSP 77

[XP_023481904.1](https://www.ncbi.nlm.nih.gov/protein/XP_023481904.1?report=genbank&log$=protalign&blast_rank=6&RID=0) 1 ------------------------------------MVAGTHCLLALLLPQVLLGGAAGLIPELGRRKFAAS---TGRSS 41

[XP_004014402.1](https://www.ncbi.nlm.nih.gov/protein/XP_004014402.1?report=genbank&log$=protalign&blast_rank=7&RID=0) 1 ------------------------------------MVAGTRCLLALLLPQVLLGGAAGLIPELGRRKFAAS---AGRSS 41

[XP_514508.2](https://www.ncbi.nlm.nih.gov/protein/XP_514508.2?report=genbank&log$=protalign&blast_rank=8&RID=0) 1 ------------------------------------MVAGTRCLLALLLPQVLLGGAAGLVPELGRRKFAAAAaaSGRPS 44

[XP_001115987.1](https://www.ncbi.nlm.nih.gov/protein/XP_001115987.1?report=genbank&log$=protalign&blast_rank=9&RID=0) 1 ------------------------------------MVAGTRCLLALLLPQVLLGGAAGLVPELGRRKFAASS--SGRPS 42

[XP_003983818.1](https://www.ncbi.nlm.nih.gov/protein/XP_003983818.1?report=genbank&log$=protalign&blast_rank=10&RID=0) 1 ------------------------------------MVAGTRCLLALLLPQVLLGGAAGLIPELGRRKFAAS---TGRSS 41

[NP_001274493.1](https://www.ncbi.nlm.nih.gov/protein/NP_001274493.1?report=genbank&log$=protalign&blast_rank=11&RID=0) 1 ------------------------------------MVAGTRCLLALLLPQVLLGGAAGLIPELGRRKFAAS---AGRSS 41

[XP_004061840.1](https://www.ncbi.nlm.nih.gov/protein/XP_004061840.1?report=genbank&log$=protalign&blast_rank=12&RID=0) 1 ------------------------------------MVAGTRCLLALLLPQVLLGGAAGLVPELGRRKFAAAS--SGRPS 42

[XP_002829993.1](https://www.ncbi.nlm.nih.gov/protein/XP_002829993.1?report=genbank&log$=protalign&blast_rank=13&RID=0) 1 ------------------------------------MVAGTRCLLALLLPQVLLGGAAGLVPELGRRKFAASS--SGRPS 42

[XP_031998602.1](https://www.ncbi.nlm.nih.gov/protein/XP_031998602.1?report=genbank&log$=protalign&blast_rank=14&RID=0) 1 ------------------------------------MVAGTRCLLALLLPQVLLGGAAGLVPELGRRKFAAAS--SGRPS 42

[NP_001191.1](https://www.ncbi.nlm.nih.gov/protein/NP_001191.1?report=genbank&log$=protalign&blast_rank=0&RID=0) 43 SQPSDEVLSEFELRLLSMFGLKQRPTPSRDAVVPPYMLDLYRRHSGQPGSPAPDHRLERAASRANTVRSFHHEESLEELP 122

[NP_031579.2](https://www.ncbi.nlm.nih.gov/protein/NP_031579.2?report=genbank&log$=protalign&blast_rank=1&RID=0) 42 SRPSEDVLSEFELRLLSMFGLKQRPTPSKDVVVPPYMLDLYRRHSGQPGAPAPDHRLERAASRANTVRSFHHEEAVEELP 121

[NP_058874.2](https://www.ncbi.nlm.nih.gov/protein/NP_058874.2?report=genbank&log$=protalign&blast_rank=2&RID=0) 42 SRPSDDVLSEFELRLLSMFGLKQRPTPSKDVVVPPYMLDLYRRHSGQPGAPAPDHRLERAASRANTVRSFHHEEAIEELP 121

[NP_001092611.1](https://www.ncbi.nlm.nih.gov/protein/NP_001092611.1?report=genbank&log$=protalign&blast_rank=3&RID=0) 42 SQPSDDVLSEFELRLLSMFGLKQRPTPSRDAVVPPYMLDLYRQHSGQPGAPAPDHRLERAASLANTVRSFHHEESLEELP 121

[NP_001182328.1](https://www.ncbi.nlm.nih.gov/protein/NP_001182328.1?report=genbank&log$=protalign&blast_rank=4&RID=0) 42 SQPSDDVLSEFELRLLSMFGLKQRPTPSRDAVVPPYMLDLYRRHSGQPGAPAPDHRLERAASLANTVRSFHHEESLEELP 121

[XP_038289102.1](https://www.ncbi.nlm.nih.gov/protein/XP_038289102.1?report=genbank&log$=protalign&blast_rank=5&RID=0) 78 SQPSDEVLSEFELRLLSMFGLKRRPTPSRDAVVPPYMLDLYRRHSGQPGAPAPDHRLERAASLANTVRSFHHEESLEELP 157

[XP_023481904.1](https://www.ncbi.nlm.nih.gov/protein/XP_023481904.1?report=genbank&log$=protalign&blast_rank=6&RID=0) 42 SQPSDDVLSEFELRLLSMFGLKQRPTPSRDAVVPPYMLDLYRRHSGQPDAPAPDHRLERAASLANTVRSFHHEESLEELP 121

[XP_004014402.1](https://www.ncbi.nlm.nih.gov/protein/XP_004014402.1?report=genbank&log$=protalign&blast_rank=7&RID=0) 42 SQPSDEVLSEFELRLLSMFGLKQRPTPSRDAVVPPYMLDLYRQHSGQPGAPAPDHRLERAASLANTVRSFHHEESLEELP 121

[XP_514508.2](https://www.ncbi.nlm.nih.gov/protein/XP_514508.2?report=genbank&log$=protalign&blast_rank=8&RID=0) 45 SQPSDEVLSEFELRLLSMFGLKQRPTPSRDAVVPPYMLDLYRRHSGQPGSPAPDHRLERAASRANTVRSFHHEESLEELP 124

[XP_001115987.1](https://www.ncbi.nlm.nih.gov/protein/XP_001115987.1?report=genbank&log$=protalign&blast_rank=9&RID=0) 43 SQPSDEVLSEFELRLLSMFGLKQRPTPSRDAVVPPYMLDLYRRHSGQPGSPAPDHRLERAASRANTVRSFHHEESLEELP 122

[XP_003983818.1](https://www.ncbi.nlm.nih.gov/protein/XP_003983818.1?report=genbank&log$=protalign&blast_rank=10&RID=0) 42 SQPSDEVLSEFELRLLSMFGLKRRPTPSRDAVVPPYMLDLYRRHSGQPGAPAPDHRLERAASLANTVRSFHHEESVEELP 121

[NP_001274493.1](https://www.ncbi.nlm.nih.gov/protein/NP_001274493.1?report=genbank&log$=protalign&blast_rank=11&RID=0) 42 SQPSDEVLSEFELRLLSMFGLKQRPTPSRDAVVPPYMLDLYRQHSGQPGAPAPDHRLERAASLANTVRSFHHEESLEELP 121

[XP_004061840.1](https://www.ncbi.nlm.nih.gov/protein/XP_004061840.1?report=genbank&log$=protalign&blast_rank=12&RID=0) 43 SQPSDEVLSEFELRLLSMFGLKQRPTPSRDAVVPPYMLDLYRRHSGQPGSPAPDHRLERAASRANTVRSFHHEESLEELP 122

[XP_002829993.1](https://www.ncbi.nlm.nih.gov/protein/XP_002829993.1?report=genbank&log$=protalign&blast_rank=13&RID=0) 43 SQPSDEVLSEFELRLLSMFGLKQRPTPSRDAVVPPYMLDLYHRHSGQPGSPAPDHRLERAASRANTVRSFHHEESLEELP 122

[XP_031998602.1](https://www.ncbi.nlm.nih.gov/protein/XP_031998602.1?report=genbank&log$=protalign&blast_rank=14&RID=0) 43 SQPSDEVLSEFELRLLSMFGLKQRPTPSRDAVVPPYMLELYRRHSGQPGSPAPDHRLERAASRANTVRSFHHEESLEELP 122

[NP_001191.1](https://www.ncbi.nlm.nih.gov/protein/NP_001191.1?report=genbank&log$=protalign&blast_rank=0&RID=0) 123 ETSGKTTRRFFFNLSSIPTEEFITSAELQVFREQMQDALGNNSSFHHRINIYEIIKPATANSKFPVTRLLDTRLVNQNAS 202

[NP_031579.2](https://www.ncbi.nlm.nih.gov/protein/NP_031579.2?report=genbank&log$=protalign&blast_rank=1&RID=0) 122 EMSGKTARRFFFNLSSVPSDEFLTSAELQIFREQIQEALGN-SSFQHRINIYEIIKPAAANLKFPVTRLLDTRLVNQNTS 200

[NP_058874.2](https://www.ncbi.nlm.nih.gov/protein/NP_058874.2?report=genbank&log$=protalign&blast_rank=2&RID=0) 122 EMSGKTSRRFFFNLSSVPTDEFLTSAELQIFREQMQEALGN-SSFQHRINIYEIIKPATASSKFPVTRLLDTRLVTQNTS 200

[NP_001092611.1](https://www.ncbi.nlm.nih.gov/protein/NP_001092611.1?report=genbank&log$=protalign&blast_rank=3&RID=0) 122 EMSGKTTRRFFFNLTSIPTEEFITSAELQVFRKHMPEALENNSSFHHRINIYEIIKPATANSKFPVTRLLDTRLVTQNAS 201

[NP_001182328.1](https://www.ncbi.nlm.nih.gov/protein/NP_001182328.1?report=genbank&log$=protalign&blast_rank=4&RID=0) 122 EMSGKTTRRFFFNLTSVPTEEFITSAELQVFREQTQETLDNSSSFHHRINIYEIIKPATANSKFPVTRLLDTRLVTPNAS 201

[XP_038289102.1](https://www.ncbi.nlm.nih.gov/protein/XP_038289102.1?report=genbank&log$=protalign&blast_rank=5&RID=0) 158 EMSGKTTRRFFFNLTSIPTDEFITSAELQVFREQMQEPLENDSNFHHRINIYEIIKPAAANLKFPVTRLLDTRLVNQNAS 237

[XP_023481904.1](https://www.ncbi.nlm.nih.gov/protein/XP_023481904.1?report=genbank&log$=protalign&blast_rank=6&RID=0) 122 EMSGKTTRRFFFNLTSIPTEEFITSAELQVFREQMQDPWENNSNFHHRINIYEIIKPATANSKFPVTRLLDTRLVNQNAS 201

[XP_004014402.1](https://www.ncbi.nlm.nih.gov/protein/XP_004014402.1?report=genbank&log$=protalign&blast_rank=7&RID=0) 122 EMSGKTTRRFFFNLTSIPTEEFITSAELQVFRKHMPEALENNSSFHHRINIYEIIKPATANSKFPVTRLLDTRLVTQNAS 201

[XP_514508.2](https://www.ncbi.nlm.nih.gov/protein/XP_514508.2?report=genbank&log$=protalign&blast_rank=8&RID=0) 125 ETSGKTTRRFFFNLSSIPTEESITSAELQVFREQMRDALGNNSSFHHRINIYEIIKPATANSKFPVTRLLDTRLVNQNAS 204

[XP_001115987.1](https://www.ncbi.nlm.nih.gov/protein/XP_001115987.1?report=genbank&log$=protalign&blast_rank=9&RID=0) 123 EMSGKTTRRFFFNLSSIPTEEFVTSAELQVFREQMQDALGDNSSFHHRINIYEIIKPATANSKFPVTRLLDTRLVNQNAS 202

[XP_003983818.1](https://www.ncbi.nlm.nih.gov/protein/XP_003983818.1?report=genbank&log$=protalign&blast_rank=10&RID=0) 122 EMSGKTTRRFFFNLTSIPTDEFITSAELQVFREQMQETLENSSSFHHRINIYEIIKPATANLKFPMTRLLDTRLVNQNTS 201

[NP_001274493.1](https://www.ncbi.nlm.nih.gov/protein/NP_001274493.1?report=genbank&log$=protalign&blast_rank=11&RID=0) 122 EMSGKTTRRFFFNLTSIPTEEFITSAELQVFRKHMPEALENNSSFHHRINIYEIIKPATANSKFPVTRLLDTRLVTQNAS 201

[XP_004061840.1](https://www.ncbi.nlm.nih.gov/protein/XP_004061840.1?report=genbank&log$=protalign&blast_rank=12&RID=0) 123 ETSGKTTRRFFFNLSSIPTEEFITSAELQVFREQMQDALGNNSSFHHRINIYEIIKPATANSKFPVTRLLDTRLVNQNAS 202

[XP_002829993.1](https://www.ncbi.nlm.nih.gov/protein/XP_002829993.1?report=genbank&log$=protalign&blast_rank=13&RID=0) 123 ETSGKTTRRFFFNLSSIPTEEFITSAELQVFREQMQDALGNNSSFHHRINIYEIIKPATANSKFPMTRLLDTRLVNQNAS 202

[XP_031998602.1](https://www.ncbi.nlm.nih.gov/protein/XP_031998602.1?report=genbank&log$=protalign&blast_rank=14&RID=0) 123 ETSGKTTRRFFFNLSSIPTEEFITSAELQVFREQMQDALGNNSSFHHRINIYEIIKPATANLKFPVTRLLDTRLVNQNAS 202

[NP_001191.1](https://www.ncbi.nlm.nih.gov/protein/NP_001191.1?report=genbank&log$=protalign&blast_rank=0&RID=0) 203 RWESFDVTPAVMRWTAQGHANHGFVVEVAHLEEKQGVSKRHVRISRSLHQDEHSWSQIRPLLVTFGHDGKGHPLHKREKR 282

[NP_031579.2](https://www.ncbi.nlm.nih.gov/protein/NP_031579.2?report=genbank&log$=protalign&blast_rank=1&RID=0) 201 QWESFDVTPAVMRWTTQGHTNHGFVVEVAHLEENPGVSKRHVRISRSLHQDEHSWSQIRPLLVTFGHDGKGHPLHKREKR 280

[NP_058874.2](https://www.ncbi.nlm.nih.gov/protein/NP_058874.2?report=genbank&log$=protalign&blast_rank=2&RID=0) 201 QWESFDVTPAVMRWTAQGHTNHGFVVEVAHLEEKPGVSKRHVRISRSLHQDEHSWSQVRPLLVTFGHDGKGHPLHKREKR 280

[NP_001092611.1](https://www.ncbi.nlm.nih.gov/protein/NP_001092611.1?report=genbank&log$=protalign&blast_rank=3&RID=0) 202 RWESFDVTPAVMRWTAQGLTNHGFVVEVAHPEDSHGASKRHVRISRSLHQDEHSWSQIRPLLVTFGHDGKGHPLHRREKR 281

[NP_001182328.1](https://www.ncbi.nlm.nih.gov/protein/NP_001182328.1?report=genbank&log$=protalign&blast_rank=4&RID=0) 202 RWESFDVTPAVMRWTAQGVANHGFVVEVAHPEDSPEVSKRHVRISRSLHQDEHSWSQIRPLLVTFGHDGKGHPLHKREKR 281

[XP_038289102.1](https://www.ncbi.nlm.nih.gov/protein/XP_038289102.1?report=genbank&log$=protalign&blast_rank=5&RID=0) 238 RWESFDVTPAVMRWTAQGLANHGFVVEVTHLEENQGVSKRHVRISRSLHQDEHSWSQIRPLLVTFGHDGKGHPLHKREKR 317

[XP_023481904.1](https://www.ncbi.nlm.nih.gov/protein/XP_023481904.1?report=genbank&log$=protalign&blast_rank=6&RID=0) 202 RWERFDVTPAVMRWTAQGLANHGFVVEVAHLEENRGASKRHVRISRSLHQDEHSWSQIRPLLVTFGHDGKGHPLHKREKR 281

[XP_004014402.1](https://www.ncbi.nlm.nih.gov/protein/XP_004014402.1?report=genbank&log$=protalign&blast_rank=7&RID=0) 202 RWESFDVTPAVMRWTAQGLTNHGFVVEVAHPEDSHGASKRHVRISRSLHQDEHSWSQIRPLLVTFGHDGKGHPLHRREKR 281

[XP_514508.2](https://www.ncbi.nlm.nih.gov/protein/XP_514508.2?report=genbank&log$=protalign&blast_rank=8&RID=0) 205 RWESFDVTPAVMRWTAQGHANHGFVVEVAHLEEKQGVSKRHVRISRSLHQDEHSWSQIRPLLVTFGHDGKGHPLHKREKR 284

[XP_001115987.1](https://www.ncbi.nlm.nih.gov/protein/XP_001115987.1?report=genbank&log$=protalign&blast_rank=9&RID=0) 203 RWESFDVTPAVMRWTAQGHANHGFVVEVTHLEEKQGVSKRHVRISRSLHQDEHSWSQIRPLLVTFGHDGKGHPLHKREKR 282

[XP_003983818.1](https://www.ncbi.nlm.nih.gov/protein/XP_003983818.1?report=genbank&log$=protalign&blast_rank=10&RID=0) 202 RWESFDVTPAVMRWTAQGLTNHGFVVEVTHLEENQGVSKRHVRISRSLHQDEHSWSQIRPLLVTFGHDGKGHPLHKREKR 281

[NP_001274493.1](https://www.ncbi.nlm.nih.gov/protein/NP_001274493.1?report=genbank&log$=protalign&blast_rank=11&RID=0) 202 RWESFDVTPAVMRWTAQGLTNHGFVVEVAHPEDSHGASKRHVRISRSLHQDEHSWSQIRPLLVTFGHDGKGHPLHRREKR 281

[XP_004061840.1](https://www.ncbi.nlm.nih.gov/protein/XP_004061840.1?report=genbank&log$=protalign&blast_rank=12&RID=0) 203 RWESFDVTPAVMRWTAQGHANHGFVVEVTHLEEKQGVSKRHVRISRSLHQDEHSWSQIRPLLVTFGHDGKGHPLHKREKR 282

[XP_002829993.1](https://www.ncbi.nlm.nih.gov/protein/XP_002829993.1?report=genbank&log$=protalign&blast_rank=13&RID=0) 203 RWESFDVTPAVMRWTAQGHANHGFVVEVTHLEEKQGVSKRHVRISRSLHQDEHSWSQIRPLLVTFGHDGKGHPLHKREKR 282

[XP_031998602.1](https://www.ncbi.nlm.nih.gov/protein/XP_031998602.1?report=genbank&log$=protalign&blast_rank=14&RID=0) 203 RWESFDVTPAVMRWTAQGHANHGFVVEVTHLEEKQGVSKRHVRISRSLHQDEHSWSQIRPLLVTFGHDGKGHPLHKREKR 282

[NP_001191.1](https://www.ncbi.nlm.nih.gov/protein/NP_001191.1?report=genbank&log$=protalign&blast_rank=0&RID=0) 283 QAKHKQRKRLKSSCKRHPLYVDFSDVGWNDWIVAPPGYHAFYCHGECPFPLADHLNSTNHAIVQTLVNSVNSKIPKACCV 362

[NP_031579.2](https://www.ncbi.nlm.nih.gov/protein/NP_031579.2?report=genbank&log$=protalign&blast_rank=1&RID=0) 281 QAKHKQRKRLKSSCKRHPLYVDFSDVGWNDWIVAPPGYHAFYCHGECPFPLADHLNSTNHAIVQTLVNSVNSKIPKACCV 360

[NP_058874.2](https://www.ncbi.nlm.nih.gov/protein/NP_058874.2?report=genbank&log$=protalign&blast_rank=2&RID=0) 281 QAKHKQRKRLKSSCKRHPLYVDFSDVGWNDWIVAPPGYHAFYCHGECPFPLADHLNSTNHAIVQTLVNSVNSKIPKACCV 360

[NP_001092611.1](https://www.ncbi.nlm.nih.gov/protein/NP_001092611.1?report=genbank&log$=protalign&blast_rank=3&RID=0) 282 QAKHKQRKRLKSSCKRHPLYVDFSDVGWNDWIVAPPGYHAFYCHGECPFPLADHLNSTNHAIVQTLVNSVNSKIPKACCV 361

[NP_001182328.1](https://www.ncbi.nlm.nih.gov/protein/NP_001182328.1?report=genbank&log$=protalign&blast_rank=4&RID=0) 282 QAKHKQRKRLKSSCKRHPLYVDFSDVGWNDWIVAPPGYHAFYCHGECPFPLADHLNSTNHAIVQTLVNSVNSKIPKACCV 361

[XP_038289102.1](https://www.ncbi.nlm.nih.gov/protein/XP_038289102.1?report=genbank&log$=protalign&blast_rank=5&RID=0) 318 QAKHKQRKRLKSSCKRHPLYVDFSDVGWNDWIVAPPGYHAFYCHGECPFPLADHLNSTNHAIVQTLVNSVNSKIPKACCV 397

[XP_023481904.1](https://www.ncbi.nlm.nih.gov/protein/XP_023481904.1?report=genbank&log$=protalign&blast_rank=6&RID=0) 282 QAKHKQRKRLKSSCKRHPLYVDFSDVGWNDWIVAPPGYHAFYCHGECPFPLADHLNSTNHAIVQTLVNSVNSKIPKACCV 361

[XP_004014402.1](https://www.ncbi.nlm.nih.gov/protein/XP_004014402.1?report=genbank&log$=protalign&blast_rank=7&RID=0) 282 QAKHKQRKRLKSSCKRHPLYVDFSDVGWNDWIVAPPGYHAFYCHGECPFPLADHLNSTNHAIVQTLVNSVNSKIPKACCV 361

[XP_514508.2](https://www.ncbi.nlm.nih.gov/protein/XP_514508.2?report=genbank&log$=protalign&blast_rank=8&RID=0) 285 QAKHKQRKRLKSSCKRHPLYVDFSDVGWNDWIVAPPGYHAFYCHGECPFPLADHLNSTNHAIVQTLVNSVNSKIPKACCV 364

[XP_001115987.1](https://www.ncbi.nlm.nih.gov/protein/XP_001115987.1?report=genbank&log$=protalign&blast_rank=9&RID=0) 283 QAKHKQRKRLKSSCKRHPLYVDFSDVGWNDWIVAPPGYHAFYCHGECPFPLADHLNSTNHAIVQTLVNSVNSKIPKACCV 362

[XP_003983818.1](https://www.ncbi.nlm.nih.gov/protein/XP_003983818.1?report=genbank&log$=protalign&blast_rank=10&RID=0) 282 QAKHKQRKRLKSSCKRHPLYVDFSDVGWNDWIVAPPGYHAFYCHGECPFPLADHLNSTNHAIVQTLVNSVNSKIPKACCV 361

[NP_001274493.1](https://www.ncbi.nlm.nih.gov/protein/NP_001274493.1?report=genbank&log$=protalign&blast_rank=11&RID=0) 282 QAKHKQRKRLKSSCKRHPLYVDFSDVGWNDWIVAPPGYHAFYCHGECPFPLADHLNSTNHAIVQTLVNSVNSKIPKACCV 361

[XP_004061840.1](https://www.ncbi.nlm.nih.gov/protein/XP_004061840.1?report=genbank&log$=protalign&blast_rank=12&RID=0) 283 QAKHKQRKRLKSSCKRHPLYVDFSDVGWNDWIVAPPGYHAFYCHGECPFPLADHLNSTNHAIVQTLVNSVNSKIPKACCV 362

[XP_002829993.1](https://www.ncbi.nlm.nih.gov/protein/XP_002829993.1?report=genbank&log$=protalign&blast_rank=13&RID=0) 283 QAKHKQRKRLKSSCKRHPLYVDFSDVGWNDWIVAPPGYHAFYCHGECPFPLADHLNSTNHAIVQTLVNSVNSKIPKACCV 362

[XP_031998602.1](https://www.ncbi.nlm.nih.gov/protein/XP_031998602.1?report=genbank&log$=protalign&blast_rank=14&RID=0) 283 QAKHKQRKRLKSSCKRHPLYVDFSDVGWNDWIVAPPGYHAFYCHGECPFPLADHLNSTNHAIVQTLVNSVNSKIPKACCV 362

[NP_001191.1](https://www.ncbi.nlm.nih.gov/protein/NP_001191.1?report=genbank&log$=protalign&blast_rank=0&RID=0) 363 PTELSAISMLYLDENEKVVLKNYQDMVVEGCGCR 396

[NP_031579.2](https://www.ncbi.nlm.nih.gov/protein/NP_031579.2?report=genbank&log$=protalign&blast_rank=1&RID=0) 361 PTELSAISMLYLDENEKVVLKNYQDMVVEGCGCR 394

[NP_058874.2](https://www.ncbi.nlm.nih.gov/protein/NP_058874.2?report=genbank&log$=protalign&blast_rank=2&RID=0) 361 PTELSAISMLYLDENEKVVLKNYQDMVVEGCGCR 394

[NP_001092611.1](https://www.ncbi.nlm.nih.gov/protein/NP_001092611.1?report=genbank&log$=protalign&blast_rank=3&RID=0) 362 PTELSAISMLYLDENEKVVLKNYQDMVVEGCGCR 395

[NP_001182328.1](https://www.ncbi.nlm.nih.gov/protein/NP_001182328.1?report=genbank&log$=protalign&blast_rank=4&RID=0) 362 PTELSAISMLYLDENEKVVLKNYQDMVVEGCGCR 395

[XP_038289102.1](https://www.ncbi.nlm.nih.gov/protein/XP_038289102.1?report=genbank&log$=protalign&blast_rank=5&RID=0) 398 PTELSAISMLYLDENEKVVLKNYQDMVVEGCGCR 431

[XP_023481904.1](https://www.ncbi.nlm.nih.gov/protein/XP_023481904.1?report=genbank&log$=protalign&blast_rank=6&RID=0) 362 PTELSAISMLYLDENEKVVLKNYQDMVVEGCGCR 395

[XP_004014402.1](https://www.ncbi.nlm.nih.gov/protein/XP_004014402.1?report=genbank&log$=protalign&blast_rank=7&RID=0) 362 PTELSAISMLYLDENEKVVLKNYQDMVVEGCGCR 395

[XP_514508.2](https://www.ncbi.nlm.nih.gov/protein/XP_514508.2?report=genbank&log$=protalign&blast_rank=8&RID=0) 365 PTELSAISMLYLDENEKVVLKNYQDMVVEGCGCR 398

[XP_001115987.1](https://www.ncbi.nlm.nih.gov/protein/XP_001115987.1?report=genbank&log$=protalign&blast_rank=9&RID=0) 363 PTELSAISMLYLDENEKVVLKNYQDMVVEGCGCR 396

[XP_003983818.1](https://www.ncbi.nlm.nih.gov/protein/XP_003983818.1?report=genbank&log$=protalign&blast_rank=10&RID=0) 362 PTELSAISMLYLDENEKVVLKNYQDMVVEGCGCR 395

[NP_001274493.1](https://www.ncbi.nlm.nih.gov/protein/NP_001274493.1?report=genbank&log$=protalign&blast_rank=11&RID=0) 362 PTELSAISMLYLDENEKVVLKNYQDMVVEGCGCR 395

[XP_004061840.1](https://www.ncbi.nlm.nih.gov/protein/XP_004061840.1?report=genbank&log$=protalign&blast_rank=12&RID=0) 363 PTELSAISMLYLDENEKVVLKNYQDMVVEGCGCR 396

[XP_002829993.1](https://www.ncbi.nlm.nih.gov/protein/XP_002829993.1?report=genbank&log$=protalign&blast_rank=13&RID=0) 363 PTELSAISMLYLDENEKVVLKNYQDMVVEGCGCR 396

[XP_031998602.1](https://www.ncbi.nlm.nih.gov/protein/XP_031998602.1?report=genbank&log$=protalign&blast_rank=14&RID=0) 363 PTELSAISMLYLDENEKVVLKNYQDMVVEGCGCR 396

BMP4

NP_001193.2 bone morphogenetic protein 4 isoform a preproprotein [Homo sapiens]

NP_031580.2 bone morphogenetic protein 4 preproprotein [Mus musculus]

NP_036959.2 bone morphogenetic protein 4 precursor [Rattus norvegicus]

XP_024853077.1 bone morphogenetic protein 4 isoform X2 [Bos taurus]

XP_020925510.1 bone morphogenetic protein 4 isoform X2 [Sus scrofa]

XP_038528824.1 bone morphogenetic protein 4 isoform X1 [Canis lupus familiaris]

XP_023483474.1 bone morphogenetic protein 4 isoform X2 [Equus caballus]

NP_001103747.1 bone morphogenetic protein 4 precursor [Ovis aries]

XP_024204496.1 bone morphogenetic protein 4 isoform X2 [Pan troglodytes]

XP_028707390.1 bone morphogenetic protein 4 isoform X2 [Macaca mulatta]

XP_019688586.1 bone morphogenetic protein 4 isoform X1 [Felis catus]

NP_001272575.1 bone morphogenetic protein 4 precursor [Capra hircus]

XP_030857866.1 bone morphogenetic protein 4 isoform X2 [Gorilla gorilla gorilla]

XP_024087813.1 bone morphogenetic protein 4 isoform X3 [Pongo abelii]

XP_032036116.1 bone morphogenetic protein 4 isoform X2 [Hylobates moloch]

[NP_001193.2](https://www.ncbi.nlm.nih.gov/protein/NP_001193.2?report=genbank&log$=protalign&blast_rank=0&RID=0) 1 MIPG-----------------------------------------------NRMLMVVLLCQVLLGGASHASLIPETGKK 33

[NP_031580.2](https://www.ncbi.nlm.nih.gov/protein/NP_031580.2?report=genbank&log$=protalign&blast_rank=1&RID=0) 1 MIPG-----------------------------------------------NRMLMVVLLCQVLLGGASHASLIPETGKK 33

[NP_036959.2](https://www.ncbi.nlm.nih.gov/protein/NP_036959.2?report=genbank&log$=protalign&blast_rank=2&RID=0) 1 MIPG-----------------------------------------------NRMLMVVLLCQVLLGGASHASLIPETGKK 33

[XP_024853077.1](https://www.ncbi.nlm.nih.gov/protein/XP_024853077.1?report=genbank&log$=protalign&blast_rank=3&RID=0) 1 MQEGRGGRRGETGAELGPEARSHSVVPSRATHCRSSSEPFQQVCSRLAVKNHGLLLYALFSVILLGGASHASLIPETGKK 80

[XP_020925510.1](https://www.ncbi.nlm.nih.gov/protein/XP_020925510.1?report=genbank&log$=protalign&blast_rank=4&RID=0) 1 MQEGRGGGREVKGVELCPEARSHSVVPSRATHCRSSSEPFQQVCSRLAVKNHGLLLYALFSVILLGGASHASLIPETGKK 80

[XP_038528824.1](https://www.ncbi.nlm.nih.gov/protein/XP_038528824.1?report=genbank&log$=protalign&blast_rank=5&RID=0) 1 MHEGRGGGREGRRAEPCPEARSHSVVPSRATHCRSSSEPFQQVCSRLAVKNHGLLLYALFSVILLGGSSHASLIPETGKK 80

[XP_023483474.1](https://www.ncbi.nlm.nih.gov/protein/XP_023483474.1?report=genbank&log$=protalign&blast_rank=6&RID=0) 1 MQEGRGGGREGRRAEPGPEARSHSVVPSRATHCRSSSEPFQQVCSRLAVKNHGLLLYALFSVILLGGASHASLIPETGKK 80

[NP_001103747.1](https://www.ncbi.nlm.nih.gov/protein/NP_001103747.1?report=genbank&log$=protalign&blast_rank=7&RID=0) 1 MIPG-----------------------------------------------NRMLMVVLLCQVLLGGASHASLIPETGKK 33

[XP_024204496.1](https://www.ncbi.nlm.nih.gov/protein/XP_024204496.1?report=genbank&log$=protalign&blast_rank=8&RID=0) 1 MREGRGGGREGRSAEPGPEARSHSVVPSRATHCCSFPEPFQQVCSRLAVKNHGLLLYALFSVILLGGASHASLIPETGKK 80

[XP_028707390.1](https://www.ncbi.nlm.nih.gov/protein/XP_028707390.1?report=genbank&log$=protalign&blast_rank=9&RID=0) 1 MREGRGGGREGRSAEPGPEARSHSVVPSRATHCCSFPEPFQQVCSRLAVKNHGLLLYALFSVILLGGASHASLIPETGKK 80

[XP_019688586.1](https://www.ncbi.nlm.nih.gov/protein/XP_019688586.1?report=genbank&log$=protalign&blast_rank=10&RID=0) 1 MHEGRGGGREGRRAEPCPEARSHSVVPSRATHCRSSSEPFQQVCSRLAVKNHGLLLYALFSVILLGGSSHASLIPETGKK 80

[NP_001272575.1](https://www.ncbi.nlm.nih.gov/protein/NP_001272575.1?report=genbank&log$=protalign&blast_rank=11&RID=0) 1 MIPG-----------------------------------------------NRMLMVVLLCQVLLGGASHASLIPETGKK 33

[XP_030857866.1](https://www.ncbi.nlm.nih.gov/protein/XP_030857866.1?report=genbank&log$=protalign&blast_rank=12&RID=0) 1 MREGRGGGREGRSAEPGPEARSHSVVPSRATHCCSFPEPFQQVCSRLAVKNHGLLLYALFSVILLGGASHASLIPETGKK 80

[XP_024087813.1](https://www.ncbi.nlm.nih.gov/protein/XP_024087813.1?report=genbank&log$=protalign&blast_rank=13&RID=0) 1 MREGRGGGREERSAEPGPEARSHSVVPPRATHCCSFPEPFQQVCSRLAVKNHGLLLYALFSVILLGGASHASLIPETGKK 80

[XP_032036116.1](https://www.ncbi.nlm.nih.gov/protein/XP_032036116.1?report=genbank&log$=protalign&blast_rank=14&RID=0) 1 MREGRGGGREGRSAEPGPEARSHSVVPSRATHCCSFPEPFQQVCSRLAVKNHGLLLYALFSVILLGGASHASLIPETGKK 80

[NP_001193.2](https://www.ncbi.nlm.nih.gov/protein/NP_001193.2?report=genbank&log$=protalign&blast_rank=0&RID=0) 34 KVAEIQGHAGGRRSGQSHELLRDFEATLLQMFGLRRRPQPSKSAVIPDYMRDLYRLQSGEEEEE-QIHSTGLEYPERPAS 112

[NP_031580.2](https://www.ncbi.nlm.nih.gov/protein/NP_031580.2?report=genbank&log$=protalign&blast_rank=1&RID=0) 34 KVAEIQGHAGGRRSGQSHELLRDFEATLLQMFGLRRRPQPSKSAVIPDYMRDLYRLQSGEEEEEEQSQGTGLEYPERPAS 113

[NP_036959.2](https://www.ncbi.nlm.nih.gov/protein/NP_036959.2?report=genbank&log$=protalign&blast_rank=2&RID=0) 34 KVAEIQGHAGGRRSGQSHELLRDFEATLLQMFGLRRRPQPSKSAVIPDYMRDLYRLQSGEEEEEEQSQGTGLEYPERPAS 113

[XP_024853077.1](https://www.ncbi.nlm.nih.gov/protein/XP_024853077.1?report=genbank&log$=protalign&blast_rank=3&RID=0) 81 KVAEIQGHAGGRRSGQSHELLRDFEATLLQMFGLRRRPQPSKSAVIPDYMRDLYRLQSGEEEEEEQIQGIGLEYPERPAS 160

[XP_020925510.1](https://www.ncbi.nlm.nih.gov/protein/XP_020925510.1?report=genbank&log$=protalign&blast_rank=4&RID=0) 81 KVAEIQGHAGGRRSGQSHELLRDFEATLLQMFGLRRRPQPSKSAVIPDYMRDLYRLQSGEEEEEEQTHSVGLEYPERPAS 160

[XP_038528824.1](https://www.ncbi.nlm.nih.gov/protein/XP_038528824.1?report=genbank&log$=protalign&blast_rank=5&RID=0) 81 KVAEIQGHAGGRRSGQSHELLRDFEATLLQMFGLRRRPQPSKSAVIPDYMRDLYRLQSGEEEEEEQIHSIGLEYPERPAS 160

[XP_023483474.1](https://www.ncbi.nlm.nih.gov/protein/XP_023483474.1?report=genbank&log$=protalign&blast_rank=6&RID=0) 81 KVAEIQGHAGGRRSGQSHELLRDFEATLLQMFGLRRRPQPSKNAVVPDYMRDLYRLQSGEEEEEEQIHSVGLEYPERPAS 160

[NP_001103747.1](https://www.ncbi.nlm.nih.gov/protein/NP_001103747.1?report=genbank&log$=protalign&blast_rank=7&RID=0) 34 KVAEIQGHAGGRRSGQSHELLRDFEATLLQMFGLRRRPQPSKSAVIPDYMRDLYRLQSGEEEEEEQIQGIGLEYPERPAS 113

[XP_024204496.1](https://www.ncbi.nlm.nih.gov/protein/XP_024204496.1?report=genbank&log$=protalign&blast_rank=8&RID=0) 81 KVAEIQGHAGGRRSGQSHELLRDFEATLLQMFGLRRRPQPSKSAVIPDYMRDLYRLQSGEEEEE-QIHSTGLEYPERPAS 159

[XP_028707390.1](https://www.ncbi.nlm.nih.gov/protein/XP_028707390.1?report=genbank&log$=protalign&blast_rank=9&RID=0) 81 KVAEIQGHAGGRRSGQSHELLRDFEATLLQMFGLRRRPQPSKSAVIPDYMRDLYRLQSGEEEEE-QIHSAGLEYPERPAS 159

[XP_019688586.1](https://www.ncbi.nlm.nih.gov/protein/XP_019688586.1?report=genbank&log$=protalign&blast_rank=10&RID=0) 81 KVAEIQGHAGGRRSGQSHELLRDFEATLLQMFGLRRRPQPSKSAVIPDYMRDLYRLQSGEEEEEEQTHSVGLEYPERPAS 160

[NP_001272575.1](https://www.ncbi.nlm.nih.gov/protein/NP_001272575.1?report=genbank&log$=protalign&blast_rank=11&RID=0) 34 KVAEIQGHAGGRRSGQSHELLRDFEATLLQMFGLRRRPQPSKSAVIPDYMRDLYRLQSGEEEEEEQIQGIGLEYPERPAS 113

[XP_030857866.1](https://www.ncbi.nlm.nih.gov/protein/XP_030857866.1?report=genbank&log$=protalign&blast_rank=12&RID=0) 81 KVAEIQGHAGGRRSGQSHELLRDFEATLLQMFGLRRRPQPSKSAVIPDYMRDLYRLQSGEEEEE-QIHSTGLEYPERPAS 159

[XP_024087813.1](https://www.ncbi.nlm.nih.gov/protein/XP_024087813.1?report=genbank&log$=protalign&blast_rank=13&RID=0) 81 KVAEIQGHAGGRRSGQSHELLRDFEATLLQMFGLRRRPQPSKSAVIPDYMRDLYRLQSGEEEEE-QIHSTGLEYPERPAS 159

[XP_032036116.1](https://www.ncbi.nlm.nih.gov/protein/XP_032036116.1?report=genbank&log$=protalign&blast_rank=14&RID=0) 81 KVAEIQGHAGGRRSGQSHELLRDFEATLLQMFGLRRRPQPSKSAVIPDYMRDLYRLQSGEEEEE-QIHSTGLEYPERPAS 159

[NP_001193.2](https://www.ncbi.nlm.nih.gov/protein/NP_001193.2?report=genbank&log$=protalign&blast_rank=0&RID=0) 113 RANTVRSFHHEEHLENIPGTSENSAFRFLFNLSSIPENEVISSAELRLFREQVDQGPDWERGFHRINIYEVMKPPAEVVP 192

[NP_031580.2](https://www.ncbi.nlm.nih.gov/protein/NP_031580.2?report=genbank&log$=protalign&blast_rank=1&RID=0) 114 RANTVRSFHHEEHLENIPGTSESSAFRFLFNLSSIPENEVISSAELRLFREQVDQGPDWEQGFHRINIYEVMKPPAEMVP 193

[NP_036959.2](https://www.ncbi.nlm.nih.gov/protein/NP_036959.2?report=genbank&log$=protalign&blast_rank=2&RID=0) 114 RANTVRSFHHEEHLENIPGTSESSAFRFFFNLSSIPENEVISSAELRLFREQVDQGPDWEQGFHRINIYEVMKPPAEMVP 193

[XP_024853077.1](https://www.ncbi.nlm.nih.gov/protein/XP_024853077.1?report=genbank&log$=protalign&blast_rank=3&RID=0) 161 RANTVRSFHHEEHLENIPGTSENSAFRFLFNLSSIPENEVISSAELRLFREQVDQGPDWDQGFHRINIYEVMKPPAEVVP 240

[XP_020925510.1](https://www.ncbi.nlm.nih.gov/protein/XP_020925510.1?report=genbank&log$=protalign&blast_rank=4&RID=0) 161 RANTVRSFHHEEHLENIPGTSENSAFRFLFNLSSIPENEVISSAELRLFREQVDQGPDWEQGFHRINIYEVMKPPPEVVP 240

[XP_038528824.1](https://www.ncbi.nlm.nih.gov/protein/XP_038528824.1?report=genbank&log$=protalign&blast_rank=5&RID=0) 161 RANTVRSFHHEEHLENIPGTSENSAFRFLFNLSSIPENEVISSAELRLFREQVNQDPDWEQGFHRINIYEVMKPPAEVVP 240

[XP_023483474.1](https://www.ncbi.nlm.nih.gov/protein/XP_023483474.1?report=genbank&log$=protalign&blast_rank=6&RID=0) 161 RANTVRSFHHEEHLESIPGTSENSAFRFLFNLSSIPENEVISSAELRLFREQVDQGPDWEQGFHRINVYEVMKPPAEVVP 240

[NP_001103747.1](https://www.ncbi.nlm.nih.gov/protein/NP_001103747.1?report=genbank&log$=protalign&blast_rank=7&RID=0) 114 RANTVRSFHHEEHLENIPGTSENSAFRFLFNLSSIPENEVISSAELRLFREQVDQGPDWEQGFHRINIYEVMKPPAEVVP 193

[XP_024204496.1](https://www.ncbi.nlm.nih.gov/protein/XP_024204496.1?report=genbank&log$=protalign&blast_rank=8&RID=0) 160 RANTVRSFHHEEHLENIPGTSENSAFRFLFNLSSIPENEVISSAELRLFREQVDQGPDWERGFHRINIYEVMKPPAEVVP 239

[XP_028707390.1](https://www.ncbi.nlm.nih.gov/protein/XP_028707390.1?report=genbank&log$=protalign&blast_rank=9&RID=0) 160 RANTVRSFHHEEHLENIPGTSENSAFRFLFNLSSIPENEVISSAELRLFREQVDQGPDWERGFHRINIYEVMKPPAEVVP 239

[XP_019688586.1](https://www.ncbi.nlm.nih.gov/protein/XP_019688586.1?report=genbank&log$=protalign&blast_rank=10&RID=0) 161 RANTVRSFHHEEHLENIPGTSENSAFRFLFNLSSIPENEVVSSAELRLFREQVNQGPDWEQGFHRINIYEVMKPPAEVVP 240

[NP_001272575.1](https://www.ncbi.nlm.nih.gov/protein/NP_001272575.1?report=genbank&log$=protalign&blast_rank=11&RID=0) 114 RANTVRSFHHEEHLENIPGTSENSAFRFLFNLSSIPENEVISSAELRLFREQVDQGPDWEQGFHRINIYEVMKPPAEVVP 193

[XP_030857866.1](https://www.ncbi.nlm.nih.gov/protein/XP_030857866.1?report=genbank&log$=protalign&blast_rank=12&RID=0) 160 RANTVRSFHHEEHLENIPGTSENSAFRFLFNLSSIPENEVISSAELRLFREQVDQGPDWERGFHRINIYEVMKPPAEVVP 239

[XP_024087813.1](https://www.ncbi.nlm.nih.gov/protein/XP_024087813.1?report=genbank&log$=protalign&blast_rank=13&RID=0) 160 RANTVRSFHHEEHLENIPGTSENSAFRFLFNLSSIPENEVISSAELRLFREQVDQGPDWERGFHRINIYEVMKPPAEVVP 239

[XP_032036116.1](https://www.ncbi.nlm.nih.gov/protein/XP_032036116.1?report=genbank&log$=protalign&blast_rank=14&RID=0) 160 RANTVRSFHHEEHLENIPGTSENSAFRFLFNLSSIPENEVISSAELRLFREQVDQGPDWEQGFHRINIYEVMKPPAEVVP 239

[NP_001193.2](https://www.ncbi.nlm.nih.gov/protein/NP_001193.2?report=genbank&log$=protalign&blast_rank=0&RID=0) 193 GHLITRLLDTRLVHHNVTRWETFDVSPAVLRWTREKQPNYGLAIEVTHLHQTRTHQGQHVRISRSLPQGSGNWAQLRPLL 272

[NP_031580.2](https://www.ncbi.nlm.nih.gov/protein/NP_031580.2?report=genbank&log$=protalign&blast_rank=1&RID=0) 194 GHLITRLLDTRLVHHNVTRWETFDVSPAVLRWTREKQPNYGLAIEVTHLHQTRTHQGQHVRISRSLPQGSGDWAQLRPLL 273

[NP_036959.2](https://www.ncbi.nlm.nih.gov/protein/NP_036959.2?report=genbank&log$=protalign&blast_rank=2&RID=0) 194 GHLITRLLDTRLVHHNVTRWETFDVSPAVLRWTREKQPNYGLAIEVTHLHQTRTHQGQHVRISRSLPQGSGNWAQLRPLL 273

[XP_024853077.1](https://www.ncbi.nlm.nih.gov/protein/XP_024853077.1?report=genbank&log$=protalign&blast_rank=3&RID=0) 241 GHLITRLLDTRLVHHNVTRWETFDVSPAVLRWTREKQPNYGLAIEVTHLHQTRTHQGQHVRISRSLPQGSGDWAQLRPLL 320

[XP_020925510.1](https://www.ncbi.nlm.nih.gov/protein/XP_020925510.1?report=genbank&log$=protalign&blast_rank=4&RID=0) 241 GHLITRLLDTRLVHHNVTRWETFDVSPAVLRWTREKQPNYGLAIEVTHLHQTRTHQGQHVRISRSLPQGSGDWAQLRPLL 320

[XP_038528824.1](https://www.ncbi.nlm.nih.gov/protein/XP_038528824.1?report=genbank&log$=protalign&blast_rank=5&RID=0) 241 GHLITRLLDTRLVHHNVTRWETFDVSPAVLRWTREKQPNYGLAIEVTHLHQTRTHQGQHVRISRSLPQGSGDWAQLRPLL 320

[XP_023483474.1](https://www.ncbi.nlm.nih.gov/protein/XP_023483474.1?report=genbank&log$=protalign&blast_rank=6&RID=0) 241 GRLITRLLDTRLVHHSVTRWETFDVSPAVLRWTREKQPNYGLAIEVTPLHQTRTHQGQHVRISRALPQGSGDWAQLRPLL 320

[NP_001103747.1](https://www.ncbi.nlm.nih.gov/protein/NP_001103747.1?report=genbank&log$=protalign&blast_rank=7&RID=0) 194 GHLITRLLDTRLVHHNVTRWETFDVSPAVLRWTREKQPNYGLAIEVTHLHQTRTHQGQHVRISRSLPQGSGDWAQLRPLL 273

[XP_024204496.1](https://www.ncbi.nlm.nih.gov/protein/XP_024204496.1?report=genbank&log$=protalign&blast_rank=8&RID=0) 240 GHLITRLLDTRLVHHNVTRWETFDVSPAVLRWTREKQPNYGLAIEVTHLHQTRTHQGQHVRISRSLPQGSGNWAQLRPLL 319

[XP_028707390.1](https://www.ncbi.nlm.nih.gov/protein/XP_028707390.1?report=genbank&log$=protalign&blast_rank=9&RID=0) 240 GHLITRLLDTRLVHHNVTRWETFDVSPAVLRWTREKQPNYGLAIEVTHLHQTRTHQGQHVRISRSLPQGSGNWAQLRPLL 319

[XP_019688586.1](https://www.ncbi.nlm.nih.gov/protein/XP_019688586.1?report=genbank&log$=protalign&blast_rank=10&RID=0) 241 GHLITRLLDTRLVHHNVTRWETFDVSPAVLRWTREKQPNYGLAIEVTHLHQTRTHQGQHVRISRSLPQGSGDWAQLRPLL 320

[NP_001272575.1](https://www.ncbi.nlm.nih.gov/protein/NP_001272575.1?report=genbank&log$=protalign&blast_rank=11&RID=0) 194 GHLITRLLDTRLVHHNVTRWETFDVSPAVLRWTREKQPNYGLAIEVTHLHQTRTHQDQHVRISRSLPQGSGDWAQLRPLL 273

[XP_030857866.1](https://www.ncbi.nlm.nih.gov/protein/XP_030857866.1?report=genbank&log$=protalign&blast_rank=12&RID=0) 240 GHLITRLLDTRLVHHNVTRWETFDVSPAVLRWTREKQPNYGLAIEVTHLHQTRTHQGQHVRISRSLPQGSGNWAQLRPLL 319

[XP_024087813.1](https://www.ncbi.nlm.nih.gov/protein/XP_024087813.1?report=genbank&log$=protalign&blast_rank=13&RID=0) 240 GHLITRLLDTRLIHHNVTRWETFDVSPAVLRWTREKQPNYGLAIEVTHLHQTRTHQGQHVRISRSLPQGSGNWAQLRPLL 319

[XP_032036116.1](https://www.ncbi.nlm.nih.gov/protein/XP_032036116.1?report=genbank&log$=protalign&blast_rank=14&RID=0) 240 GHLITRLLDTRLVHHNVTRWETFDVSPAVLRWTGEKQPNYGLAIEVTHLHQTRTHQGQHVRISRSLPQGSGNWAQLRPLL 319

[NP_001193.2](https://www.ncbi.nlm.nih.gov/protein/NP_001193.2?report=genbank&log$=protalign&blast_rank=0&RID=0) 273 VTFGHDGRGHALTRRRRAKRSPKHHSQRARKKNKNCRRHSLYVDFSDVGWNDWIVAPPGYQAFYCHGDCPFPLADHLNST 352

[NP_031580.2](https://www.ncbi.nlm.nih.gov/protein/NP_031580.2?report=genbank&log$=protalign&blast_rank=1&RID=0) 274 VTFGHDGRGHTLTRRR-AKRSPKHHPQRSRKKNKNCRRHSLYVDFSDVGWNDWIVAPPGYQAFYCHGDCPFPLADHLNST 352

[NP_036959.2](https://www.ncbi.nlm.nih.gov/protein/NP_036959.2?report=genbank&log$=protalign&blast_rank=2&RID=0) 274 VTFGHDGRGHTLTRRR-AKRSPKHHPQRSRKKNKNCRRHSLYVDFSDVGWNDWIVAPPGYQAFYCHGDCPFPLADHLNST 352

[XP_024853077.1](https://www.ncbi.nlm.nih.gov/protein/XP_024853077.1?report=genbank&log$=protalign&blast_rank=3&RID=0) 321 VTFGHDGRGHALTRRRRAKRSPKHHPQRARKKNKNCRRHSLYVDFSDVGWNDWIVAPPGYQAFYCHGDCPFPLADHLNST 400

[XP_020925510.1](https://www.ncbi.nlm.nih.gov/protein/XP_020925510.1?report=genbank&log$=protalign&blast_rank=4&RID=0) 321 VTFGHDGRGHALTRRRRAKRSPKHHPQRARKKNKNCRRHSLYVDFSDVGWNDWIVAPPGYQAFYCHGDCPFPLADHLNST 400

[XP_038528824.1](https://www.ncbi.nlm.nih.gov/protein/XP_038528824.1?report=genbank&log$=protalign&blast_rank=5&RID=0) 321 VTFGHDGRGHALTRRQRAKRSPKHHAQRARKKNKNCRRHSLYVDFSDVGWNDWIVAPPGYQAFYCHGDCPFPLADHLNST 400

[XP_023483474.1](https://www.ncbi.nlm.nih.gov/protein/XP_023483474.1?report=genbank&log$=protalign&blast_rank=6&RID=0) 321 VTFGHDGRGHALTRRRRAKRSPKHHPQRARKKTKNCRRHSLYVDFSDVGWNDWIVAPPGYQAFYCHGDCPFPLADHLNST 400

[NP_001103747.1](https://www.ncbi.nlm.nih.gov/protein/NP_001103747.1?report=genbank&log$=protalign&blast_rank=7&RID=0) 274 VTFGHDGRGHALTRRRRAKRSPKHHPQRARKKNKNCRRHSLYVDFSDVGWNDWIVAPPGYQAFYCHGDCPFPLADHLNST 353

[XP_024204496.1](https://www.ncbi.nlm.nih.gov/protein/XP_024204496.1?report=genbank&log$=protalign&blast_rank=8&RID=0) 320 VTFGHDGRGHALTRRRRAKRSPKHHPQRARKKNKNCRRHSLYVDFSDVGWNDWIVAPPGYQAFYCHGDCPFPLADHLNST 399

[XP_028707390.1](https://www.ncbi.nlm.nih.gov/protein/XP_028707390.1?report=genbank&log$=protalign&blast_rank=9&RID=0) 320 VTFGHDGRGHALTRRRRAKRSPKHHPQRARKKNKNCRRHSLYVDFSDVGWNDWIVAPPGYQAFYCHGDCPFPLADHLNST 399

[XP_019688586.1](https://www.ncbi.nlm.nih.gov/protein/XP_019688586.1?report=genbank&log$=protalign&blast_rank=10&RID=0) 321 VTFGHDGRGHALTRRQRAKRSPKHHPQRARKKNKNCRRHSLYVDFSDVGWNDWIVAPPGYQAFYCHGDCPFPLADHLNST 400

[NP_001272575.1](https://www.ncbi.nlm.nih.gov/protein/NP_001272575.1?report=genbank&log$=protalign&blast_rank=11&RID=0) 274 VTFGHDGRGHALTRRRRAKRSPKHHPQRARKKNKNCRRHSLYVDFSDVGWNDWIVAPPGYQAFYCHGDCPFPLADHLNST 353

[XP_030857866.1](https://www.ncbi.nlm.nih.gov/protein/XP_030857866.1?report=genbank&log$=protalign&blast_rank=12&RID=0) 320 VTFGHDGRGHALTRRRRAKRSPKHHPQRARKKNKNCRRHSLYVDFSDVGWNDWIVAPPGYQAFYCHGDCPFPLADHLNST 399

[XP_024087813.1](https://www.ncbi.nlm.nih.gov/protein/XP_024087813.1?report=genbank&log$=protalign&blast_rank=13&RID=0) 320 VTFGHDGRGHALTRRRRAKRSPKHHPQRARKKNKNCRRHSLYVDFSDVGWNDWIVAPPGYQAFYCHGDCPFPLADHLNST 399

[XP_032036116.1](https://www.ncbi.nlm.nih.gov/protein/XP_032036116.1?report=genbank&log$=protalign&blast_rank=14&RID=0) 320 VTFGHDGRGHALTRRRRAKRSPKHHPQRARKKNKNCRRHSLYVDFSDVGWNDWIVAPPGYQAFYCHGDCPFPLADHLNST 399

[NP_001193.2](https://www.ncbi.nlm.nih.gov/protein/NP_001193.2?report=genbank&log$=protalign&blast_rank=0&RID=0) 353 NHAIVQTLVNSVNSSIPKACCVPTELSAISMLYLDEYDKVVLKNYQEMVVEGCGCR 408

[NP_031580.2](https://www.ncbi.nlm.nih.gov/protein/NP_031580.2?report=genbank&log$=protalign&blast_rank=1&RID=0) 353 NHAIVQTLVNSVNSSIPKACCVPTELSAISMLYLDEYDKVVLKNYQEMVVEGCGCR 408

[NP_036959.2](https://www.ncbi.nlm.nih.gov/protein/NP_036959.2?report=genbank&log$=protalign&blast_rank=2&RID=0) 353 NHAIVQTLVNSVNSSIPKACCVPTELSAISMLYLDEYDKVVLKNYQEMVVEGCGCR 408

[XP_024853077.1](https://www.ncbi.nlm.nih.gov/protein/XP_024853077.1?report=genbank&log$=protalign&blast_rank=3&RID=0) 401 NHAIVQTLVNSVNSSIPKACCVPTELSAISMLYLDEYDKVVLKNYQEMVVEGCGCR 456

[XP_020925510.1](https://www.ncbi.nlm.nih.gov/protein/XP_020925510.1?report=genbank&log$=protalign&blast_rank=4&RID=0) 401 NHAIVQTLVNSVNSSIPKACCVPTELSAISMLYLDEYDKVVLKNYQEMVVEGCGCR 456

[XP_038528824.1](https://www.ncbi.nlm.nih.gov/protein/XP_038528824.1?report=genbank&log$=protalign&blast_rank=5&RID=0) 401 NHAIVQTLVNSVNSSIPKACCVPTELSAISMLYLDEYDKVVLKNYQEMVVEGCGCR 456

[XP_023483474.1](https://www.ncbi.nlm.nih.gov/protein/XP_023483474.1?report=genbank&log$=protalign&blast_rank=6&RID=0) 401 NHAIVQTLVNSVNSSIPKACCVPTELSAISMLYLDEYDKVVLKNYQEMVVEGCGCR 456

[NP_001103747.1](https://www.ncbi.nlm.nih.gov/protein/NP_001103747.1?report=genbank&log$=protalign&blast_rank=7&RID=0) 354 NHAIVQTLVNSVNSSIPKACCVPTELSAISMLYLDEYDKVALKNYQEMVVEGCGCR 409

[XP_024204496.1](https://www.ncbi.nlm.nih.gov/protein/XP_024204496.1?report=genbank&log$=protalign&blast_rank=8&RID=0) 400 NHAIVQTLVNSVNSSIPKACCVPTELSAISMLYLDEYDKVVLKNYQEMVVEGCGCR 455

[XP_028707390.1](https://www.ncbi.nlm.nih.gov/protein/XP_028707390.1?report=genbank&log$=protalign&blast_rank=9&RID=0) 400 NHAIVQTLVNSVNSSIPKACCVPTELSAISMLYLDEYDKVVLKNYQEMVVEGCGCR 455

[XP_019688586.1](https://www.ncbi.nlm.nih.gov/protein/XP_019688586.1?report=genbank&log$=protalign&blast_rank=10&RID=0) 401 NHAIVQTLVNSVNSSIPKACCVPTELSAISMLYLDEYDKVVLKNYQEMVVEGCGCR 456

[NP_001272575.1](https://www.ncbi.nlm.nih.gov/protein/NP_001272575.1?report=genbank&log$=protalign&blast_rank=11&RID=0) 354 NHAIVQTLVNSVNSSIPKACCVPTELSAISMLYLDEYDKVVLKNYQEMVVEGCGCR 409

[XP_030857866.1](https://www.ncbi.nlm.nih.gov/protein/XP_030857866.1?report=genbank&log$=protalign&blast_rank=12&RID=0) 400 NHAIVQTLVNSVNSSIPKACCVPTELSAISMLYLDEYDKVVLKNYQEMVVEGCGCR 455

[XP_024087813.1](https://www.ncbi.nlm.nih.gov/protein/XP_024087813.1?report=genbank&log$=protalign&blast_rank=13&RID=0) 400 NHAIVQTLVNSVNSSIPKACCVPTELSAISMLYLDEYDKVVLKNYQEMVVEGCGCR 455

[XP_032036116.1](https://www.ncbi.nlm.nih.gov/protein/XP_032036116.1?report=genbank&log$=protalign&blast_rank=14&RID=0) 400 NHAIVQTLVNSVNSSIPKACCVPTELSAISMLYLDEYDKVVLKNYQEMVVEGCGCR 455

BMP7

NP_001710.1 bone morphogenetic protein 7 preproprotein [Homo sapiens]

NP_031583.2 bone morphogenetic protein 7 preproprotein [Mus musculus]

NP_001178785.1 bone morphogenetic protein 7 precursor [Rattus norvegicus]

NP_001192944.1 bone morphogenetic protein 7 precursor [Bos taurus]

XP_005673101.1 bone morphogenetic protein 7 isoform X1 [Sus scrofa]

NP_001183981.1 bone morphogenetic protein 7 precursor [Canis lupus familiaris]

NP_001182087.1 bone morphogenetic protein 7 precursor [Equus caballus]

NP_001295493.1 bone morphogenetic protein 7 precursor [Ovis aries]

XP_001170064.1 bone morphogenetic protein 7 [Pan troglodytes]

XP_001089245.1 bone morphogenetic protein 7 [Macaca mulatta]

XP_011279062.2 LOW QUALITY PROTEIN: bone morphogenetic protein 7 [Felis catus]

XP_017913119.1 PREDICTED: bone morphogenetic protein 7 [Capra hircus]

XP_030860862.1 bone morphogenetic protein 7 [Gorilla gorilla gorilla]

XP_024094889.1 bone morphogenetic protein 7 [Pongo abelii]

XP_031998385.1 bone morphogenetic protein 7 [Hylobates moloch]

[NP_001710.1](https://www.ncbi.nlm.nih.gov/protein/NP_001710.1?report=genbank&log$=protalign&blast_rank=0&RID=0) 1 MHVRSLRAAAPHSFVALWAPLFLLRSALADFSLDNEVHSSFIHRRLRSQERREMQREILSILGLPHRPRPHLQGKHNSAP 80

[NP_031583.2](https://www.ncbi.nlm.nih.gov/protein/NP_031583.2?report=genbank&log$=protalign&blast_rank=1&RID=0) 1 MHVRSLRAAAPHSFVALWAPLFLLRSALADFSLDNEVHSSFIHRRLRSQERREMQREILSILGLPHRPRPHLQGKHNSAP 80

[NP_001178785.1](https://www.ncbi.nlm.nih.gov/protein/NP_001178785.1?report=genbank&log$=protalign&blast_rank=2&RID=0) 1 MHVRSLRAAAPHSFVALWAPLFLLRSALADFSLDNEVHSSFIHRRLRSQERREMQREILSILGLPHRPRPHLQGKHNSAP 80

[NP_001192944.1](https://www.ncbi.nlm.nih.gov/protein/NP_001192944.1?report=genbank&log$=protalign&blast_rank=3&RID=0) 1 MHMRSLRAAAPHSFVALWAPLFLLRSALADFSLDNEVHSSFIHRRLRSQERREMQREILSILGLPHRPRPHLQGKHNSAP 80

[XP_005673101.1](https://www.ncbi.nlm.nih.gov/protein/XP_005673101.1?report=genbank&log$=protalign&blast_rank=4&RID=0) 1 MHVRSLRAAAPHSFVALWAPLFLLRSALADFSLDNEVHSSFIHRRLRSQERREMQREILSILGLPHRPRPHLQGKHNSAP 80

[NP_001183981.1](https://www.ncbi.nlm.nih.gov/protein/NP_001183981.1?report=genbank&log$=protalign&blast_rank=5&RID=0) 1 MHVRSPCAAAPRSFVALWAPLLLLRSALADFSLDNEVHSSFIHRRLRSQERREMQREILSILGLPHRPRPHLQGKHNSAP 80

[NP_001182087.1](https://www.ncbi.nlm.nih.gov/protein/NP_001182087.1?report=genbank&log$=protalign&blast_rank=6&RID=0) 1 MHVRSLRTAAPHSFVALWAPLFLLRSALADFSLDNEVHSSFIHRRLRSQERREMQREILSILGLPHRPRPHLQGKHNSAP 80

[NP_001295493.1](https://www.ncbi.nlm.nih.gov/protein/NP_001295493.1?report=genbank&log$=protalign&blast_rank=7&RID=0) 1 MHMRSLRAAAPHSFVALWAPLFLLRSALADFSLDNEVHSSFIHRRLRSQERREMQREILSILGLPHRPRPHLQGKHNSAP 80

[XP_001170064.1](https://www.ncbi.nlm.nih.gov/protein/XP_001170064.1?report=genbank&log$=protalign&blast_rank=8&RID=0) 1 MHVRSLRAAAPHSFVALWAPLFLLRSALADFSLDNEVHSSFIHRRLRSQERREMQREILSILGLPHRPRPHLQGKHNSAP 80

[XP_001089245.1](https://www.ncbi.nlm.nih.gov/protein/XP_001089245.1?report=genbank&log$=protalign&blast_rank=9&RID=0) 1 MHVRSLRAAAPHSFVALWAPLFLLRSALADFSLDNEVHSSFIHRRLRSQERREMQREILSILGLPHRPRPHLQGKHNSAP 80

[XP_011279062.2](https://www.ncbi.nlm.nih.gov/protein/XP_011279062.2?report=genbank&log$=protalign&blast_rank=10&RID=0) 1 MHVRSLRAAAPHSFVALWAPLFLLRSALADFSLDNEVHSSFIHRRLRSQERREMQREILSILGLPHRPRPHLQGKHNSAP 80

[XP_017913119.1](https://www.ncbi.nlm.nih.gov/protein/XP_017913119.1?report=genbank&log$=protalign&blast_rank=11&RID=0) 1 MHMRSLRAAAPHSFVALWAPLFLLRSALADFSLDNEVHSSFIHRRLRSQERREMQREILSILGLPHRPRPHLQGKHNSAP 80

[XP_030860862.1](https://www.ncbi.nlm.nih.gov/protein/XP_030860862.1?report=genbank&log$=protalign&blast_rank=12&RID=0) 1 MHVRSLRAAAPHSFVALWAPLFLLRSALADFSLDNEVHSSFIHRRLRSQERREMQREILSILGLPHRPRPHLQGKHNSAP 80

[XP_024094889.1](https://www.ncbi.nlm.nih.gov/protein/XP_024094889.1?report=genbank&log$=protalign&blast_rank=13&RID=0) 1 MHVRSLRAAAPHSFVALWAPLFLLRSALADFSLDNEVHSSFIHRRLRSQERREMQREILSILGLPHRPRPHLQGKHNSAP 80

[XP_031998385.1](https://www.ncbi.nlm.nih.gov/protein/XP_031998385.1?report=genbank&log$=protalign&blast_rank=14&RID=0) 1 MHVRSLRAAAPHSFVALWAPLFLLRSALADFSLDNEVHSSFIHRRLRSQERREMQREILSILGLPHRPRPHLQGKHNSAP 80

[NP_001710.1](https://www.ncbi.nlm.nih.gov/protein/NP_001710.1?report=genbank&log$=protalign&blast_rank=0&RID=0) 81 MFMLDLYNAMAVEEGGGPGGQGFSYPYKAVFSTQGPPLASLQDSHFLTDADMVMSFVNLVEHDKEFFHPRYHHREFRFDL 160

[NP_031583.2](https://www.ncbi.nlm.nih.gov/protein/NP_031583.2?report=genbank&log$=protalign&blast_rank=1&RID=0) 81 MFMLDLYNAMAVEESG-PDGQGFSYPYKAVFSTQGPPLASLQDSHFLTDADMVMSFVNLVEHDKEFFHPRYHHREFRFDL 159

[NP_001178785.1](https://www.ncbi.nlm.nih.gov/protein/NP_001178785.1?report=genbank&log$=protalign&blast_rank=2&RID=0) 81 MFMLDLYNAMAVEESG-PDGQGFSYPYKAVFSTQGPPLASLQDSHFLTDADMVMSFVNLVEHDKEFFHPRYHHREFRFDL 159

[NP_001192944.1](https://www.ncbi.nlm.nih.gov/protein/NP_001192944.1?report=genbank&log$=protalign&blast_rank=3&RID=0) 81 MFMLDLYNAMAVEEGGGPDGQGFSYPYKAVFSTQGPPLASLQDSHFLTDADMVMSFVNLVEHDKEFFHPRYHHREFRFDL 160

[XP_005673101.1](https://www.ncbi.nlm.nih.gov/protein/XP_005673101.1?report=genbank&log$=protalign&blast_rank=4&RID=0) 81 MFMLDLYNAMAVEEGGGPDGQGFSYPYKAVFSTQGPPLASLQDSHFLTDADMVMSFVNLVEHDKEFFHPRYHHREFRFDL 160

[NP_001183981.1](https://www.ncbi.nlm.nih.gov/protein/NP_001183981.1?report=genbank&log$=protalign&blast_rank=5&RID=0) 81 MFMLDLYNAMAVEEGGGPDGQGFSYPYKAVFSTQGPPLASLQDSHFLTDADMVMSFVNLVEHDKEFFYPRYHHREFRFDL 160

[NP_001182087.1](https://www.ncbi.nlm.nih.gov/protein/NP_001182087.1?report=genbank&log$=protalign&blast_rank=6&RID=0) 81 MFMLDLYNAMAVEESGGPDGQGFSYPHKAVSSTQGPPLASLQDSHFLTDADMVMSFVNLVEHDKEFFHPRYHHREFRFDL 160

[NP_001295493.1](https://www.ncbi.nlm.nih.gov/protein/NP_001295493.1?report=genbank&log$=protalign&blast_rank=7&RID=0) 81 MFMLDLYNAMAVEEGGGPDGQGFSYPYKAVFSTQGPPLASLRDSHFLTDADMVMSFVNLVEHDKEFFHPRYHHREFRFDL 160

[XP_001170064.1](https://www.ncbi.nlm.nih.gov/protein/XP_001170064.1?report=genbank&log$=protalign&blast_rank=8&RID=0) 81 MFMLDLYNAMAVEEGGGPGGQGFSYPYKAVFSTQGPPLASLQDSHFLTDADMVMSFVNLVEHDKEFFHPRYHHREFRFDL 160

[XP_001089245.1](https://www.ncbi.nlm.nih.gov/protein/XP_001089245.1?report=genbank&log$=protalign&blast_rank=9&RID=0) 81 MFMLDLYNAMAVEEGGGPGGQGFSYPYKAVFSTQGPPLASLQDSHFLTDADMVMSFVNLVEHDKEFFHPRYHHREFRFDL 160

[XP_011279062.2](https://www.ncbi.nlm.nih.gov/protein/XP_011279062.2?report=genbank&log$=protalign&blast_rank=10&RID=0) 81 MFMLDLYNAMAVEEGGGPDGQGFSYPYKAVFSTQGPPLASLQDSRFLTDADMVMSFVNLVEHDKEFFHPRYHHREFRFDL 160

[XP_017913119.1](https://www.ncbi.nlm.nih.gov/protein/XP_017913119.1?report=genbank&log$=protalign&blast_rank=11&RID=0) 81 MFMLDLYNAMAVEEGGGPDGQGFSYPYKAVFSTQGPPLASLQDSHFLTDADMVMSFVNLVEHDKEFFHPRYHHREFRFDL 160

[XP_030860862.1](https://www.ncbi.nlm.nih.gov/protein/XP_030860862.1?report=genbank&log$=protalign&blast_rank=12&RID=0) 81 MFMLDLYNAMAVEEGGGPGGQGFSYPYKAVFSTQGPPLASLQDSHFLTDADMVMSFVNLVEHDKEFFHPRYHHREFRFDL 160

[XP_024094889.1](https://www.ncbi.nlm.nih.gov/protein/XP_024094889.1?report=genbank&log$=protalign&blast_rank=13&RID=0) 81 MFMLDLYNAMAVEEGGGPGGQGFSYPYKAVFSTQGPPLASLQDSHFLTDADMVMSFVNLVEHDKEFFHPRYHHREFRFDL 160

[XP_031998385.1](https://www.ncbi.nlm.nih.gov/protein/XP_031998385.1?report=genbank&log$=protalign&blast_rank=14&RID=0) 81 MFMLDLYNAMAVEEGGGPGGQGFSYPYKAVFSTQGPPLASLQDSHFLTDADMVMSFVNLVEHDKEFFHPRYHHREFRFDL 160

[NP_001710.1](https://www.ncbi.nlm.nih.gov/protein/NP_001710.1?report=genbank&log$=protalign&blast_rank=0&RID=0) 161 SKIPEGEAVTAAEFRIYKDYIRERFDNETFRISVYQVLQEHLGRESD-LFLLDSRTLWASEEGWLVFDITATSNHWVVNP 239

[NP_031583.2](https://www.ncbi.nlm.nih.gov/protein/NP_031583.2?report=genbank&log$=protalign&blast_rank=1&RID=0) 160 SKIPEGEAVTAAEFRIYKDYIRERFDNETFQITVYQVLQEHSGRESD-LFLLDSRTIWASEEGWLVFDITATSNHWVVNP 238

[NP_001178785.1](https://www.ncbi.nlm.nih.gov/protein/NP_001178785.1?report=genbank&log$=protalign&blast_rank=2&RID=0) 160 SKIPEGEAVTAAEFRIYKDYIRERFDNETFQITVYQVLQEHSGRESD-LFLLDSRTIWASEEGWLVFDITATSNHWVVNP 238

[NP_001192944.1](https://www.ncbi.nlm.nih.gov/protein/NP_001192944.1?report=genbank&log$=protalign&blast_rank=3&RID=0) 161 SKIPEGEAVTAAEFRIYKDYIREHFHNETFRISVYQVLQEHLGRESD-LFLLDSRTLWASEEGWLVFDITATSNHWVVNP 239

[XP_005673101.1](https://www.ncbi.nlm.nih.gov/protein/XP_005673101.1?report=genbank&log$=protalign&blast_rank=4&RID=0) 161 SKIPEGEAVTAAEFRIYKDYIREHFNNETFRISVYQVLQEHLGRDSD-LFLLDSRTLWASEEGWLVFDITATSNHWVVNP 239

[NP_001183981.1](https://www.ncbi.nlm.nih.gov/protein/NP_001183981.1?report=genbank&log$=protalign&blast_rank=5&RID=0) 161 SKIPEGEAVTAAEFRIYKDYIRERFDNETFRISVYQVLQEPQDSSLDyLFLLDSRTLWASEEGWLVFDITATSNHWVVNP 240

[NP_001182087.1](https://www.ncbi.nlm.nih.gov/protein/NP_001182087.1?report=genbank&log$=protalign&blast_rank=6&RID=0) 161 SKIPEGEAVTAAEFRIYKDYVRERFDNETFRISVYQVLQEHLARESD-LFLLDSRTLWASEEGWLVFDITATSNHWVVNP 239

[NP_001295493.1](https://www.ncbi.nlm.nih.gov/protein/NP_001295493.1?report=genbank&log$=protalign&blast_rank=7&RID=0) 161 SKIPEGEAVTAAEFRIYKDYIREHFHNETFRISVYQVLQEHLGRESD-LFLLDSRTLWASEEGWLVFDITATSNHWVVNP 239

[XP_001170064.1](https://www.ncbi.nlm.nih.gov/protein/XP_001170064.1?report=genbank&log$=protalign&blast_rank=8&RID=0) 161 SKIPEGEAVTAAEFRIYKDYIRERFDNETFRISVYQVLQEHLGRESD-LFLLDSRTLWASEEGWLVFDITATSNHWVVNP 239

[XP_001089245.1](https://www.ncbi.nlm.nih.gov/protein/XP_001089245.1?report=genbank&log$=protalign&blast_rank=9&RID=0) 161 SKIPEGEAVTAAEFRIYKDYIRERFDNETFRISVYQVLQEHLGRESD-LFLLDSRTLWASEEGWLVFDITATSNHWVVNP 239

[XP_011279062.2](https://www.ncbi.nlm.nih.gov/protein/XP_011279062.2?report=genbank&log$=protalign&blast_rank=10&RID=0) 161 SKIPEGKPWTAAEFRIYKDYIRKXLNNETFRISVYQVLQEHLGRESD-LFLLDSRTLWASEEGWLVFDITATSNHWVVNP 239

[XP_017913119.1](https://www.ncbi.nlm.nih.gov/protein/XP_017913119.1?report=genbank&log$=protalign&blast_rank=11&RID=0) 161 SKIPEGEAVTAAEFRIYKDYIREHFHNETFRISVYQVLQEHLGRESD-LFLLDSRTLWASEEGWLVFDITATSNHWVVNP 239

[XP_030860862.1](https://www.ncbi.nlm.nih.gov/protein/XP_030860862.1?report=genbank&log$=protalign&blast_rank=12&RID=0) 161 SKIPEGEAVTAAEFRIYKDYIRERFDNETFRISVYQVLQEHLGRESD-LFLLDSRTLWASEEGWLVFDITATSNHWVVNP 239

[XP_024094889.1](https://www.ncbi.nlm.nih.gov/protein/XP_024094889.1?report=genbank&log$=protalign&blast_rank=13&RID=0) 161 SKIPEGEAVTAAEFRIYKDYIRERFDNETFRISVYQVLQEHLGRESD-LFLLDSRTLWASEEGWLVFDITATSNHWVVNP 239

[XP_031998385.1](https://www.ncbi.nlm.nih.gov/protein/XP_031998385.1?report=genbank&log$=protalign&blast_rank=14&RID=0) 161 SKIPEGEAVTAAEFRIYKDYIRERFDNETFRISVFQVLQEHLGRESD-LFLLDSRTLWASEEGWLVFDITATSNHWVVNP 239

[NP_001710.1](https://www.ncbi.nlm.nih.gov/protein/NP_001710.1?report=genbank&log$=protalign&blast_rank=0&RID=0) 240 RHNLGLQLSVETLDGQSINPKLAGLIGRHGPQNKQPFMVAFFKATEVHFRSIRSTGSKQRSQNRSKTPKNQEALRMANV- 318

[NP_031583.2](https://www.ncbi.nlm.nih.gov/protein/NP_031583.2?report=genbank&log$=protalign&blast_rank=1&RID=0) 239 RHNLGLQLSVETLDGQSINPKLAGLIGRHGPQNKQPFMVAFFKATEVHLRSIRSTGGKQRSQNRSKTPKNQEALRMASV- 317

[NP_001178785.1](https://www.ncbi.nlm.nih.gov/protein/NP_001178785.1?report=genbank&log$=protalign&blast_rank=2&RID=0) 239 RHNLGLQLSVETLDGQSINPKLAGLIGRHGPQNKQPFMVAFFKATEVHLRSIRSTGGKQRSQNRSKTPKNQEALRMASV- 317

[NP_001192944.1](https://www.ncbi.nlm.nih.gov/protein/NP_001192944.1?report=genbank&log$=protalign&blast_rank=3&RID=0) 240 RHNLGLQLSVETLDGQSINPKLAGLIGRQGPQNKQPFMVAFFKATEVHLRSTRSTGGKQRSQNRSKTPKNQEALRVANV- 318

[XP_005673101.1](https://www.ncbi.nlm.nih.gov/protein/XP_005673101.1?report=genbank&log$=protalign&blast_rank=4&RID=0) 240 RHNLGLQLSVETLDGQSINPKLAGLIGRQGPQNKQPFMVAFFKATEVHLRSTRSTGGKQRSQNRSKTPKNQEALRVANV- 318

[NP_001183981.1](https://www.ncbi.nlm.nih.gov/protein/NP_001183981.1?report=genbank&log$=protalign&blast_rank=5&RID=0) 241 RHNLGLQLCVETLDGQSINPKLAGLIGRHGPQNKQPFMVAFFKATEVHLRSTRSTGAKQRSQNRSKTPKNQEALRVANVa 320

[NP_001182087.1](https://www.ncbi.nlm.nih.gov/protein/NP_001182087.1?report=genbank&log$=protalign&blast_rank=6&RID=0) 240 RHNLGLQLSVETLDGQSVNPKLAGLIGRHGPQTKQPFMVAFFKATEVHLRSTRSTGGKQRSQNRSKTPKNQEALRVANV- 318

[NP_001295493.1](https://www.ncbi.nlm.nih.gov/protein/NP_001295493.1?report=genbank&log$=protalign&blast_rank=7&RID=0) 240 RHNLGLQLSVETLDGQSINPKLAGLIGRQGPQNKQPFMVAFFKATEVHLRSTRSTGGKQRSQNRSKTPKNQEALRVANV- 318

[XP_001170064.1](https://www.ncbi.nlm.nih.gov/protein/XP_001170064.1?report=genbank&log$=protalign&blast_rank=8&RID=0) 240 RHNLGLQLSVETLDGQSINPKLAGLIGRHGPQNKQPFMVAFFKATEVHFRSIRSTGSKQRSQNRSKTPKNQEALRMANV- 318

[XP_001089245.1](https://www.ncbi.nlm.nih.gov/protein/XP_001089245.1?report=genbank&log$=protalign&blast_rank=9&RID=0) 240 RHNLGLQLSVETLDGQSINPKLAGLIGRHGPQNKQPFMVAFFKATEVHFRSIRSTGSKQRSQNRSKTPKNQEALRMANV- 318

[XP_011279062.2](https://www.ncbi.nlm.nih.gov/protein/XP_011279062.2?report=genbank&log$=protalign&blast_rank=10&RID=0) 240 RHNLGLQLCVETLDGQSINPKLAGLIGRHGPQNKQPFMVAFFKATEVHLRSTRSTGGKQRSQNRSKTPXNQEALRVTNV- 318

[XP_017913119.1](https://www.ncbi.nlm.nih.gov/protein/XP_017913119.1?report=genbank&log$=protalign&blast_rank=11&RID=0) 240 RHNLGLQLSVETLDGQSINPKLAGLIGRQGPQNKQPFMVAFFKATEVHLRSTRSTGGKQRSQNRSKTPKNQEALRVANV- 318

[XP_030860862.1](https://www.ncbi.nlm.nih.gov/protein/XP_030860862.1?report=genbank&log$=protalign&blast_rank=12&RID=0) 240 RHNLGLQLSVETLDGQSINPKLAGLIGRHGPQNKQPFMVAFFKATEVHFRSIRSTGSKQRSQNRSKTPKNQEALRMANV- 318

[XP_024094889.1](https://www.ncbi.nlm.nih.gov/protein/XP_024094889.1?report=genbank&log$=protalign&blast_rank=13&RID=0) 240 RHNLGLQLSVETLDGQSINPKLAGLIGRHGPQNKQPFMVAFFKATEVHFRSIRSTGSKQRSQNRSKTPKNQEALRMANV- 318

[XP_031998385.1](https://www.ncbi.nlm.nih.gov/protein/XP_031998385.1?report=genbank&log$=protalign&blast_rank=14&RID=0) 240 RHNLGLQLSVETLDGQSINPKLAGLIGRHGPQNKQPFMVAFFKATEVHFRSIRSTGSKQRSQNRSKTPKNQEALRMANV- 318

[NP_001710.1](https://www.ncbi.nlm.nih.gov/protein/NP_001710.1?report=genbank&log$=protalign&blast_rank=0&RID=0) 319 AENSSSDQRQACKKHELYVSFRDLGWQDWIIAPEGYAAYYCEGECAFPLNSYMNATNHAIVQTLVHFINPETVPKPCCAP 398

[NP_031583.2](https://www.ncbi.nlm.nih.gov/protein/NP_031583.2?report=genbank&log$=protalign&blast_rank=1&RID=0) 318 AENSSSDQRQACKKHELYVSFRDLGWQDWIIAPEGYAAYYCEGECAFPLNSYMNATNHAIVQTLVHFINPDTVPKPCCAP 397

[NP_001178785.1](https://www.ncbi.nlm.nih.gov/protein/NP_001178785.1?report=genbank&log$=protalign&blast_rank=2&RID=0) 318 AENSSSDQRQACKKHELYVSFRDLGWQDWIIAPEGYAAYYCEGECAFPLNSYMNATNHAIVQTLVHFINPDTVPKPCCAP 397

[NP_001192944.1](https://www.ncbi.nlm.nih.gov/protein/NP_001192944.1?report=genbank&log$=protalign&blast_rank=3&RID=0) 319 AENSSSDQRQACKKHELYVSFRDLGWQDWIIAPEGYAAYYCEGECAFPLNSYMNATNHAIVQTLVHFINPETVPKPCCAP 398

[XP_005673101.1](https://www.ncbi.nlm.nih.gov/protein/XP_005673101.1?report=genbank&log$=protalign&blast_rank=4&RID=0) 319 AENSSSDQRQACKKHELYVSFRDLGWQDWIIAPEGYAAYYCEGECAFPLNSYMNATNHAIVQTLVHFINPETVPKPCCAP 398

[NP_001183981.1](https://www.ncbi.nlm.nih.gov/protein/NP_001183981.1?report=genbank&log$=protalign&blast_rank=5&RID=0) 321 AKNSSSDQRQACKKHELYVSFRDLGWQDWIIAPEGYAAYYCEGECAFPLNSYMNATNHAIVQTLVHFINPETVPKPCCAP 400

[NP_001182087.1](https://www.ncbi.nlm.nih.gov/protein/NP_001182087.1?report=genbank&log$=protalign&blast_rank=6&RID=0) 319 AENSSSDQRQACKKHELYVSFRDLGWQDWIIAPEGYAAYYCEGECAFPLNSYMNATNHAIVQTLVHFINPETVPKPCCAP 398

[NP_001295493.1](https://www.ncbi.nlm.nih.gov/protein/NP_001295493.1?report=genbank&log$=protalign&blast_rank=7&RID=0) 319 AENSSSDQRQACKKHELYVSFRDLGWQDWIIAPEGYAAYYCEGECAFPLNSYMNATNHAIVQTLVHFINPETVPKPCCAP 398

[XP_001170064.1](https://www.ncbi.nlm.nih.gov/protein/XP_001170064.1?report=genbank&log$=protalign&blast_rank=8&RID=0) 319 AENSSSDQRQACKKHELYVSFRDLGWQDWIIAPEGYAAYYCEGECAFPLNSYMNATNHAIVQTLVHFINPETVPKPCCAP 398

[XP_001089245.1](https://www.ncbi.nlm.nih.gov/protein/XP_001089245.1?report=genbank&log$=protalign&blast_rank=9&RID=0) 319 AENSSSDQRQACKKHELYVSFRDLGWQDWIIAPEGYAAYYCEGECAFPLNSYMNATNHAIVQTLVHFINPETVPKPCCAP 398

[XP_011279062.2](https://www.ncbi.nlm.nih.gov/protein/XP_011279062.2?report=genbank&log$=protalign&blast_rank=10&RID=0) 319 AENSSSDQRQACKKHELYVSFRDLGWQDWIIAPEGYAAYYCEGECAFPLNSYMNATNHAIVQTLVHFINPETVPKPCCAP 398

[XP_017913119.1](https://www.ncbi.nlm.nih.gov/protein/XP_017913119.1?report=genbank&log$=protalign&blast_rank=11&RID=0) 319 AENSSSDQRQACKKHELYVSFRDLGWQDWIIAPEGYAAYYCEGECAFPLNSYMNATNHAIVQTLVHFINPETVPKPCCAP 398

[XP_030860862.1](https://www.ncbi.nlm.nih.gov/protein/XP_030860862.1?report=genbank&log$=protalign&blast_rank=12&RID=0) 319 AENSSSDQRQACKKHELYVSFRDLGWQDWIIAPEGYAAYYCEGECAFPLNSYMNATNHAIVQTLVHFINPETVPKPCCAP 398

[XP_024094889.1](https://www.ncbi.nlm.nih.gov/protein/XP_024094889.1?report=genbank&log$=protalign&blast_rank=13&RID=0) 319 AENSSSDQRQACKKHELYVSFRDLGWQDWIIAPEGYAAYYCEGECAFPLNSYMNATNHAIVQTLVHFINPETVPKPCCAP 398

[XP_031998385.1](https://www.ncbi.nlm.nih.gov/protein/XP_031998385.1?report=genbank&log$=protalign&blast_rank=14&RID=0) 319 AENSSSDQRQACKKHELYVSFRDLGWQDWIIAPEGYAAYYCEGECAFPLNSYMNATNHAIVQTLVHFINPETVPKPCCAP 398

[NP_001710.1](https://www.ncbi.nlm.nih.gov/protein/NP_001710.1?report=genbank&log$=protalign&blast_rank=0&RID=0) 399 TQLNAISVLYFDDSSNVILKKYRNMVVRACGCH 431

[NP_031583.2](https://www.ncbi.nlm.nih.gov/protein/NP_031583.2?report=genbank&log$=protalign&blast_rank=1&RID=0) 398 TQLNAISVLYFDDSSNVILKKYRNMVVRACGCH 430

[NP_001178785.1](https://www.ncbi.nlm.nih.gov/protein/NP_001178785.1?report=genbank&log$=protalign&blast_rank=2&RID=0) 398 TQLNAISVLYFDDSSNVILKKYRNMVVRACGCH 430

[NP_001192944.1](https://www.ncbi.nlm.nih.gov/protein/NP_001192944.1?report=genbank&log$=protalign&blast_rank=3&RID=0) 399 TQLNAISVLYFDDSSNVILKKYRNMVVRACGCH 431

[XP_005673101.1](https://www.ncbi.nlm.nih.gov/protein/XP_005673101.1?report=genbank&log$=protalign&blast_rank=4&RID=0) 399 TQLNAISVLYFDDSSNVILKKYRNMVVRACGCH 431

[NP_001183981.1](https://www.ncbi.nlm.nih.gov/protein/NP_001183981.1?report=genbank&log$=protalign&blast_rank=5&RID=0) 401 TQLNAISVLYFDDSSNVILKKYRNMVVRACGCH 433

[NP_001182087.1](https://www.ncbi.nlm.nih.gov/protein/NP_001182087.1?report=genbank&log$=protalign&blast_rank=6&RID=0) 399 TQLNAISVLYFDDSSNVILKKYRNMVVRACGCH 431

[NP_001295493.1](https://www.ncbi.nlm.nih.gov/protein/NP_001295493.1?report=genbank&log$=protalign&blast_rank=7&RID=0) 399 TQLNAISVLYFDDSSNVILKKYRNMVVRACGCH 431

[XP_001170064.1](https://www.ncbi.nlm.nih.gov/protein/XP_001170064.1?report=genbank&log$=protalign&blast_rank=8&RID=0) 399 TQLNAISVLYFDDSSNVILKKYRNMVVRACGCH 431

[XP_001089245.1](https://www.ncbi.nlm.nih.gov/protein/XP_001089245.1?report=genbank&log$=protalign&blast_rank=9&RID=0) 399 TQLNAISVLYFDDSSNVILKKYRNMVVRACGCH 431

[XP_011279062.2](https://www.ncbi.nlm.nih.gov/protein/XP_011279062.2?report=genbank&log$=protalign&blast_rank=10&RID=0) 399 TQLNAISVLYFDDSSNVILKKYRNMVVRACGCH 431

[XP_017913119.1](https://www.ncbi.nlm.nih.gov/protein/XP_017913119.1?report=genbank&log$=protalign&blast_rank=11&RID=0) 399 TQLNAISVLYFDDSSNVILKKYRNMVVRACGCH 431

[XP_030860862.1](https://www.ncbi.nlm.nih.gov/protein/XP_030860862.1?report=genbank&log$=protalign&blast_rank=12&RID=0) 399 TQLNAISVLYFDDSSNVILKKYRNMVVRACGCH 431

[XP_024094889.1](https://www.ncbi.nlm.nih.gov/protein/XP_024094889.1?report=genbank&log$=protalign&blast_rank=13&RID=0) 399 TQLNAISVLYFDDSSNVILKKYRNMVVRACGCH 431

[XP_031998385.1](https://www.ncbi.nlm.nih.gov/protein/XP_031998385.1?report=genbank&log$=protalign&blast_rank=14&RID=0) 399 TQLNAISVLYFDDSSNVILKKYRNMVVRACGCH 431

BMP5

NP_066551.1 bone morphogenetic protein 5 isoform 1 preproprotein [Homo sapiens]

NP_031581.2 bone morphogenetic protein 5 preproprotein [Mus musculus]

NP_001101638.1 bone morphogenetic protein 5 precursor [Rattus norvegicus]

NP_001291945.1 bone morphogenetic protein 5 precursor [Bos taurus]

NP_001191830.1 bone morphogenetic protein 5 [Sus scrofa]

XP_532179.2 bone morphogenetic protein 5 [Canis lupus familiaris]

XP_001503274.1 bone morphogenetic protein 5 [Equus caballus]

XP_004018779.1 bone morphogenetic protein 5 [Ovis aries]

XP_518553.2 bone morphogenetic protein 5 isoform X1 [Pan troglodytes]

XP_001109809.1 bone morphogenetic protein 5 [Macaca mulatta]

XP_003986323.1 bone morphogenetic protein 5 [Felis catus]

XP_005696245.1 PREDICTED: bone morphogenetic protein 5 [Capra hircus]

XP_030868477.1 bone morphogenetic protein 5 isoform X1 [Gorilla gorilla gorilla]

XP_002817070.2 bone morphogenetic protein 5 isoform X1 [Pongo abelii]

XP_031996280.1 bone morphogenetic protein 5 isoform X1 [Hylobates moloch]

[NP_066551.1](https://www.ncbi.nlm.nih.gov/protein/NP_066551.1?report=genbank&log$=protalign&blast_rank=0&RID=0) 1 MHLTVFLLKGIVGFLWSCWVLVGYAKGGLGDNHVHSSFIYRRLRNHERREIQREILSILGLPHRPRPFSPGKQASSAPLF 80

[NP_031581.2](https://www.ncbi.nlm.nih.gov/protein/NP_031581.2?report=genbank&log$=protalign&blast_rank=1&RID=0) 1 MHWTVFLLRGIVGFLWSGWVQVGYAKGGLGDNHVHSSFIYRRLRNHERREIQREILSILGLPHRPRPFSPGKQASSAPLF 80

[NP_001101638.1](https://www.ncbi.nlm.nih.gov/protein/NP_001101638.1?report=genbank&log$=protalign&blast_rank=2&RID=0) 1 MHWTVFLLRGIVGFLWSSWVQVGYAKGGLGDNHVHSSFIYRRLRNHERREIQREILSILGLPHRPRPFSPGKQASSAPLF 80

[NP_001291945.1](https://www.ncbi.nlm.nih.gov/protein/NP_001291945.1?report=genbank&log$=protalign&blast_rank=3&RID=0) 1 MHLTVFLLRGIVGFLWSCWVLVGSAKGSLGDNHVHSSFIYRRLRNHERREIQREILSILGLPHRPRPFSPGKQASSAPLF 80

[NP_001191830.1](https://www.ncbi.nlm.nih.gov/protein/NP_001191830.1?report=genbank&log$=protalign&blast_rank=4&RID=0) 1 MHLTVFLLRGIVGFLWSCWVLVGYAKGGLGDNHVHSSFIYRRLRNHERREIQREILSILGLPHRPRPFSPGKQASSAPLF 80

[XP_532179.2](https://www.ncbi.nlm.nih.gov/protein/XP_532179.2?report=genbank&log$=protalign&blast_rank=5&RID=0) 1 MHLTVFLLRSIVGFLWSCWVLVGYAKGGLGDNHVHSSFIYRRLRNHERREIQREILSILGLPHRPRPFSPGKQASSAPLF 80

[XP_001503274.1](https://www.ncbi.nlm.nih.gov/protein/XP_001503274.1?report=genbank&log$=protalign&blast_rank=6&RID=0) 1 MHLTVFLLRGIVGFLWSCWVLVGYAKGGLGDNHVHSSFIYRRLRNHERREIQREILSILGLPHRPRPFSPGKQASSAPLF 80

[XP_004018779.1](https://www.ncbi.nlm.nih.gov/protein/XP_004018779.1?report=genbank&log$=protalign&blast_rank=7&RID=0) 1 MHLTVFLLRGIVGFLWSCWVLVGSAKGSLGDNHVHSSFIYRRLRNHERREIQREILSILGLPHRPRPFSPGKQASSAPLF 80

[XP_518553.2](https://www.ncbi.nlm.nih.gov/protein/XP_518553.2?report=genbank&log$=protalign&blast_rank=8&RID=0) 1 MHLTVFLLKGIVGFLWSCWVLVGYAKGGLGDNHVHSSFIYRRLRNHERREIQREILSILGLPHRPRPFSPGKQASSAPLF 80

[XP_001109809.1](https://www.ncbi.nlm.nih.gov/protein/XP_001109809.1?report=genbank&log$=protalign&blast_rank=9&RID=0) 1 MHLTVFLLKGIVGFLWSCWVLVGYAKGGLGDNHVHSSFIYRRLRNHERREIQREILSILGLPHRPRPFSPGKQASSAPLF 80

[XP_003986323.1](https://www.ncbi.nlm.nih.gov/protein/XP_003986323.1?report=genbank&log$=protalign&blast_rank=10&RID=0) 1 MHLTVFLLRSIVGFLWSCWVLVGYAKGGLGDNHVHSSFIYRRLRNHERREIQREILSILGLPHRPRPFSPGKQASSAPLF 80

[XP_005696245.1](https://www.ncbi.nlm.nih.gov/protein/XP_005696245.1?report=genbank&log$=protalign&blast_rank=11&RID=0) 1 MHLTVFLLRGIVGFLWSCWVLVGSAKGSLGDNHVHSSFIYRRLRNHERREIQREILSILGLPHRPRPFSPGKQASSAPLF 80

[XP_030868477.1](https://www.ncbi.nlm.nih.gov/protein/XP_030868477.1?report=genbank&log$=protalign&blast_rank=12&RID=0) 1 MHLTVFLLKGIVGFLWSCWVLVGYAKGGLGDNHVHSSFIYRRLRNHERREIQREILSILGLPHRPRPFSPGKQASSAPLF 80

[XP_002817070.2](https://www.ncbi.nlm.nih.gov/protein/XP_002817070.2?report=genbank&log$=protalign&blast_rank=13&RID=0) 1 MHLTVFLLKGIVGFLWSCWVLVGYAKGGLGDNHVHSSFIYRRLRNHERREIQREILSILGLPHRPRPFSPGKQASSAPLF 80

[XP_031996280.1](https://www.ncbi.nlm.nih.gov/protein/XP_031996280.1?report=genbank&log$=protalign&blast_rank=14&RID=0) 1 MHLTVFLLKGIVGFLWSCWVLVGYAKGGLGDNHVHSSFIYRRLRNHERREIQREILSILGLPHRPRPFSPGKQASSAPLF 80

[NP_066551.1](https://www.ncbi.nlm.nih.gov/protein/NP_066551.1?report=genbank&log$=protalign&blast_rank=0&RID=0) 81 MLDLYNAMTNEENPEESEYSVRASLAEETRGARKGYPASPNGYPRRIQLSRTTPLTTQSPPLASLHDTNFLNDADMVMSF 160

[NP_031581.2](https://www.ncbi.nlm.nih.gov/protein/NP_031581.2?report=genbank&log$=protalign&blast_rank=1&RID=0) 81 MLDLYNAMASEDNPEESEYLVRVSLAGEAKETRKGYPASPNGYAHRLHLPPRTPLTTQSPPLASLHDTNFLNDADMVMSF 160

[NP_001101638.1](https://www.ncbi.nlm.nih.gov/protein/NP_001101638.1?report=genbank&log$=protalign&blast_rank=2&RID=0) 81 MLDLYNAMASEENPEESEYLVRVSLAGEAKETRKGYPASPNGYAHRLHLPPRIPLTTQSPPLASLHDTNFLNDADMVMSF 160

[NP_001291945.1](https://www.ncbi.nlm.nih.gov/protein/NP_001291945.1?report=genbank&log$=protalign&blast_rank=3&RID=0) 81 MLDLYNAMASEENPEELEYSVRAPLAADSRGARKGSPASPNGYPRRIQLSRASPLTTQSPPLASLHDANFLNDADMVMSF 160

[NP_001191830.1](https://www.ncbi.nlm.nih.gov/protein/NP_001191830.1?report=genbank&log$=protalign&blast_rank=4&RID=0) 81 MLDLYNAMASEENPEEPEYSVRASLAGETRGARKGSPASPNGYPRRIQLSRTTPLTTQSPPLASLHDANFLNDADMVMSF 160

[XP_532179.2](https://www.ncbi.nlm.nih.gov/protein/XP_532179.2?report=genbank&log$=protalign&blast_rank=5&RID=0) 81 MLDLYNAMANEENPEESEYSVRASLAGETRGTRKGYPASPNGYPRRIQLSRTTPLTTQSPPLASLHDTNFLNDADMVMSF 160

[XP_001503274.1](https://www.ncbi.nlm.nih.gov/protein/XP_001503274.1?report=genbank&log$=protalign&blast_rank=6&RID=0) 81 MLDLYNAMANEENPDETEYSVRASLAGETRGGRKGYPASPNGYPRGIQLSRTAPLTTQSPPLASLHDTNFLNDADMVMSF 160

[XP_004018779.1](https://www.ncbi.nlm.nih.gov/protein/XP_004018779.1?report=genbank&log$=protalign&blast_rank=7&RID=0) 81 MLDLYNAMASEENPEELEYSVTAPLAADSRGSRKGSPASPNGYPRRIQLSRASPLTTQSPPLASLHDANFLNDADMVMSF 160

[XP_518553.2](https://www.ncbi.nlm.nih.gov/protein/XP_518553.2?report=genbank&log$=protalign&blast_rank=8&RID=0) 81 MLDLYNAMTNEENPEESEYSVRASLAEETRGARKGYPASPNGYPRRIQLSRTTPLTTQSPPLASLHDTNFLNDADMVMSF 160

[XP_001109809.1](https://www.ncbi.nlm.nih.gov/protein/XP_001109809.1?report=genbank&log$=protalign&blast_rank=9&RID=0) 81 MLDLYNAMTNEENPEESEYSVRASLAEETRGARKGYPASPNGYPRRIQLSRTTPLTTQSPPLASLHDTNFLNDADMVMSF 160

[XP_003986323.1](https://www.ncbi.nlm.nih.gov/protein/XP_003986323.1?report=genbank&log$=protalign&blast_rank=10&RID=0) 81 MLDLYNAMANEENPEESEYSVRASLAGEARGARKGYPASPNGYPRRIQLSRTTPLTTQSPPLASLHDTNFLNDADMVMSF 160

[XP_005696245.1](https://www.ncbi.nlm.nih.gov/protein/XP_005696245.1?report=genbank&log$=protalign&blast_rank=11&RID=0) 81 MLDLYNAMASEENPEELEYSVTAPLAADSRGSRKGSPASPNGYPRRIQLSRASPLTTQSPPLASLHDANFLNDADMVMSF 160

[XP_030868477.1](https://www.ncbi.nlm.nih.gov/protein/XP_030868477.1?report=genbank&log$=protalign&blast_rank=12&RID=0) 81 MLDLYNAMTNEENPEESEYSVRASLAEETRGARKGYPASPNGYPRRIQLSRTTPLTTQSPPLASLHDTNFLNDADMVMSF 160

[XP_002817070.2](https://www.ncbi.nlm.nih.gov/protein/XP_002817070.2?report=genbank&log$=protalign&blast_rank=13&RID=0) 81 MLDLYNAMTNEENPEESEYSVRASLAEETRGARKGYPASPNGYPRRIQLSRTTPLTTQSPPLASLHDTNFLNDADMVMSF 160

[XP_031996280.1](https://www.ncbi.nlm.nih.gov/protein/XP_031996280.1?report=genbank&log$=protalign&blast_rank=14&RID=0) 81 MLDLYNAMTNEENPEESEYSVRASLAEETRGARRGYPASPNGYPRGIQLSRTTPLTTQSPPLASLHDTNFLNDADMVMSF 160

[NP_066551.1](https://www.ncbi.nlm.nih.gov/protein/NP_066551.1?report=genbank&log$=protalign&blast_rank=0&RID=0) 161 VNLVERDKDFSHQRRHYKEFRFDLTQIPHGEAVTAAEFRIYKDRSNNRFENETIKISIYQIIKEYTNRDADLFLLDTRKA 240

[NP_031581.2](https://www.ncbi.nlm.nih.gov/protein/NP_031581.2?report=genbank&log$=protalign&blast_rank=1&RID=0) 161 VNLVERDKDFSHQRRHYKEFRFDLTQIPHGEAVTAAEFRIYKDKGNHRFENETIKISIYQIIKEYTNRDADLFLLDTRKT 240

[NP_001101638.1](https://www.ncbi.nlm.nih.gov/protein/NP_001101638.1?report=genbank&log$=protalign&blast_rank=2&RID=0) 161 VNLVERDKDFSHQRRHYKEFRFDLTQIPHGEAVTAAEFRIYKDKSNHRFENETIKISIYQIIKEYTNRDADLFLLDTRKA 240

[NP_001291945.1](https://www.ncbi.nlm.nih.gov/protein/NP_001291945.1?report=genbank&log$=protalign&blast_rank=3&RID=0) 161 VNLVERDKDFSHQRRHYKEFRFDLTQIPQGEAVTAAEFRIYKDRSNGRFENETIKISIYQIIKEYANRDADLFLLDTRKT 240

[NP_001191830.1](https://www.ncbi.nlm.nih.gov/protein/NP_001191830.1?report=genbank&log$=protalign&blast_rank=4&RID=0) 161 VNLVERDKDFSHQRRHYKEFRFDLTQIPHGEAVTAAEFRIYKDRSNSRFENETIKISIYQIIKEYTNRDADLFLLDTRKV 240

[XP_532179.2](https://www.ncbi.nlm.nih.gov/protein/XP_532179.2?report=genbank&log$=protalign&blast_rank=5&RID=0) 161 VNLVERDKDFSHQRRHYKEFRFDLTQIPHGEAVTAAEFRIYKDQSNSRFENETIKISIYQIIKEYTNRDADLFLLDTRKA 240

[XP_001503274.1](https://www.ncbi.nlm.nih.gov/protein/XP_001503274.1?report=genbank&log$=protalign&blast_rank=6&RID=0) 161 VNLVERDKDFSHQRRHYKEFRFDLTQIPHGEAVTAAEFRIYKDRSNSRFENETIKISIYQIIKEYTNRDADLFLLDTRKA 240

[XP_004018779.1](https://www.ncbi.nlm.nih.gov/protein/XP_004018779.1?report=genbank&log$=protalign&blast_rank=7&RID=0) 161 VNLVERDKDFSHQRRHYKEFRFDLTQIPQGEAVTAAEFRIYKDRSNGRFENETIKISIYQIIKEYTNRDADLFLLDTRKT 240

[XP_518553.2](https://www.ncbi.nlm.nih.gov/protein/XP_518553.2?report=genbank&log$=protalign&blast_rank=8&RID=0) 161 VNLVERDKDFSHQRRHYKEFRFDLTQIPHGEAVTAAEFRIYKDRSNNRFENETIKISIYQIIKEYTNRDADLFLLDTRKA 240

[XP_001109809.1](https://www.ncbi.nlm.nih.gov/protein/XP_001109809.1?report=genbank&log$=protalign&blast_rank=9&RID=0) 161 VNLVERDKDFSHQRRHYKEFRFDLTQIPHGEAVTAAEFRIYKDRSNNRFENETIKISIYQIIKEYTNRDADLFLLDTRKA 240

[XP_003986323.1](https://www.ncbi.nlm.nih.gov/protein/XP_003986323.1?report=genbank&log$=protalign&blast_rank=10&RID=0) 161 VNLVERDKDFSHQRRHYKEFRFDLTQIPHGEAVTAAEFRIYKDQSNSRFENETIKISIYQIIKEYTNRDADLFLLDTRKT 240

[XP_005696245.1](https://www.ncbi.nlm.nih.gov/protein/XP_005696245.1?report=genbank&log$=protalign&blast_rank=11&RID=0) 161 VNLVERDKDFSHQRRHYKEFRFDLTQIPQGEAVTAAEFRIYKDRSNGRFENETIKISIYQIIKEYTNRDADLFLLDTRKT 240

[XP_030868477.1](https://www.ncbi.nlm.nih.gov/protein/XP_030868477.1?report=genbank&log$=protalign&blast_rank=12&RID=0) 161 VNLVERDKDFSHQRRHYKEFRFDLTQIPHGEAVTAAEFRIYKDRSNNRFENETIKISIYQIIKEYTNRDADLFLLDTRKA 240

[XP_002817070.2](https://www.ncbi.nlm.nih.gov/protein/XP_002817070.2?report=genbank&log$=protalign&blast_rank=13&RID=0) 161 VNLVERDKDFSHQRRHYKEFRFDLTQIPHGEAVTAAEFRIYKDRSNNRFENETIKISIYQIIKEYTNRDADLFLLDTRKA 240

[XP_031996280.1](https://www.ncbi.nlm.nih.gov/protein/XP_031996280.1?report=genbank&log$=protalign&blast_rank=14&RID=0) 161 VNLVERDKDFSHQRRHYKEFRFDLTQIPHGEAVTAAEFRIYKDRSNNRFENETIKISIYQIIKEYTNRDADLFLLDTRKA 240

[NP_066551.1](https://www.ncbi.nlm.nih.gov/protein/NP_066551.1?report=genbank&log$=protalign&blast_rank=0&RID=0) 241 QALDVGWLVFDITVTSNHWVINPQNNLGLQLCAETGDGRSINVKSAGLVGRQGPQSKQPFMVAFFKASEVLLRSVRAANK 320

[NP_031581.2](https://www.ncbi.nlm.nih.gov/protein/NP_031581.2?report=genbank&log$=protalign&blast_rank=1&RID=0) 241 QALDVGWLVFDITVTSNHWVINPQNNLGLQLCAETGDGRSINVKSAGLVGRHGPQSKQPFMVAFFKASEVLLRSVRAASK 320

[NP_001101638.1](https://www.ncbi.nlm.nih.gov/protein/NP_001101638.1?report=genbank&log$=protalign&blast_rank=2&RID=0) 241 QALDVGWLVFDITVTSNHWVINPQNNLGLQLCAETGDGRSINVKSAGLVGRHGPQSKQPFMVAFFKASEVLLRSVRAASK 320

[NP_001291945.1](https://www.ncbi.nlm.nih.gov/protein/NP_001291945.1?report=genbank&log$=protalign&blast_rank=3&RID=0) 241 QALDVGWLVFDITVTSNHWVINPQNNLGLQLCAETGDGHSINVKSAGLVGRHGPQSKQPFMVAFFKASEVLLRSVRAANK 320

[NP_001191830.1](https://www.ncbi.nlm.nih.gov/protein/NP_001191830.1?report=genbank&log$=protalign&blast_rank=4&RID=0) 241 QALDVGWLVFDITVTSNHWVINPQNNLGLQLCAETGDGHSISVKSAGLVGRHGPQSKQPFMVAFFKASEVLLRSVRAANK 320

[XP_532179.2](https://www.ncbi.nlm.nih.gov/protein/XP_532179.2?report=genbank&log$=protalign&blast_rank=5&RID=0) 241 QALDVGWLVFDITVTSNHWVINPQNNLGLQLCAETGDGRSINVKSAGLVGRHGPQSKQPFMVAFFKASEVLLRSVRAANK 320

[XP_001503274.1](https://www.ncbi.nlm.nih.gov/protein/XP_001503274.1?report=genbank&log$=protalign&blast_rank=6&RID=0) 241 EALDVGWLVFDITVTSNHWVINPQNNLGLQLCAETGDGRSINVKSAGLVGRHGPQSKQPFMVAFFKASEVLLRSVRAANK 320

[XP_004018779.1](https://www.ncbi.nlm.nih.gov/protein/XP_004018779.1?report=genbank&log$=protalign&blast_rank=7&RID=0) 241 QALDVGWLVFDITVTSNHWVINPQNNLGLQLCAETGDGHSINVKSAGLVGRHGPQSKQPFMVAFFKASEVLLRSVRAANK 320

[XP_518553.2](https://www.ncbi.nlm.nih.gov/protein/XP_518553.2?report=genbank&log$=protalign&blast_rank=8&RID=0) 241 QALDVGWLVFDITVTSNHWVINPQNNLGLQLCAETGDGRSINVKSAGLVGRQGPQSKQPFMVAFFKASEVLLRSVRAANK 320

[XP_001109809.1](https://www.ncbi.nlm.nih.gov/protein/XP_001109809.1?report=genbank&log$=protalign&blast_rank=9&RID=0) 241 QALDVGWLVFDITVTSNHWVINPQNNLGLQLCAETGDGRSINVKSAGLVGRQGPQSKQPFMVAFFKASEVLLRSVRAANK 320

[XP_003986323.1](https://www.ncbi.nlm.nih.gov/protein/XP_003986323.1?report=genbank&log$=protalign&blast_rank=10&RID=0) 241 QALDVGWLVFDITVTSNHWVINPQNNLGLQLCAETGDGRSINVKSAGLVGRHGPQSKQPFMVAFFKASEVLLRSVRAANK 320

[XP_005696245.1](https://www.ncbi.nlm.nih.gov/protein/XP_005696245.1?report=genbank&log$=protalign&blast_rank=11&RID=0) 241 QALDVGWLVFDITVTSNHWVINPQNNLGLQLCAETGDGHSINVKSAGLVGRHGPQSKQPFMVAFFKASEVLLRSVRAANK 320

[XP_030868477.1](https://www.ncbi.nlm.nih.gov/protein/XP_030868477.1?report=genbank&log$=protalign&blast_rank=12&RID=0) 241 QALDVGWLVFDITVTSNHWVINPQNNLGLQLCAETGDGRSINVKSAGLVGRQGPQSKQPFMVAFFKASEVLLRSVRAANK 320

[XP_002817070.2](https://www.ncbi.nlm.nih.gov/protein/XP_002817070.2?report=genbank&log$=protalign&blast_rank=13&RID=0) 241 QALDVGWLVFDITVTSNHWVINPQNNLGLQLCAETGDGRSINVKSAGLVGRQGPQSKQPFMVAFFKASEVLLRSVRAANK 320

[XP_031996280.1](https://www.ncbi.nlm.nih.gov/protein/XP_031996280.1?report=genbank&log$=protalign&blast_rank=14&RID=0) 241 QALDVGWLVFDITVTSNHWVINPQNNLGLQLCAETGDGRSINVKSAGLVGRQGPQSKQPFMVAFFKASEVLLRSVRAANK 320

[NP_066551.1](https://www.ncbi.nlm.nih.gov/protein/NP_066551.1?report=genbank&log$=protalign&blast_rank=0&RID=0) 321 RKNQNRNKSSSHQDSSRMSSVGDYNTSEQKQACKKHELYVSFRDLGWQDWIIAPEGYAAFYCDGECSFPLNAHMNATNHA 400

[NP_031581.2](https://www.ncbi.nlm.nih.gov/protein/NP_031581.2?report=genbank&log$=protalign&blast_rank=1&RID=0) 321 RKNQNRNKSNSHQDPSRMPSAGDYNTSEQKQACKKHELYVSFRDLGWQDWIIAPEGYAAFYCDGECSFPLNAHMNATNHA 400

[NP_001101638.1](https://www.ncbi.nlm.nih.gov/protein/NP_001101638.1?report=genbank&log$=protalign&blast_rank=2&RID=0) 321 RKNQNRNKSSSHQDPSRIPSAGDYNTSEQKQACKKHELYVSFRDLGWQDWIIAPEGYAAFYCDGECSFPLNAHMNATNHA 400

[NP_001291945.1](https://www.ncbi.nlm.nih.gov/protein/NP_001291945.1?report=genbank&log$=protalign&blast_rank=3&RID=0) 321 RKNQNRNKSGSHQDSSRMSSVGDYNTSEQKQACKKHELYVSFRDLGWQDWIIAPEGYAAFYCDGECSFPLNAHMNATNHA 400

[NP_001191830.1](https://www.ncbi.nlm.nih.gov/protein/NP_001191830.1?report=genbank&log$=protalign&blast_rank=4&RID=0) 321 RKNQNRNKSSSHQDSSRMSSVGDYNTSEQKQACKKHELYVSFRDLGWQDWIIAPEGYAAFYCDGECSFPLNAHMNATNHA 400

[XP_532179.2](https://www.ncbi.nlm.nih.gov/protein/XP_532179.2?report=genbank&log$=protalign&blast_rank=5&RID=0) 321 RKNQNRNKSSSHQDSSRMSSVGDYNTSEQKQACKKHELYVSFRDLGWQDWIIAPEGYAAFYCDGECSFPLNAHMNATNHA 400

[XP_001503274.1](https://www.ncbi.nlm.nih.gov/protein/XP_001503274.1?report=genbank&log$=protalign&blast_rank=6&RID=0) 321 RKNQNRNKSSSHQDSSRVSSVGDYNTSEQKQACKKHELYVSFRDLGWQDWIIAPEGYAAFYCDGECSFPLNAHMNATNHA 400

[XP_004018779.1](https://www.ncbi.nlm.nih.gov/protein/XP_004018779.1?report=genbank&log$=protalign&blast_rank=7&RID=0) 321 RKNQNRNKSSSHQDSSRMSSVGDYNTSEQKQACKKHELYVSFRDLGWQDWIIAPEGYAAFYCDGECSFPLNAHMNATNHA 400

[XP_518553.2](https://www.ncbi.nlm.nih.gov/protein/XP_518553.2?report=genbank&log$=protalign&blast_rank=8&RID=0) 321 RKNQNRNKSSSHQDSSRMSSVGDYNTSEQKQACKKHELYVSFRDLGWQDWIIAPEGYAAFYCDGECSFPLNAHMNATNHA 400

[XP_001109809.1](https://www.ncbi.nlm.nih.gov/protein/XP_001109809.1?report=genbank&log$=protalign&blast_rank=9&RID=0) 321 RKNQNRNKSSSHQDSSRMSSVGDYNTSEQKQACKKHELYVSFRDLGWQDWIIAPEGYAAFYCDGECSFPLNAHMNATNHA 400

[XP_003986323.1](https://www.ncbi.nlm.nih.gov/protein/XP_003986323.1?report=genbank&log$=protalign&blast_rank=10&RID=0) 321 RKNQNRNKSNSHQDSSRMSSVGDYNTSEQKQACKKHELYVSFRDLGWQDWIIAPEGYAAFYCDGECSFPLNAHMNATNHA 400

[XP_005696245.1](https://www.ncbi.nlm.nih.gov/protein/XP_005696245.1?report=genbank&log$=protalign&blast_rank=11&RID=0) 321 RKNQNRNKSSSHQDSSRMSSVGDYNTSEQKQACKKHELYVSFRDLGWQDWIIAPEGYAAFYCDGECSFPLNAHMNATNHA 400

[XP_030868477.1](https://www.ncbi.nlm.nih.gov/protein/XP_030868477.1?report=genbank&log$=protalign&blast_rank=12&RID=0) 321 RKNQNRNKSSSHQDSSRMSNVGDYNTSEQKQACKKHELYVSFRDLGWQDWIIAPEGYAAFYCDGECSFPLNAHMNATNHA 400

[XP_002817070.2](https://www.ncbi.nlm.nih.gov/protein/XP_002817070.2?report=genbank&log$=protalign&blast_rank=13&RID=0) 321 RKNQNRNKSSSHQDSSRMSSVGDYNTSEQKQACKKHELYVSFRDLGWQDWIIAPEGYAAFYCDGECSFPLNAHMNATNHA 400

[XP_031996280.1](https://www.ncbi.nlm.nih.gov/protein/XP_031996280.1?report=genbank&log$=protalign&blast_rank=14&RID=0) 321 RKNQNRNKSSSHQDSSRMSSVGDYNTSEQKQACKKHELYVSFRDLGWQDWIIAPEGYAAFYCDGECSFPLNAHMNATNHA 400

[NP_066551.1](https://www.ncbi.nlm.nih.gov/protein/NP_066551.1?report=genbank&log$=protalign&blast_rank=0&RID=0) 401 IVQTLVHLMFPDHVPKPCCAPTKLNAISVLYFDDSSNVILKKYRNMVVRSCGCH 454

[NP_031581.2](https://www.ncbi.nlm.nih.gov/protein/NP_031581.2?report=genbank&log$=protalign&blast_rank=1&RID=0) 401 IVQTLVHLMFPDHVPKPCCAPTKLNAISVLYFDDSSNVILKKYRNMVVRSCGCH 454

[NP_001101638.1](https://www.ncbi.nlm.nih.gov/protein/NP_001101638.1?report=genbank&log$=protalign&blast_rank=2&RID=0) 401 IVQTLVHLMFPDHVPKPCCAPTKLNAISVLYFDDSSNVILKKYRNMVVRSCGCH 454

[NP_001291945.1](https://www.ncbi.nlm.nih.gov/protein/NP_001291945.1?report=genbank&log$=protalign&blast_rank=3&RID=0) 401 IVQTLVHLMFPDHVPKPCCAPTKLNAISVLYFDDSSNVILKKYRNMVVRSCGCH 454

[NP_001191830.1](https://www.ncbi.nlm.nih.gov/protein/NP_001191830.1?report=genbank&log$=protalign&blast_rank=4&RID=0) 401 IVQTLVHLMFPDHVPKPCCAPTKLNAISVLYFDDSSNVILKKYRNMVVRSCGCH 454

[XP_532179.2](https://www.ncbi.nlm.nih.gov/protein/XP_532179.2?report=genbank&log$=protalign&blast_rank=5&RID=0) 401 IVQTLVHLMFPDHVPKPCCAPTKLNAISVLYFDDSSNVILKKYRNMVVRSCGCH 454

[XP_001503274.1](https://www.ncbi.nlm.nih.gov/protein/XP_001503274.1?report=genbank&log$=protalign&blast_rank=6&RID=0) 401 IVQTLVHLMFPDHVPKPCCAPTKLNAISVLYFDDSSNVILKKYRNMVVRSCGCH 454

[XP_004018779.1](https://www.ncbi.nlm.nih.gov/protein/XP_004018779.1?report=genbank&log$=protalign&blast_rank=7&RID=0) 401 IVQTLVHLMFPDHVPKPCCAPTKLNAISVLYFDDSSNVILKKYRNMVVRSCGCH 454

[XP_518553.2](https://www.ncbi.nlm.nih.gov/protein/XP_518553.2?report=genbank&log$=protalign&blast_rank=8&RID=0) 401 IVQTLVHLMFPDHVPKPCCAPTKLNAISVLYFDDSSNVILKKYRNMVVRSCGCH 454

[XP_001109809.1](https://www.ncbi.nlm.nih.gov/protein/XP_001109809.1?report=genbank&log$=protalign&blast_rank=9&RID=0) 401 IVQTLVHLMFPDHVPKPCCAPTKLNAISVLYFDDSSNVILKKYRNMVVRSCGCH 454

[XP_003986323.1](https://www.ncbi.nlm.nih.gov/protein/XP_003986323.1?report=genbank&log$=protalign&blast_rank=10&RID=0) 401 IVQTLVHLMFPDHVPKPCCAPTKLNAISVLYFDDSSNVILKKYRNMVVRSCGCH 454

[XP_005696245.1](https://www.ncbi.nlm.nih.gov/protein/XP_005696245.1?report=genbank&log$=protalign&blast_rank=11&RID=0) 401 IVQTLVHLMFPDHVPKPCCAPTKLNAISVLYFDDSSNVILKKYRNMVVRSCGCH 454

[XP_030868477.1](https://www.ncbi.nlm.nih.gov/protein/XP_030868477.1?report=genbank&log$=protalign&blast_rank=12&RID=0) 401 IVQTLVHLMFPDHVPKPCCAPTKLNAISVLYFDDSSNVILKKYRNMVVRSCGCH 454

[XP_002817070.2](https://www.ncbi.nlm.nih.gov/protein/XP_002817070.2?report=genbank&log$=protalign&blast_rank=13&RID=0) 401 IVQTLVHLMFPDHVPKPCCAPTKLNAISVLYFDDSSNVILKKYRNMVVRSCGCH 454

[XP_031996280.1](https://www.ncbi.nlm.nih.gov/protein/XP_031996280.1?report=genbank&log$=protalign&blast_rank=14&RID=0) 401 IVQTLVHLMFPDHVPKPCCAPTKLNAISVLYFDDSSNVILKKYRNMVVRSCGCH 454

BMP6

NP_001709.1 bone morphogenetic protein 6 preproprotein [Homo sapiens]

NP_031582.1 bone morphogenetic protein 6 preproprotein [Mus musculus]

XP_038951315.1 bone morphogenetic protein 6 isoform X1 [Rattus norvegicus]

XP_002697666.2 bone morphogenetic protein 6 isoform X1 [Bos taurus]

NP_001161473.1 bone morphogenetic protein 6 precursor [Sus scrofa]

XP_038302254.1 LOW QUALITY PROTEIN: bone morphogenetic protein 6 [Canis lupus familiaris]

XP_023480199.1 bone morphogenetic protein 6 [Equus caballus]

XP_027814245.1 bone morphogenetic protein 6 isoform X1 [Ovis aries]

XP_003950760.2 bone morphogenetic protein 6 [Pan troglodytes]

XP_001085364.1 bone morphogenetic protein 6 [Macaca mulatta]

XP_023109461.1 bone morphogenetic protein 6 [Felis catus]

XP_017894411.1 PREDICTED: bone morphogenetic protein 6 isoform X1 [Capra hircus]

XP_030868423.1 bone morphogenetic protein 6 [Gorilla gorilla gorilla]

XP_024104721.1 bone morphogenetic protein 6 [Pongo abelii]

XP_031997037.1 bone morphogenetic protein 6 [Hylobates moloch]

[NP_001709.1](https://www.ncbi.nlm.nih.gov/protein/NP_001709.1?report=genbank&log$=protalign&blast_rank=0&RID=0) 1 MPGLGRRAQWLCWWWGLLCSCCGPPPLRPPLPAAAAA-AaGGQLLGDGGSPGRTEQPPPSP QSSS-GFLYRRLKTQ 74

[NP_031582.1](https://www.ncbi.nlm.nih.gov/protein/NP_031582.1?report=genbank&log$=protalign&blast_rank=1&RID=0) 1 MPGLGRRAQWLCWWWGLLCSC-GPPPLRPPLPVAAAA-A-GGQLLGAGGSPVRAEQPPPQ- SSSS-GFLYRRLKTH 71

[XP_038951315.1](https://www.ncbi.nlm.nih.gov/protein/XP_038951315.1?report=genbank&log$=protalign&blast_rank=2&RID=0) 1 MPGLGRRAQWLCWWWGLLCSC-GPPPLRPPLPVAAAA-A-GGQLLGAGGSPVRAEQPPPQ- SSSS-GFLYRRLKTH 71

[XP_002697666.2](https://www.ncbi.nlm.nih.gov/protein/XP_002697666.2?report=genbank&log$=protalign&blast_rank=3&RID=0) 1 M--LGRTAQWLCWWWGLLCSFCGPPPL----PAAAAA---GGALLGDGGSPGHAERPPPPQ TSSS-GFLYRRLKTH 66

[NP_001161473.1](https://www.ncbi.nlm.nih.gov/protein/NP_001161473.1?report=genbank&log$=protalign&blast_rank=4&RID=0) 1 MPGLGRRAQWLCWWWGLLCSCCGPPSLRPPLPAAAAA-A-GGALLGDGGSPGHAEQPPPPQ TSSS-GFLYRRLKTH 73

[XP_038302254.1](https://www.ncbi.nlm.nih.gov/protein/XP_038302254.1?report=genbank&log$=protalign&blast_rank=5&RID=0) 1 M--LGPRAPWLCWWWGLLCSCCGPPPL-PPAAAAAAAaA-GGALLGDGGSPGHAEPTPPPP[14]SSSS-GFLYRRLKTH 85

[XP_023480199.1](https://www.ncbi.nlm.nih.gov/protein/XP_023480199.1?report=genbank&log$=protalign&blast_rank=6&RID=0) 1 MPGLARRAQWLCWWWGLLCSCCGPPPLRPSLPAAAAT-G-GGALLGDGGSPGHAEQPPPPP QSSSsGFLYRRLKTH 74

[XP_027814245.1](https://www.ncbi.nlm.nih.gov/protein/XP_027814245.1?report=genbank&log$=protalign&blast_rank=7&RID=0) 1 M--LGRTAQWLCWWWGLLCSFCGPPPL----PAAAAA---GGALLGDGGSPGHAERPPPPQ TSSS-GFLYRRLKTH 66

[XP_003950760.2](https://www.ncbi.nlm.nih.gov/protein/XP_003950760.2?report=genbank&log$=protalign&blast_rank=8&RID=0) 1 MPGLGRRAQWLCWWWGLLCSCCGPPPLRPPLPAAAAA-AaGGQLLGDGGSPGRTEQPPPSP QSSS-GFLYRRLKTQ 74

[XP_001085364.1](https://www.ncbi.nlm.nih.gov/protein/XP_001085364.1?report=genbank&log$=protalign&blast_rank=9&RID=0) 1 MPGLGRRAQWLCWWWGLLCSCCGPPPLRPPLPAAAAA-AaGGQLLGDGGSPGRTEQPPPSP QSSS-GFLYRRLKTH 74

[XP_023109461.1](https://www.ncbi.nlm.nih.gov/protein/XP_023109461.1?report=genbank&log$=protalign&blast_rank=10&RID=0) 1 M--LGRRAQWLCWWWGLLCSCCGPPPLRPPLPAAAAAaG-GGALLGDGGSPGHAEQPPPPP QSSS-GFLYRRLKTH 72

[XP_017894411.1](https://www.ncbi.nlm.nih.gov/protein/XP_017894411.1?report=genbank&log$=protalign&blast_rank=11&RID=0) 1 M--LGRTAQWLCWWWGLLCSFCGPPPL----PAAAAA--aGGALLGDGGSPGHAERPPPPQ TSSS-GFLYRRLKTH 67

[XP_030868423.1](https://www.ncbi.nlm.nih.gov/protein/XP_030868423.1?report=genbank&log$=protalign&blast_rank=12&RID=0) 1 MPGLGRRAQWLCWWWGLLCSCCGPPPLRPPLPATAAA-AaGGQLLGDGGSPGRTEQPSPSP QSSS-GFLYRRLKTQ 74

[XP_024104721.1](https://www.ncbi.nlm.nih.gov/protein/XP_024104721.1?report=genbank&log$=protalign&blast_rank=13&RID=0) 1 MPGLGRRAQWLCWWWGLLCSCCGPPPLRPPLPAAAAA-AaGGQLLGDGGSPGQTEQPPPSP QSSS-GFLYRRLKTQ 74

[XP_031997037.1](https://www.ncbi.nlm.nih.gov/protein/XP_031997037.1?report=genbank&log$=protalign&blast_rank=14&RID=0) 1 MPGLGRRAQWLCWWWGLLCSCCGPPPLRPPLPAAAAA-AaGGQLLGDGGSPGRTEQPPPSP QSSS-GFLYRRLKTQ 74

[NP_001709.1](https://www.ncbi.nlm.nih.gov/protein/NP_001709.1?report=genbank&log$=protalign&blast_rank=0&RID=0) 75 EKREMQKEILSVLGLPHRPRPLHGLQQPQPPAL-R---QQEeQQQQQq--LP-RGEPPPGRLKSAPLFMLDLYNALSADN 147

[NP_031582.1](https://www.ncbi.nlm.nih.gov/protein/NP_031582.1?report=genbank&log$=protalign&blast_rank=1&RID=0) 72 EKREMQKEILSVLGLPHRPRPLHGLQQPQPPVL-PpqqQQQ-QQQQQ---TA-REEPPPGRLKSAPLFMLDLYNALSNDD 145

[XP_038951315.1](https://www.ncbi.nlm.nih.gov/protein/XP_038951315.1?report=genbank&log$=protalign&blast_rank=2&RID=0) 72 EKREMQKEILSVLGLPHRPRPLHGLQQPQSPVL-P----QQ-QQSQQ---TA-REEPPPGRLKSAPLFMLDLYNSLSKDD 141

[XP_002697666.2](https://www.ncbi.nlm.nih.gov/protein/XP_002697666.2?report=genbank&log$=protalign&blast_rank=3&RID=0) 67 EKREMQKEILSVLGLPHRPRPLHGL--PQPPVF-P---QQQ-----Q----PaRGEPPPGRLKSAPLFMLDLYNALSADD 131

[NP_001161473.1](https://www.ncbi.nlm.nih.gov/protein/NP_001161473.1?report=genbank&log$=protalign&blast_rank=4&RID=0) 74 EKREMQKEILSVLGLPHRPRPLHGLQQPQPPAL-P---QQQ-QQQTH-------GEPPPGRLKSAPLFMLDLYNALSADD 141

[XP_038302254.1](https://www.ncbi.nlm.nih.gov/protein/XP_038302254.1?report=genbank&log$=protalign&blast_rank=5&RID=0) 86 EKREMQKEILSVLGLPHRPRPLHGLPPPQPAAF-P---QQQ----------P-RGEPPPGRLKSAPLFMLDLYNALAAAD 150

[XP_023480199.1](https://www.ncbi.nlm.nih.gov/protein/XP_023480199.1?report=genbank&log$=protalign&blast_rank=6&RID=0) 75 EKREMQKEILSVLGLPHRPRPLHGLQQLQPPAL-P---QQQ----------P-RGEPPPGRLKSAPLFMLDLYNALAAAD 139

[XP_027814245.1](https://www.ncbi.nlm.nih.gov/protein/XP_027814245.1?report=genbank&log$=protalign&blast_rank=7&RID=0) 67 EKREMQREILSVLGLPHRPRPLHGL--PQPPVF-P---QQQ----------PaRGEPPPGRLKSAPLFMLDLYNALSADD 130

[XP_003950760.2](https://www.ncbi.nlm.nih.gov/protein/XP_003950760.2?report=genbank&log$=protalign&blast_rank=8&RID=0) 75 EKREMQKEILSVLGLPHRPRPLHGLQQPQPPAL-R---QQEeQQQQQ---LP-RGEPPPGRLKSAPLFMLDLYNALSADN 146

[XP_001085364.1](https://www.ncbi.nlm.nih.gov/protein/XP_001085364.1?report=genbank&log$=protalign&blast_rank=9&RID=0) 75 EKREMQKEILSVLGLPHRPRPLHGLQQPQPPAL-P---QQQ-QQQQQ---PP-RGEPPPGQLKSAPLFMLDLYNALSADD 145

[XP_023109461.1](https://www.ncbi.nlm.nih.gov/protein/XP_023109461.1?report=genbank&log$=protalign&blast_rank=10&RID=0) 73 EKREMQKEILSVLGLPHRPRPLHGLQQPQPAAL-P---QEQ----------P-RGEPPPGRLKSAPLFMLDLYNALAADD 137

[XP_017894411.1](https://www.ncbi.nlm.nih.gov/protein/XP_017894411.1?report=genbank&log$=protalign&blast_rank=11&RID=0) 68 EKREMQREILSVLGLPHRPRPLHGL--PQPPVF-P---QQQ----------PaRGEPPPGRLKSAPLFMLDLYNALSADD 131

[XP_030868423.1](https://www.ncbi.nlm.nih.gov/protein/XP_030868423.1?report=genbank&log$=protalign&blast_rank=12&RID=0) 75 EKREMQKEILSVLGLPHRPRPLHGLQQPQPPAL-R---QQEeQQQQQqqqLP-RGEPPPGRLKSAPLFMLDLYNALSADN 149

[XP_024104721.1](https://www.ncbi.nlm.nih.gov/protein/XP_024104721.1?report=genbank&log$=protalign&blast_rank=13&RID=0) 75 EKREMQKEILSVLGLPHRPRPLHGLQQPQPPAL-R---QQEeQQQQQ---LP-RGEPPPGRLKSAPLFMLDLYNALSADN 146

[XP_031997037.1](https://www.ncbi.nlm.nih.gov/protein/XP_031997037.1?report=genbank&log$=protalign&blast_rank=14&RID=0) 75 EKREMQKEILSVLGLPHRPRPLHGLQEPQPPALpQ---QQQqQQQQQ---LP-RGEPPPGRLKSAPLFMLDLYNALSTDD 147

[NP_001709.1](https://www.ncbi.nlm.nih.gov/protein/NP_001709.1?report=genbank&log$=protalign&blast_rank=0&RID=0) 148 DEDGASEGERQQSWPHEAASSSQRRQPPPGAAHPLNRKSLLAPGSGSGGA S-PLTSAQDSAFLNDADMVMSFVNLVE 223

[NP_031582.1](https://www.ncbi.nlm.nih.gov/protein/NP_031582.1?report=genbank&log$=protalign&blast_rank=1&RID=0) 146 EEDGASEGVGQEPGSHGGASSSQLRQPSPGAAHSLNRKSLLAPGPG-GGA S-PLTSAQDSAFLNDADMVMSFVNLVE 220

[XP_038951315.1](https://www.ncbi.nlm.nih.gov/protein/XP_038951315.1?report=genbank&log$=protalign&blast_rank=2&RID=0) 142 EEDGVSEGEGLEPESHGRASSSQLKQPSPGAAHSLNRKSLLAPGPG-GSA S-PLTSAQDSAFLNDADMVMSFVNLVE 216

[XP_002697666.2](https://www.ncbi.nlm.nih.gov/protein/XP_002697666.2?report=genbank&log$=protalign&blast_rank=3&RID=0) 132 EEDGASDEERRQPGQRGGADASQPRSPSSGAAHPLGSGSLLATGPGGG-- AsPLTSAQDSAFLNDADMVMSFVNLVE 206

[NP_001161473.1](https://www.ncbi.nlm.nih.gov/protein/NP_001161473.1?report=genbank&log$=protalign&blast_rank=4&RID=0) 142 EEDLASDKEMGQPGLRGGTDSSPPRQPPPRAAHPLGSRSLLAAGPG-GSA S-PLTSAQESAFLNDADMVMSFVNLVE 216

[XP_038302254.1](https://www.ncbi.nlm.nih.gov/protein/XP_038302254.1?report=genbank&log$=protalign&blast_rank=5&RID=0) 151 DEGWEAGEQRRPRGPRGGAGSSPPRRPPPGAAPPVGGRSLVAAGPGGGGG[4]AsALTSAQDSAFLNDADMVMSFVNLVE 231

[XP_023480199.1](https://www.ncbi.nlm.nih.gov/protein/XP_023480199.1?report=genbank&log$=protalign&blast_rank=6&RID=0) 140 DEDGPSDEERRPPAPRGGAGSPQPGQPPPGAAHPLNRKSLLAPGPGGGGA AtPLTSAQDSAFLNDADMVMSFVNLVE 216

[XP_027814245.1](https://www.ncbi.nlm.nih.gov/protein/XP_027814245.1?report=genbank&log$=protalign&blast_rank=7&RID=0) 131 EEDGASDEERRQPGPRGGAGASQPRSPSPGAAHPLGSGSLLATGPGGG-- AsPLTSAQDSAFLNDADMVMSFVNLVE 205

[XP_003950760.2](https://www.ncbi.nlm.nih.gov/protein/XP_003950760.2?report=genbank&log$=protalign&blast_rank=8&RID=0) 147 DEDGASEGERQQSWPHEAASSSQRRQPPPGAAHPLNRKSLLAPGSGSGGA S-PLTSAQDSAFLNDADMVMSFVNLVE 222

[XP_001085364.1](https://www.ncbi.nlm.nih.gov/protein/XP_001085364.1?report=genbank&log$=protalign&blast_rank=9&RID=0) 146 EEDGASEGERQQPWPHEGASSSQPRQPAPGAAHPLNRKSLLAPGPGSGGA S-PLTSAQDSAFLNDADMVMSFVNLVE 221

[XP_023109461.1](https://www.ncbi.nlm.nih.gov/protein/XP_023109461.1?report=genbank&log$=protalign&blast_rank=10&RID=0) 138 DEDWASDEERRQPGPRGGAGSSQPRQPPPGAAHPVNGKSLLASGPGGG-- TsPLTSAQDSAFLNDADMVMSFVNLVE 212

[XP_017894411.1](https://www.ncbi.nlm.nih.gov/protein/XP_017894411.1?report=genbank&log$=protalign&blast_rank=11&RID=0) 132 EEDGASDEERRQPGPRGGAGASQSRSPSPGAAHPLGSGSLLATGPGGG-- AsPLTSAQDSAFLNDADMVMSFVNLVE 206

[XP_030868423.1](https://www.ncbi.nlm.nih.gov/protein/XP_030868423.1?report=genbank&log$=protalign&blast_rank=12&RID=0) 150 DEDGASEGERQQSWPHEAASSSQRRQPPPGAAHPLNRKSLLAPGSGSGGA S-PLTSAQDSAFLSDADMVMSFVNLVE 225

[XP_024104721.1](https://www.ncbi.nlm.nih.gov/protein/XP_024104721.1?report=genbank&log$=protalign&blast_rank=13&RID=0) 147 DEDGASEGERQQSWPHEAASSSQRRQPPPGAAHPLNRKSLLAPGPGSGGA S-PLASAQDSAFLNDADMVMSFVNLVE 222

[XP_031997037.1](https://www.ncbi.nlm.nih.gov/protein/XP_031997037.1?report=genbank&log$=protalign&blast_rank=14&RID=0) 148 DEDGASEEKKQQSWPREAASSSQRRQPPPGAVHPLNRKSLLAPGPGSGGA S-PLTSAQDSAFLNDADMVMSFVNLVE 223

[NP_001709.1](https://www.ncbi.nlm.nih.gov/protein/NP_001709.1?report=genbank&log$=protalign&blast_rank=0&RID=0) 224 YDKEFSPRQRHHKEFKFNLSQIPEGEVVTAAEFRIYKDCVMGSFKNQTFLISIYQVLQEHQHRDSDLFLLDTRVVWASEE 303

[NP_031582.1](https://www.ncbi.nlm.nih.gov/protein/NP_031582.1?report=genbank&log$=protalign&blast_rank=1&RID=0) 221 YDKEFSPHQRHHKEFKFNLSQIPEGEAVTAAEFRVYKDCVVGSFKNQTFLISIYQVLQEHQHRDSDLFLLDTRVVWASEE 300

[XP_038951315.1](https://www.ncbi.nlm.nih.gov/protein/XP_038951315.1?report=genbank&log$=protalign&blast_rank=2&RID=0) 217 YDKEFSPRQRHHKEFKFNLSQIPEGEAVTAAEFRVYKDCVVGSFKNQTFLISIYQVLQEHQHRDSDLFLLDTRVVWASEE 296

[XP_002697666.2](https://www.ncbi.nlm.nih.gov/protein/XP_002697666.2?report=genbank&log$=protalign&blast_rank=3&RID=0) 207 YDKEFSPRQRHHKEFKFNLSQIPEGEAVTAAEFRIYKDCVVGSFKNQTFLISIYQVLQEHQHRDSDLFLLGTRAVWASEA 286

[NP_001161473.1](https://www.ncbi.nlm.nih.gov/protein/NP_001161473.1?report=genbank&log$=protalign&blast_rank=4&RID=0) 217 YDKELSPRQRHHKEFKFNLSQVPEGEAVTAAEFRIYKDCVVGSFKNQTFLISIYQVLQQRQHRDSDLFLLDTRVVWASEE 296

[XP_038302254.1](https://www.ncbi.nlm.nih.gov/protein/XP_038302254.1?report=genbank&log$=protalign&blast_rank=5&RID=0) 232 YEKEFSPGQRHHKEFKFNLSQIPEGEAVTAAEFRIYKDCVVGSFKNQTFLISIYQVLQEHQHRDSDLFLLDTRVVWASEE 311

[XP_023480199.1](https://www.ncbi.nlm.nih.gov/protein/XP_023480199.1?report=genbank&log$=protalign&blast_rank=6&RID=0) 217 YDKEFSPRQRHHKEFKFNLSQIPEGEAVTAAEFRIYKDCVVGSFKNQTFLISIYQVLQEHQHRDSDLFLLDTRMVWASEE 296

[XP_027814245.1](https://www.ncbi.nlm.nih.gov/protein/XP_027814245.1?report=genbank&log$=protalign&blast_rank=7&RID=0) 206 YDKEFSPRQRHHKEFKFNLSQIPEGEAVTAAEFRIYKDCVVGSFKNQTFLISIYQVLQEHQHRDSDLFLLGTRAVWASEA 285

[XP_003950760.2](https://www.ncbi.nlm.nih.gov/protein/XP_003950760.2?report=genbank&log$=protalign&blast_rank=8&RID=0) 223 YDKEFSPRQRHHKEFKFNLSQIPEGEAVTAAEFRIYKDCVMGSFKNQTFLISIYQVLQEHQHRDSDLFLLDTRVVWASEE 302

[XP_001085364.1](https://www.ncbi.nlm.nih.gov/protein/XP_001085364.1?report=genbank&log$=protalign&blast_rank=9&RID=0) 222 YDKEFSPRQRHHKEFKFNLSQIPEGEAVTAAEFRIYKDCVVGSFKNQTFLISIYQVLQEHQHRDSDLFLLDTRVVWASEE 301

[XP_023109461.1](https://www.ncbi.nlm.nih.gov/protein/XP_023109461.1?report=genbank&log$=protalign&blast_rank=10&RID=0) 213 HEKEFSPGQRYHKEFKFNLSQIPEGEAVTAAEFRIYKDCVVGSFKNQTFLISIYQVLQEHQHRDSDLFLLDTRVVWASEE 292

[XP_017894411.1](https://www.ncbi.nlm.nih.gov/protein/XP_017894411.1?report=genbank&log$=protalign&blast_rank=11&RID=0) 207 YDKEFSPRQRHHKEFKFNLSQIPEGEAVTAAEFRIYKDCVVGSFKNQTFLISIYQVLQEHQHRDSDLFLLGTRAVWASEA 286

[XP_030868423.1](https://www.ncbi.nlm.nih.gov/protein/XP_030868423.1?report=genbank&log$=protalign&blast_rank=12&RID=0) 226 YDKEFSPRQQHHKEFKFNLSQIPEGEAVTAAEFRIYKDCVMGSFKNQTFLISIYQVLQEHQHRDSDLFLLDTRVVWASEE 305

[XP_024104721.1](https://www.ncbi.nlm.nih.gov/protein/XP_024104721.1?report=genbank&log$=protalign&blast_rank=13&RID=0) 223 YDKEFSPRQRHHKEFKFNLSQIPEGEAVTAAEFRIYKDCVMGSFKNQTFLISIYQVLQEHQHRDSDLFLLDTRVVWASEE 302

[XP_031997037.1](https://www.ncbi.nlm.nih.gov/protein/XP_031997037.1?report=genbank&log$=protalign&blast_rank=14&RID=0) 224 YDKEFSPRQRHHKEFKFNLSQIPEGEAVTAAEFRIYKDCVMGSFKNQTFLISIYQVLQEHQHRDSDLCLLDTRVVWASEE 303

[NP_001709.1](https://www.ncbi.nlm.nih.gov/protein/NP_001709.1?report=genbank&log$=protalign&blast_rank=0&RID=0) 304 GWLEFDITATSNLWVVTPQHNMGLQLSVVTRDGVHVHPRAAGLVGRDGPYDKQPFMVAFFKVSEVHVRTTRSASSRRRQQ 383

[NP_031582.1](https://www.ncbi.nlm.nih.gov/protein/NP_031582.1?report=genbank&log$=protalign&blast_rank=1&RID=0) 301 GWLEFDITATSNLWVVTPQHNMGLQLSVVTRDGLHVNPRAAGLVGRDGPYDKQPFMVAFFKVSEVHVRTTRSASSRRRQQ 380

[XP_038951315.1](https://www.ncbi.nlm.nih.gov/protein/XP_038951315.1?report=genbank&log$=protalign&blast_rank=2&RID=0) 297 GWLEFDITATSNLWVVTPQHNMGLQLSVVTRDGLHINPRAAGLVGRDGPYDKQPFMVAFFKVSEVHVRTTRSASSRRRQQ 376

[XP_002697666.2](https://www.ncbi.nlm.nih.gov/protein/XP_002697666.2?report=genbank&log$=protalign&blast_rank=3&RID=0) 287 GWLEFDITATSNLWVLTPQHNMGLQLSVVTRDGLSISPGAAGLVGRDGPYDKQPFMVAFFKASEVHVRSARSAPGRRRQQ 366

[NP_001161473.1](https://www.ncbi.nlm.nih.gov/protein/NP_001161473.1?report=genbank&log$=protalign&blast_rank=4&RID=0) 297 GWLEFDITATSNLWVLTPQHNLGLQLGVVTQDGLSISPRAAGLVGRDGPYDKQPFMVAFFKVSEVHVRTARSATGRRRQQ 376

[XP_038302254.1](https://www.ncbi.nlm.nih.gov/protein/XP_038302254.1?report=genbank&log$=protalign&blast_rank=5&RID=0) 312 GWLEFDITATSNLWVVTPQHNMGLQLSVVTRDGLPINPRAAGLVGRDGPYDKQPFMVAFFKVSEVHVRTTRSAPGRRRQQ 391

[XP_023480199.1](https://www.ncbi.nlm.nih.gov/protein/XP_023480199.1?report=genbank&log$=protalign&blast_rank=6&RID=0) 297 GWLEFDITATSNLWVVTPQHNMGLQLSVVTRDGLSINPRAAGLVGRDGPYDKQPFMVAFFKVSEVHVRTTRSATGRRRQQ 376

[XP_027814245.1](https://www.ncbi.nlm.nih.gov/protein/XP_027814245.1?report=genbank&log$=protalign&blast_rank=7&RID=0) 286 GWLEFDITATSNLWVLTPQHNMGLQLSVVTRDGLSISPGAAGLVGRDGPYDKQPFMVAFFKASEVHVRSARSAPGRRRQQ 365

[XP_003950760.2](https://www.ncbi.nlm.nih.gov/protein/XP_003950760.2?report=genbank&log$=protalign&blast_rank=8&RID=0) 303 GWLEFDITATSNLWVVTPQHNMGLQLSVVTRDGVHVHPRAAGLVGRDGPYDKQPFMVAFFKVSEVHVRTTRSASSRRRQQ 382

[XP_001085364.1](https://www.ncbi.nlm.nih.gov/protein/XP_001085364.1?report=genbank&log$=protalign&blast_rank=9&RID=0) 302 GWLEFDITATSNLWVVTPQHNMGLQLSVVTRDGVHIHPRAAGLVGRDGPYDKQPFMVAFFKVSEVHVRTTRSASGRRRQQ 381

[XP_023109461.1](https://www.ncbi.nlm.nih.gov/protein/XP_023109461.1?report=genbank&log$=protalign&blast_rank=10&RID=0) 293 GWLEFDITATSNLWVVTPQHNMGLQLSVVTRDGLNINPRAAGLVGRDGPYDKQPFMVAFFKVSEVHVRTTRSAPGRRRQQ 372

[XP_017894411.1](https://www.ncbi.nlm.nih.gov/protein/XP_017894411.1?report=genbank&log$=protalign&blast_rank=11&RID=0) 287 GWLEFDITATSNLWVLTPQHNMGLQLSVVTRDGLSISPGAAGLVGRDGPYDKQPFMVAFFKASEVHVRSARSAPGRRRQQ 366

[XP_030868423.1](https://www.ncbi.nlm.nih.gov/protein/XP_030868423.1?report=genbank&log$=protalign&blast_rank=12&RID=0) 306 GWLEFDITATSNLWVVTPQHNMGLQLSVVTRDGVHVHPRAAGLVGRDGPYDKQPFMVAFFKVSEVHVRTTRSASSRRRQQ 385

[XP_024104721.1](https://www.ncbi.nlm.nih.gov/protein/XP_024104721.1?report=genbank&log$=protalign&blast_rank=13&RID=0) 303 GWLEFDITATSNLWVVTPQHNMGLQLSVVTRDGVHIHPRAAGLVGRDGPYDKQPFMVAFFKVSEVHVRTTRSASSRRRQQ 382

[XP_031997037.1](https://www.ncbi.nlm.nih.gov/protein/XP_031997037.1?report=genbank&log$=protalign&blast_rank=14&RID=0) 304 GWLEFDITATSNLWVVTPQHNMGLQLSVVTRNGVHVHPRAAGLVGRDGPYDKQPFMVAFFKVSEVHVRATRSASSRRRQQ 383

[NP_001709.1](https://www.ncbi.nlm.nih.gov/protein/NP_001709.1?report=genbank&log$=protalign&blast_rank=0&RID=0) 384 SRNRSTQSQDVARVSSAS-DYNSSELKTACRKHELYVSFQDLGWQDWIIAPKGYAANYCDGECSFPLNAHMNATNHAIVQ 462

[NP_031582.1](https://www.ncbi.nlm.nih.gov/protein/NP_031582.1?report=genbank&log$=protalign&blast_rank=1&RID=0) 381 SRNRSTQSQDVSRGSGSS-DYNGSELKTACKKHELYVSFQDLGWQDWIIAPKGYAANYCDGECSFPLNAHMNATNHAIVQ 459

[XP_038951315.1](https://www.ncbi.nlm.nih.gov/protein/XP_038951315.1?report=genbank&log$=protalign&blast_rank=2&RID=0) 377 SRNRSTQSQDVSRGSSAS-DYNSSELKTACKKHELYVSFQDLGWQDWIIAPKGYAANYCDGECSFPLNAHMNATNHAIVQ 455

[XP_002697666.2](https://www.ncbi.nlm.nih.gov/protein/XP_002697666.2?report=genbank&log$=protalign&blast_rank=3&RID=0) 367 ARNRSTPAQDVSRASSASaDYNSSELKTACRKHELYVSFQDLGWQDWIIAPKGYAANYCDGECSFPLNAHMNATNHAIVQ 446

[NP_001161473.1](https://www.ncbi.nlm.nih.gov/protein/NP_001161473.1?report=genbank&log$=protalign&blast_rank=4&RID=0) 377 SRNRSTQAQDVSRASSAS-DYNSSELKTACRKHELYVSFQDLGWQDWIIAPKGYAANYCDGECSFPLNAHMNATNHAIVQ 455

[XP_038302254.1](https://www.ncbi.nlm.nih.gov/protein/XP_038302254.1?report=genbank&log$=protalign&blast_rank=5&RID=0) 392 SRNRSTQSQDVSRVSSAS-DYNSSELKTACRKHELYVSFQDLGWQDWIIAPKGYAANYCDGECSFPLNAHMNATNHAIVQ 470

[XP_023480199.1](https://www.ncbi.nlm.nih.gov/protein/XP_023480199.1?report=genbank&log$=protalign&blast_rank=6&RID=0) 377 SRNRSTQSQDVSRVSSAS-DYNSSELKTACRKHELYVSFQDLGWQDWIIAPKGYAANYCDGECSFPLNAHMNATNHAIVQ 455

[XP_027814245.1](https://www.ncbi.nlm.nih.gov/protein/XP_027814245.1?report=genbank&log$=protalign&blast_rank=7&RID=0) 366 ARNRSTPAQDVSRASSASaDYNSSELKTACRKHELYVSFQDLGWQDWIIAPKGYAANYCDGECSFPLNAHMNATNHAIVQ 445

[XP_003950760.2](https://www.ncbi.nlm.nih.gov/protein/XP_003950760.2?report=genbank&log$=protalign&blast_rank=8&RID=0) 383 SRNRSTQSQDVARVSSAS-DYNSSELKTACRKHELYVSFQDLGWQDWIIAPKGYAANYCDGECSFPLNAHMNATNHAIVQ 461

[XP_001085364.1](https://www.ncbi.nlm.nih.gov/protein/XP_001085364.1?report=genbank&log$=protalign&blast_rank=9&RID=0) 382 SRNRSTQSQDVARVSSAS-DYNSSELKTACRKHELYVSFQDLGWQDWIIAPKGYAANYCDGECSFPLNAHMNATNHAIVQ 460

[XP_023109461.1](https://www.ncbi.nlm.nih.gov/protein/XP_023109461.1?report=genbank&log$=protalign&blast_rank=10&RID=0) 373 SRNRSTQSQDVSRVSSAS-DYNSSELKTACRKHELYVSFQDLGWQDWIIAPKGYAANYCDGECSFPLNAHMNATNHAIVQ 451

[XP_017894411.1](https://www.ncbi.nlm.nih.gov/protein/XP_017894411.1?report=genbank&log$=protalign&blast_rank=11&RID=0) 367 ARNRSTPAQDVSRASSASaDYNSSELKTACRKHELYVSFQDLGWQDWIIAPKGYAANYCDGECSFPLNAHMNATNHAIVQ 446

[XP_030868423.1](https://www.ncbi.nlm.nih.gov/protein/XP_030868423.1?report=genbank&log$=protalign&blast_rank=12&RID=0) 386 SRNRSTQSQDVARVSSAS-DYNSSELKTACRKHELYVSFQDLGWQDWIIAPKGYAANYCDGECSFPLNAHMNATNHAIVQ 464

[XP_024104721.1](https://www.ncbi.nlm.nih.gov/protein/XP_024104721.1?report=genbank&log$=protalign&blast_rank=13&RID=0) 383 SRNRSTQSQDVARVSSAS-DYNSSELKTACRKHELYVSFQDLGWQDWIIAPKGYAANYCDGECSFPLNAHMNATNHAIVQ 461

[XP_031997037.1](https://www.ncbi.nlm.nih.gov/protein/XP_031997037.1?report=genbank&log$=protalign&blast_rank=14&RID=0) 384 SRNRSTQSQDVARVSSAS-DYNSSELKTACRKHELYVSFQDLGWQDWIIAPKGYAANYCDGECSFPLNAHMNATNHAIVQ 462

[NP_001709.1](https://www.ncbi.nlm.nih.gov/protein/NP_001709.1?report=genbank&log$=protalign&blast_rank=0&RID=0) 463 TLV HLMNPEYVPKPCCAPTKLNAISVLYFDDNSNVILKKYRNMVVRACGCH 513

[NP_031582.1](https://www.ncbi.nlm.nih.gov/protein/NP_031582.1?report=genbank&log$=protalign&blast_rank=1&RID=0) 460 TLV HLMNPEYVPKPCCAPTKLNAISVLYFDDNSNVILKKYRNMVVRACGCH 510

[XP_038951315.1](https://www.ncbi.nlm.nih.gov/protein/XP_038951315.1?report=genbank&log$=protalign&blast_rank=2&RID=0) 456 TLV[29]HLMNPEYVPKPCCAPTKLNAISVLYFDDNSNVILKKYRNMVVRACGCH 535

[XP_002697666.2](https://www.ncbi.nlm.nih.gov/protein/XP_002697666.2?report=genbank&log$=protalign&blast_rank=3&RID=0) 447 TLV HLMNPEYVPKPCCAPTKLNAISVLYFDDNSNVILKKYRNMVVRACGCH 497

[NP_001161473.1](https://www.ncbi.nlm.nih.gov/protein/NP_001161473.1?report=genbank&log$=protalign&blast_rank=4&RID=0) 456 TLV HLMNPEYVPKPCCAPTKLNAISVLYFDDNSNVILKKYRNMVVRACGCH 506

[XP_038302254.1](https://www.ncbi.nlm.nih.gov/protein/XP_038302254.1?report=genbank&log$=protalign&blast_rank=5&RID=0) 471 TLV HLMNPEYVPKPCCAPTKLNAISVLYFDDNSNVILKKYRNMVVRACGCH 521

[XP_023480199.1](https://www.ncbi.nlm.nih.gov/protein/XP_023480199.1?report=genbank&log$=protalign&blast_rank=6&RID=0) 456 TLV HLMNPEYVPKPCCAPTKLNAISVLYFDDNSNVILKKYRNMVVRACGCH 506

[XP_027814245.1](https://www.ncbi.nlm.nih.gov/protein/XP_027814245.1?report=genbank&log$=protalign&blast_rank=7&RID=0) 446 TLV HLMNPEYVPKPCCAPTKLNAISVLYFDDNSNVILKKYRNMVVRACGCH 496

[XP_003950760.2](https://www.ncbi.nlm.nih.gov/protein/XP_003950760.2?report=genbank&log$=protalign&blast_rank=8&RID=0) 462 TLV HLMNPEYVPKPCCAPTKLNAISVLYFDDNSNVILKKYRNMVVRACGCH 512

[XP_001085364.1](https://www.ncbi.nlm.nih.gov/protein/XP_001085364.1?report=genbank&log$=protalign&blast_rank=9&RID=0) 461 TLV HLMNPEYVPKPCCAPTKLNAISVLYFDDNSNVILKKYRNMVVRACGCH 511

[XP_023109461.1](https://www.ncbi.nlm.nih.gov/protein/XP_023109461.1?report=genbank&log$=protalign&blast_rank=10&RID=0) 452 TLV HLMNPEYVPKPCCAPTKLNAISVLYFDDNSNVILKKYRNMVVRACGCH 502

[XP_017894411.1](https://www.ncbi.nlm.nih.gov/protein/XP_017894411.1?report=genbank&log$=protalign&blast_rank=11&RID=0) 447 TLV HLMNPEYVPKPCCAPTKLNAISVLYFDDNSNVILKKYRNMVVRACGCH 497

[XP_030868423.1](https://www.ncbi.nlm.nih.gov/protein/XP_030868423.1?report=genbank&log$=protalign&blast_rank=12&RID=0) 465 TLV HLMNPEYVPKPCCAPTKLNAISVLYFDDNSNVILKKYRNMVVRACGCH 515

[XP_024104721.1](https://www.ncbi.nlm.nih.gov/protein/XP_024104721.1?report=genbank&log$=protalign&blast_rank=13&RID=0) 462 TLV HLMNPEYVPKPCCAPTKLNAISVLYFDDNSNVILKKYRNMVVRACGCH 512

[XP_031997037.1](https://www.ncbi.nlm.nih.gov/protein/XP_031997037.1?report=genbank&log$=protalign&blast_rank=14&RID=0) 463 TLV HLMNPEYVPKPCCAPTKLNAISVLYFDDNSNVILKKYRNMVVRACGCH 513

GDF2/BMP9

NP_057288.1 growth/differentiation factor 2 preproprotein [Homo sapiens]

NP_062379.3 growth/differentiation factor 2 preproprotein [Mus musculus]

NP_001099566.1 growth/differentiation factor 2 precursor [Rattus norvegicus]

NP_001179349.1 growth/differentiation factor 2 precursor [Bos taurus]

XP_003133152.2 growth/differentiation factor 2 [Sus scrofa]

XP_853886.2 growth/differentiation factor 2 isoform X1 [Canis lupus familiaris]

XP_001500704.1 growth/differentiation factor 2 [Equus caballus]

XP_004021599.3 growth/differentiation factor 2 [Ovis aries]

XP_507775.4 growth/differentiation factor 2 [Pan troglodytes]

XP_001109523.2 growth/differentiation factor 2 [Macaca mulatta]

XP_003994191.1 growth/differentiation factor 2 [Felis catus]

XP_005699391.1 PREDICTED: growth/differentiation factor 2 [Capra hircus]

XP_004049419.1 growth/differentiation factor 2 [Gorilla gorilla gorilla]

XP_002820746.1 growth/differentiation factor 2 [Pongo abelii]

XP_032026853.1 growth/differentiation factor 2 [Hylobates moloch]

[NP_057288.1](https://www.ncbi.nlm.nih.gov/protein/NP_057288.1?report=genbank&log$=protalign&blast_rank=0&RID=0) 1 MCPGALWVALPL-LS---LLAGSLQGKPLQSWGRGSAGGNAHSPLGVPGGGLPEHTFNLKMFLENVKVDFLRSLNLSGVP 76

[NP_062379.3](https://www.ncbi.nlm.nih.gov/protein/NP_062379.3?report=genbank&log$=protalign&blast_rank=1&RID=0) 1 MSPGAFRVAL---LP-lfLLVCVTQQKPLQNWEQASPGENAHSSLGLSGAGEE-GVFDLQMFLENMKVDFLRSLNLSGIP 75

[NP_001099566.1](https://www.ncbi.nlm.nih.gov/protein/NP_001099566.1?report=genbank&log$=protalign&blast_rank=2&RID=0) 1 MSPGAFRVVL---LT-llLLVCPTQQKPLQSWGQASPGGNARSSLGLSGSREE-GVFDLKMFLENMKVDFLRSLNLSGIP 75

[NP_001179349.1](https://www.ncbi.nlm.nih.gov/protein/NP_001179349.1?report=genbank&log$=protalign&blast_rank=3&RID=0) 1 MGRGALWVALPV-LC---LLACSALGKPLENRGRPSTGADAHGLLGGPGGEQEGVTFDLRMFLENMKVDFLRNLNLSGVP 76

[XP_003133152.2](https://www.ncbi.nlm.nih.gov/protein/XP_003133152.2?report=genbank&log$=protalign&blast_rank=4&RID=0) 1 MCRGVLWVALPVaLS---LLVCSTQGKPLDSRVRASAGGDAHRLLGGAGGEQERGTFDLRMFLENMKVDFLRSLNLSGVP 77

[XP_853886.2](https://www.ncbi.nlm.nih.gov/protein/XP_853886.2?report=genbank&log$=protalign&blast_rank=5&RID=0) 1 MCGGAVGGALRA-LCalwLLGCGARGRPLEGRRRPGS-------QGAPGGPR-----DLRALPQAVQRDLLRGLNLSGVP 67

[XP_001500704.1](https://www.ncbi.nlm.nih.gov/protein/XP_001500704.1?report=genbank&log$=protalign&blast_rank=6&RID=0) 1 MCRGALRVAL---LA---LLACSAQGKPLESRGRAAGGGDAHRPRGGPGGEQEAGTFDLRMFLENMKVDFLRSLNLSGVP 74

[XP_004021599.3](https://www.ncbi.nlm.nih.gov/protein/XP_004021599.3?report=genbank&log$=protalign&blast_rank=7&RID=0) 1 MGRGALRMALPV-LS---LLACSALGKPLENRGRPSTGGDAHRLLRGPGGEQEGATFDLRMFLENMKVDFLRNLNLSGVP 76

[XP_507775.4](https://www.ncbi.nlm.nih.gov/protein/XP_507775.4?report=genbank&log$=protalign&blast_rank=8&RID=0) 1 MCPGALWVALPL-LS---LLAGSLQGKPLQSWGRGSAGGNAHSPLGVPGGGLPEHTFNLKMFLENVKVDFLRSLNLSGVP 76

[XP_001109523.2](https://www.ncbi.nlm.nih.gov/protein/XP_001109523.2?report=genbank&log$=protalign&blast_rank=9&RID=0) 1 MCPGALWVALPL-LS---LLAGSLQGKPLQSWGRGSAGGTAHNPLGVPGGELPEHTFNLKMFLENMKVDFLRSLNLSGVP 76

[XP_003994191.1](https://www.ncbi.nlm.nih.gov/protein/XP_003994191.1?report=genbank&log$=protalign&blast_rank=10&RID=0) 1 MCCGALWVALPV-LS---LLACSAQGKPLESRGRAPAGGDAHRLLGGPGGEREGGTFDLRMFLENMKVDFLRSLNLSGVP 76

[XP_005699391.1](https://www.ncbi.nlm.nih.gov/protein/XP_005699391.1?report=genbank&log$=protalign&blast_rank=11&RID=0) 1 MGRGALRMALPV-LS---LLACSALGKPLGNRGRPSTGGDAHRLLGGPGGEQEGVTFDLRMFLENMKVDFLRNLNLSGVP 76

[XP_004049419.1](https://www.ncbi.nlm.nih.gov/protein/XP_004049419.1?report=genbank&log$=protalign&blast_rank=12&RID=0) 1 MCPGALWVALPL-LS---LLAGSLQGKPLQSWGRGSAGGNAHSPLGVPGGGLPEHTFNLKMFLENVKVDFLRSLNLSGVP 76

[XP_002820746.1](https://www.ncbi.nlm.nih.gov/protein/XP_002820746.1?report=genbank&log$=protalign&blast_rank=13&RID=0) 1 MCLGALWVALPL-LS---LLAGSLQGKPLQSWGRGSAGGNAHSPLGVPGGGLPEHTFNLKMFLENVKVDFLRSLNLSGVP 76

[XP_032026853.1](https://www.ncbi.nlm.nih.gov/protein/XP_032026853.1?report=genbank&log$=protalign&blast_rank=14&RID=0) 1 MCPGALWVALPL-LS---LLAGSLQGKPLQSWGRGSAGGNAHSPLGVPGGGLPEHTFNLKMFLENMKVDFLRSLNLSGVP 76

[NP_057288.1](https://www.ncbi.nlm.nih.gov/protein/NP_057288.1?report=genbank&log$=protalign&blast_rank=0&RID=0) 77 SQDKTRVEPPQYMIDLYNRYTSDKSTTPASNIVRSFSMEDAISITATEDFPFQKHILLFNISIPRHEQITRAELRLYVSC 156

[NP_062379.3](https://www.ncbi.nlm.nih.gov/protein/NP_062379.3?report=genbank&log$=protalign&blast_rank=1&RID=0) 76 SQDKTRAEPPQYMIDLYNRYTTDKSSTPASNIVRSFSVEDAISTAATEDFPFQKHILIFNISIPRHEQITRAELRLYVSC 155

[NP_001099566.1](https://www.ncbi.nlm.nih.gov/protein/NP_001099566.1?report=genbank&log$=protalign&blast_rank=2&RID=0) 76 SQDKTRAEPPQYMIDLYNRYTTDKSSTPASNIVRSFSVEDAISTAATEDFPFQKHILIFNISIPRHEQITRAELRLYVSC 155

[NP_001179349.1](https://www.ncbi.nlm.nih.gov/protein/NP_001179349.1?report=genbank&log$=protalign&blast_rank=3&RID=0) 77 SQDRTRAEPPQYMIDLYNRYTTDKTSTPASNIVRSFSVEDAVSIATTEDFPFQKHILLFNISIPRHEQITRAELRLYLSC 156

[XP_003133152.2](https://www.ncbi.nlm.nih.gov/protein/XP_003133152.2?report=genbank&log$=protalign&blast_rank=4&RID=0) 78 SQDKTRAEPPQYMIDLYNRYTTDKTSTPASNIVRSFSVEDAVSVSATEDFPFQKHILLFNISVPRHEQITRAELRLYLSC 157

[XP_853886.2](https://www.ncbi.nlm.nih.gov/protein/XP_853886.2?report=genbank&log$=protalign&blast_rank=5&RID=0) 68 AQPRARAEPPQYMLDLYHRYASDKAAAPASNVVRSFSVEDAVSIMATEDFPFQKHILLFNVSIPRHEQITRAELRLYVSC 147

[XP_001500704.1](https://www.ncbi.nlm.nih.gov/protein/XP_001500704.1?report=genbank&log$=protalign&blast_rank=6&RID=0) 75 SQDKTRAEPPQYMIDLYNRYTTDKSSTPTSNIVRSFSVEDAVSVMATEDLSFQKHILFFNISIPRHEQITRAELRLHISC 154

[XP_004021599.3](https://www.ncbi.nlm.nih.gov/protein/XP_004021599.3?report=genbank&log$=protalign&blast_rank=7&RID=0) 77 SQDRTRAEPPQYMIDLYNRYTTDKTSTPASNIVRSFSVEDAVSLATTEDFPFQKHFLLFNISIPRHEQITRAELRLYVSC 156

[XP_507775.4](https://www.ncbi.nlm.nih.gov/protein/XP_507775.4?report=genbank&log$=protalign&blast_rank=8&RID=0) 77 SQDKTRVEPPQYMIDLYNRYTSDKSTTPASNIVRSFSMEDAISITATEDFPFQKHILLFNISIPRHEQITRAELRLYVSC 156

[XP_001109523.2](https://www.ncbi.nlm.nih.gov/protein/XP_001109523.2?report=genbank&log$=protalign&blast_rank=9&RID=0) 77 SQDKTRVEPPQYMIDLYNRYTSDKSTTPASNIVRSFSMEDAISMTATEDFPFQKHILLFNISIPRHEQITRAELRLYVSC 156

[XP_003994191.1](https://www.ncbi.nlm.nih.gov/protein/XP_003994191.1?report=genbank&log$=protalign&blast_rank=10&RID=0) 77 SQDKTRAEPPQYMIDLYNRYTTDKSTTPASNIVRSFSVEDAVSLAATEEFPFQKHILLFNISIPRHEQITRAELRLYASC 156

[XP_005699391.1](https://www.ncbi.nlm.nih.gov/protein/XP_005699391.1?report=genbank&log$=protalign&blast_rank=11&RID=0) 77 SQDRTRAEPPQYMIDLYNRYTTDKTSTPASNIVRSFSVEDAVSLATTEDFPFQKHILLFNISIPRHEQITRAELRLYVSC 156

[XP_004049419.1](https://www.ncbi.nlm.nih.gov/protein/XP_004049419.1?report=genbank&log$=protalign&blast_rank=12&RID=0) 77 SQDKTRVEPPQYMIDLYNRYTSDKSTTPASNIVRSFSMEDAISITATEDFPFQKHILLFNISIPRHEQITRAELRLYVSC 156

[XP_002820746.1](https://www.ncbi.nlm.nih.gov/protein/XP_002820746.1?report=genbank&log$=protalign&blast_rank=13&RID=0) 77 SQDKTRVEPPQYMIDLYNRYTSDKSTTPASNIVRSFSMEDAISITATEDFPFQKHILLFNISIPRHEQITRAELRLYVSC 156

[XP_032026853.1](https://www.ncbi.nlm.nih.gov/protein/XP_032026853.1?report=genbank&log$=protalign&blast_rank=14&RID=0) 77 SQDKTRVEPPQYMIDLYNRYTSDKSTTPASNIVRSFSMEDAISITATEDFPFQKHILLFNISIPRHEQITRAELRLYVSC 156

[NP_057288.1](https://www.ncbi.nlm.nih.gov/protein/NP_057288.1?report=genbank&log$=protalign&blast_rank=0&RID=0) 157 QNHVDPSHDLKGSVVIYDVLDGTDAWDSATETKTFLVSQDIQDEGWETLEVSSAVKRWVRSDSTKSKNKLEVTVESHRKG 236

[NP_062379.3](https://www.ncbi.nlm.nih.gov/protein/NP_062379.3?report=genbank&log$=protalign&blast_rank=1&RID=0) 156 QNDVDSTHGLEGSMVVYDVLEDSETWDQATGTKTFLVSQDIRDEGWETLEVSSAVKRWVRADSTTNKNKLEVTVQSHRES 235

[NP_001099566.1](https://www.ncbi.nlm.nih.gov/protein/NP_001099566.1?report=genbank&log$=protalign&blast_rank=2&RID=0) 156 QNHVDSTHGLEGNMVVYDVLDVDETWDSASGTKTFLVSQDIQDEGWETLEVSSAVKRWVRADSTTNKNKLEVTVQRHREG 235

[NP_001179349.1](https://www.ncbi.nlm.nih.gov/protein/NP_001179349.1?report=genbank&log$=protalign&blast_rank=3&RID=0) 157 QSHMDSPHELKGNMVIYDVLDGAEVWDAPAGTKTFLVSQDIRDEGWETFEVSSAVKRWIRADSTKSKNKLEVTVESHRKG 236

[XP_003133152.2](https://www.ncbi.nlm.nih.gov/protein/XP_003133152.2?report=genbank&log$=protalign&blast_rank=4&RID=0) 158 QSHVDASHELKGNMIIYDVLDGA-----SEGTKTFLVSQDIRDEGWETFEVSSAVKRWAQADSTKSKNKLEVTVESHRKG 232

[XP_853886.2](https://www.ncbi.nlm.nih.gov/protein/XP_853886.2?report=genbank&log$=protalign&blast_rank=5&RID=0) 148 QGHGAASRELRGNMAIYDVLDGADAWDASAGTKTFLVSQDISDEGWETFEVSSAVKRWVRADSTKSKNKLEVTVESHRKG 227

[XP_001500704.1](https://www.ncbi.nlm.nih.gov/protein/XP_001500704.1?report=genbank&log$=protalign&blast_rank=6&RID=0) 155 QSHVDSSHELKGNMVIYDVLDGADAWDTSMGTKTFLVSQDIRDEGWETFEVSSAVKRWVRADSTKSKNKLEVTVESHRKG 234

[XP_004021599.3](https://www.ncbi.nlm.nih.gov/protein/XP_004021599.3?report=genbank&log$=protalign&blast_rank=7&RID=0) 157 QSHVDSSHELKGNMVIYDVLDGAEVWDAPGGTKTFLVSQDIRDEGWETFEVSSAVKRWVRADSTKSKNKLEVTVESHRKG 236

[XP_507775.4](https://www.ncbi.nlm.nih.gov/protein/XP_507775.4?report=genbank&log$=protalign&blast_rank=8&RID=0) 157 QNHVDPSHDLKGSVVIYDVLDGTDAWDSATETKTFLVSQDIQDEGWETLEVSSAVKRWVRSDSIKSKNKLEVTVESHRKG 236

[XP_001109523.2](https://www.ncbi.nlm.nih.gov/protein/XP_001109523.2?report=genbank&log$=protalign&blast_rank=9&RID=0) 157 QNHMDHSHDLKGSMVIYDVLDGTDAWDSAAETKTFLVSQDIRDEGWETLEVSSAVKRWVRSDSTKSKNKLEVTVESHRKG 236

[XP_003994191.1](https://www.ncbi.nlm.nih.gov/protein/XP_003994191.1?report=genbank&log$=protalign&blast_rank=10&RID=0) 157 QNHGDSSHELKGNMAIYDVLDGADAWDASTGTKTFLVSQDIRDEGWETFEVSSAVKRWARADSTKSKNKLEVTVESHRKG 236

[XP_005699391.1](https://www.ncbi.nlm.nih.gov/protein/XP_005699391.1?report=genbank&log$=protalign&blast_rank=11&RID=0) 157 QSHVDSSHELKGNMVIYDVLDGAEVWDAPGGTKTFLVSQDIRDEGWETFEVSSAVKRWVRADSTKSKNKLEVTVESRRKG 236

[XP_004049419.1](https://www.ncbi.nlm.nih.gov/protein/XP_004049419.1?report=genbank&log$=protalign&blast_rank=12&RID=0) 157 QNHVDPSHDLKGSVVIYDVLDGTDAWDSATETKTFLVSQDIQDEGWETLEVSSAVKRWVRSDSIKSKNKLEVTVESHRKG 236

[XP_002820746.1](https://www.ncbi.nlm.nih.gov/protein/XP_002820746.1?report=genbank&log$=protalign&blast_rank=13&RID=0) 157 QNHVDPSHDLKGSVVIYDVLDGTDAWDSAAETKTFLVSQDIQDEGWETLEVSSAVKRWVRSDSTKSKNKLEVTVESHRKG 236

[XP_032026853.1](https://www.ncbi.nlm.nih.gov/protein/XP_032026853.1?report=genbank&log$=protalign&blast_rank=14&RID=0) 157 QNHVDPSHDLKGNVVIYDVLDGTDAWDSATETKTFLVSQDIQDEGWETLEVSSAVKRWVRSDSTKSKNKLEVTVENHRKG 236

[NP_057288.1](https://www.ncbi.nlm.nih.gov/protein/NP_057288.1?report=genbank&log$=protalign&blast_rank=0&RID=0) 237 CDTLDISVPPGSRNLPFFVVFSNDHSSGTKETRLELREMISHEQESVLKKLSKDGSTEAGESSH--EEDTDGHVAAGSTL 314

[NP_062379.3](https://www.ncbi.nlm.nih.gov/protein/NP_062379.3?report=genbank&log$=protalign&blast_rank=1&RID=0) 236 CDTLDISVPPGSKNLPFFVVFSNDRSNGTKETRLELKEMIGHEQETMLVKTAKNAYQVAGES-Q-eEEGLDGYTAVGPLL 313

[NP_001099566.1](https://www.ncbi.nlm.nih.gov/protein/NP_001099566.1?report=genbank&log$=protalign&blast_rank=2&RID=0) 236 CSTLDISVPPGSQNLPFFVVFSNDRSNGTKETRLELKEMIGHEQETVLVKTSKNAYQEAGESRE-eEERIDGYTAVGPLL 314

[NP_001179349.1](https://www.ncbi.nlm.nih.gov/protein/NP_001179349.1?report=genbank&log$=protalign&blast_rank=3&RID=0) 237 CDKLDISVPPGSKNLPFFVVFSNDRSNGTKETRLELREMIGHEQESVLRKLSKNTVVEAGENKDeeEEDVRSHTPTESSL 316

[XP_003133152.2](https://www.ncbi.nlm.nih.gov/protein/XP_003133152.2?report=genbank&log$=protalign&blast_rank=4&RID=0) 233 CDKLDISVPPGSKNLPFFVVFSNDRSNGTKETTLELREMISHEQESVLKKLSRNSLLEAGEDKDgeEEGMEGHVAMGSSL 312

[XP_853886.2](https://www.ncbi.nlm.nih.gov/protein/XP_853886.2?report=genbank&log$=protalign&blast_rank=5&RID=0) 228 CDKLDISVPPGSKNLPFFVVFSNDRSNGTKETRLELREMISHEQDSVLTKWSKNSPAGAGDRKA--EGGGEGHMATGSSL 305

[XP_001500704.1](https://www.ncbi.nlm.nih.gov/protein/XP_001500704.1?report=genbank&log$=protalign&blast_rank=6&RID=0) 235 CDRLDISVPPGSKNLPFFVVFSNDRSNGTKETRLELREMIGHEQESVLRKLSKDGLAEADENKD--EEDVEGSMAAGSSL 312

[XP_004021599.3](https://www.ncbi.nlm.nih.gov/protein/XP_004021599.3?report=genbank&log$=protalign&blast_rank=7&RID=0) 237 CDKLDISVPPGSKNLPFFVVFSNDRSNGTKETRLELREMISHEQESVIKKLSRNTVVEAGENKDeeQ-DVQGHVPTAASL 315

[XP_507775.4](https://www.ncbi.nlm.nih.gov/protein/XP_507775.4?report=genbank&log$=protalign&blast_rank=8&RID=0) 237 CDTLDISVPPGSRNLPFFVVFSNDHSSGTKETRLELREMISHEQESVLKKLSKDGSTEAGESSH--EEDTDGHVAAGSTL 314

[XP_001109523.2](https://www.ncbi.nlm.nih.gov/protein/XP_001109523.2?report=genbank&log$=protalign&blast_rank=9&RID=0) 237 CDKLDISVPPGSRNLPFFVVFSNDHSSGTKETRLELREMISHEQESVLKKLSKEGSTEAGESSH--EEDADGHVAVGSTL 314

[XP_003994191.1](https://www.ncbi.nlm.nih.gov/protein/XP_003994191.1?report=genbank&log$=protalign&blast_rank=10&RID=0) 237 CDKLDISVPPGPQNLPFFVVFSNDRSNGTKETRLELREMIGHEQESVLKKLSKNGPAEAGDNKD--E-DGEGRKATGSSL 313

[XP_005699391.1](https://www.ncbi.nlm.nih.gov/protein/XP_005699391.1?report=genbank&log$=protalign&blast_rank=11&RID=0) 237 CDKLDISVPPGSKNLPFFVVFSNDRSNGTKETRLELREMISHEQESVLKKLSRNTVVEAGENKDeeQ-DAQGPVPTAASL 315

[XP_004049419.1](https://www.ncbi.nlm.nih.gov/protein/XP_004049419.1?report=genbank&log$=protalign&blast_rank=12&RID=0) 237 CDTLDISVPPGSRNLPFFVVFSNDHSSGTKETRLELREMISHEQESVLKKLSKDGSTEAGESSH--EEDTDGHVAAGSTL 314

[XP_002820746.1](https://www.ncbi.nlm.nih.gov/protein/XP_002820746.1?report=genbank&log$=protalign&blast_rank=13&RID=0) 237 CDTLDINVPPGSRNLPFFVVFSNDHSSGTKETRLELREMISHEQESVLKKLSKDGSTEAGESSH--EEDTDGHVAAGSTL 314

[XP_032026853.1](https://www.ncbi.nlm.nih.gov/protein/XP_032026853.1?report=genbank&log$=protalign&blast_rank=14&RID=0) 237 CDTLDISVPPGSRNLPFFVVFSNDHSSGTKETRLELREMISHEQESVLKKLSKDGSTEQGESSH--EEDADGHVAAGSTL 314

[NP_057288.1](https://www.ncbi.nlm.nih.gov/protein/NP_057288.1?report=genbank&log$=protalign&blast_rank=0&RID=0) 315 ARRKRSAG-AGSHCQKTSLRVNFEDIGWDSWIIAPKEYEAYECKGGCFFPLADDVTPTKHAIVQTLVHLKFPTKVGKACC 393

[NP_062379.3](https://www.ncbi.nlm.nih.gov/protein/NP_062379.3?report=genbank&log$=protalign&blast_rank=1&RID=0) 314 ARRKRSTG-ASSHCQKTSLRVNFEDIGWDSWIIAPKEYDAYECKGGCFFPLADDVTPTKHAIVQTLVHLKFPTKVGKACC 392

[NP_001099566.1](https://www.ncbi.nlm.nih.gov/protein/NP_001099566.1?report=genbank&log$=protalign&blast_rank=2&RID=0) 315 ARRKRSTGaASSHCQKTSLRVNFEDIGWDSWIIAPKEYDAYECKGGCFFPLADDVTPTKHAIVQTLVHLKFPTKVGKACC 394

[NP_001179349.1](https://www.ncbi.nlm.nih.gov/protein/NP_001179349.1?report=genbank&log$=protalign&blast_rank=3&RID=0) 317 VRRKRSTG-ANNHCQKTSLRVNFEDIGWDSWIIAPKEYDAFECKGGCFFPLADDVTPTKHAIVKTLVHLKFPMKVGKACC 395

[XP_003133152.2](https://www.ncbi.nlm.nih.gov/protein/XP_003133152.2?report=genbank&log$=protalign&blast_rank=4&RID=0) 313 ARRKRSAG-ANNHCQKTSLRVNFEDIGWDSWIIAPKEYDAYECKGGCFFPLADDVTPTKHAIVQTLVHLKFPMKVGKACC 391

[XP_853886.2](https://www.ncbi.nlm.nih.gov/protein/XP_853886.2?report=genbank&log$=protalign&blast_rank=5&RID=0) 306 ARRKRSAG-ANNHCQKTSLRVNFEDIGWDSWIIAPKEYDAYECKGGCFFPLADDMTPTKHAIVQTLVHLKFPMKVGKACC 384

[XP_001500704.1](https://www.ncbi.nlm.nih.gov/protein/XP_001500704.1?report=genbank&log$=protalign&blast_rank=6&RID=0) 313 ARRKRSAG-AGNHCQKTSLRVNFEDIGWDSWIIAPKEYDAYECKGGCFFPLADDVTPTKHAIVQTLVHLKFPMKVGKACC 391

[XP_004021599.3](https://www.ncbi.nlm.nih.gov/protein/XP_004021599.3?report=genbank&log$=protalign&blast_rank=7&RID=0) 316 VRRKRSAG-ANSHCQKTSLRVNFEDIGWDSWIIAPKEYDAFECKGGCFFPLADDVTPTKHAIVKTLVHLKFPMKVGKACC 394

[XP_507775.4](https://www.ncbi.nlm.nih.gov/protein/XP_507775.4?report=genbank&log$=protalign&blast_rank=8&RID=0) 315 ARRKRSAG-AGSHCQKTSLRVNFEDIGWDSWIIAPKEYEAYECKGGCFFPLADDVTPTKHAIVQTLVHLKFPTKVGKACC 393

[XP_001109523.2](https://www.ncbi.nlm.nih.gov/protein/XP_001109523.2?report=genbank&log$=protalign&blast_rank=9&RID=0) 315 SRRKRSTG-AGSHCQKTSLRVNFEDIGWNSWIIAPKEYEAYECKGGCFFPLADDVTPTKHAIVQTLVHLKFPTKVGKACC 393

[XP_003994191.1](https://www.ncbi.nlm.nih.gov/protein/XP_003994191.1?report=genbank&log$=protalign&blast_rank=10&RID=0) 314 ARRKRSAG-ANNHCQKTSLRVNFEDIGWDSWIIAPKEYDAYECKGGCFFPLADDVTPTKHAIVQTLVHLKFPMKVGKACC 392

[XP_005699391.1](https://www.ncbi.nlm.nih.gov/protein/XP_005699391.1?report=genbank&log$=protalign&blast_rank=11&RID=0) 316 VRRKRSAG-ANSHCQKTSLRVNFEDIGWDSWIIAPKEYDAFECKGGCFFPLADDVTPTKHAIVKTLVHLKFPMKVGKACC 394

[XP_004049419.1](https://www.ncbi.nlm.nih.gov/protein/XP_004049419.1?report=genbank&log$=protalign&blast_rank=12&RID=0) 315 ARRKRSAG-AGSHCQKTSLRVNFEDIGWDSWIIAPKEYEAYECKGGCFFPLADDVTPTKHAIVQTLVHLKFPTKVGKACC 393

[XP_002820746.1](https://www.ncbi.nlm.nih.gov/protein/XP_002820746.1?report=genbank&log$=protalign&blast_rank=13&RID=0) 315 ARRKRSAG-AGSHCQKTSLRVNFEDIGWDSWIIAPKEYEAYECKGGCFFPLADDVTPTKHAIVQTLVHLKFPTKVGKACC 393

[XP_032026853.1](https://www.ncbi.nlm.nih.gov/protein/XP_032026853.1?report=genbank&log$=protalign&blast_rank=14&RID=0) 315 ARRKRSTG-AGSHCQKTSLRVNFEDIGWDSWIIAPKEYEAYECKGGCFFPLADDVTPTKHAIVQTLVHLKFPTKVGKACC 393

[NP_057288.1](https://www.ncbi.nlm.nih.gov/protein/NP_057288.1?report=genbank&log$=protalign&blast_rank=0&RID=0) 394 VPTKLSPISVLYKDDMGVPTLKYHYEGMSVAECGCR 429

[NP_062379.3](https://www.ncbi.nlm.nih.gov/protein/NP_062379.3?report=genbank&log$=protalign&blast_rank=1&RID=0) 393 VPTKLSPISILYKDDMGVPTLKYHYEGMSVAECGCR 428

[NP_001099566.1](https://www.ncbi.nlm.nih.gov/protein/NP_001099566.1?report=genbank&log$=protalign&blast_rank=2&RID=0) 395 VPTKLSPISILYKDDMGVPTLKYHYEGMSVAECGCR 430

[NP_001179349.1](https://www.ncbi.nlm.nih.gov/protein/NP_001179349.1?report=genbank&log$=protalign&blast_rank=3&RID=0) 396 VPTKLSPISILYKDDMGVPTLKYHYEGMSVAECGCR 431

[XP_003133152.2](https://www.ncbi.nlm.nih.gov/protein/XP_003133152.2?report=genbank&log$=protalign&blast_rank=4&RID=0) 392 VPTKLSPISILYKDDMGVPTLKYHYEGMSVAECGCR 427

[XP_853886.2](https://www.ncbi.nlm.nih.gov/protein/XP_853886.2?report=genbank&log$=protalign&blast_rank=5&RID=0) 385 VPTKLSPISILYKDDMGVPTLKYHYEGMSVAECGCR 420

[XP_001500704.1](https://www.ncbi.nlm.nih.gov/protein/XP_001500704.1?report=genbank&log$=protalign&blast_rank=6&RID=0) 392 VPTKLSPISILYKDDMGVPTLKYHYEGMSVAECGCR 427

[XP_004021599.3](https://www.ncbi.nlm.nih.gov/protein/XP_004021599.3?report=genbank&log$=protalign&blast_rank=7&RID=0) 395 VPTKLSPISILYKDDMGVPTLKYHYEGMSVAECGCR 430

[XP_507775.4](https://www.ncbi.nlm.nih.gov/protein/XP_507775.4?report=genbank&log$=protalign&blast_rank=8&RID=0) 394 VPTKLSPISVLYKDDMGVPTLKYHYEGMSVAECGCR 429

[XP_001109523.2](https://www.ncbi.nlm.nih.gov/protein/XP_001109523.2?report=genbank&log$=protalign&blast_rank=9&RID=0) 394 VPTKLSPISILYKDDMGVPTLKYHYEGMSVAECGCR 429

[XP_003994191.1](https://www.ncbi.nlm.nih.gov/protein/XP_003994191.1?report=genbank&log$=protalign&blast_rank=10&RID=0) 393 VPTKLSPISILYKDDMGVPTLKYHYEGMSVAECGCR 428

[XP_005699391.1](https://www.ncbi.nlm.nih.gov/protein/XP_005699391.1?report=genbank&log$=protalign&blast_rank=11&RID=0) 395 VPTKLSPISILYKDDMGVPTLKYHYEGMSVAECGCR 430

[XP_004049419.1](https://www.ncbi.nlm.nih.gov/protein/XP_004049419.1?report=genbank&log$=protalign&blast_rank=12&RID=0) 394 VPTKLSPISVLYKDDMGVPTLKYHYEGMSVAECGCR 429

[XP_002820746.1](https://www.ncbi.nlm.nih.gov/protein/XP_002820746.1?report=genbank&log$=protalign&blast_rank=13&RID=0) 394 VPTKLSPISVLYKDDMGVPTLKYHYEGMSVAECGCR 429

[XP_032026853.1](https://www.ncbi.nlm.nih.gov/protein/XP_032026853.1?report=genbank&log$=protalign&blast_rank=14&RID=0) 394 VPTKLSPISVLYKDDMGVPTLKYHYEGMSVAECGCR 429

GDF9

NP_005251.1 growth/differentiation factor 9 isoform 1 preproprotein [Homo sapiens]

NP_032136.2 growth/differentiation factor 9 precursor [Mus musculus]

NP_067704.1 growth/differentiation factor 9 precursor [Rattus norvegicus]

NP_777106.1 growth/differentiation factor 9 precursor [Bos taurus]

NP_001001909.1 growth/differentiation factor 9 precursor [Sus scrofa]

XP_038536812.1 growth/differentiation factor 9 isoform X1 [Canis lupus familiaris]

XP_001504477.1 growth/differentiation factor 9 [Equus caballus]

NP_001136360.2 growth/differentiation factor 9 precursor [Ovis aries]

XP_527008.1 growth/differentiation factor 9 isoform X1 [Pan troglodytes]

XP_014996256.1 growth/differentiation factor 9 [Macaca mulatta]

NP_001159372.1 growth/differentiation factor 9 precursor [Felis catus]

NP_001272637.1 growth/differentiation factor 9 precursor [Capra hircus]

XP_018883145.1 growth/differentiation factor 9 [Gorilla gorilla gorilla]

XP_002815918.1 growth/differentiation factor 9 isoform X1 [Pongo abelii]

XP_032012334.1 growth/differentiation factor 9 isoform X1 [Hylobates moloch]

[NP_005251.1](https://www.ncbi.nlm.nih.gov/protein/NP_005251.1?report=genbank&log$=protalign&blast_rank=0&RID=0) 1 MARPNKFLLWFCCFAW LCFPISLGSQASGGEAQIAASAELESGAMPWS LLQHIDERDRAGLLPALFKVL 69

[NP_032136.2](https://www.ncbi.nlm.nih.gov/protein/NP_032136.2?report=genbank&log$=protalign&blast_rank=1&RID=0) 1 MALPSNFLLGVCCFAW LCFLSSLSSQASTEESQSGASENVESEADPWS LLLPVDGTDRSGLLPPLFKVL 69

[NP_067704.1](https://www.ncbi.nlm.nih.gov/protein/NP_067704.1?report=genbank&log$=protalign&blast_rank=2&RID=0) 1 MAFPSRFLLGVCCFAW LCLLISLSSQASTGESQAGASENLESEADPWS LLLPVDGTDRSGLLPPLFKVL 69

[NP_777106.1](https://www.ncbi.nlm.nih.gov/protein/NP_777106.1?report=genbank&log$=protalign&blast_rank=3&RID=0) 1 MALPNKFFLWFCCFAW LCFPISLDSQPSRGEAQIVARTALESEAETWS LLKHLDGRHRPGLLSPLLNVL 69

[NP_001001909.1](https://www.ncbi.nlm.nih.gov/protein/NP_001001909.1?report=genbank&log$=protalign&blast_rank=4&RID=0) 1 MALPRKFFLCFCCFAL FCFPVSCGSQASRREAHFATSAVLESEAEPWS LLRPPDERHRSGLPSPLFNVL 69

[XP_038536812.1](https://www.ncbi.nlm.nih.gov/protein/XP_038536812.1?report=genbank&log$=protalign&blast_rank=5&RID=0) 1 [4]LGGLGPTGLWRACACL[25]AAQPVRPRRAAVRPRFSSAPAAASGFREAPWA[48]ALARNRTENSQTCLSFMTLVL 146

[XP_001504477.1](https://www.ncbi.nlm.nih.gov/protein/XP_001504477.1?report=genbank&log$=protalign&blast_rank=6&RID=0) 1 MALPSKFFLWFCCSAW LCFPISLGSQASREAAQIAASAELESEAEPWS LLQPLNGGNRSGLLPALFKVL 69

[NP_001136360.2](https://www.ncbi.nlm.nih.gov/protein/NP_001136360.2?report=genbank&log$=protalign&blast_rank=7&RID=0) 1 MALPNKFFLWFCCFAW LCFPISLDSLPSRGEAQIVARTALESEAETWS LLNHLGGRHRPGLLSPLLEVL 69

[XP_527008.1](https://www.ncbi.nlm.nih.gov/protein/XP_527008.1?report=genbank&log$=protalign&blast_rank=8&RID=0) 1 MALPNKFLLWFCCFAW LCFPISLGSQASGGEAQIAASAELESGAMPWS LLQHIDERDRAGLLPALFKVL 69

[XP_014996256.1](https://www.ncbi.nlm.nih.gov/protein/XP_014996256.1?report=genbank&log$=protalign&blast_rank=9&RID=0) 1 MALPNKFLLWFYCFAW LCFPVSLGSQASGGDAQIAASAELESGATPWS LLQPIDERDRAGLLPPLFKVL 69

[NP_001159372.1](https://www.ncbi.nlm.nih.gov/protein/NP_001159372.1?report=genbank&log$=protalign&blast_rank=10&RID=0) 1 MALLSNFFLWFLCFSW LCFPMSLCSRAST-EVQIAAGAEWEAKAEPWS LVQPLDEKDRLGFLPPLFKVL 68

[NP_001272637.1](https://www.ncbi.nlm.nih.gov/protein/NP_001272637.1?report=genbank&log$=protalign&blast_rank=11&RID=0) 1 MALPNKFFLWFCCFAW LCFPISLDSLPSRGEAQIVARTALESEAETWS LLNHLGGRHRPGLLSPLLKVL 69

[XP_018883145.1](https://www.ncbi.nlm.nih.gov/protein/XP_018883145.1?report=genbank&log$=protalign&blast_rank=12&RID=0) 1 MALPNKFLLWFCCFAW LCFPISLGSQASGGEAQIAASAELESGAMPWS LLQHIDERDRAGLLPPLFKVL 69

[XP_002815918.1](https://www.ncbi.nlm.nih.gov/protein/XP_002815918.1?report=genbank&log$=protalign&blast_rank=13&RID=0) 1 MALPNKFLLWFCCFAW LCFPITLGSQASGGDAQIAASAELESGATPWS LLQPIDERDRAGLLPPLFKVL 69

[XP_032012334.1](https://www.ncbi.nlm.nih.gov/protein/XP_032012334.1?report=genbank&log$=protalign&blast_rank=14&RID=0) 1 MALPNKFLLWFCCIAW LCFPISLGSQASGGDAQIAASGELESGATPRS LLQPIDERDRAGLLPPLFKVL 69

[NP_005251.1](https://www.ncbi.nlm.nih.gov/protein/NP_005251.1?report=genbank&log$=protalign&blast_rank=0&RID=0) 70 SVGRGGSPRLQPDSRALHYMKKLYKTYATKEGIPKSNRSHLYNTVRLFTPCTRHKQAPGDQVTGILPSVELLFNLDRITT 149

[NP_032136.2](https://www.ncbi.nlm.nih.gov/protein/NP_032136.2?report=genbank&log$=protalign&blast_rank=1&RID=0) 70 SDRRGETPKLQPDSRALYYMKKLYKTYATKEGVPKPSRSHLYNTVRLFSPCAQQEQAPSNQVTGPLPMVDLLFNLDRVTA 149

[NP_067704.1](https://www.ncbi.nlm.nih.gov/protein/NP_067704.1?report=genbank&log$=protalign&blast_rank=2&RID=0) 70 SDRRSETPKLQPDSRALYYMKKLYKTYATKEGVPKPSRSHLYNTVRLFSPCAQQEQAPSNQMTGPLPMVDLLFNLDRVTA 149

[NP_777106.1](https://www.ncbi.nlm.nih.gov/protein/NP_777106.1?report=genbank&log$=protalign&blast_rank=3&RID=0) 70 YDGHREPPRLQPDDRALSYMKRLYKAYATKEGTPKSNRSHLYNTVRLFTPCAQHKQAPGDQAAGTLPSVDLLFNLDRVTV 149

[NP_001001909.1](https://www.ncbi.nlm.nih.gov/protein/NP_001001909.1?report=genbank&log$=protalign&blast_rank=4&RID=0) 70 YDGHGGLPRLQPDSRALRYMKRLYKTYATKEGIPKANRSHLYNTVRLFTPCAQHKQAPGDQVTGTLPSGDLRFNLDRVTA 149

[XP_038536812.1](https://www.ncbi.nlm.nih.gov/protein/XP_038536812.1?report=genbank&log$=protalign&blast_rank=5&RID=0) 147 YDGQGGTP------RALRYMKRLYKACATKEGIPKSNRSPLYNTVRLFTSCAQHTQAPGDQATGSVPPVDLLFNLDRVPP 220

[XP_001504477.1](https://www.ncbi.nlm.nih.gov/protein/XP_001504477.1?report=genbank&log$=protalign&blast_rank=6&RID=0) 70 YDGQGGAPRLQPDSRALRYMKRLYKAYATKEGIPKSNRGHLYNTVRLFTPCAQHKQAPGDQVAGTLPSVDLLFNLDCVTA 149

[NP_001136360.2](https://www.ncbi.nlm.nih.gov/protein/NP_001136360.2?report=genbank&log$=protalign&blast_rank=7&RID=0) 70 YDGHGEPPRLQPDDRALRYMKRLYKAYATKEGTPKSNRRHLYNTVRLFTPCAQHKQAPGDLAAGTFPSVDLLFNLDRVTV 149

[XP_527008.1](https://www.ncbi.nlm.nih.gov/protein/XP_527008.1?report=genbank&log$=protalign&blast_rank=8&RID=0) 70 SVGRGGSPRLQPDSRALHYMKKLYKTYATKEGIPKSNRSHLYNTVRLFTPCTRHKQAPGDQVTGILPSVELLFNLDRITT 149

[XP_014996256.1](https://www.ncbi.nlm.nih.gov/protein/XP_014996256.1?report=genbank&log$=protalign&blast_rank=9&RID=0) 70 SVGRGGAPRLQPDSRALHYMKNLYKTYATKEGIPKSNRSHLYNTVRLFTPCTQHKQVPGDQVTGILPSVDLLFNLDRITT 149

[NP_001159372.1](https://www.ncbi.nlm.nih.gov/protein/NP_001159372.1?report=genbank&log$=protalign&blast_rank=10&RID=0) 69 YNGQGDAPRLQPDSRALRYMKRLYKSFATKEGIPKSNRSPLYNTARLFTSYAQHKQAPGDQVTGTVPSVDLLFHLDRVTA 148

[NP_001272637.1](https://www.ncbi.nlm.nih.gov/protein/NP_001272637.1?report=genbank&log$=protalign&blast_rank=11&RID=0) 70 YDGHGEPPRLQPDDRALRYMKRLYKAYATKEGTPKSNRRHLYNTVRLFTPCAQHKQAPGDLAAGTFPSVDLLFNLDRVTV 149

[XP_018883145.1](https://www.ncbi.nlm.nih.gov/protein/XP_018883145.1?report=genbank&log$=protalign&blast_rank=12&RID=0) 70 SVGRGGSPRLQPDSRALHYMKKLYKTYATKEGIPKSNRSHLYNTVRLFTPCTRHKQAPGDQVTGILPSVELLFNLDRITT 149

[XP_002815918.1](https://www.ncbi.nlm.nih.gov/protein/XP_002815918.1?report=genbank&log$=protalign&blast_rank=13&RID=0) 70 SVGRGRAPRLQPDSRALHYMKKLYKTYATKEGIPKSNRSHLYNTVRLFTPCTQHKQAPGDQVTGILPSVELLFNLDRITT 149

[XP_032012334.1](https://www.ncbi.nlm.nih.gov/protein/XP_032012334.1?report=genbank&log$=protalign&blast_rank=14&RID=0) 70 SVGQGGAPRLQPDSRALHYMKKLYKTYATKEGIPKSNRSHLYNTVRLFTPCTQHKQAPGDQVTGILPSVELLFNLDRITT 149

[NP_005251.1](https://www.ncbi.nlm.nih.gov/protein/NP_005251.1?report=genbank&log$=protalign&blast_rank=0&RID=0) 150 VEHLLKSVLLYNINNSVSFSSAVKCVCNLMIKEPKSSSRTLGRAPYSFTFNSQFEFGKKHKWIQIDVTSLLQPLVASNKR 229

[NP_032136.2](https://www.ncbi.nlm.nih.gov/protein/NP_032136.2?report=genbank&log$=protalign&blast_rank=1&RID=0) 150 MEHLLKSVLLYTLNNSASSSSTVTCMCDLVVKEAMSSGRAPPRAPYSFTL-------KKHRWIEIDVTSLLQPLVTSSER 222

[NP_067704.1](https://www.ncbi.nlm.nih.gov/protein/NP_067704.1?report=genbank&log$=protalign&blast_rank=2&RID=0) 150 MEHLLKSVLLYTLNNSAASSSTVTCVCDLVVKEPMSSSKATPRAPYSFTL-------RKHRWIEMDVTSLLQPLVASSER 222

[NP_777106.1](https://www.ncbi.nlm.nih.gov/protein/NP_777106.1?report=genbank&log$=protalign&blast_rank=3&RID=0) 150 VEHLFKSVLLYTFNNSISFPFPVKCICNLVIKEPEFSSKTLPRAPYSFTFNSQFEFRKKYKWIEIDVTAPLEPLVASHKR 229

[NP_001001909.1](https://www.ncbi.nlm.nih.gov/protein/NP_001001909.1?report=genbank&log$=protalign&blast_rank=4&RID=0) 150 VEHLLKSVLLYTWNNSISFPSPVKCECSLVVKEPELSNKTLPKAPYSFTLNSPFTFQKKHKWIEIDVTAILQPLVVSNKR 229

[XP_038536812.1](https://www.ncbi.nlm.nih.gov/protein/XP_038536812.1?report=genbank&log$=protalign&blast_rank=5&RID=0) 221 VEHLLKSGLLYSFSNSISFPSAVKCLCHLVIKEPEFSSWTPQRAPSLLTFNSQFELKKKYKWFEVDVTTALRPLVALHKR 300

[XP_001504477.1](https://www.ncbi.nlm.nih.gov/protein/XP_001504477.1?report=genbank&log$=protalign&blast_rank=6&RID=0) 150 VEHLLKSVLLYTFNNSVSFPSAVKCVCNLVIKEPE--SKTLPGTPYSFTFNSQFEFRKKYKWIEMDVTPLLQPLVASNKK 227

[NP_001136360.2](https://www.ncbi.nlm.nih.gov/protein/NP_001136360.2?report=genbank&log$=protalign&blast_rank=7&RID=0) 150 VEHLFKSVLLYTFNNSISFPFPVKCICNLVIKEPEFSSKTLPRAPYSFTYNSQFEFRKKYKWMEIDVTAPLEPLVASHKR 229

[XP_527008.1](https://www.ncbi.nlm.nih.gov/protein/XP_527008.1?report=genbank&log$=protalign&blast_rank=8&RID=0) 150 VERLLKSVLLYNINNSVSFSSAVKCVCNLMIKESKSSSRTLGRAPYSFTFNSQFEFGKKHKWIQIDVTSLLQPLVASNKR 229

[XP_014996256.1](https://www.ncbi.nlm.nih.gov/protein/XP_014996256.1?report=genbank&log$=protalign&blast_rank=9&RID=0) 150 VEHLLKSVLLYTINNSVSFSSAVKCVCNLMIKEPKFSSKTLHRAPYSFTFNSQFEFGKKHKWIEIDVTSLLQPLVASNKR 229

[NP_001159372.1](https://www.ncbi.nlm.nih.gov/protein/NP_001159372.1?report=genbank&log$=protalign&blast_rank=10&RID=0) 149 VEHFIKSVLLFTFSNPISFSSAVKCVCNLVMKEPESSSGNPHRTLSSLTFDSQFEFRKKYRWIEVDVTAPLQPLVASNQR 228

[NP_001272637.1](https://www.ncbi.nlm.nih.gov/protein/NP_001272637.1?report=genbank&log$=protalign&blast_rank=11&RID=0) 150 VEHLFKSVLLYTFNNSISFPFPVKCICNLVIKEPEFSSKTLPRAPYSFTYNSQFEFRKKYKWMEIDVTAPLEPLVASHKR 229

[XP_018883145.1](https://www.ncbi.nlm.nih.gov/protein/XP_018883145.1?report=genbank&log$=protalign&blast_rank=12&RID=0) 150 VEHLLKSVLLYNINNSVSFSSAVRCVCNLMIKEPKSSSRTLCRAPYSFTFNSQFEFGKKHKWIQIDVTSLLQPLVASNKR 229

[XP_002815918.1](https://www.ncbi.nlm.nih.gov/protein/XP_002815918.1?report=genbank&log$=protalign&blast_rank=13&RID=0) 150 VEHLLKSVLLYTINNSVSFSSAVKCVCNLMIKEPESSSRTLCRAPYSFTFNSQFEFGKKHKWIQIDVTSLLQPLVASNKR 229

[XP_032012334.1](https://www.ncbi.nlm.nih.gov/protein/XP_032012334.1?report=genbank&log$=protalign&blast_rank=14&RID=0) 150 VEHLLKSVLLYTINNSVSFSSAVKCVCNLMIKEPKSSSRTLCRAPYSFTFNSQFEFGKKHKWIQIDVTSLLQPLVASNKR 229

[NP_005251.1](https://www.ncbi.nlm.nih.gov/protein/NP_005251.1?report=genbank&log$=protalign&blast_rank=0&RID=0) 230 SIHMSINFTCMKDQLEHPSAQNGLFNMTL-VSPSLILYLNDTSAQAYHSWYSLHYKRRPSQGPDQERSLSAYPVGEEAAE 308

[NP_032136.2](https://www.ncbi.nlm.nih.gov/protein/NP_032136.2?report=genbank&log$=protalign&blast_rank=1&RID=0) 223 SIHLSVNFTCTKDQVP----EDGVFSMPLSVPPSLILYLNDTSTQAYHSWQSLQSTWRPLQHPGQA-GVAARPVKEEAIE 297

[NP_067704.1](https://www.ncbi.nlm.nih.gov/protein/NP_067704.1?report=genbank&log$=protalign&blast_rank=2&RID=0) 223 SIHLSVNFTCTRDQAP----ENGTFNMPLSVPPSLILYLNDTSTQAYHSWQSLQSTQRHSQHPGQD-SVTTRPVEEEATE 297

[NP_777106.1](https://www.ncbi.nlm.nih.gov/protein/NP_777106.1?report=genbank&log$=protalign&blast_rank=3&RID=0) 230 NIHMSVNFTCVKDQLQHPSARDSLFNMTLLLAPSLLLYLNDTSAQAFHRWHSLHPKRKPSQDPDQKRGLSACPMGEEAAE 309

[NP_001001909.1](https://www.ncbi.nlm.nih.gov/protein/NP_001001909.1?report=genbank&log$=protalign&blast_rank=4&RID=0) 230 EIHMSIDFTCVKDQLQHLSAQESPCNMTLLAAPSLLLYLNDTSTQAYHRWYSLPWKRRPSQGPDQKRGLS---------E 300

[XP_038536812.1](https://www.ncbi.nlm.nih.gov/protein/XP_038536812.1?report=genbank&log$=protalign&blast_rank=5&RID=0) 301 SLHMSVNFTCVRNQQQDPVAQDGPWNRTLLVPPSLLLYLNDTSAQAHHRWDSLHYKRRSSQRADQKGGLSACPIGEESTE 380

[XP_001504477.1](https://www.ncbi.nlm.nih.gov/protein/XP_001504477.1?report=genbank&log$=protalign&blast_rank=6&RID=0) 228 SIHMSVNLTCGKDQLQHPSAQDSPLNTTLLLFPSLLLYLNDTSAQAYHRWHSLHYKRRPSQGPDQKRDLSACPEGEGAAE 307

[NP_001136360.2](https://www.ncbi.nlm.nih.gov/protein/NP_001136360.2?report=genbank&log$=protalign&blast_rank=7&RID=0) 230 NIHMSVNFTCAEDQLQHPSARDSLFNMTLLVAPSLLLYLNDTSAQAFHRWHSLHPKRKPSQGPDQKRGLSAYPVGEEAAE 309

[XP_527008.1](https://www.ncbi.nlm.nih.gov/protein/XP_527008.1?report=genbank&log$=protalign&blast_rank=8&RID=0) 230 SIHMSINFTCMKDQLEHPSAQNGLFNMTLLVSPSLILYLNDTSAQAYHSWYSLHYKRRPSQGPDQERSLSAYPVGEEAAE 309

[XP_014996256.1](https://www.ncbi.nlm.nih.gov/protein/XP_014996256.1?report=genbank&log$=protalign&blast_rank=9&RID=0) 230 SIHMSINFTCMKDQLEHPSAQNGLFNMTLLVPPSLILYLNDTSAQAYHRWYSLYYKRRPSQGPDQERSLSAYPVGEDAAE 309

[NP_001159372.1](https://www.ncbi.nlm.nih.gov/protein/NP_001159372.1?report=genbank&log$=protalign&blast_rank=10&RID=0) 229 NIHMSVNLTCVKAQLQPPSARDGPFNVTLLVPPSLLLYLNDTSAQAHHRWYSLHYKRRPSQGAGQKRGLSACPQGEESAE 308

[NP_001272637.1](https://www.ncbi.nlm.nih.gov/protein/NP_001272637.1?report=genbank&log$=protalign&blast_rank=11&RID=0) 230 NIHMSVNFTCAKDQLQHPSARDSLFNMTLLVAPSLLLYLNDTSAQAFHRWHSLHPKRKPSQGPDQRRELSAYPVGEEAAE 309

[XP_018883145.1](https://www.ncbi.nlm.nih.gov/protein/XP_018883145.1?report=genbank&log$=protalign&blast_rank=12&RID=0) 230 SIHMSINFTCMKDQLEHPSAQNGLFNMTLLVSPSLILYLNDTSAQAYHSWYSLHYKRRPSQGPDQERSLSAYPVGEEAAE 309

[XP_002815918.1](https://www.ncbi.nlm.nih.gov/protein/XP_002815918.1?report=genbank&log$=protalign&blast_rank=13&RID=0) 230 SIHMSINFTCMKDQLEHPSAQNGLFNMTLLVSPSLILYLNDTSAQAYHSWYSLNYKRRPSQGPDQERSLSAYPVGEEAAE 309

[XP_032012334.1](https://www.ncbi.nlm.nih.gov/protein/XP_032012334.1?report=genbank&log$=protalign&blast_rank=14&RID=0) 230 SIHMSINFTCMKDQLEHLSVQNGLFNMTLLVSPSLILYLNDTSAQAYHSWYSLHYKRRPSQGPDQERSLSAYPVGKEAAE 309

[NP_005251.1](https://www.ncbi.nlm.nih.gov/protein/NP_005251.1?report=genbank&log$=protalign&blast_rank=0&RID=0) 309 DGRSShhRHRRGQETVSSELKKPLGPASFNLSEYFRQFLLPQNECELHDFRLSFSQLKWDNWIVAPHRYNPRYCKGDCPR 388

[NP_032136.2](https://www.ncbi.nlm.nih.gov/protein/NP_032136.2?report=genbank&log$=protalign&blast_rank=1&RID=0) 298 VERSP--RRRRGQKAIRSEAKGPLLTASFNLSEYFKQFLFPQNECELHDFRLSFSQLKWDNWIVAPHRYNPRYCKGDCPR 375

[NP_067704.1](https://www.ncbi.nlm.nih.gov/protein/NP_067704.1?report=genbank&log$=protalign&blast_rank=2&RID=0) 298 VERSP--RHRRGQKTLSSETKKPL-TASFNLSEYFRQFLFPQNECELHDFRLSFSQLKWDNWIVAPHRYNPRYCKGDCPR 374

[NP_777106.1](https://www.ncbi.nlm.nih.gov/protein/NP_777106.1?report=genbank&log$=protalign&blast_rank=3&RID=0) 310 GVRLS--RHRRDQESVSSELKKPLVPASFNLSEYFKQFLFPQNECELHDFRLSFSQLKWDNWIVAPHKYNPRYCKGDCPR 387

[NP_001001909.1](https://www.ncbi.nlm.nih.gov/protein/NP_001001909.1?report=genbank&log$=protalign&blast_rank=4&RID=0) 301 GVPSS--RHRRAQDTVSSELKKPLVPASFNLSEYFKQFLFPQNECELHDFRLSFSQLKWDNWIVAPHKYNPRYCKGDCPR 378

[XP_038536812.1](https://www.ncbi.nlm.nih.gov/protein/XP_038536812.1?report=genbank&log$=protalign&blast_rank=5&RID=0) 381 GGRSS--RHRRGQDTVSLELHKPLAPASFNLSEYLKHFLFPQHECELHDFRLSFSQLKWDNWIVAPHRYNPRYCKGDCPR 458

[XP_001504477.1](https://www.ncbi.nlm.nih.gov/protein/XP_001504477.1?report=genbank&log$=protalign&blast_rank=6&RID=0) 308 GITSS--RHRRSQEAVSSELKKPLVPASLNLSEYFKQFLFPQNECELHDFRLSFSQLKWDNWIVAPQRYNPRYCKGDCPR 385

[NP_001136360.2](https://www.ncbi.nlm.nih.gov/protein/NP_001136360.2?report=genbank&log$=protalign&blast_rank=7&RID=0) 310 GVRSS--RHRRDQESASSELKKPLVPASVNLSEYFKQFLFPQNECELHDFRLSFSQLKWDNWIVAPHKYNPRYCKGDCPR 387

[XP_527008.1](https://www.ncbi.nlm.nih.gov/protein/XP_527008.1?report=genbank&log$=protalign&blast_rank=8&RID=0) 310 DGRSShhRHRRGQETVSSELKKPLGPASFNLSEYFKQFLLPQNECELHDFRLSFSQLKWDNWIVAPHRYNPRYCKGDCPR 389

[XP_014996256.1](https://www.ncbi.nlm.nih.gov/protein/XP_014996256.1?report=genbank&log$=protalign&blast_rank=9&RID=0) 310 DGRSShhRHRRGQETVSSELKKPLVPASFNLSEYFKQFLFPQNECELHDFRLSFSQLKWDNWIVAPHRYNPRYCKGDCPR 389

[NP_001159372.1](https://www.ncbi.nlm.nih.gov/protein/NP_001159372.1?report=genbank&log$=protalign&blast_rank=10&RID=0) 309 AVRPS--RHRRGQETIGLEPQKPLVPASFNLSEYFKQFLFPQNECELHDFRLSFSQLKWDSWIVAPHRYNPRYCKGDCPR 386

[NP_001272637.1](https://www.ncbi.nlm.nih.gov/protein/NP_001272637.1?report=genbank&log$=protalign&blast_rank=11&RID=0) 310 GVRSS--RHRRDQESVSSELKKPLVPASVNLSEYFKQFLFPQNECELHDFRLSFSQLKWDNWIVAPHKYNPRYCKGDCPR 387

[XP_018883145.1](https://www.ncbi.nlm.nih.gov/protein/XP_018883145.1?report=genbank&log$=protalign&blast_rank=12&RID=0) 310 DGRSShhRHRRGQETVSSELKKPLGPASFNLSEYFKQFLLPQNECELHDFRLSFSQLKWDNWIVAPHRYNPRYCKGDCPR 389

[XP_002815918.1](https://www.ncbi.nlm.nih.gov/protein/XP_002815918.1?report=genbank&log$=protalign&blast_rank=13&RID=0) 310 DGRSShrRHRRGQETVSSELKKPLVPASFNLSEYFKQFIFPQNECELHDFRLSFSQLKWDNWIVAPHRYNPRYCKGDCPR 389

[XP_032012334.1](https://www.ncbi.nlm.nih.gov/protein/XP_032012334.1?report=genbank&log$=protalign&blast_rank=14&RID=0) 310 DGRSShhRHRRGQETVSSELKKPLVPASFNLSEYFKQFLFPQNECELHDFRLSFSQLKWDNWIVAPHRYNPRYCKGDCPR 389

[NP_005251.1](https://www.ncbi.nlm.nih.gov/protein/NP_005251.1?report=genbank&log$=protalign&blast_rank=0&RID=0) 389 AVGHRYGSPVHTMVQNIIYEKLDSSVPRPSCVPAKYSPLSVLTIEPDGSIAYKEYEDMIATKCTCR 454

[NP_032136.2](https://www.ncbi.nlm.nih.gov/protein/NP_032136.2?report=genbank&log$=protalign&blast_rank=1&RID=0) 376 AVRHRYGSPVHTMVQNIIYEKLDPSVPRPSCVPGKYSPLSVLTIEPDGSIAYKEYEDMIATRCTCR 441

[NP_067704.1](https://www.ncbi.nlm.nih.gov/protein/NP_067704.1?report=genbank&log$=protalign&blast_rank=2&RID=0) 375 AVRHRYGSPVHTMVQNIIYEKLDPSVPRPSCVPGKYSPLSVLTIEPDGSIAYKEYEDMIATRCTCR 440

[NP_777106.1](https://www.ncbi.nlm.nih.gov/protein/NP_777106.1?report=genbank&log$=protalign&blast_rank=3&RID=0) 388 AVGHRYGSPVHTMVMNIIHEKLDSSVPRPSCVPAKYSPLSVLAIEPDGSIAYKEYEDMIATKCTCR 453

[NP_001001909.1](https://www.ncbi.nlm.nih.gov/protein/NP_001001909.1?report=genbank&log$=protalign&blast_rank=4&RID=0) 379 AVGHRYGSPVHTMVQNIIHEKLDSSVPRPSCVPAKYSPLSVLAIEPDGSIAYKEYEDMIATKCTCR 444

[XP_038536812.1](https://www.ncbi.nlm.nih.gov/protein/XP_038536812.1?report=genbank&log$=protalign&blast_rank=5&RID=0) 459 AVGHRYGSPVHTMVQNIIHEKLNSSVPRPSCVPAKYSPLSVLTIEPDGSIAYKEYEDMIATKCTCR 524

[XP_001504477.1](https://www.ncbi.nlm.nih.gov/protein/XP_001504477.1?report=genbank&log$=protalign&blast_rank=6&RID=0) 386 AVGHRYGSPVHTMVQNIIHEKLDSSVPRPSCVPAKYSPLSVLTIESDGSITYKEYEDMIATKCTCR 451

[NP_001136360.2](https://www.ncbi.nlm.nih.gov/protein/NP_001136360.2?report=genbank&log$=protalign&blast_rank=7&RID=0) 388 AVGHRYGSPVHTMVQNIIHEKLDSSVPRPSCVPAKYSPLSVLAIEPDGSIAYKEYEDMIATKCTCR 453

[XP_527008.1](https://www.ncbi.nlm.nih.gov/protein/XP_527008.1?report=genbank&log$=protalign&blast_rank=8&RID=0) 390 AVGHRYGSPVHTMVQNIIYEKLDSSVPRPSCVPAKYSPLSVLTIEPDGSIAYKEYEDMIATKCTCR 455

[XP_014996256.1](https://www.ncbi.nlm.nih.gov/protein/XP_014996256.1?report=genbank&log$=protalign&blast_rank=9&RID=0) 390 AVGHRYGSPVHTMVQNIIYEKLDSSVPRPSCVPAKYSPLSVLTIEPDGSIAYKEYEDMIATKCTCR 455

[NP_001159372.1](https://www.ncbi.nlm.nih.gov/protein/NP_001159372.1?report=genbank&log$=protalign&blast_rank=10&RID=0) 387 ALGHRYGSPVHTMVQNIIHEKLDSSVPRPSCVPAKYSPLSVLTIESDGSIAYKEYEDMIATKCTCR 452

[NP_001272637.1](https://www.ncbi.nlm.nih.gov/protein/NP_001272637.1?report=genbank&log$=protalign&blast_rank=11&RID=0) 388 AVGHRYGSPVHTMVQNIIHEKLDSSVPRPSCVPAKYSPLSVLAIEPDGSIAYKEYEDMIATKCTCR 453

[XP_018883145.1](https://www.ncbi.nlm.nih.gov/protein/XP_018883145.1?report=genbank&log$=protalign&blast_rank=12&RID=0) 390 AVGHRYGSPVHTMVQNIIYEKLDSSVPRPSCVPAKYSPLSVLTIEPDGSIAYKEYEDMIATKCTCR 455

[XP_002815918.1](https://www.ncbi.nlm.nih.gov/protein/XP_002815918.1?report=genbank&log$=protalign&blast_rank=13&RID=0) 390 AVGHRYGSPVHTMVQNIIYEKLDSSVPRPSCVPAKYSPLSVLTIEPDGSIAYKEYEDMIATKCTCR 455

[XP_032012334.1](https://www.ncbi.nlm.nih.gov/protein/XP_032012334.1?report=genbank&log$=protalign&blast_rank=14&RID=0) 390 AVGHRYGSPVHTMVQNIIYEKLDSSVPRPSCVPAKYSPLSVLTIEPDGSIAYKEYEDMIATKCTCR 455

GDF5

NP_000548.2 growth/differentiation factor 5 preproprotein [Homo sapiens]

NP_032135.2 growth/differentiation factor 5 preproprotein [Mus musculus]

XP_003749648.1 growth/differentiation factor 5 [Rattus norvegicus]

NP_001179202.1 growth/differentiation factor 5 precursor [Bos taurus]

NP_001231226.1 growth/differentiation factor 5 precursor [Sus scrofa]

XP_542974.1 growth/differentiation factor 5 [Canis lupus familiaris]

NP_001296325.1 growth/differentiation factor 5 precursor [Equus caballus]

XP_004014573.2 growth/differentiation factor 5 [Ovis aries]

XP_530287.4 growth/differentiation factor 5 [Pan troglodytes]

XP_001099806.2 growth/differentiation factor 5 [Macaca mulatta]

XP_003983628.1 growth/differentiation factor 5 [Felis catus]

XP_017913249.1 PREDICTED: growth/differentiation factor 5 [Capra hircus]

XP_018872428.1 PREDICTED: growth/differentiation factor 5 [Gorilla gorilla gorilla]

XP_003779391.1 growth/differentiation factor 5 [Pongo abelii]

XP_031998077.1 growth/differentiation factor 5 [Hylobates moloch]

[NP_000548.2](https://www.ncbi.nlm.nih.gov/protein/NP_000548.2?report=genbank&log$=protalign&blast_rank=0&RID=0) 1 MRLPKLLTFLLWYLAWLDLEFICTVLGAPDLGQRPQGTRPGLAKAEAKERPPLARNVFRPGGHSYGGGAtnANARAKGGT 80

[NP_032135.2](https://www.ncbi.nlm.nih.gov/protein/NP_032135.2?report=genbank&log$=protalign&blast_rank=1&RID=0) 1 MRLPKLLTLLLWHLAWLDLELICTVLGAPDLGQRTPGAKPGLTKAEAKERPPLARNVFRPGGHIYGVGA--TNARAKGSS 78

[XP_003749648.1](https://www.ncbi.nlm.nih.gov/protein/XP_003749648.1?report=genbank&log$=protalign&blast_rank=2&RID=0) 1 MRLPKLLTLLLWHLAWLDLGLVCTVLGAPDLGQRPPGARPGLAKAEAKERPPLTRNIFRPGGHSYGVGA--TSARAKGSS 78

[NP_001179202.1](https://www.ncbi.nlm.nih.gov/protein/NP_001179202.1?report=genbank&log$=protalign&blast_rank=3&RID=0) 1 MRLPKLLTLLLWHLAWLDLEFICTVLGAPDLGQRPQGARPGLAKAEAKERPPLAQNIFRPGGHSYGGGA--TNARAKGGT 78

[NP_001231226.1](https://www.ncbi.nlm.nih.gov/protein/NP_001231226.1?report=genbank&log$=protalign&blast_rank=4&RID=0) 1 MRLPKLLTFLLWHLAWLDLEFICTVLGAPDLGQRPQGARPGLAKAEAKERPPLAQNIFRPGGHSYGGGA--TNARAKGGT 78

[XP_542974.1](https://www.ncbi.nlm.nih.gov/protein/XP_542974.1?report=genbank&log$=protalign&blast_rank=5&RID=0) 1 MRLPKLLTFLLWHLAWLDLEFICTVLGAPDLGQRPQGARPGLAKAEAKERPPLARSVFRPGGHSYGGGA--ANARAKGGT 78

[NP_001296325.1](https://www.ncbi.nlm.nih.gov/protein/NP_001296325.1?report=genbank&log$=protalign&blast_rank=6&RID=0) 1 MRLPKLLTFLLWHLAWLDLEFICTVLGAPDLGQRPQGARPGLAKAEAKERPPLARNIFRPGGHSYGGGA--TSARAKGGT 78

[XP_004014573.2](https://www.ncbi.nlm.nih.gov/protein/XP_004014573.2?report=genbank&log$=protalign&blast_rank=7&RID=0) 1 MRLPKLLTLLLWHLAWLDLEFICTVLGAPDLGQRPQGARPGLAKAEAKERPPLAQNIFRPGGHSYGGGA--TNARAKGGT 78

[XP_530287.4](https://www.ncbi.nlm.nih.gov/protein/XP_530287.4?report=genbank&log$=protalign&blast_rank=8&RID=0) 1 MRLPKLLTLLLWYLAWLDLEFICTVLGAPDLGQRPQGTRPGLAKAEAKERPPLARNVFRPGGHSYGGGAtnANARAKGGT 80

[XP_001099806.2](https://www.ncbi.nlm.nih.gov/protein/XP_001099806.2?report=genbank&log$=protalign&blast_rank=9&RID=0) 1 MRLPKLLTFLLWYLAWLDLEFICTVLGAPDWGQKAQGTRPGLAKAEAKERPPLARNVFRPGGHSYGGGA--ANARAKGGT 78

[XP_003983628.1](https://www.ncbi.nlm.nih.gov/protein/XP_003983628.1?report=genbank&log$=protalign&blast_rank=10&RID=0) 1 MRLPKLLTFLLWHLAWLDLEFICTVLGAPDLGQRPQGARPGLAKAEAKERPPLARSIFRPGGHSYGGGA--TNARAKGGT 78

[XP_017913249.1](https://www.ncbi.nlm.nih.gov/protein/XP_017913249.1?report=genbank&log$=protalign&blast_rank=11&RID=0) 1 MRLPKLLTLLLWHLAWLDLEFICTVLGAPDLGQRPQGARPGLAKAEAKERPPLAQNIFRPGGHSYGGGA--TNARAKGGT 78

[XP_018872428.1](https://www.ncbi.nlm.nih.gov/protein/XP_018872428.1?report=genbank&log$=protalign&blast_rank=12&RID=0) 1 MRLPKLLTFFLWYLAWLDLEFICTVLGAPDLGQRPQGTRPGLAKAEAKERPPLARNVFRPGGHSYGGGAtnANARAKGGT 80

[XP_003779391.1](https://www.ncbi.nlm.nih.gov/protein/XP_003779391.1?report=genbank&log$=protalign&blast_rank=13&RID=0) 1 MRLPKLLTFLLWYLAWLDLEFICTVLGAPDLGQRPQGTRPGLAKAEAKERPPLARNIFRPGGHSYGGGAanANARAKGGT 80

[XP_031998077.1](https://www.ncbi.nlm.nih.gov/protein/XP_031998077.1?report=genbank&log$=protalign&blast_rank=14&RID=0) 1 MRLPKLLTFLLWYLAWLDLEFICTVLGAPDLGQRPQGTRPGLAKAEAKERPPLARNVFRPGGHSYGGGAsnANARAKGGT 80

[NP_000548.2](https://www.ncbi.nlm.nih.gov/protein/NP_000548.2?report=genbank&log$=protalign&blast_rank=0&RID=0) 81 GQTGGLTQPKKDEPKKLPPRPGGPEPKPGHPPQTRQATARTVTPKGQLPGGKAPPKAGSVPSSFLLKKAREPGPPREPKE 160

[NP_032135.2](https://www.ncbi.nlm.nih.gov/protein/NP_032135.2?report=genbank&log$=protalign&blast_rank=1&RID=0) 79 GQ----TQAKKDEPRKMPPRSGGPETKPGPSSQTRQAAARTVTPKGQLPGGKASSKAGSAPSSFLLKKTREPGTPREPKE 154

[XP_003749648.1](https://www.ncbi.nlm.nih.gov/protein/XP_003749648.1?report=genbank&log$=protalign&blast_rank=2&RID=0) 79 GQ----TQAKKDEPRKVPPRASGSETKPGPSPQTRQAAARTVTPKGQLSGGKASAKAGSAPSSFLLKKTREPGTPREPKE 154

[NP_001179202.1](https://www.ncbi.nlm.nih.gov/protein/NP_001179202.1?report=genbank&log$=protalign&blast_rank=3&RID=0) 79 GQTGGLTQPKKDEPKKLPPRSGGPEPKPGHPPQTRQGATRTVTPKGQLPGGKAPSKAGSVPSPFLLKKAREPGSPREPKE 158

[NP_001231226.1](https://www.ncbi.nlm.nih.gov/protein/NP_001231226.1?report=genbank&log$=protalign&blast_rank=4&RID=0) 79 GQTGGLTQPKKDEPKKLPPRSGGLEPKPGHPPQTRQAATRTVTPKGQLPGGKAPQKAGSVPSPFLLKKAREPGSPREPKE 158

[XP_542974.1](https://www.ncbi.nlm.nih.gov/protein/XP_542974.1?report=genbank&log$=protalign&blast_rank=5&RID=0) 79 GQTGGLTQPKKDEPKKLPPRPGSPEPKPGHPTQTRQAAPRTVTPKGQLPGGKAPPKAGSVPGPFLLKKARETGPPPEPKE 158

[NP_001296325.1](https://www.ncbi.nlm.nih.gov/protein/NP_001296325.1?report=genbank&log$=protalign&blast_rank=6&RID=0) 79 GQTGGPTQPKKDEPKKLPPRPGGSEPKPGHPPQTRQAATRTVTPKGQLPGGKAPPKAGSVPSPFLLKKAREPGPPREPKE 158

[XP_004014573.2](https://www.ncbi.nlm.nih.gov/protein/XP_004014573.2?report=genbank&log$=protalign&blast_rank=7&RID=0) 79 GQTGGLTQPKKDEPKKLPSRSGGPESKPGHPPQTRQGATRTVTPKGQLPGGKAPSKAGSVPSPFLLKKAREPGSPREPKE 158

[XP_530287.4](https://www.ncbi.nlm.nih.gov/protein/XP_530287.4?report=genbank&log$=protalign&blast_rank=8&RID=0) 81 GQKGGLTQPKKDEPKKLPPRPGGPEPKPGHPPQTRQTTARTVTPKGQLPGGKAPPKAGSVPSSFLLKKAREPGPPREPKE 160

[XP_001099806.2](https://www.ncbi.nlm.nih.gov/protein/XP_001099806.2?report=genbank&log$=protalign&blast_rank=9&RID=0) 79 GHTGGLTQPKKDEPKKLPPRPGGPEPKPGHPPQTRQATARTVTPKGQLPGGKVPPKAGSVPSSFLLKKAREPGSPREPKE 158

[XP_003983628.1](https://www.ncbi.nlm.nih.gov/protein/XP_003983628.1?report=genbank&log$=protalign&blast_rank=10&RID=0) 79 GQTAGLTQPKKDEPKKLPPRSGGPEPKPGHPSQTRQAATRTVTPKGQLPGGKSPPKAGSVPSPFLLKKARETGPPQEPKE 158

[XP_017913249.1](https://www.ncbi.nlm.nih.gov/protein/XP_017913249.1?report=genbank&log$=protalign&blast_rank=11&RID=0) 79 GQTGGLTQPKKDEPKKLPSRSGGPEPKPGHPPQTRQGATRTVTPKGQLPGGKAPSKAGSVPSPFLLKKAREPGSPREPKE 158

[XP_018872428.1](https://www.ncbi.nlm.nih.gov/protein/XP_018872428.1?report=genbank&log$=protalign&blast_rank=12&RID=0) 81 GQTGGLTQPRKDEPKKLPPRPGGPEPKPGHPPQTRQATARTVTPKGQLPGGKAPPKAGSVPSSFLLKKAREPGPPREPKE 160

[XP_003779391.1](https://www.ncbi.nlm.nih.gov/protein/XP_003779391.1?report=genbank&log$=protalign&blast_rank=13&RID=0) 81 GQTGGLTQPKKDEPKKLSPRPGGPEPKPGHPPQTRQATARTVTPKGQLPGGKAPPKAGSVPSSFLLKKAREPGPPREPKE 160

[XP_031998077.1](https://www.ncbi.nlm.nih.gov/protein/XP_031998077.1?report=genbank&log$=protalign&blast_rank=14&RID=0) 81 GQTGGLTQPKKDEPKK-PPRPGGPEPKPGHPPQTRQATARTVTPKGQLPGGKAPPKAGSVPSSFLLKKAREPGPPREPKE 159

[NP_000548.2](https://www.ncbi.nlm.nih.gov/protein/NP_000548.2?report=genbank&log$=protalign&blast_rank=0&RID=0) 161 PFRPPPITPHEYMLSLYRTLSDADRKGGNSSVKLEAGLANTITSFIDKGQDDRGPVVRKQRYVFDISALEKDGLLGAELR 240

[NP_032135.2](https://www.ncbi.nlm.nih.gov/protein/NP_032135.2?report=genbank&log$=protalign&blast_rank=1&RID=0) 155 PFRPPPITPHEYMLSLYRTLSDADRKGGNSSVKLEAGLANTITSFIDKGQDDRGPAVRKQRYVFDISALEKDGLLGAELR 234

[XP_003749648.1](https://www.ncbi.nlm.nih.gov/protein/XP_003749648.1?report=genbank&log$=protalign&blast_rank=2&RID=0) 155 PFRPPPITPHEYMLSLYRTLSDADRKGGNSSVKLEAGLANTITSFIDKGQDDRGPVVRKQRYVFDISALEKDGLLGAELR 234

[NP_001179202.1](https://www.ncbi.nlm.nih.gov/protein/NP_001179202.1?report=genbank&log$=protalign&blast_rank=3&RID=0) 159 PFRPPPITPHEYMLSLYRTLSDADRKGGNSSVKLEAGLANTITSFIDKGQDDRGPAVRKQRYVFDISALEKDGLLGAELR 238

[NP_001231226.1](https://www.ncbi.nlm.nih.gov/protein/NP_001231226.1?report=genbank&log$=protalign&blast_rank=4&RID=0) 159 PFRPPPITPHEYMLSLYRTLSDADRKGGNSSVKLEAGLANTITSFIDKGQDDRGPAVRKQRYVFDISALEKDGLLAAELR 238

[XP_542974.1](https://www.ncbi.nlm.nih.gov/protein/XP_542974.1?report=genbank&log$=protalign&blast_rank=5&RID=0) 159 PFRPPPITPHEYMLSLYRTLSDADRKGGNSSVKLEAGLANTITSFIDKGQDDRGPVVRKQRYVFDISALEKDGLLGAELR 238

[NP_001296325.1](https://www.ncbi.nlm.nih.gov/protein/NP_001296325.1?report=genbank&log$=protalign&blast_rank=6&RID=0) 159 PFRPPPITPHEYMLSLYRTLSDADRKGGNSSVKLEAGLANTITSFIDKGQDDRGPVVRKQRYVFDISALEKDGLLGAELR 238

[XP_004014573.2](https://www.ncbi.nlm.nih.gov/protein/XP_004014573.2?report=genbank&log$=protalign&blast_rank=7&RID=0) 159 PFRPPPITPHEYMLSLYRTLSDADRKGGNSSVKLEAGLANTITSFIDKGQDDRGPAVRKQRYVFDISALEKDGLLGAELR 238

[XP_530287.4](https://www.ncbi.nlm.nih.gov/protein/XP_530287.4?report=genbank&log$=protalign&blast_rank=8&RID=0) 161 PFRPPPITPHEYMLSLYRTLSDADRKGGNSSVKLEAGLANTITSFIDKGQDDRGPVVRKQRYVFDISALEKNGLLGAELR 240

[XP_001099806.2](https://www.ncbi.nlm.nih.gov/protein/XP_001099806.2?report=genbank&log$=protalign&blast_rank=9&RID=0) 159 PFRPPPITPHEYMLSLYRTLSDADRKGGNSSVKLEAGLANTITSFIDKGQDDRGPVVRKQRYVFDISALEKDGLLGAELR 238

[XP_003983628.1](https://www.ncbi.nlm.nih.gov/protein/XP_003983628.1?report=genbank&log$=protalign&blast_rank=10&RID=0) 159 PFRPPPITPHEYMLSLYRTLSDADRKGGNSSVKLEAGLANTITSFIDKGQDDRGPVVRKQRYVFDISALEKDGLLGAELR 238

[XP_017913249.1](https://www.ncbi.nlm.nih.gov/protein/XP_017913249.1?report=genbank&log$=protalign&blast_rank=11&RID=0) 159 PFRPPPITPHEYMLSLYRTLSDADRKGGNSSVKLEAGLANTITSFIDKGQDDRGPAVRKQRYVFDISALEKDGLLGAELR 238

[XP_018872428.1](https://www.ncbi.nlm.nih.gov/protein/XP_018872428.1?report=genbank&log$=protalign&blast_rank=12&RID=0) 161 PFRPPPITPHEYMLSLYRTLSDADRKGGNSSVKLEAGLANTITSFIDKGQDDRGPVVRKQRYVFDISALEKDGLLGAELR 240

[XP_003779391.1](https://www.ncbi.nlm.nih.gov/protein/XP_003779391.1?report=genbank&log$=protalign&blast_rank=13&RID=0) 161 PFRPPPITPHEYMLSLYRTLSDADRKGGNSSVKLEAGLANTITSFIDKGQDDRGPVVRKQRYVFDISALEKDGLLGAELR 240

[XP_031998077.1](https://www.ncbi.nlm.nih.gov/protein/XP_031998077.1?report=genbank&log$=protalign&blast_rank=14&RID=0) 160 PFRPPPITPHEYMLSLYRTLSDADRKGGNSSVKLEAGLANTITSFIDKGQDDRGPVVRKQRYVFDISALEKDGLLGAELR 239

[NP_000548.2](https://www.ncbi.nlm.nih.gov/protein/NP_000548.2?report=genbank&log$=protalign&blast_rank=0&RID=0) 241 ILRKKPSDTAKPAAPGGGRAAQLKLSSCPSGRQPAALLDVRSVPGLDGSGWEVFDIWKLFRNFKNSAQLCLELEAWERGR 320

[NP_032135.2](https://www.ncbi.nlm.nih.gov/protein/NP_032135.2?report=genbank&log$=protalign&blast_rank=1&RID=0) 235 ILRKKPLDVAKPAVPSSGRVAQLKLSSCPSGRQPAALLDVRSVPGLDGSGWEVFDIWKLFRNFKNSAQLCLELEAWERGR 314

[XP_003749648.1](https://www.ncbi.nlm.nih.gov/protein/XP_003749648.1?report=genbank&log$=protalign&blast_rank=2&RID=0) 235 ILRKKPLDVAKPAVPSSGRVAQLKLSSCPSGRQPAALLDVRSVPGLDGSGWEVFDIWKLFRNFKNSAQLCLELEAWERGR 314

[NP_001179202.1](https://www.ncbi.nlm.nih.gov/protein/NP_001179202.1?report=genbank&log$=protalign&blast_rank=3&RID=0) 239 ILRKKPLDAAKPVAPGSGRAAQLKLSSCPSGRQPAALLDVRSVPGLDGSGWEVFDIWKLFRNFKNSAQLCLELEAWERGR 318

[NP_001231226.1](https://www.ncbi.nlm.nih.gov/protein/NP_001231226.1?report=genbank&log$=protalign&blast_rank=4&RID=0) 239 ILRKKPSDTAKPVAPGIGRAAQLKLSSCPSGRQPAALLDVRSVPGLDGSGWEVFDIWKLFRNFKNSAQLCLELEAWERGR 318

[XP_542974.1](https://www.ncbi.nlm.nih.gov/protein/XP_542974.1?report=genbank&log$=protalign&blast_rank=5&RID=0) 239 ILRKKPSDTAKPVAPSIGRAAQLKLSSCPSGRQPAALLDVRSVPGLDGSGWEVFDIWKLFRNFKNSAQLCLELEAWERGR 318

[NP_001296325.1](https://www.ncbi.nlm.nih.gov/protein/NP_001296325.1?report=genbank&log$=protalign&blast_rank=6&RID=0) 239 ILRKKSSDTAKPGAPSSRRAAQLKLSSCPSGRQPAALLDVRSVPGLDGSGWEVFDIWKLFRNFKNSAQLCLELEAWERGR 318

[XP_004014573.2](https://www.ncbi.nlm.nih.gov/protein/XP_004014573.2?report=genbank&log$=protalign&blast_rank=7&RID=0) 239 ILRKKPLDVAKPVAPGSGRAAQLKLSSCPSGRQPAALLDVRSVPGLDSSGWEVFDIWKLFRSFKNSAQLCLELEAWERGR 318

[XP_530287.4](https://www.ncbi.nlm.nih.gov/protein/XP_530287.4?report=genbank&log$=protalign&blast_rank=8&RID=0) 241 ILRKKPSDTAKPAAPGGGRAAQLKLSSCPSGRQPAALLDVRSVPGLDGSGWEVFDIWKLFRNFKNSAQLCLELEAWERGR 320

[XP_001099806.2](https://www.ncbi.nlm.nih.gov/protein/XP_001099806.2?report=genbank&log$=protalign&blast_rank=9&RID=0) 239 ILRKKPSDTAKSAAPVGGRAAQLKLSSCPSGRQPAALLDVRSVPGLDGSGWEVFDIWKLFRNFKNSAQLCLELEAWERGR 318

[XP_003983628.1](https://www.ncbi.nlm.nih.gov/protein/XP_003983628.1?report=genbank&log$=protalign&blast_rank=10&RID=0) 239 ILRKKPSDAAKPVAPGIRRAAQLKLSSCPSGRQPAALLDVRSVPGLDGSGWEVFDIWKLFRNFKNSAQLCLELEAWERGR 318

[XP_017913249.1](https://www.ncbi.nlm.nih.gov/protein/XP_017913249.1?report=genbank&log$=protalign&blast_rank=11&RID=0) 239 ILRKKPLDAAKPVAPSSGRAAQLKLSSCPSGRQPAALLDVRSVPGLDGSGWEVFDIWKLFRSFKNSAQLCLELEAWERGR 318

[XP_018872428.1](https://www.ncbi.nlm.nih.gov/protein/XP_018872428.1?report=genbank&log$=protalign&blast_rank=12&RID=0) 241 ILRKKPSDTAKPAAPGGGRAAQLKLSSCPSGRQPAALLDVRSVPGLDGSGWEVFDIWKLFRNFKNSAQLCLELEAWERGR 320

[XP_003779391.1](https://www.ncbi.nlm.nih.gov/protein/XP_003779391.1?report=genbank&log$=protalign&blast_rank=13&RID=0) 241 ILRKKPSDTAKPVAPGGGRAAQLKLSSCPSGRQPAALLDVRSVPGLDGSGWEVFDIWKLFRNFKNSAQLCLELEAWERGR 320

[XP_031998077.1](https://www.ncbi.nlm.nih.gov/protein/XP_031998077.1?report=genbank&log$=protalign&blast_rank=14&RID=0) 240 ILRKKPSDTAKPAAPGGGRAAQLKLSSCPSGRQPAALLDVRSVPGLDGSGWEVFDIWKLFRNFKNSAQLCLELEAWERGR 319

[NP_000548.2](https://www.ncbi.nlm.nih.gov/protein/NP_000548.2?report=genbank&log$=protalign&blast_rank=0&RID=0) 321 AVDLRGLGFDRAARQVHEKALFLVFGRTKKRDLFFNEIKARSGQDDKTVYEYLFSQRRKRRAPLATRQGKRPSKNLKARC 400

[NP_032135.2](https://www.ncbi.nlm.nih.gov/protein/NP_032135.2?report=genbank&log$=protalign&blast_rank=1&RID=0) 315 AVDLRGLGFERTARQVHEKALFLVFGRTKKRDLFFNEIKARSGQDDKTVYEYLFSQRRKRRAPLANRQGKRPSKNLKARC 394

[XP_003749648.1](https://www.ncbi.nlm.nih.gov/protein/XP_003749648.1?report=genbank&log$=protalign&blast_rank=2&RID=0) 315 AVDLRGLGFERAARQVHEKALFLVFGRTKKRDLFFNEIKARSGQDDKTVYEYLFSQRRKRRAPLANRQGKRPSKNLKARC 394

[NP_001179202.1](https://www.ncbi.nlm.nih.gov/protein/NP_001179202.1?report=genbank&log$=protalign&blast_rank=3&RID=0) 319 AMDLRGLGFDRAARQVHEKALFLVFGRTKKRDLFFNEIKARSGQDDKTVYEYLFSQRRKRRAPLATRQGKRPSKNPKARC 398

[NP_001231226.1](https://www.ncbi.nlm.nih.gov/protein/NP_001231226.1?report=genbank&log$=protalign&blast_rank=4&RID=0) 319 AVDLRGLGFDRAARQVHEKALFLVFGRTKKRDLFFNEIKARSGQDDKTVYEYLFSQRRKRRAPLATRQGKRPSKNPKARC 398

[XP_542974.1](https://www.ncbi.nlm.nih.gov/protein/XP_542974.1?report=genbank&log$=protalign&blast_rank=5&RID=0) 319 AVDLRGLGFDRAARQVHEKALFLVFGRTKKRDLFFNEIKARSGQDDKTVYEYLFSQRRKRRAPLATRQGKRPSKNPKARC 398

[NP_001296325.1](https://www.ncbi.nlm.nih.gov/protein/NP_001296325.1?report=genbank&log$=protalign&blast_rank=6&RID=0) 319 AVDLRGLGFDRTARQVHEKALFLVFGRTKKRDLFFNEIKARSGQDDKTVYEYLFSQRRKRRAPLATRQGKRPTKNPKARC 398

[XP_004014573.2](https://www.ncbi.nlm.nih.gov/protein/XP_004014573.2?report=genbank&log$=protalign&blast_rank=7&RID=0) 319 AMDLRSLGFDRAARQVHEKALFLVFGRTKKRDLFFNEIKARSGQDDKTVYEYLFSQRRKRRAPLATRQGKRPSKNPKARC 398

[XP_530287.4](https://www.ncbi.nlm.nih.gov/protein/XP_530287.4?report=genbank&log$=protalign&blast_rank=8&RID=0) 321 AVDLRGLGFDRAARQVHEKALFLVFGRTKKRDLFFNEIKARSGQDDKTVYEYLFSQRRKRRAPLATRQGKRPSKNLKARC 400

[XP_001099806.2](https://www.ncbi.nlm.nih.gov/protein/XP_001099806.2?report=genbank&log$=protalign&blast_rank=9&RID=0) 319 AVDLRGLGFDRAARQVHEKALFLVFGRTKKRDLFFNEIKARSGQDDKTVYEYLFSQRRKRRAPLATRQGKRPSKNLKTRC 398

[XP_003983628.1](https://www.ncbi.nlm.nih.gov/protein/XP_003983628.1?report=genbank&log$=protalign&blast_rank=10&RID=0) 319 AMDLRGLGFDRAARQVHEKALFLVFGRTKKRDLFFNEIKARSGQDDKTVYEYLFSQRRKRRAPLATGQGKRPSKNPKARC 398

[XP_017913249.1](https://www.ncbi.nlm.nih.gov/protein/XP_017913249.1?report=genbank&log$=protalign&blast_rank=11&RID=0) 319 AMDLRSLGFDRAARQVHEKALFLVFGRTKKRDLFFNEIKARSGQDDKTVYEYLFSQRRKRRAPLATRQGKRPSKNPKARC 398

[XP_018872428.1](https://www.ncbi.nlm.nih.gov/protein/XP_018872428.1?report=genbank&log$=protalign&blast_rank=12&RID=0) 321 AVDLRGLGFDRAARQVHEKALFLVFGRTKKRDLFFNEIKARSGQDDKTVYEYLFSQRRKRRAPLATRQGKRPSKNLKARC 400

[XP_003779391.1](https://www.ncbi.nlm.nih.gov/protein/XP_003779391.1?report=genbank&log$=protalign&blast_rank=13&RID=0) 321 TVDLRGLGFDRAARQVHEKALFLVFGRTKKRDLFFNEIKARSGQDDKTVYEYLFSQRRKRRAPLATRQGKRPSKNLKARC 400

[XP_031998077.1](https://www.ncbi.nlm.nih.gov/protein/XP_031998077.1?report=genbank&log$=protalign&blast_rank=14&RID=0) 320 AVDLRGLGFDRAARQVHEKALFLVFGRTKKRDLFFNEIKARSGQDDKTVYEYLFSQRRKRRAPLATRQGKRPSKNLKARC 399

[NP_000548.2](https://www.ncbi.nlm.nih.gov/protein/NP_000548.2?report=genbank&log$=protalign&blast_rank=0&RID=0) 401 SRKALHVNFKDMGWDDWIIAPLEYEAFHCEGLCEFPLRSHLEPTNHAVIQTLMNSMDPESTPPTCCVPTRLSPISILFID 480

[NP_032135.2](https://www.ncbi.nlm.nih.gov/protein/NP_032135.2?report=genbank&log$=protalign&blast_rank=1&RID=0) 395 SRKALHVNFKDMGWDDWIIAPLEYEAFHCEGLCEFPLRSHLEPTNHAVIQTLMNSMDPESTPPTCCVPTRLSPISILFID 474

[XP_003749648.1](https://www.ncbi.nlm.nih.gov/protein/XP_003749648.1?report=genbank&log$=protalign&blast_rank=2&RID=0) 395 SRKALHVNFKDMGWDDWIIAPLEYEAFHCEGLCEFPLRSHLEPTNHAVIQTLMNSMDPESTPPTCCVPTRLSPISILFID 474

[NP_001179202.1](https://www.ncbi.nlm.nih.gov/protein/NP_001179202.1?report=genbank&log$=protalign&blast_rank=3&RID=0) 399 SRKALHVNFKDMGWDDWIIAPLEYEAFHCEGLCEFPLRSHLEPTNHAVIQTLMNSMDPESTPPTCCVPTRLSPISILFID 478

[NP_001231226.1](https://www.ncbi.nlm.nih.gov/protein/NP_001231226.1?report=genbank&log$=protalign&blast_rank=4&RID=0) 399 SRKALHVNFKDMGWDDWIIAPLEYEAFHCEGLCEFPLRSHLEPTNHAVIQTLMNSMDPESTPPTCCVPTRLSPISILFID 478

[XP_542974.1](https://www.ncbi.nlm.nih.gov/protein/XP_542974.1?report=genbank&log$=protalign&blast_rank=5&RID=0) 399 SRKALHVNFKDMGWDDWIIAPLEYEAFHCEGLCEFPLRSHLEPTNHAVIQTLMNSMDPESTPPTCCVPTRLSPISILFID 478

[NP_001296325.1](https://www.ncbi.nlm.nih.gov/protein/NP_001296325.1?report=genbank&log$=protalign&blast_rank=6&RID=0) 399 SRKALHVNFKDMGWDDWIIAPLEYEAFHCEGLCEFPLRSHLEPTNHAVIQTLMNSMDPESTPPTCCVPTRLSPISILFID 478

[XP_004014573.2](https://www.ncbi.nlm.nih.gov/protein/XP_004014573.2?report=genbank&log$=protalign&blast_rank=7&RID=0) 399 SRKALHVNFKDMGWDDWIIAPLEYEAFHCEGLCEFPLRSHLEPTNHAVIQTLMNSMDPESTPPTCCVPTRLSPISILFID 478

[XP_530287.4](https://www.ncbi.nlm.nih.gov/protein/XP_530287.4?report=genbank&log$=protalign&blast_rank=8&RID=0) 401 SRKALHVNFKDMGWDDWIIAPLEYEAFHCEGLCEFPLRSHLEPTNHAVIQTLMNSMDPESTPPTCCVPTRLSPISILFID 480

[XP_001099806.2](https://www.ncbi.nlm.nih.gov/protein/XP_001099806.2?report=genbank&log$=protalign&blast_rank=9&RID=0) 399 SRKALHVNFKDMGWDDWIIAPLEYEAFHCEGLCEFPLRSHLEPTNHAVIQTLMNSMDPESTPPTCCVPTRLSPISILFID 478

[XP_003983628.1](https://www.ncbi.nlm.nih.gov/protein/XP_003983628.1?report=genbank&log$=protalign&blast_rank=10&RID=0) 399 SRKALHVNFKDMGWDDWIIAPLEYEAFHCEGLCEFPLRSHLEPTNHAVIQTLMNSMDPESTPPTCCVPTRLSPISILFID 478

[XP_017913249.1](https://www.ncbi.nlm.nih.gov/protein/XP_017913249.1?report=genbank&log$=protalign&blast_rank=11&RID=0) 399 SRKALHVNFKDMGWDDWIIAPLEYEAFHCEGLCEFPLRSHLEPTNHAVIQTLMNSMDPESTPPTCCVPTRLSPISILFID 478

[XP_018872428.1](https://www.ncbi.nlm.nih.gov/protein/XP_018872428.1?report=genbank&log$=protalign&blast_rank=12&RID=0) 401 SRKALHVNFKDMGWDDWIIAPLEYEAFHCEGLCEFPLRSHLEPTNHAVIQTLMNSMDPESTPPTCCVPTRLSPISILFID 480

[XP_003779391.1](https://www.ncbi.nlm.nih.gov/protein/XP_003779391.1?report=genbank&log$=protalign&blast_rank=13&RID=0) 401 SRKALHVNFKDMGWDDWIIAPLEYEAFHCEGLCEFPLRSHLEPTNHAVIQTLMNSMDPESTPPTCCVPTRLSPISILFID 480

[XP_031998077.1](https://www.ncbi.nlm.nih.gov/protein/XP_031998077.1?report=genbank&log$=protalign&blast_rank=14&RID=0) 400 SRKALHVNFKDMGWDDWIIAPLEYEAFHCEGLCEFPLRSHLEPTNHAVIQTLMNSMDPESTPPTCCVPTRLSPISILFID 479

[NP_000548.2](https://www.ncbi.nlm.nih.gov/protein/NP_000548.2?report=genbank&log$=protalign&blast_rank=0&RID=0) 481 SANNVVYKQYEDMVVESCGCR 501

[NP_032135.2](https://www.ncbi.nlm.nih.gov/protein/NP_032135.2?report=genbank&log$=protalign&blast_rank=1&RID=0) 475 SANNVVYKQYEDMVVESCGCR 495

[XP_003749648.1](https://www.ncbi.nlm.nih.gov/protein/XP_003749648.1?report=genbank&log$=protalign&blast_rank=2&RID=0) 475 SANNVVYKQYEDMVVESCGCR 495

[NP_001179202.1](https://www.ncbi.nlm.nih.gov/protein/NP_001179202.1?report=genbank&log$=protalign&blast_rank=3&RID=0) 479 SANNVVYKQYEDMVVESCGCR 499

[NP_001231226.1](https://www.ncbi.nlm.nih.gov/protein/NP_001231226.1?report=genbank&log$=protalign&blast_rank=4&RID=0) 479 SANNVVYKQYEDMVVESCGCR 499

[XP_542974.1](https://www.ncbi.nlm.nih.gov/protein/XP_542974.1?report=genbank&log$=protalign&blast_rank=5&RID=0) 479 SANNVVYKQYEDMVVESCGCR 499

[NP_001296325.1](https://www.ncbi.nlm.nih.gov/protein/NP_001296325.1?report=genbank&log$=protalign&blast_rank=6&RID=0) 479 SANNVVYKQYEDMVVESCGCR 499

[XP_004014573.2](https://www.ncbi.nlm.nih.gov/protein/XP_004014573.2?report=genbank&log$=protalign&blast_rank=7&RID=0) 479 SANNVVYKQYEDMVVESCGCR 499

[XP_530287.4](https://www.ncbi.nlm.nih.gov/protein/XP_530287.4?report=genbank&log$=protalign&blast_rank=8&RID=0) 481 SANNVVYKQYEDMVVESCGCR 501

[XP_001099806.2](https://www.ncbi.nlm.nih.gov/protein/XP_001099806.2?report=genbank&log$=protalign&blast_rank=9&RID=0) 479 SANNVVYKQYEDMVVESCGCR 499

[XP_003983628.1](https://www.ncbi.nlm.nih.gov/protein/XP_003983628.1?report=genbank&log$=protalign&blast_rank=10&RID=0) 479 SANNVVYKQYEDMVVESCGCR 499

[XP_017913249.1](https://www.ncbi.nlm.nih.gov/protein/XP_017913249.1?report=genbank&log$=protalign&blast_rank=11&RID=0) 479 SANNVVYKQYEDMVVESCGCR 499

[XP_018872428.1](https://www.ncbi.nlm.nih.gov/protein/XP_018872428.1?report=genbank&log$=protalign&blast_rank=12&RID=0) 481 SANNVVYKQYEDMVVESCGCR 501

[XP_003779391.1](https://www.ncbi.nlm.nih.gov/protein/XP_003779391.1?report=genbank&log$=protalign&blast_rank=13&RID=0) 481 SANNVVYKQYEDMVVESCGCR 501

[XP_031998077.1](https://www.ncbi.nlm.nih.gov/protein/XP_031998077.1?report=genbank&log$=protalign&blast_rank=14&RID=0) 480 SANNVVYKQYEDMVVESCGCR 500

GDF6

NP_001001557.1 growth/differentiation factor 6 preproprotein [Homo sapiens]

NP_038554.1 growth/differentiation factor 6 preproprotein [Mus musculus]

NP_001013056.1 growth/differentiation factor 6 precursor [Rattus norvegicus]

NP_001001140.1 growth/differentiation factor 6 precursor [Bos taurus]

XP_020944737.1 growth/differentiation factor 6 [Sus scrofa]

XP_038297057.1 growth/differentiation factor 6 isoform X1 [Canis lupus familiaris]

XP_023504500.1 growth/differentiation factor 6 [Equus caballus]

XP_027829270.1 growth/differentiation factor 6 isoform X1 [Ovis aries]

XP_016815191.1 growth/differentiation factor 6 [Pan troglodytes]

XP_001090825.1 growth/differentiation factor 6 [Macaca mulatta]

XP_023104260.1 growth/differentiation factor 6 [Felis catus]

XP_017913739.1 PREDICTED: growth/differentiation factor 6 [Capra hircus]

XP_004047376.1 growth/differentiation factor 6 [Gorilla gorilla gorilla]

XP_002819344.1 growth/differentiation factor 6 [Pongo abelii]

XP_032614364.1 growth/differentiation factor 6 [Hylobates moloch]

[NP_001001557.1](https://www.ncbi.nlm.nih.gov/protein/NP_001001557.1?report=genbank&log$=protalign&blast_rank=0&RID=0) 1 MDTPRVLLSAVFLISFLWDLPGFQQASISSSSSSA-ELGSTKGMRSRKEGKMQRAPRDSDAGREG---QEPQPRPQDEPR 76

[NP_038554.1](https://www.ncbi.nlm.nih.gov/protein/NP_038554.1?report=genbank&log$=protalign&blast_rank=1&RID=0) 1 MDTPRVLLWAIFLISFLWDLPGFQQASISSSSSSStELDSTKDVGNRKEGKMQRTPQESAEGRTP---PEHGLRQKDLRR 77

[NP_001013056.1](https://www.ncbi.nlm.nih.gov/protein/NP_001013056.1?report=genbank&log$=protalign&blast_rank=2&RID=0) 1 MDTPRVLLWAIFLISFLWDLPGFQQASISSSSS--tELDSTKDVENRKGGKMQRTPQESAEGRTP---KEHRPRPNELRR 75

[NP_001001140.1](https://www.ncbi.nlm.nih.gov/protein/NP_001001140.1?report=genbank&log$=protalign&blast_rank=3&RID=0) 1 MDTSRVLLSAVFLISFLWDLPGFQQASISSSSSSA-ELGSAKGMRSRKEGRMPRAPRENATAREPldrQEPPPRPQEEPQ 79

[XP_020944737.1](https://www.ncbi.nlm.nih.gov/protein/XP_020944737.1?report=genbank&log$=protalign&blast_rank=4&RID=0) 1 MDTPKVLLSAVFLISFLWDLPGFQQASISSSSSSA-ELDSAKGMRSRKEGKMPRAPRESATAQAPperQEPQPRPQEEPR 79

[XP_038297057.1](https://www.ncbi.nlm.nih.gov/protein/XP_038297057.1?report=genbank&log$=protalign&blast_rank=5&RID=0) 1 MDAPRVLLSAVFLVGFLWDLPGFQQASIPSSSPSA-QPAAAKGTRSRRDG---RAPRDRAP-REPl---EPEP-----PE 67

[XP_023504500.1](https://www.ncbi.nlm.nih.gov/protein/XP_023504500.1?report=genbank&log$=protalign&blast_rank=6&RID=0) 1 MDTPRVLLSAVFLISFLWDLPGFQQASISSSSS-A-ELGSAKGIRSRREGKMPRAPQESATAQAPlerQEHQPQRQDEPR 78

[XP_027829270.1](https://www.ncbi.nlm.nih.gov/protein/XP_027829270.1?report=genbank&log$=protalign&blast_rank=7&RID=0) 1 MDTSRVLLSAVFLISFLWDLPGFQQASISSSSSSA-ELGSAKGMRSRKEGKMPRAPRENATARAPldrQEPPPRPQEEPQ 79

[XP_016815191.1](https://www.ncbi.nlm.nih.gov/protein/XP_016815191.1?report=genbank&log$=protalign&blast_rank=8&RID=0) 1 MDTPRVLLSAVFLISFLWDLPGFQQASISSSSSSA-ELGSTKGMRSRKEGKMQRAPRDSDAGREG---QEPQPRPQDEPR 76

[XP_001090825.1](https://www.ncbi.nlm.nih.gov/protein/XP_001090825.1?report=genbank&log$=protalign&blast_rank=9&RID=0) 1 MDTPRVLLSAVFLISFLWDLPGFQQASISSSSSSA-ELGSTKGMRSRKEGKMQRAPRESDAGREG---QEPQRRPQDEPQ 76

[XP_023104260.1](https://www.ncbi.nlm.nih.gov/protein/XP_023104260.1?report=genbank&log$=protalign&blast_rank=10&RID=0) 1 MDTPRVLLSAVFLISFLWDLPGFQQASISSSSSSA-ELGSAKGMRSRKEGKIPRAPRDSATAGEPqqrHEPQPRPQDEPR 79

[XP_017913739.1](https://www.ncbi.nlm.nih.gov/protein/XP_017913739.1?report=genbank&log$=protalign&blast_rank=11&RID=0) 1 MDTSRVLLSAVFLISFLWDLPGFQQASISSSSS-A-ELGSAKGMRSRKEGKMPRAPRENATARAPldrQEPPPRPQEEPQ 78

[XP_004047376.1](https://www.ncbi.nlm.nih.gov/protein/XP_004047376.1?report=genbank&log$=protalign&blast_rank=12&RID=0) 1 MDTPRVLLSAVFLISFLWDLPGFQQASISSSSSSA-ELGSTKGMRSRKEGKMQRAPRESDAGREG---QEPQPRPQDEPR 76

[XP_002819344.1](https://www.ncbi.nlm.nih.gov/protein/XP_002819344.1?report=genbank&log$=protalign&blast_rank=13&RID=0) 1 MDTPRVLLSAVFLISFLWDLPGFQQASISSSSSSA-ELGSTKGMRSRKEGKMQRAPRESDAGREG---QEPQPRPQDEPR 76

[XP_032614364.1](https://www.ncbi.nlm.nih.gov/protein/XP_032614364.1?report=genbank&log$=protalign&blast_rank=14&RID=0) 1 MDTPRVLLSAVFLISFLWDLPGFQQASISSSSSSA-ELGSTKGMRSRKEGKMQRAPRESDAAREG---QQPQPPPQDKPR 76

[NP_001001557.1](https://www.ncbi.nlm.nih.gov/protein/NP_001001557.1?report=genbank&log$=protalign&blast_rank=0&RID=0) 77 A----QQPRAQEPPGRGPRVVPHEYMLSIYRTYSIAEKLGINASFFQSSKSANTITSFVDRGLDDLSHTPLRRQKYLFDV 152

[NP_038554.1](https://www.ncbi.nlm.nih.gov/protein/NP_038554.1?report=genbank&log$=protalign&blast_rank=1&RID=0) 78 R--PPGQHQGQEPPGRGLRVVPHEYMLSIYKTYSIAEKLGINASFFQSSKSANTITSFVDRGLDDLSHTPLRRQKYLFDV 155

[NP_001013056.1](https://www.ncbi.nlm.nih.gov/protein/NP_001013056.1?report=genbank&log$=protalign&blast_rank=2&RID=0) 76 R--LPGQSLGQEPPGRGPRVVPHEYMLSIYRTYSIAEKLGINASFFQSSKSANTITSFVDRGLDDLSHTPLRRQKYLFDV 153

[NP_001001140.1](https://www.ncbi.nlm.nih.gov/protein/NP_001001140.1?report=genbank&log$=protalign&blast_rank=3&RID=0) 80 R-rPPQQPEAREPPGRGPRVVPHEYMLSIYRTYSIAEKLGINASFFQSSKSANTITSFVDRGLDDLSHTPLRRQKYLFDV 158

[XP_020944737.1](https://www.ncbi.nlm.nih.gov/protein/XP_020944737.1?report=genbank&log$=protalign&blast_rank=4&RID=0) 80 R-rPPQQPEAQEPPGRGPRVVPHEYMLSIYRTYSIAEKLGINASFFQSSKSANTITSFVDRGLDDLSHTPLRRQKYLFDV 158

[XP_038297057.1](https://www.ncbi.nlm.nih.gov/protein/XP_038297057.1?report=genbank&log$=protalign&blast_rank=5&RID=0) 68 P----------QPPGGAARAVPHDYMLSVYRTHSIAEKLGINASLFQSSGAANTITSFVDRGRDAAARAPLRRQEYVFDV 137

[XP_023504500.1](https://www.ncbi.nlm.nih.gov/protein/XP_023504500.1?report=genbank&log$=protalign&blast_rank=6&RID=0) 79 RrrPPKQHQAQESPGRGPRVVPHEYMLSIYRTYSIAEKLGINASFFQSSKSANTITSFVDRGLDDLSHTPLRRQKYLFDV 158

[XP_027829270.1](https://www.ncbi.nlm.nih.gov/protein/XP_027829270.1?report=genbank&log$=protalign&blast_rank=7&RID=0) 80 R-rPPERREAREPPGRGPRVVPHEYMLSIYRTYSIAETLGINASFSQSSKSANTITSFVDRGLDDLSHTPLRRQKYLFDV 158

[XP_016815191.1](https://www.ncbi.nlm.nih.gov/protein/XP_016815191.1?report=genbank&log$=protalign&blast_rank=8&RID=0) 77 A----QQPRAQEPPGRGPRVVPHEYMLSIYRTYSIAEKLGINASFFQSSKSANMITSFVDRGLDDLSHTPLRRQKYLFDV 152

[XP_001090825.1](https://www.ncbi.nlm.nih.gov/protein/XP_001090825.1?report=genbank&log$=protalign&blast_rank=9&RID=0) 77 A----QQPRAQEPPGRGPRVVPHEYMLSIYRTYSIAEKLGINASFFQSSKSANTITSFVDRGLDDLSHTPLRRQKYLFDV 152

[XP_023104260.1](https://www.ncbi.nlm.nih.gov/protein/XP_023104260.1?report=genbank&log$=protalign&blast_rank=10&RID=0) 80 R-rPPQQPEAQEPPGRGPRVVPHEYMLSIYRTYSIAEKLGINASFFQSSKSANTITSFVDRGLDDLSHTPLRRQKYLFDV 158

[XP_017913739.1](https://www.ncbi.nlm.nih.gov/protein/XP_017913739.1?report=genbank&log$=protalign&blast_rank=11&RID=0) 79 R-rPPERREAREPPGRGPRVVPHEYMLSIYRTYSIAEKLGINASFFQSSKSANTITSFVDRGLDDLSHTPLRRQKYLFDV 157

[XP_004047376.1](https://www.ncbi.nlm.nih.gov/protein/XP_004047376.1?report=genbank&log$=protalign&blast_rank=12&RID=0) 77 A----QQPRAQEPPGRGLRVVPHEYMLSIYRTYSIAEKLGINASFFQSSKSANTITSFVDRGLDDLSHTPLRRQKYLFDV 152

[XP_002819344.1](https://www.ncbi.nlm.nih.gov/protein/XP_002819344.1?report=genbank&log$=protalign&blast_rank=13&RID=0) 77 A----QQPRAQEPPGRGPRVVPHEYMLSIYRTYSIAEKLGINASFFQSSKSANTITSFVDRGLDDLSHTPLRRQKYLFDV 152

[XP_032614364.1](https://www.ncbi.nlm.nih.gov/protein/XP_032614364.1?report=genbank&log$=protalign&blast_rank=14&RID=0) 77 A----QQPRAQEPPGRGPRVVPHEYMLSIYRTYSIAEKLGINASFFQSSKSANTITSFVDRGLDDLSHTPLRRQKYLFDV 152

[NP_001001557.1](https://www.ncbi.nlm.nih.gov/protein/NP_001001557.1?report=genbank&log$=protalign&blast_rank=0&RID=0) 153 SMLSDKEELVGAELRLFRQAPSAPWGPPAGPLHVQLFPCLSPLLLDARTLDPQGAPPAGWEVFDVWQGLRHQPWKQLCLE 232

[NP_038554.1](https://www.ncbi.nlm.nih.gov/protein/NP_038554.1?report=genbank&log$=protalign&blast_rank=1&RID=0) 156 STLSDKEELVGAELRLYRQAPPTPWGLPARPLHLQLFPCLSPLLLDARTLDPQGPTQAGWEVFDVWQGLRPQPWKQLCLE 235

[NP_001013056.1](https://www.ncbi.nlm.nih.gov/protein/NP_001013056.1?report=genbank&log$=protalign&blast_rank=2&RID=0) 154 STLSDKEELVGAELRLYRQAPPTPWGPQTRPLHLQLFPCLSPLLLDSRTLDPQGPTEAGWEVFDVWQVLRPQPWKQLCLE 233

[NP_001001140.1](https://www.ncbi.nlm.nih.gov/protein/NP_001001140.1?report=genbank&log$=protalign&blast_rank=3&RID=0) 159 STLSDKEELVGAELRLFRQAPAAPWGPPAGPLRLQLFACQSPLLLEARSLDPQGAPRPGWEVFDVWRGLRPQPWKQLCLE 238

[XP_020944737.1](https://www.ncbi.nlm.nih.gov/protein/XP_020944737.1?report=genbank&log$=protalign&blast_rank=4&RID=0) 159 STLSDKEELVGAELRLFRQAPAAPGGPPAGPLHVQLFPCLSPLLLDARTLDPQGAPRAGWEVFDVWQGLRHQPRKQLCLE 238

[XP_038297057.1](https://www.ncbi.nlm.nih.gov/protein/XP_038297057.1?report=genbank&log$=protalign&blast_rank=5&RID=0) 138 SALPEREELVGAELRLFRRAPPPPRGPP-RPLRLQLSPCLSPRLLGARTLSPQGPPRAGWEVFDVRPGLRPPPRGPLCLE 216

[XP_023504500.1](https://www.ncbi.nlm.nih.gov/protein/XP_023504500.1?report=genbank&log$=protalign&blast_rank=6&RID=0) 159 STLSDKEELVGAELRLFRQAPAVPWGPPAGPLHVQLFPCLSPLQLDARTLDPQGAPRAGWEVFDVWQGLRHQPWKQLCLE 238

[XP_027829270.1](https://www.ncbi.nlm.nih.gov/protein/XP_027829270.1?report=genbank&log$=protalign&blast_rank=7&RID=0) 159 STLSDKEELVGAELRLFRQAPAVPWGPPAGPLHLQLFACQSPLLLEARSLDPQGAPRPGWEVFDVWRGLRPQPWKQLCLE 238

[XP_016815191.1](https://www.ncbi.nlm.nih.gov/protein/XP_016815191.1?report=genbank&log$=protalign&blast_rank=8&RID=0) 153 SMLSDKEELVGAELRLFRQAPSAPWGPPAGPLHVQLFPCLSPLLLDARTLDPQGAPPAGWEVFDVWQGLRHQPWKQLCLE 232

[XP_001090825.1](https://www.ncbi.nlm.nih.gov/protein/XP_001090825.1?report=genbank&log$=protalign&blast_rank=9&RID=0) 153 SMLSDKEELVGAELRLFRQAPSAPWGPPAGPLHVQLFPCLSPLLLDARTLDPQGAPPAGWEVFDVWQGLRHQPWKQLCLE 232

[XP_023104260.1](https://www.ncbi.nlm.nih.gov/protein/XP_023104260.1?report=genbank&log$=protalign&blast_rank=10&RID=0) 159 STLSDKEELVGAELRLFRQAPATPWGPPAGPLHVQLFPCLSPQLLDARTLDPQGAPRAGWEVFDVWQGLRQQPWKQLCLE 238

[XP_017913739.1](https://www.ncbi.nlm.nih.gov/protein/XP_017913739.1?report=genbank&log$=protalign&blast_rank=11&RID=0) 158 STLSDKEELVGAELRLFRQAPAAPWGPPAGPLHLQLFACQSPLLLEARSLDPQGAPRPGWEVFDVWRGLRPQPWKQLCLE 237

[XP_004047376.1](https://www.ncbi.nlm.nih.gov/protein/XP_004047376.1?report=genbank&log$=protalign&blast_rank=12&RID=0) 153 SMLSDKEELVGAELRLFRQAPSAPWGPPAGPLHVQLFPCLSPLLLDARTLDPQGAPPAGWEVFDVWQGLRHQPWKQLCLE 232

[XP_002819344.1](https://www.ncbi.nlm.nih.gov/protein/XP_002819344.1?report=genbank&log$=protalign&blast_rank=13&RID=0) 153 SMLSDKEELVGAELRLFRQAPSAPWGPPAGPLHVQLFPCLSSLLLDARTLDPQGAPPAGWEVFDVWQGLRRQPWKQLCLE 232

[XP_032614364.1](https://www.ncbi.nlm.nih.gov/protein/XP_032614364.1?report=genbank&log$=protalign&blast_rank=14&RID=0) 153 SMLSDKEELVGAELRLFRQAPSAPWGPPAGPLHVQLFPCLSPQLLDARTLDPQGAPPAGWEVFDVWQGLRHQPWKQLCLE 232

[NP_001001557.1](https://www.ncbi.nlm.nih.gov/protein/NP_001001557.1?report=genbank&log$=protalign&blast_rank=0&RID=0) 233 LRAAW-GELDAGE AEARARGPQQ-PPPPDLRSLGFGRRVRPPQERALLVVFTRSQRKNLFAEMREQLGSA-EAAGP 305

[NP_038554.1](https://www.ncbi.nlm.nih.gov/protein/NP_038554.1?report=genbank&log$=protalign&blast_rank=1&RID=0) 236 LRAAW-GELDAGD TGARARGPQQ-PPPLDLRSLGFGRRVRPPQERALLVVFTRSQRKNLFTEMHEQLGSA-EAA-- 306

[NP_001013056.1](https://www.ncbi.nlm.nih.gov/protein/NP_001013056.1?report=genbank&log$=protalign&blast_rank=2&RID=0) 234 LRAVW-GELDARD SGARPRGPQQ-SPPLDLRSLGFGRRVRPPQERALLVVFTRSQRKNLFTEMHEQLGSA-EAA-- 304

[NP_001001140.1](https://www.ncbi.nlm.nih.gov/protein/NP_001001140.1?report=genbank&log$=protalign&blast_rank=3&RID=0) 239 LRAAWgGEPGAAE DEARAPGPQQ-PPPPDLRSLGFGRRVRTPQERALLVVFSRSQRKTLFAEMREQLGSAtEVVGP 313

[XP_020944737.1](https://www.ncbi.nlm.nih.gov/protein/XP_020944737.1?report=genbank&log$=protalign&blast_rank=4&RID=0) 239 LRAAW-GEPGAGE AEARVPGPQQ-PPSPDLRSLGFGRRVRTPQERALLVVFTRSQRKNLFAEMREQLGSA-EVAGP 311

[XP_038297057.1](https://www.ncbi.nlm.nih.gov/protein/XP_038297057.1?report=genbank&log$=protalign&blast_rank=5&RID=0) 217 LRAAW-AGAGAGA[11]AGAGARGPGP---GPDLRSLGFGRGARRPQERALLVVFSRSRRRSLLAEARGRPGGG------ 293

[XP_023504500.1](https://www.ncbi.nlm.nih.gov/protein/XP_023504500.1?report=genbank&log$=protalign&blast_rank=6&RID=0) 239 LRASW-GELGAGE DEARAPGPQQ-PPPPDLRSLGFGRRVRPPQERALLVVFTRSQRKNLFAEMREQLGSA-EVAGP 311

[XP_027829270.1](https://www.ncbi.nlm.nih.gov/protein/XP_027829270.1?report=genbank&log$=protalign&blast_rank=7&RID=0) 239 LRAAWgGEPGGEE AEAPAPGPQQ-PPPPDLRSLGFGRRVRTPQERALLVVFSRSQRKTLFAEMREQLGSAtEVVGP 313

[XP_016815191.1](https://www.ncbi.nlm.nih.gov/protein/XP_016815191.1?report=genbank&log$=protalign&blast_rank=8&RID=0) 233 LRAAW-GELDAGE AEARARGPQQ-PPPPDLRSLGFGRRVRPPQERALLVVFTRSQRKNLFAEMREQLGSA-EAAGP 305

[XP_001090825.1](https://www.ncbi.nlm.nih.gov/protein/XP_001090825.1?report=genbank&log$=protalign&blast_rank=9&RID=0) 233 LRAAW-GEPDARE AETHARGPQQ-PPPPDLRSLGFGRRVRPPQERALLVVFTRSQRKNLFAEMREQLGSA-EAAGP 305

[XP_023104260.1](https://www.ncbi.nlm.nih.gov/protein/XP_023104260.1?report=genbank&log$=protalign&blast_rank=10&RID=0) 239 LRAAW-GEPGAGE AEARAPGPQQqPPPPDLRSLGFGRRVRPPQERALLVVFTRSQRKNLFAEMREQLGSA-EVAGP 312

[XP_017913739.1](https://www.ncbi.nlm.nih.gov/protein/XP_017913739.1?report=genbank&log$=protalign&blast_rank=11&RID=0) 238 LRAAWgGEPGAEE AEARAPGPQQ-PPPPDLRSLGFGRRVRTPQERALLVVFSRSQRKTLFAEMREQLGSAtEVVGP 312

[XP_004047376.1](https://www.ncbi.nlm.nih.gov/protein/XP_004047376.1?report=genbank&log$=protalign&blast_rank=12&RID=0) 233 LRAAW-GELDAGE AEARARGPQQ-PPPPDLRSLGFGRRVRPPQERALLVVFTRSQRKNLFAEVREQLGSA-EAAGP 305

[XP_002819344.1](https://www.ncbi.nlm.nih.gov/protein/XP_002819344.1?report=genbank&log$=protalign&blast_rank=13&RID=0) 233 LRAAW-GEPDAGE AETRARGPQQ-PPPPDLRSLGFGRRVRPPQERALLVVFTRSQRKNLFAEMREQLGSA-EAADL 305

[XP_032614364.1](https://www.ncbi.nlm.nih.gov/protein/XP_032614364.1?report=genbank&log$=protalign&blast_rank=14&RID=0) 233 LRAAW-GEPEAGE AEARARGPQQ-PPPPDLWSLGFGRRVRPPQERALLVVFTRSQRKNLFAEMREQLGSA-EAAGP 305

[NP_001001557.1](https://www.ncbi.nlm.nih.gov/protein/NP_001001557.1?report=genbank&log$=protalign&blast_rank=0&RID=0) 306 GAGAEGSW PPPSGAPDARPWLPSPGRRRRRTAFASRHGKRHGKKSRLRCSKKPLHVNFKELGWDDWIIAPLEYEAYH 382

[NP_038554.1](https://www.ncbi.nlm.nih.gov/protein/NP_038554.1?report=genbank&log$=protalign&blast_rank=1&RID=0) 307 --GAEGSW PAPSGSPDAGSWLPSPGRRRRRTAFASRHGKRHGKKSRLRCSRKPLHVNFKELGWDDWIIAPLEYEAYH 381

[NP_001013056.1](https://www.ncbi.nlm.nih.gov/protein/NP_001013056.1?report=genbank&log$=protalign&blast_rank=2&RID=0) 305 --GAEGSW PAPSGAPDAGSWLPSPGRRRRRTALSSRHGKRHGKKSRLRCSRKPLHVNFKELGWDDWIIAPLEYEAYH 379

[NP_001001140.1](https://www.ncbi.nlm.nih.gov/protein/NP_001001140.1?report=genbank&log$=protalign&blast_rank=3&RID=0) 314 GAGAEGSG[7]PPPSGTPDAGLWSPSPGRRRRRTAFASRHGKRHGKKSRLRCSKKPLHVNFKELGWDDWIIAPLEYEAYH 397

[XP_020944737.1](https://www.ncbi.nlm.nih.gov/protein/XP_020944737.1?report=genbank&log$=protalign&blast_rank=4&RID=0) 312 GA--EGSW PPPSGTPDAGPWLPSPGRRRRRTAFASRHGKRHGKKSRLRCSKKPLHVNFKELGWDDWIIAPLEYEAYH 386

[XP_038297057.1](https://www.ncbi.nlm.nih.gov/protein/XP_038297057.1?report=genbank&log$=protalign&blast_rank=5&RID=0) 294 --------[1]APAPGTPLAAPWPPPPGRRRR-TALASRHGKRHGKKSRLRCGKKPLHVNFKELGWDDWIIAPLEYEAFH 362

[XP_023504500.1](https://www.ncbi.nlm.nih.gov/protein/XP_023504500.1?report=genbank&log$=protalign&blast_rank=6&RID=0) 312 SGGAEGSW PPPSGSPDVGPWLPSPGRRRRRTAFASRHGKRHGKKSRLRCSKKPLHVNFKELGWDDWIIAPLEYEAYH 388

[XP_027829270.1](https://www.ncbi.nlm.nih.gov/protein/XP_027829270.1?report=genbank&log$=protalign&blast_rank=7&RID=0) 314 GAGAEGSG[1]PPPSGIPDAGPWSPSPGRRRRRTAFASRHGKRHGKKSRLRCSKKPLHVNFKELGWDDWIIAPLEYEAYH 391

[XP_016815191.1](https://www.ncbi.nlm.nih.gov/protein/XP_016815191.1?report=genbank&log$=protalign&blast_rank=8&RID=0) 306 GAGAEGSW PPPSGAPDARPWLPSPGRRRRRTAFASRHGKRHGKKSRLRCSKKPLHVNFKELGWDDWIIAPLEYEAYH 382

[XP_001090825.1](https://www.ncbi.nlm.nih.gov/protein/XP_001090825.1?report=genbank&log$=protalign&blast_rank=9&RID=0) 306 GVGAEGSW PPPSGAPDAGPWLPSPGRRRRRTAFASRHGKRHGKKSRLRCSKKPLHVNFKELGWDDWIIAPLEYEAYH 382

[XP_023104260.1](https://www.ncbi.nlm.nih.gov/protein/XP_023104260.1?report=genbank&log$=protalign&blast_rank=10&RID=0) 313 GAGADGSW PPPSGAPDAGPWLPSPGRRRRRTAFASRHGKRHGKKSRLRCSKKPLHVNFKELGWDDWIIAPLEYEAYH 389

[XP_017913739.1](https://www.ncbi.nlm.nih.gov/protein/XP_017913739.1?report=genbank&log$=protalign&blast_rank=11&RID=0) 313 GAGAEGSG[1]PPPSGIPDAGPWSPSPGRRRRRTAFASRHGKRHGKKSRLRCSKKPLHVNFKELGWDDWIIAPLEYEAYH 390

[XP_004047376.1](https://www.ncbi.nlm.nih.gov/protein/XP_004047376.1?report=genbank&log$=protalign&blast_rank=12&RID=0) 306 GAGAEGSW PPPSGAPDARPWLPSPGRRRRRTAFASRHGKRHGKKSRLRCSKKPLHVNFKELGWDDWIIAPLEYEAYH 382

[XP_002819344.1](https://www.ncbi.nlm.nih.gov/protein/XP_002819344.1?report=genbank&log$=protalign&blast_rank=13&RID=0) 306 GTGAEGSW PPLSGAPDAGPWVPSPGRRRRRTAFASRHGKRHGKKSRLRCSKKPLHVNFKELGWDDWIIAPLEYEAYH 382

[XP_032614364.1](https://www.ncbi.nlm.nih.gov/protein/XP_032614364.1?report=genbank&log$=protalign&blast_rank=14&RID=0) 306 GAGAEGSW PPPSGAPDAGPWLPSPGRRRRRTAFASRHGKRHGKKSRLRCSKKPLHVNFKELGWDDWIIAPLEYEAYH 382

[NP_001001557.1](https://www.ncbi.nlm.nih.gov/protein/NP_001001557.1?report=genbank&log$=protalign&blast_rank=0&RID=0) 383 CEGVCDFPLRSHLEPTNHAIIQTLMNSMDPGSTPPSCCVPTKLTPISILYIDAGNNVVYKQYEDMVVESCGCR 455

[NP_038554.1](https://www.ncbi.nlm.nih.gov/protein/NP_038554.1?report=genbank&log$=protalign&blast_rank=1&RID=0) 382 CEGVCDFPLRSHLEPTNHAIIQTLMNSMDPGSTPPSCCVPTKLTPISILYIDAGNNVVYKQYEDMVVESCGCR 454

[NP_001013056.1](https://www.ncbi.nlm.nih.gov/protein/NP_001013056.1?report=genbank&log$=protalign&blast_rank=2&RID=0) 380 CEGVCDFPLRSHLEPTNHAIIQTLMNSMDPGSTPPSCCVPTKLTPISILYIDAGNNVVYKQYEDMVVESCGCR 452

[NP_001001140.1](https://www.ncbi.nlm.nih.gov/protein/NP_001001140.1?report=genbank&log$=protalign&blast_rank=3&RID=0) 398 CEGVCDFPLRSHLEPTNHAIIQTLMNSMDPGSTPPSCCVPTKLTPISILYIDAGNNVVYKQYEEMVVESCGCR 470

[XP_020944737.1](https://www.ncbi.nlm.nih.gov/protein/XP_020944737.1?report=genbank&log$=protalign&blast_rank=4&RID=0) 387 CEGVCDFPLRSHLEPTNHAIIQTLMNSMDPGSTPPSCCVPTKLTPISILYIDAGNNVVYKQYEDMVVESCGCR 459

[XP_038297057.1](https://www.ncbi.nlm.nih.gov/protein/XP_038297057.1?report=genbank&log$=protalign&blast_rank=5&RID=0) 363 CEGVCDFPLRSHLEPTNHAIIQTLMNSMDPGSTPPSCCVPTRLTPISILYIDAGNNVVYKQYEDMVVESCGCR 435

[XP_023504500.1](https://www.ncbi.nlm.nih.gov/protein/XP_023504500.1?report=genbank&log$=protalign&blast_rank=6&RID=0) 389 CEGVCDFPLRSHLEPTNHAIIQTLMNSMDPGSTPPSCCVPTKLTPISILYIDAGNNVVYKQYEDMVVESCGCR 461

[XP_027829270.1](https://www.ncbi.nlm.nih.gov/protein/XP_027829270.1?report=genbank&log$=protalign&blast_rank=7&RID=0) 392 CEGVCDFPLRSHLEPTNHAIIQTLMNSMDPGSTPPSCCVPTKLTPISILYIDAGNNVVYKQYEEMVVESCGCR 464

[XP_016815191.1](https://www.ncbi.nlm.nih.gov/protein/XP_016815191.1?report=genbank&log$=protalign&blast_rank=8&RID=0) 383 CEGVCDFPLRSHLEPTNHAIIQTLMNSMDPGSTPPSCCVPTKLTPISILYIDAGNNVVYKQYEDMVVESCGCR 455

[XP_001090825.1](https://www.ncbi.nlm.nih.gov/protein/XP_001090825.1?report=genbank&log$=protalign&blast_rank=9&RID=0) 383 CEGVCDFPLRSHLEPTNHAIIQTLMNSMDPGSTPPSCCVPTKLTPISILYIDAGNNVVYKQYEDMVVESCGCR 455

[XP_023104260.1](https://www.ncbi.nlm.nih.gov/protein/XP_023104260.1?report=genbank&log$=protalign&blast_rank=10&RID=0) 390 CEGVCDFPLRSHLEPTNHAIIQTLMNSMDPGSTPPSCCVPTKLTPISILYIDAGNNVVYKQYEDMVVESCGCR 462

[XP_017913739.1](https://www.ncbi.nlm.nih.gov/protein/XP_017913739.1?report=genbank&log$=protalign&blast_rank=11&RID=0) 391 CEGVCDFPLRSHLEPTNHAIIQTLMNSMDPGSTPPSCCVPTKLTPISILYIDAGNNVVYKQYEEMVVESCGCR 463

[XP_004047376.1](https://www.ncbi.nlm.nih.gov/protein/XP_004047376.1?report=genbank&log$=protalign&blast_rank=12&RID=0) 383 CEGVCDFPLRSHLEPTNHAIIQTLMNSMDPGSTPPSCCVPTKLTPISILYIDAGNNVVYKQYEDMVVESCGCR 455

[XP_002819344.1](https://www.ncbi.nlm.nih.gov/protein/XP_002819344.1?report=genbank&log$=protalign&blast_rank=13&RID=0) 383 CEGVCDFPLRSHLEPTNHAIIQTLMNSMDPGSTPPSCCVPTKLTPISILYIDAGNNVVYKQYEDMVVESCGCR 455

[XP_032614364.1](https://www.ncbi.nlm.nih.gov/protein/XP_032614364.1?report=genbank&log$=protalign&blast_rank=14&RID=0) 383 CEGVCDFPLRSHLEPTNHAIIQTLMNSMDPGSTPPSCCVPTKLTPISILYIDAGNNVVYKQYEDMVVESCGCR 455

GDF7

NP_878248.2 growth/differentiation factor 7 preproprotein [Homo sapiens]

NP_001299805.1 growth/differentiation factor 7 isoform 1 preproprotein [Mus musculus]

XP_006239940.1 growth/differentiation factor 7 isoform X1 [Rattus norvegicus]

NP_001193030.1 growth/differentiation factor 7 precursor [Bos taurus]

XP_003354958.1 growth/differentiation factor 7 isoform X1 [Sus scrofa]

XP_038309370.1 LOW QUALITY PROTEIN: growth/differentiation factor 7 [Canis lupus familiaris]

XP_023475218.1 growth/differentiation factor 7 [Equus caballus]

XP_027824132.2 growth/differentiation factor 7 [Ovis aries]

XP_003308955.2 growth/differentiation factor 7 [Pan troglodytes]

XP_001096970.2 growth/differentiation factor 7 [Macaca mulatta]

XP_023107842.1 growth/differentiation factor 7 [Felis catus]

XP_017910266.1 PREDICTED: growth/differentiation factor 7 [Capra hircus]

XP_004028950.1 growth/differentiation factor 7 [Gorilla gorilla gorilla]

XP_002812295.2 growth/differentiation factor 7 [Pongo abelii]

XP_032028233.1 growth/differentiation factor 7 [Hylobates moloch]

[NP_878248.2](https://www.ncbi.nlm.nih.gov/protein/NP_878248.2?report=genbank&log$=protalign&blast_rank=0&RID=0) 1 MDLSAAAALCLWLLSACRPRDGL--EAAAVLRAAGAGPVRSPGG--GGGG--GGggRTLAQAAGAAAVPAAAVPRARAAR 74

[NP_001299805.1](https://www.ncbi.nlm.nih.gov/protein/NP_001299805.1?report=genbank&log$=protalign&blast_rank=1&RID=0) 1 MDLSAAAALCLWLLSACRPRDGL--EAAAVLRAAGAGPAWSPGG--GGGG------RTLARAPGPSALQAAAVPGPRAVR 70

[XP_006239940.1](https://www.ncbi.nlm.nih.gov/protein/XP_006239940.1?report=genbank&log$=protalign&blast_rank=2&RID=0) 1 MDLSAAAALCLWLLSACRPRDGL--EAAAVLRAAGAGPAWSPGG--GGGG------RTLAPAPGPSALQAAAVPGPRAVR 70

[NP_001193030.1](https://www.ncbi.nlm.nih.gov/protein/NP_001193030.1?report=genbank&log$=protalign&blast_rank=3&RID=0) 1 MDLSAAAALCLWLLSACRPRDGL--EAAAVLRAAGAGPAESPGG--GGGG--SG--TTLAAAEGTSAALAAASPGPRGAR 72

[XP_003354958.1](https://www.ncbi.nlm.nih.gov/protein/XP_003354958.1?report=genbank&log$=protalign&blast_rank=4&RID=0) 1 MDLSAAAALCLWLLSACRPRDGL--EAAAVLRAAGAGPAGSPGG--GGGG--R---RTLAAAAGASAAPAAAAPGPRAAR 71

[XP_038309370.1](https://www.ncbi.nlm.nih.gov/protein/XP_038309370.1?report=genbank&log$=protalign&blast_rank=5&RID=0) 1 MDLSAAAALCLWLLSACRPRDGLglEAAAVLRAAGAGX-GPGGRggGGGG--GG--RALAPAAGVSAAPAAAAPGARAAR 75

[XP_023475218.1](https://www.ncbi.nlm.nih.gov/protein/XP_023475218.1?report=genbank&log$=protalign&blast_rank=6&RID=0) 1 MDLSAAAALCLWLLSACRPRDGL--EAAAVLRAAGAGPAGSPGG--GGGG--GG--RTLAAAAGASTGPAAAAPGARAAR 72

[XP_027824132.2](https://www.ncbi.nlm.nih.gov/protein/XP_027824132.2?report=genbank&log$=protalign&blast_rank=7&RID=0) 1 MDLSAAAALCLWLLSACRPRDGL--EAAAVLRAAGAGPAESPGG--GGGGggGG--TTLAAAAGASAALAAASPEPRGAR 74

[XP_003308955.2](https://www.ncbi.nlm.nih.gov/protein/XP_003308955.2?report=genbank&log$=protalign&blast_rank=8&RID=0) 1 MDLSAAAALCLWLLSACRPRDGL--EAAAVLRAAGAGPVRSPGG--GGGG--G---RTLAQAAGAAAVPAAAVPRARAAR 71

[XP_001096970.2](https://www.ncbi.nlm.nih.gov/protein/XP_001096970.2?report=genbank&log$=protalign&blast_rank=9&RID=0) 1 MDLSAAAALCLWLLSACRPRDGL--EAAAVLRAAGAGPVRSPGG--GGGG--GGggRTLAQAAGAAAVPAAAVSRARAPR 74

[XP_023107842.1](https://www.ncbi.nlm.nih.gov/protein/XP_023107842.1?report=genbank&log$=protalign&blast_rank=10&RID=0) 1 MDLSAAAALCLWLLSACRPRDGL--EAAAVLRAAGAGPVGSPGGdgGSGS--GG--RTLAPAAGVSAAPAAAAPGVRAAR 74

[XP_017910266.1](https://www.ncbi.nlm.nih.gov/protein/XP_017910266.1?report=genbank&log$=protalign&blast_rank=11&RID=0) 1 MDLSAAAALCLWLLSACRPRDGL--EAAAVLRAAGAGPAESPGG--GGGG--GG--TTLAAAAGASAAPAAASPGPRGAR 72

[XP_004028950.1](https://www.ncbi.nlm.nih.gov/protein/XP_004028950.1?report=genbank&log$=protalign&blast_rank=12&RID=0) 1 MDLSAAAALCLWLLSACRPRDGL--EAAAVLRAAGAGPVRSPGG--GGGG--G---RTLAQAAGAAAVPAAAVPRARAAR 71

[XP_002812295.2](https://www.ncbi.nlm.nih.gov/protein/XP_002812295.2?report=genbank&log$=protalign&blast_rank=13&RID=0) 1 MDLSAAAALCLWLLSACRPRDGL--EAAAVLRAAGAGPVRSPGG--GGGG--G---RTLAQAAGASAVPAAAIPRARAAR 71

[XP_032028233.1](https://www.ncbi.nlm.nih.gov/protein/XP_032028233.1?report=genbank&log$=protalign&blast_rank=14&RID=0) 1 MDLNAAAALCLWLLSACRPRDGL--EAAAVLRAAGVGPVRSPGG--GGGG--E---RTLAQAAGAATVPAAAVPRARAAR 71

[NP_878248.2](https://www.ncbi.nlm.nih.gov/protein/NP_878248.2?report=genbank&log$=protalign&blast_rank=0&RID=0) 75 RAAGSG FRNGSVVPHHFMMSLYRSLAGRAPAGAAAVSASG---HGRADTITGFTDQATQDESAA-ETGQSFLFDVSS 147

[NP_001299805.1](https://www.ncbi.nlm.nih.gov/protein/NP_001299805.1?report=genbank&log$=protalign&blast_rank=1&RID=0) 71 RAAGSG FRNGSVVPHHFMMSLYRSLAGRAP----VAAASG---HGRVDTITGFTDQATQDETAAaEPGQSFLFDVSS 140

[XP_006239940.1](https://www.ncbi.nlm.nih.gov/protein/XP_006239940.1?report=genbank&log$=protalign&blast_rank=2&RID=0) 71 RAAGSG FRNGSVVPHHFMMSLYRSLAGRAP----AAATSG---HGRVDTITGFTDQATQEESAA-EPGQSFLFDVSS 139

[NP_001193030.1](https://www.ncbi.nlm.nih.gov/protein/NP_001193030.1?report=genbank&log$=protalign&blast_rank=3&RID=0) 73 RATGSG FRNGSVVPHQFMMSLYRSLTGRTPAGPVAVSTSGsgrHGRADTVTGFADQAIPDESAA-QTGLNFLFDVSS 148

[XP_003354958.1](https://www.ncbi.nlm.nih.gov/protein/XP_003354958.1?report=genbank&log$=protalign&blast_rank=4&RID=0) 72 RAAGSG FRNGSVVPHQFMMSLYRSLAGRAPTGAVAASTSGagrHGRADTITGFADQATQDESAA-ETGQRFLFDVSS 147

[XP_038309370.1](https://www.ncbi.nlm.nih.gov/protein/XP_038309370.1?report=genbank&log$=protalign&blast_rank=5&RID=0) 76 RAAGSG[8]FGNGSVVPHQFMMSLYRNLAGRAPAGGAAASTSGsgrRGRADTITGFADQANQDDPPA-ETGQSFLFDVSS 159

[XP_023475218.1](https://www.ncbi.nlm.nih.gov/protein/XP_023475218.1?report=genbank&log$=protalign&blast_rank=6&RID=0) 73 RAASSG FRNGSVVPHQFMMSLYRSLAGRAPAGAAAASTSGsgrHGRADTITGFADQATQDESAA-ETGQSFLFDVSS 148

[XP_027824132.2](https://www.ncbi.nlm.nih.gov/protein/XP_027824132.2?report=genbank&log$=protalign&blast_rank=7&RID=0) 75 RATGSG FRNGSVVPHQFMMSLYRSLAGRTPAGPVAASTSGsgrHGRADTVTGFADQATPDESAA-ETGLNFLFDVSS 150

[XP_003308955.2](https://www.ncbi.nlm.nih.gov/protein/XP_003308955.2?report=genbank&log$=protalign&blast_rank=8&RID=0) 72 RAAGSG FRNGSVVPHHFMMSLYRSLAGRAPAGAAAVSASG---HGRADTIIGFTDQATQDESAA-ETGQSFLFDVSS 144

[XP_001096970.2](https://www.ncbi.nlm.nih.gov/protein/XP_001096970.2?report=genbank&log$=protalign&blast_rank=9&RID=0) 75 RAAGSG FRNGSVVPHHFMMSLYRSLAGRAPARAAAVSASG---HGRADTITGFTDQATQDESAA-ETGQSFLFDVSS 147

[XP_023107842.1](https://www.ncbi.nlm.nih.gov/protein/XP_023107842.1?report=genbank&log$=protalign&blast_rank=10&RID=0) 75 RAAGSG FRNGSVVPHQFMMSLYRNLAGRAPAGAAAASTSGsgrHGRADTITGFADQANQDESAA-ETGQSFLFDVSS 150

[XP_017910266.1](https://www.ncbi.nlm.nih.gov/protein/XP_017910266.1?report=genbank&log$=protalign&blast_rank=11&RID=0) 73 RATGSG FRNGSVVPHQFMMSLYRSLAGRTPAGPVAASTSGsgrHGRADTVTGFADQATPDESAA-ETGLNFLFDVSS 148

[XP_004028950.1](https://www.ncbi.nlm.nih.gov/protein/XP_004028950.1?report=genbank&log$=protalign&blast_rank=12&RID=0) 72 RAAVSG FRNGSVVPHHFMMSLYRSLAGRAPAGAAAVSASG---HGRADTITGFTDQATQDESAA-ETGQSFLFDVSS 144

[XP_002812295.2](https://www.ncbi.nlm.nih.gov/protein/XP_002812295.2?report=genbank&log$=protalign&blast_rank=13&RID=0) 72 RAAGSG FRNGSVVPHHFMMSLYRSLAGRAPAGAATVSASG---HGRADTITGFTDQATQDESAA-ETGQSFLFDVSS 144

[XP_032028233.1](https://www.ncbi.nlm.nih.gov/protein/XP_032028233.1?report=genbank&log$=protalign&blast_rank=14&RID=0) 72 RAAGSG FRNGSVVPHHFMMSLYRSLAGRAPAGAAAVSASG---HGRADTITGFTDQATQDESAA-ETGQSFLFDVSS 144

[NP_878248.2](https://www.ncbi.nlm.nih.gov/protein/NP_878248.2?report=genbank&log$=protalign&blast_rank=0&RID=0) 148 LNDADEVVGAELRVLRRGSPESGPGSWTSPPLLLL-STCPGAARAPRLLYSRAAEPLVGQRWEAFDVADAMRRHRREPRP 226

[NP_001299805.1](https://www.ncbi.nlm.nih.gov/protein/NP_001299805.1?report=genbank&log$=protalign&blast_rank=1&RID=0) 141 LSEADEVVNAELRVLRRRSPEPDRDSATLLPRLLL-STCPDEAGTAHLLHSRAAEPLGGARWEAFDVTDAVQSHRRWPRA 219

[XP_006239940.1](https://www.ncbi.nlm.nih.gov/protein/XP_006239940.1?report=genbank&log$=protalign&blast_rank=2&RID=0) 140 LSDSDEVVNAELRVLRRRSPEPDRDSATLPPLLLL-STCPDEAGTAHLLHSRAAEPLDSARWEAFDVTDAVQSHRRWPRT 218

[NP_001193030.1](https://www.ncbi.nlm.nih.gov/protein/NP_001193030.1?report=genbank&log$=protalign&blast_rank=3&RID=0) 149 LPDADEVLGAELRVLRRESGARGPGSASPP--LLLlSTCPSAASAPRLLHSRAAEFLDAARWEVFDVADALRRHRREPRT 226

[XP_003354958.1](https://www.ncbi.nlm.nih.gov/protein/XP_003354958.1?report=genbank&log$=protalign&blast_rank=4&RID=0) 148 LPDADEVVGAELRVLRRESPEPGPGRATSP--LLLlSTCPGAARAPRLLHSRAAEHLEGARWEVFDVVDAVRRHRWEPRA 225

[XP_038309370.1](https://www.ncbi.nlm.nih.gov/protein/XP_038309370.1?report=genbank&log$=protalign&blast_rank=5&RID=0) 160 LPDADEVVAAELRVLRHESPGPGAGRAAAPPLLLL-STCPRAARAPRLLHSRAAPPLARARWEVFDVADAVRRHRREPRA 238

[XP_023475218.1](https://www.ncbi.nlm.nih.gov/protein/XP_023475218.1?report=genbank&log$=protalign&blast_rank=6&RID=0) 149 LSDADEVVGAELRVLRREFPEPSSGSAIPQPLLLLlSTCPGAASAPLLLHSRAAEPLDGARWEVFDVADAVRRHRREPRA 228

[XP_027824132.2](https://www.ncbi.nlm.nih.gov/protein/XP_027824132.2?report=genbank&log$=protalign&blast_rank=7&RID=0) 151 LPDADEVLGAELRVLRRESGARGPGSASLA--LLLlSTCPNAARAPRLLHSRAAESLDTARWEVFDVADALRRHRREPRP 228

[XP_003308955.2](https://www.ncbi.nlm.nih.gov/protein/XP_003308955.2?report=genbank&log$=protalign&blast_rank=8&RID=0) 145 LNDADEVVGAELRVLRRGSPESGPGSWTSPPLLQL-STCPGAARAPRLLYSRAAEPLVGQRWEAFDVADAMRRHHREPRP 223

[XP_001096970.2](https://www.ncbi.nlm.nih.gov/protein/XP_001096970.2?report=genbank&log$=protalign&blast_rank=9&RID=0) 148 LNDADEVVGAELRVLRRGSPEPGPGSSTSPPLLLL-STCPGAARAPRLLYSRAAEPLVGQRWEVFDVADAMRRHRREPRP 226

[XP_023107842.1](https://www.ncbi.nlm.nih.gov/protein/XP_023107842.1?report=genbank&log$=protalign&blast_rank=10&RID=0) 151 LPDADEVVGAELRVLRRESPELGPGSATPLPLLLL-STCPGAARAPRLLHSRAAEPLAGARWEVFDVADAVRRHRQEPHA 229

[XP_017910266.1](https://www.ncbi.nlm.nih.gov/protein/XP_017910266.1?report=genbank&log$=protalign&blast_rank=11&RID=0) 149 LPDADEVLGAELRVLRRESGARGPGSASLP--LLLlSTCPNAARAPRLLHSRAAESLDTARWEVFDVADALRRHRREPRP 226

[XP_004028950.1](https://www.ncbi.nlm.nih.gov/protein/XP_004028950.1?report=genbank&log$=protalign&blast_rank=12&RID=0) 145 LNDADEVVGAELRVLRRGSPESGPGSWTSPPLLLL-STCPGAARAPRLLYSRAAEPLVGQRWEAFDVADAMRRHRREPRP 223

[XP_002812295.2](https://www.ncbi.nlm.nih.gov/protein/XP_002812295.2?report=genbank&log$=protalign&blast_rank=13&RID=0) 145 LNDADEVVGAELRVLRRGSPEPGPGSWTSPPVLLL-STCPGAARAPRLLYSRAAEPLVGQRWEAFDVADAMRRHRREPRH 223

[XP_032028233.1](https://www.ncbi.nlm.nih.gov/protein/XP_032028233.1?report=genbank&log$=protalign&blast_rank=14&RID=0) 145 LNDADEVVGAELRVLRRGSPEPGPGSWTSPPLLLL-STCPGAARAPRLLYSRAAEPVVGQRWEAFDVADAMRRHRREPRP 223

[NP_878248.2](https://www.ncbi.nlm.nih.gov/protein/NP_878248.2?report=genbank&log$=protalign&blast_rank=0&RID=0) 227 PRAFCLLLRAVAGPVPSPLALRRLGFGWPGGGG SAAEERAVLVVSSRTQRKESLFREIRAQARALGAALASEPLPDP 303

[NP_001299805.1](https://www.ncbi.nlm.nih.gov/protein/NP_001299805.1?report=genbank&log$=protalign&blast_rank=1&RID=0) 220 SRKFCLVLRAVTASESSPLALRRLGFGWPGGGD[3]TAAEERALLVISSRTQRKESLFREIRAQARALRA--AAEPPPDP 297

[XP_006239940.1](https://www.ncbi.nlm.nih.gov/protein/XP_006239940.1?report=genbank&log$=protalign&blast_rank=2&RID=0) 219 SRKFCLVLRAVTGAESSPLALRRLGFGWPGGGD[3]TAAEERALLVISSRTHRKESLFREIRAQARALRA--AAELPPDP 296

[NP_001193030.1](https://www.ncbi.nlm.nih.gov/protein/NP_001193030.1?report=genbank&log$=protalign&blast_rank=3&RID=0) 227 SRAFCLSLSGVVGSARVPLALRPLGFGLRGGGG AAAEERALLVVSSRTQRKESLFREIRSQARALGAALAVESRPDS 303

[XP_003354958.1](https://www.ncbi.nlm.nih.gov/protein/XP_003354958.1?report=genbank&log$=protalign&blast_rank=4&RID=0) 226 TSAFCLLLRGVVGPGRDPLALRLLGFGSSGGDG AAEEERALLVVSSRTQRKESLFREIRAQARAFGAALAAEPPPDP 302

[XP_038309370.1](https://www.ncbi.nlm.nih.gov/protein/XP_038309370.1?report=genbank&log$=protalign&blast_rank=5&RID=0) 239 ARAFCLRLRSVAGPSREPLALRGLGFGSRGARG AGAEERALLVVSSRAHRKGSLFRE----ARALGAALAAEPPPDP 311

[XP_023475218.1](https://www.ncbi.nlm.nih.gov/protein/XP_023475218.1?report=genbank&log$=protalign&blast_rank=6&RID=0) 229 TRVFCLLLRAAAGPARGPLALQLLGFGSRGGGG AAAEERALLVVSSRTQRKESLFREIRAQARALGAPLAAEPPPDP 305

[XP_027824132.2](https://www.ncbi.nlm.nih.gov/protein/XP_027824132.2?report=genbank&log$=protalign&blast_rank=7&RID=0) 229 SRAFCLSLRGVVGSARVPLALRLLGFGLRGGGG[5]AAAEERALLVVSSRTQRKESLFREIRSQARALGAALAAESRPDP 310

[XP_003308955.2](https://www.ncbi.nlm.nih.gov/protein/XP_003308955.2?report=genbank&log$=protalign&blast_rank=8&RID=0) 224 PRAFCLLLRAVAGPVPSPLALRRLGFGWPGGGG SAAEERALLVVSSRTQRKESLFREIRAQARALGAALASEPLPDP 300

[XP_001096970.2](https://www.ncbi.nlm.nih.gov/protein/XP_001096970.2?report=genbank&log$=protalign&blast_rank=9&RID=0) 227 PRAFCLLLRAVTGPVRSPLALRRLGFRWPGGGG SAPEERALLVVSSRTQRKESLFREMRAQARALGAALAAQPPPDP 303

[XP_023107842.1](https://www.ncbi.nlm.nih.gov/protein/XP_023107842.1?report=genbank&log$=protalign&blast_rank=10&RID=0) 230 TRAFCLLLRSVAGPSQGPLALRLLGFGSRGRDG AAAEERALLVVSSRTQRKGSLFREIRAQARALGAALAAEPPRDP 306

[XP_017910266.1](https://www.ncbi.nlm.nih.gov/protein/XP_017910266.1?report=genbank&log$=protalign&blast_rank=11&RID=0) 227 SRAFCLSLRGVVGSARVPLALRLLGFGLRGGGG[1]AAAEERALLVVSSRTQRKESLFREIRSQARALGAARAAESRPDP 304

[XP_004028950.1](https://www.ncbi.nlm.nih.gov/protein/XP_004028950.1?report=genbank&log$=protalign&blast_rank=12&RID=0) 224 PRAFCLLLRAVAGPVPSPLALRRLGFGWPGGGG SAAEERSLLVVSSRTQRKESLFREIRAQARALGAAVASEPLPDP 300

[XP_002812295.2](https://www.ncbi.nlm.nih.gov/protein/XP_002812295.2?report=genbank&log$=protalign&blast_rank=13&RID=0) 224 PRAFCLLLRAVAGPVRSPLALRRLGFGWSGGGG SAAEERALLVVSSRTQRKESLFREIRAQARALGAALASEPPPDP 300

[XP_032028233.1](https://www.ncbi.nlm.nih.gov/protein/XP_032028233.1?report=genbank&log$=protalign&blast_rank=14&RID=0) 224 PRAFCLLLRAVAGPVRSPLALRRLGFGWPGGGG SAAEERALLVVSSRTQRKESLFREIRAQARALGAALASEQPPDP 300

[NP_878248.2](https://www.ncbi.nlm.nih.gov/protein/NP_878248.2?report=genbank&log$=protalign&blast_rank=0&RID=0) 304 GTGTASPRAVIGGRRRRRTALAGTRTAQGS GGG AGRGHGRRGRSRCSRKPLHVDFKELGWDDWIIAPLDYEAY 376

[NP_001299805.1](https://www.ncbi.nlm.nih.gov/protein/NP_001299805.1?report=genbank&log$=protalign&blast_rank=1&RID=0) 298 GPGAGSRKANLGGRRRRRTALAGTRGAQGS[4]GGG[13]AGRGHGRRGRSRCSRKSLHVDFKELGWDDWIIAPLDYEAY 387

[XP_006239940.1](https://www.ncbi.nlm.nih.gov/protein/XP_006239940.1?report=genbank&log$=protalign&blast_rank=2&RID=0) 297 GLGAGSRKATPGGRRRRRTALAGTRGAQGS GGG[13]AGRGHGRRGRSRCSRKPLHVDFKELGWDDWIIAPLDYEAY 382

[NP_001193030.1](https://www.ncbi.nlm.nih.gov/protein/NP_001193030.1?report=genbank&log$=protalign&blast_rank=3&RID=0) 304 RPGVGSPTAVIGGRRRRRTALAGARAAQGS GGG AGRGHGRRGRSRCSRKPLHVDFKELGWDDWIIAPLDYEAY 376

[XP_003354958.1](https://www.ncbi.nlm.nih.gov/protein/XP_003354958.1?report=genbank&log$=protalign&blast_rank=4&RID=0) 303 GPAIGSSTAVIGGRRRRRTALAGARAAQDS GAG GGRGHGRRSRSRCSRKPLHVDFKELGWDDWIIAPLDYEAY 375

[XP_038309370.1](https://www.ncbi.nlm.nih.gov/protein/XP_038309370.1?report=genbank&log$=protalign&blast_rank=5&RID=0) 312 GPGPRSPAAVTGGRRRRRTALAGARPAQGG GGG AGRGHGRRGRSRCSRRPLHVDFKELGWDDWIIAPLDYEAY 384

[XP_023475218.1](https://www.ncbi.nlm.nih.gov/protein/XP_023475218.1?report=genbank&log$=protalign&blast_rank=6&RID=0) 306 GPGTGSPTAVIGGRRRRRTALAGTRAAQGS GGG AGRGHGRRGRSRCSRKPLHVDFKELGWDDWIIAPLDYEAY 378

[XP_027824132.2](https://www.ncbi.nlm.nih.gov/protein/XP_027824132.2?report=genbank&log$=protalign&blast_rank=7&RID=0) 311 GPGVGSPTAVIGGRRRRRTALAGARAAQGS GGG AGRGQGRRGRSRCSRKPLHVDFKELGWDDWIIAPLDYEAY 383

[XP_003308955.2](https://www.ncbi.nlm.nih.gov/protein/XP_003308955.2?report=genbank&log$=protalign&blast_rank=8&RID=0) 301 GTRTASPRAVIGGRRRRRTALAGTRTAQGS GGG AGRGHGRRGRSRCSRKPLHVDFKELGWDDWIIAPLDYEAY 373

[XP_001096970.2](https://www.ncbi.nlm.nih.gov/protein/XP_001096970.2?report=genbank&log$=protalign&blast_rank=9&RID=0) 304 GTGTGSPRAVTAGRRRRRTALAGTRTAQGS GGG AGRGHGRRGRSRCSRKPLHVDFKELGWDDWIIAPLDYEAY 376

[XP_023107842.1](https://www.ncbi.nlm.nih.gov/protein/XP_023107842.1?report=genbank&log$=protalign&blast_rank=10&RID=0) 307 GPGTGSPTAVISGRRRRRTALTGARATQGS[2]GGG AGRGHGRRGRTRCSRRPLHVDFKELGWDDWIIAPLDYEAY 381

[XP_017910266.1](https://www.ncbi.nlm.nih.gov/protein/XP_017910266.1?report=genbank&log$=protalign&blast_rank=11&RID=0) 305 GPGVGSPTAVIGGRRRRRTALAGARAAQGS GGG AGRGHGRRVRSRCSRKPLHVDFKELGWDDWIIAPLDYEAY 377

[XP_004028950.1](https://www.ncbi.nlm.nih.gov/protein/XP_004028950.1?report=genbank&log$=protalign&blast_rank=12&RID=0) 301 GTGTGSPRAVIGGRRRRRTALAGTRTAQGS GGG AGRGHGRRGRSRCSRKPLHVDFKELGWDDWIIAPLDYEAY 373

[XP_002812295.2](https://www.ncbi.nlm.nih.gov/protein/XP_002812295.2?report=genbank&log$=protalign&blast_rank=13&RID=0) 301 GTGTGSPRAVIGGRRRRRTALAGTRTAQGS SGG AGRGHGRRGRSRCSRKPLHVDFKELGWDDWIIAPLDYEAY 373

[XP_032028233.1](https://www.ncbi.nlm.nih.gov/protein/XP_032028233.1?report=genbank&log$=protalign&blast_rank=14&RID=0) 301 GTGTGSPRAVIGGRRRRRTALAGTRTAQGS GGG AGRGHGRRGRSRCSRKPLHVDFKELGWDDWIIAPLDYEAY 373

[NP_878248.2](https://www.ncbi.nlm.nih.gov/protein/NP_878248.2?report=genbank&log$=protalign&blast_rank=0&RID=0) 377 HCEGLCDFPLRSHLEPTNHAIIQTLLNSMAPDAAPASCCVPARLSPISILYIDAANNVVYKQYEDMVVEACGCR 450

[NP_001299805.1](https://www.ncbi.nlm.nih.gov/protein/NP_001299805.1?report=genbank&log$=protalign&blast_rank=1&RID=0) 388 HCEGVCDFPLRSHLEPTNHAIIQTLLNSMAPDAAPASCCVPARLSPISILYIDAANNVVYKQYEDMVVEACGCR 461

[XP_006239940.1](https://www.ncbi.nlm.nih.gov/protein/XP_006239940.1?report=genbank&log$=protalign&blast_rank=2&RID=0) 383 HCEGVCDFPLRSHLEPTNHAIIQTLLNSMAPDAAPASCCVPARLSPISILYIDAANNVVYKQYEDMVVEACGCR 456

[NP_001193030.1](https://www.ncbi.nlm.nih.gov/protein/NP_001193030.1?report=genbank&log$=protalign&blast_rank=3&RID=0) 377 HCEGVCDFPLRSHLEPTNHAIIQTLLNSMAPDAAPASCCVPARLSPISILYIDAANNVVYKQYEDMVVEACGCR 450

[XP_003354958.1](https://www.ncbi.nlm.nih.gov/protein/XP_003354958.1?report=genbank&log$=protalign&blast_rank=4&RID=0) 376 HCEGVCDFPLRSHLEPTNHAIIQTLLNSMAPDAAPASCCVPARLSPISILYIDAANNVVYKQYEDMVVEACGCR 449

[XP_038309370.1](https://www.ncbi.nlm.nih.gov/protein/XP_038309370.1?report=genbank&log$=protalign&blast_rank=5&RID=0) 385 HCEGVCDFPLRSHLEPTNHAIIQTLLNSMAPDAAPASCCVPARLSPISILYIDAANNVVYKQYEDMVVEACGCR 458

[XP_023475218.1](https://www.ncbi.nlm.nih.gov/protein/XP_023475218.1?report=genbank&log$=protalign&blast_rank=6&RID=0) 379 HCEGVCDFPLRSHLEPTNHAIIQTLLNSMAPDAAPASCCVPARLSPISILYIDAANNVVYKQYEDMVVEACGCR 452

[XP_027824132.2](https://www.ncbi.nlm.nih.gov/protein/XP_027824132.2?report=genbank&log$=protalign&blast_rank=7&RID=0) 384 HCEGVCDFPLRSHLEPTNHAIIQTLLNSMAPDAAPASCCVPARLSPISILYIDAANNVVYKQYEDMVVEACGCR 457

[XP_003308955.2](https://www.ncbi.nlm.nih.gov/protein/XP_003308955.2?report=genbank&log$=protalign&blast_rank=8&RID=0) 374 HCEGLCDFPLRSHLEPTNHAIIQTLLNSMAPDAAPASCCVPARLSPISILYIDAANNVVYKQYEDMVVEACGCR 447

[XP_001096970.2](https://www.ncbi.nlm.nih.gov/protein/XP_001096970.2?report=genbank&log$=protalign&blast_rank=9&RID=0) 377 HCEGVCDFPLRSHLEPTNHAIIQTLLNSMAPDAAPASCCVPARLSPISILYIDAANNVVYKQYEDMVVEACGCR 450

[XP_023107842.1](https://www.ncbi.nlm.nih.gov/protein/XP_023107842.1?report=genbank&log$=protalign&blast_rank=10&RID=0) 382 HCEGVCDFPLRSHLEPTNHAIIQTLLNSMAPDAAPASCCVPARLSPISILYIDAANNVVYKQYEDMVVEACGCR 455

[XP_017910266.1](https://www.ncbi.nlm.nih.gov/protein/XP_017910266.1?report=genbank&log$=protalign&blast_rank=11&RID=0) 378 HCEGVCDFPLRSHLEPTNHAIIQTLLNSMAPDAAPASCCVPARLSPISILYIDAANNVVYKQYEDMVVEACGCR 451

[XP_004028950.1](https://www.ncbi.nlm.nih.gov/protein/XP_004028950.1?report=genbank&log$=protalign&blast_rank=12&RID=0) 374 HCEGLCDFPLRSHLEPTNHAIIQTLLNSMAPDAAPASCCVPARLSPISILYIDAANNVVYKQYEDMVVEACGCR 447

[XP_002812295.2](https://www.ncbi.nlm.nih.gov/protein/XP_002812295.2?report=genbank&log$=protalign&blast_rank=13&RID=0) 374 HCEGLCDFPLRSHLEPTNHAIIQTLLNSMAPDAAPASCCVPARLSPISILYIDAANNVVYKQYEDMVVEACGCR 447

[XP_032028233.1](https://www.ncbi.nlm.nih.gov/protein/XP_032028233.1?report=genbank&log$=protalign&blast_rank=14&RID=0) 374 HCEGLCDFPLRSHLEPTNHAIIQTLLNSMAPDAAPASCCVPARLSPISILYIDAANNVVYKQYEDMVVEACGCR 447

BMP10

NP_055297.1 bone morphogenetic protein 10 preproprotein [Homo sapiens]

NP_033886.2 bone morphogenetic protein 10 preproprotein [Mus musculus]

NP_001026994.1 bone morphogenetic protein 10 precursor [Rattus norvegicus]

NP_001179047.1 bone morphogenetic protein 10 precursor [Bos taurus]

XP_003125118.1 bone morphogenetic protein 10 [Sus scrofa]

XP_538528.2 bone morphogenetic protein 10 [Canis lupus familiaris]

XP_001491666.1 bone morphogenetic protein 10 [Equus caballus]

XP_004005868.2 bone morphogenetic protein 10 [Ovis aries]

XP_525772.1 bone morphogenetic protein 10 [Pan troglodytes]

XP_001096299.1 bone morphogenetic protein 10 [Macaca mulatta]

XP_023107498.1 bone morphogenetic protein 10 [Felis catus]

XP_005686884.1 PREDICTED: bone morphogenetic protein 10 [Capra hircus]

XP_004029400.1 bone morphogenetic protein 10 [Gorilla gorilla gorilla]

XP_024098418.1 bone morphogenetic protein 10 [Pongo abelii]

XP_032033811.1 bone morphogenetic protein 10 [Hylobates moloch]

[NP_055297.1](https://www.ncbi.nlm.nih.gov/protein/NP_055297.1?report=genbank&log$=protalign&blast_rank=0&RID=0) 1 MGSLVLTLCALFCLAAYLVSGSPIMNLEQSPLEEDMSLFGDVFSEQDGVDFNTLLQSMKDEFLKTLNLSDIPTQDSAKVD 80

[NP_033886.2](https://www.ncbi.nlm.nih.gov/protein/NP_033886.2?report=genbank&log$=protalign&blast_rank=1&RID=0) 1 MGSLVLPLSAVFCLVAHSASGSPIMGLEQSPLEEDMPFFDDIFTEQDGIDFNTLLQSMKNEFLKTLNLSDIPVQDTGRVD 80

[NP_001026994.1](https://www.ncbi.nlm.nih.gov/protein/NP_001026994.1?report=genbank&log$=protalign&blast_rank=2&RID=0) 1 MGSLVLPLSAVFCLVARLASGSPIMGLEQSPLEEDMPFFDDIFTEQDGIDFNTLLQSMKDEFLKTLNLSDIPPQDTGRVD 80

[NP_001179047.1](https://www.ncbi.nlm.nih.gov/protein/NP_001179047.1?report=genbank&log$=protalign&blast_rank=3&RID=0) 1 MGSVVLQLCTLSCLLVHSVSGNPIMSLEQSPLEEDMPLFDDVFSEQDGVDFNTLLQSMKNEFLKTLNLSDIPMQDSAKVD 80

[XP_003125118.1](https://www.ncbi.nlm.nih.gov/protein/XP_003125118.1?report=genbank&log$=protalign&blast_rank=4&RID=0) 1 MGFLALQLCALSCLVAPWVSGSPIMSLEQSPLEEDMPLFDDVFSEQDGVDFNSLLQSMKNEFLKTLNLSDIPMQDSAKVD 80

[XP_538528.2](https://www.ncbi.nlm.nih.gov/protein/XP_538528.2?report=genbank&log$=protalign&blast_rank=5&RID=0) 1 MGSLALQLCAVFGLVAHSVSGSPIMSLEQSPLEEDMPLFDDVFSEQDSVDFNTLLQTMKNEFLKTLNLSDIPPQDSAKVD 80

[XP_001491666.1](https://www.ncbi.nlm.nih.gov/protein/XP_001491666.1?report=genbank&log$=protalign&blast_rank=6&RID=0) 1 MGSLALELCALFCLVAHLVSGSPIMSLERSPLEEDMPLFDDVFSEQDGVDFNTLLQSMKNEFLKTLNLSDIPMQDSAKVD 80

[XP_004005868.2](https://www.ncbi.nlm.nih.gov/protein/XP_004005868.2?report=genbank&log$=protalign&blast_rank=7&RID=0) 1 MGSVVLQLCTLSCLLLHAVSGNPIMSLEQSPLEEDMPLFDDVFSEQDGVDFNTLLQSMKNEFLKTLNLSDIPMQDSAKVD 80

[XP_525772.1](https://www.ncbi.nlm.nih.gov/protein/XP_525772.1?report=genbank&log$=protalign&blast_rank=8&RID=0) 1 MGSLVLTLCALFCLAAYLVSGSPIMNLEQSPLEEDMSLFGDVFSEQDGVDFNTLLQSMKDEFLKTLNLSDIPTQDSAKVD 80

[XP_001096299.1](https://www.ncbi.nlm.nih.gov/protein/XP_001096299.1?report=genbank&log$=protalign&blast_rank=9&RID=0) 1 MGSLGLTLCALFCLAAHSVSGSPIMSLEQSPLEEDMPLFDDVFSEQDGVDFNTLLQSMKDEFLKTLNLSDIPTQDSAKVD 80

[XP_023107498.1](https://www.ncbi.nlm.nih.gov/protein/XP_023107498.1?report=genbank&log$=protalign&blast_rank=10&RID=0) 1 MGSLALQLCALVCLVVHSVSGSPIMSLEQSPLEEDMPLFDDVFSEQDGVDFNTLLQSMKNEFLKTLNLSDIPSQDSAKVD 80

[XP_005686884.1](https://www.ncbi.nlm.nih.gov/protein/XP_005686884.1?report=genbank&log$=protalign&blast_rank=11&RID=0) 1 MGSVVLQLCTLSCLLLHAVSGNPIMSLEQSPLEEDMPLFDDVFSEQDGVDFNTLLQSMKNEFLKTLNLSDIPMQDSAKVD 80

[XP_004029400.1](https://www.ncbi.nlm.nih.gov/protein/XP_004029400.1?report=genbank&log$=protalign&blast_rank=12&RID=0) 1 MGSLVLTLCALFCLAAYLVSGSPIMNLEQSPLEEDMSLFGDVFSEQDGVDFNTLLQSMKDEFLKTLNLSDIPTQDSAKVD 80

[XP_024098418.1](https://www.ncbi.nlm.nih.gov/protein/XP_024098418.1?report=genbank&log$=protalign&blast_rank=13&RID=0) 1 MGSLVLTLCALFCLAAYLVSGSPIMSLEQSPLEEDMPLFDDVFSEQDGVDFNTLLQSMKDEFLKTLNLSDIPTQDSAKVD 80

[XP_032033811.1](https://www.ncbi.nlm.nih.gov/protein/XP_032033811.1?report=genbank&log$=protalign&blast_rank=14&RID=0) 1 MGSLVLTLCAFFCLAAYLVSGSPIMSLEQSPLEEDMPLFDDVFSEQDGVDFNTLLQSMKDEFLKTLNLSDIPTQESAKVD 80

[NP_055297.1](https://www.ncbi.nlm.nih.gov/protein/NP_055297.1?report=genbank&log$=protalign&blast_rank=0&RID=0) 81 PPEYMLELYNKFATDRTSMPSANIIRSFKNEDLFSQPVSFNGLRKYPLLFNVSIPHHEEVIMAELRLYTLVQRDRMIYDG 160

[NP_033886.2](https://www.ncbi.nlm.nih.gov/protein/NP_033886.2?report=genbank&log$=protalign&blast_rank=1&RID=0) 81 PPEYMLELYNKFATDRTSMPSANIIRSFKNEDLFSQPVTFNGLRKYPLLFNVSIPHHEEVVMAELRLYTLVQRDRMMYDG 160

[NP_001026994.1](https://www.ncbi.nlm.nih.gov/protein/NP_001026994.1?report=genbank&log$=protalign&blast_rank=2&RID=0) 81 PPEYMLELYNKFATDRTSMPSANIIRSFKNEDLFSQPVSFNGIRKYPLLFNVSIPHHEEVVMAELRLYTLVQRDRLMYDG 160

[NP_001179047.1](https://www.ncbi.nlm.nih.gov/protein/NP_001179047.1?report=genbank&log$=protalign&blast_rank=3&RID=0) 81 PPEYMLELYNKFATDRTSMPSANIIRSFKNEDLFSQPASFNGLRKYPLLFNVSIPHHEDIIMAELRLYTLVQRDRLIYEG 160

[XP_003125118.1](https://www.ncbi.nlm.nih.gov/protein/XP_003125118.1?report=genbank&log$=protalign&blast_rank=4&RID=0) 81 PPEYMLELYNKFATDRTSMPSANIIRSFKNEDLYSQPVSFNGLRKYPLLFNVSIPHHEEVTMAELRLYTLVQRDRLMYEG 160

[XP_538528.2](https://www.ncbi.nlm.nih.gov/protein/XP_538528.2?report=genbank&log$=protalign&blast_rank=5&RID=0) 81 PPEYMLELYNKFATDRTSMPSANIIRSFKNEDLFSQPASFNGLRKYPLLFNVSIPHHEEVIMAELRLYTLVQRDRLIYDG 160

[XP_001491666.1](https://www.ncbi.nlm.nih.gov/protein/XP_001491666.1?report=genbank&log$=protalign&blast_rank=6&RID=0) 81 PPEYMLELYNKFATDRTSMPSANIIRSFKNEDLFSQPASFNGLRKYPLFFNVSIPHHEEVIMAELRLYTLVQRDRIIYDG 160

[XP_004005868.2](https://www.ncbi.nlm.nih.gov/protein/XP_004005868.2?report=genbank&log$=protalign&blast_rank=7&RID=0) 81 PPEYMLELYNKFATDRTSMPSANIIRSFKNEDLFSQPASFNGLRKYPLLFNVSIPHHEDIIMAELRLYTLVQRDRLIYEG 160

[XP_525772.1](https://www.ncbi.nlm.nih.gov/protein/XP_525772.1?report=genbank&log$=protalign&blast_rank=8&RID=0) 81 PPEYMLELYNKFATDRTSMPSANIIRSFKNEDLFSQPVSFNGLRKYPLLFNVSIPHHEEVMMAELRLYTLVQRDRMIYDG 160

[XP_001096299.1](https://www.ncbi.nlm.nih.gov/protein/XP_001096299.1?report=genbank&log$=protalign&blast_rank=9&RID=0) 81 PPEYMLELYNKFATDRTSMPSANIIRSFKNEDLFSQPVSFNGLRKYPLLFNVSIPHHEEVIMAELRLYTLVQRDRMIYDG 160

[XP_023107498.1](https://www.ncbi.nlm.nih.gov/protein/XP_023107498.1?report=genbank&log$=protalign&blast_rank=10&RID=0) 81 PPEYMLELYNKFATDRTSMPSANIIRSFKNEDLFSQPASFNGLRKYPLLFNVSIPHHEEVIMAELRLYTLVQRDRMIYDG 160

[XP_005686884.1](https://www.ncbi.nlm.nih.gov/protein/XP_005686884.1?report=genbank&log$=protalign&blast_rank=11&RID=0) 81 PPEYMLELYNKFATDRTSMPSANIIRSFKNEDLFSQPASFNRLRKYPLLFNVSIPHHEDIIMAELRLYTLVQRDRLIYEG 160

[XP_004029400.1](https://www.ncbi.nlm.nih.gov/protein/XP_004029400.1?report=genbank&log$=protalign&blast_rank=12&RID=0) 81 PPEYMLELYNKFATDRTSMPSANIIRSFKNEDLFSQPVSFNGLRKYPLLFNVSIPHHEEVIMAELRLYTLVQRDRMIYDG 160

[XP_024098418.1](https://www.ncbi.nlm.nih.gov/protein/XP_024098418.1?report=genbank&log$=protalign&blast_rank=13&RID=0) 81 PPEYMLELYNKFATDRTSMPSANIIRSFKNEDLFSQPVSFNGLRKYHLLFNVSVPHHEEVIMAELRLYTLVQRDRMIYDG 160

[XP_032033811.1](https://www.ncbi.nlm.nih.gov/protein/XP_032033811.1?report=genbank&log$=protalign&blast_rank=14&RID=0) 81 PPEYMLELYNKFATDRTSMPSANIIRSFKNEDLFSQPVSFNGLRKYPLLFNVSIPHHEEVIMAELRLYTLVQRDRMIYDG 160

[NP_055297.1](https://www.ncbi.nlm.nih.gov/protein/NP_055297.1?report=genbank&log$=protalign&blast_rank=0&RID=0) 161 VDRKITIFEVLESKGDNEGERNMLVLVSGEIYGTNSEWETFDVTDAIRRWQKSGSSTHQLEVHIESKHDEAEDASSGRLE 240

[NP_033886.2](https://www.ncbi.nlm.nih.gov/protein/NP_033886.2?report=genbank&log$=protalign&blast_rank=1&RID=0) 161 VDRKITIFEVLESADGSEEERSMLVLVSTEIYGTNSEWETFDVTDATRRWQKSGPSTHQLEIHIESRQNQAEDTGRGQLE 240

[NP_001026994.1](https://www.ncbi.nlm.nih.gov/protein/NP_001026994.1?report=genbank&log$=protalign&blast_rank=2&RID=0) 161 VDRKIIIFEVLESADGSEDERSMLVLVSTEIYGTNSEWETFDITDATRRWQKSGPSTHQLEIHIESRQNQAEDTGRGQLE 240

[NP_001179047.1](https://www.ncbi.nlm.nih.gov/protein/NP_001179047.1?report=genbank&log$=protalign&blast_rank=3&RID=0) 161 VDRKITIFEVLESKEDHEGERNMLVLVSGEIYGTNSEWETFDVTDAIRHWQKSGSSTHQLEVHIESKHEMEDTLGRGQLE 240

[XP_003125118.1](https://www.ncbi.nlm.nih.gov/protein/XP_003125118.1?report=genbank&log$=protalign&blast_rank=4&RID=0) 161 VDRKITIFEVLESQGDHEGERSLLVLVSGEIYGTNSEWETFDVTDAIRRWQNSGSSTHQLEVHIESRQGM-EDAGRGQLE 239

[XP_538528.2](https://www.ncbi.nlm.nih.gov/protein/XP_538528.2?report=genbank&log$=protalign&blast_rank=5&RID=0) 161 VDRKITIFEVLESRGDTEGERSMLVLVSGEIYGTNSEWETFDVTDAIRRWQRSGSSTHQLEVHIESRHDGTEDAGRGHLE 240

[XP_001491666.1](https://www.ncbi.nlm.nih.gov/protein/XP_001491666.1?report=genbank&log$=protalign&blast_rank=6&RID=0) 161 VDRKITIYEVLESKGDNEGERTMLVLVSGEIYGTNSEWETFDVTNAIRHWQKSGLSTHQLEVHIESRQDEAEDAGRGQLE 240

[XP_004005868.2](https://www.ncbi.nlm.nih.gov/protein/XP_004005868.2?report=genbank&log$=protalign&blast_rank=7&RID=0) 161 VDRKITIFEVLESKEDHEGERSMLVLVSGEIYGTNSEWETFDVTDAIRHWQKSGSSTHQLEVHIESKHETEDTLGKGQLE 240

[XP_525772.1](https://www.ncbi.nlm.nih.gov/protein/XP_525772.1?report=genbank&log$=protalign&blast_rank=8&RID=0) 161 VDRKITIFEVLESKGDNEGERNMLVLVSGEIYGTNSEWETFDVTDAIRRWQKSGSSTHQLEVHIESKHDEAEDASSGRLE 240

[XP_001096299.1](https://www.ncbi.nlm.nih.gov/protein/XP_001096299.1?report=genbank&log$=protalign&blast_rank=9&RID=0) 161 VDRKITIFEVLESKGDNEGERNMLVLVSGEIYGTNSEWETFDVTDAIRHWQKSGSSTHQLEVHIESKHDEAEDASSGRLE 240

[XP_023107498.1](https://www.ncbi.nlm.nih.gov/protein/XP_023107498.1?report=genbank&log$=protalign&blast_rank=10&RID=0) 161 VDRKITIFEVLESRGDSEGERSMLVLVSGEIYGTNSEWETFDVTEAIRRWQKSGSSTHQLEVHIESRHDGVEDAGRGQLE 240

[XP_005686884.1](https://www.ncbi.nlm.nih.gov/protein/XP_005686884.1?report=genbank&log$=protalign&blast_rank=11&RID=0) 161 VDRKITIFEVLESKEDHEGERSMLVLVSGEIYGTNSEWETFDVTDAIRHWQKSGSSTHQLEVHIESKHETEDTLGRGQLE 240

[XP_004029400.1](https://www.ncbi.nlm.nih.gov/protein/XP_004029400.1?report=genbank&log$=protalign&blast_rank=12&RID=0) 161 VDRKITIFEVLESKGDNEGERNMLVLVSGEIYGTNSEWETFDVTDAIRHWQKSGSSTHQLEVHIESKHDEAEDASSGRLE 240

[XP_024098418.1](https://www.ncbi.nlm.nih.gov/protein/XP_024098418.1?report=genbank&log$=protalign&blast_rank=13&RID=0) 161 VDRKITIFEVLESKGDNEGERNMLVLVSGEIYGTNSEWETFDVTDAIRRWQKSGSSTHQLEVHIESKHDEAEDASSGRLE 240

[XP_032033811.1](https://www.ncbi.nlm.nih.gov/protein/XP_032033811.1?report=genbank&log$=protalign&blast_rank=14&RID=0) 161 VDRKITIFEVLESKGGNEGERNMLVLVSGEIYGTNSEWETFDVTDAIRRWQKSGSSTHQLEVHIESKHDEADDASSGRLE 240

[NP_055297.1](https://www.ncbi.nlm.nih.gov/protein/NP_055297.1?report=genbank&log$=protalign&blast_rank=0&RID=0) 241 IDTSAQNKHNPLLIVFSDDQSSDKERKEELNEMISHEQLPELDNLGLDSFSSGPGEEALLQMRSNIIYDSTARIRRNAKG 320

[NP_033886.2](https://www.ncbi.nlm.nih.gov/protein/NP_033886.2?report=genbank&log$=protalign&blast_rank=1&RID=0) 241 IDMSAQNKHDPLLVVFSDDQSNDKEQKEELNELITHEQ--DLD-LDSDAFFSGPDEEALLQMRSNMIDDSSARIRRNAKG 317

[NP_001026994.1](https://www.ncbi.nlm.nih.gov/protein/NP_001026994.1?report=genbank&log$=protalign&blast_rank=2&RID=0) 241 IDMSAQNKHDPLLVVFSDDQSGDKEQKEELNELISHEQ--DLD-LGTDGFFGGPDEEALLQMRSNMIDDSTARIRRNAKG 317

[NP_001179047.1](https://www.ncbi.nlm.nih.gov/protein/NP_001179047.1?report=genbank&log$=protalign&blast_rank=3&RID=0) 241 IDTSARNKHDPLLVVFSDDQSSEKERKEELDEMIAHEQFPEMDNLDLDGYSNGPGEEALLQMRSNIIYDSTARIRRNAKG 320

[XP_003125118.1](https://www.ncbi.nlm.nih.gov/protein/XP_003125118.1?report=genbank&log$=protalign&blast_rank=4&RID=0) 240 IDISARNKHEPLLVVFSDDQSSEKERKEELSEMIAHEQFPELDNLGLGGYSSGPGEEALLQMRSNIIYDSTARIRRNAKG 319

[XP_538528.2](https://www.ncbi.nlm.nih.gov/protein/XP_538528.2?report=genbank&log$=protalign&blast_rank=5&RID=0) 241 IDTSARNKHVPLLVVFSDDQSSEKERKEELNEMIAHEQLLELDNLGLEGYSSGPGEEALLQMRSNIIYDSTARIRRNAKG 320

[XP_001491666.1](https://www.ncbi.nlm.nih.gov/protein/XP_001491666.1?report=genbank&log$=protalign&blast_rank=6&RID=0) 241 IDTSAENKHDPLLVVFSDDQSSEKEGKEELTEMIAHEQLLELDNLGLEGFSSGPGEEALLQMRSNIIYDSTARIRRNAKG 320

[XP_004005868.2](https://www.ncbi.nlm.nih.gov/protein/XP_004005868.2?report=genbank&log$=protalign&blast_rank=7&RID=0) 241 IDTSAQNKHEPLLVVFSDDQSSEKERKEELDEMIAHEQFPEMDNLDLDGYSNGPGEEALLQMRSNIIYDSTARIRRNAKG 320

[XP_525772.1](https://www.ncbi.nlm.nih.gov/protein/XP_525772.1?report=genbank&log$=protalign&blast_rank=8&RID=0) 241 IDTSAQNKHNPLLIVFSDDQSSDKERKEELNEMISHEQLPELDNLGLDSFSSGPGEEALLQMRSNIIYDSTARIRRNAKG 320

[XP_001096299.1](https://www.ncbi.nlm.nih.gov/protein/XP_001096299.1?report=genbank&log$=protalign&blast_rank=9&RID=0) 241 IDTSAQNKHNPLLIVFSDDQSSDKERKEELNEMISHEQLPELDNLGLDGFSGGPGEEALLQMRSNIIYDSTARIRRNAKG 320

[XP_023107498.1](https://www.ncbi.nlm.nih.gov/protein/XP_023107498.1?report=genbank&log$=protalign&blast_rank=10&RID=0) 241 IDTSARNKHVPLLVVFSDDQSSEKERTEELNEMIAHEQLLEFDNLGMDSYSSGPGEEALLQMRSNIIYDSTARIRRNAKG 320

[XP_005686884.1](https://www.ncbi.nlm.nih.gov/protein/XP_005686884.1?report=genbank&log$=protalign&blast_rank=11&RID=0) 241 IDTSARNKHEPLLVVFSDDQSSEKERKEELDEMIAHEQFPEMDNLDLDGYSNGPGEEALLQMRSNIIYDSTARIRRNAKG 320

[XP_004029400.1](https://www.ncbi.nlm.nih.gov/protein/XP_004029400.1?report=genbank&log$=protalign&blast_rank=12&RID=0) 241 IDTSAQNKHNPLLIVFSDDQSSDKERKEELNEMISHEQLPELDSLGLDSFSSGPGEEALLQMRSNIIYDSTARIRRNAKG 320

[XP_024098418.1](https://www.ncbi.nlm.nih.gov/protein/XP_024098418.1?report=genbank&log$=protalign&blast_rank=13&RID=0) 241 IDTSAQNKHNPLLIVFSDDQSSDKERKEELNEMISHEQLPELDSLGLDSFSSGPGEEALLQMRSNIIYDSTARIRRNAKG 320

[XP_032033811.1](https://www.ncbi.nlm.nih.gov/protein/XP_032033811.1?report=genbank&log$=protalign&blast_rank=14&RID=0) 241 IDTSAQNKHNPLLIVFSDDQSSDKERKEELNEMISHEQLPELDNLGLDSFSSGPAEEALLQMRSNIIYDSTARIRRNAKG 320

[NP_055297.1](https://www.ncbi.nlm.nih.gov/protein/NP_055297.1?report=genbank&log$=protalign&blast_rank=0&RID=0) 321 NYCKRTPLYIDFKEIGWDSWIIAPPGYEAYECRGVCNYPLAEHLTPTKHAIIQALVHLKNSQKASKACCVPTKLEPISIL 400

[NP_033886.2](https://www.ncbi.nlm.nih.gov/protein/NP_033886.2?report=genbank&log$=protalign&blast_rank=1&RID=0) 318 NYCKKTPLYIDFKEIGWDSWIIAPPGYEAYECRGVCNYPLAEHLTPTKHAIIQALVHLKNSQKASKACCVPTKLDPISIL 397

[NP_001026994.1](https://www.ncbi.nlm.nih.gov/protein/NP_001026994.1?report=genbank&log$=protalign&blast_rank=2&RID=0) 318 NYCKKTPLYIDFKEIGWDSWIIAPPGYEAYECRGVCNYPLAEHLTPTKHAIIQALVHLKNSQKASKACCVPTKLDPISIL 397

[NP_001179047.1](https://www.ncbi.nlm.nih.gov/protein/NP_001179047.1?report=genbank&log$=protalign&blast_rank=3&RID=0) 321 NYCKRTPLYIDFKEIGWDSWIIAPPGYEAYECRGVCNYPLAEHLTPTKHAIIQALVHLKNSQKASKACCVPTKLEPISIL 400

[XP_003125118.1](https://www.ncbi.nlm.nih.gov/protein/XP_003125118.1?report=genbank&log$=protalign&blast_rank=4&RID=0) 320 NYCKRTPLYIDFKEIGWDSWIIAPPGYEAYECRGVCNYPLAEHLTPTKHAIIQALVHLKNSQKASKACCVPTKLEPISIL 399

[XP_538528.2](https://www.ncbi.nlm.nih.gov/protein/XP_538528.2?report=genbank&log$=protalign&blast_rank=5&RID=0) 321 NYCKRTPLYIDFKEIGWDSWIIAPPGYEAYECRGVCNYPLAEHLTPTKHAIIQALVHLKNSQKASKVCCVPTKLEPISIL 400

[XP_001491666.1](https://www.ncbi.nlm.nih.gov/protein/XP_001491666.1?report=genbank&log$=protalign&blast_rank=6&RID=0) 321 NYCKRTPLYIDFKEIGWDSWIIAPPGYEAYECRGVCNYPLAEHLTPTKHAIIQALVHLKNSQKASKACCVPTKLEPISIL 400

[XP_004005868.2](https://www.ncbi.nlm.nih.gov/protein/XP_004005868.2?report=genbank&log$=protalign&blast_rank=7&RID=0) 321 NYCKRTPLYIDFKEIGWDSWIIAPPGYEAYECRGVCNYPLAEHLTPTKHAIIQALVHLKNSQKASKACCVPTKLEPISIL 400

[XP_525772.1](https://www.ncbi.nlm.nih.gov/protein/XP_525772.1?report=genbank&log$=protalign&blast_rank=8&RID=0) 321 NYCKRTPLYIDFKEIGWDSWIIAPPGYEAYECRGVCNYPLAEHLTPTKHAIIQALVHLKNSQKASKACCVPTKLEPISIL 400

[XP_001096299.1](https://www.ncbi.nlm.nih.gov/protein/XP_001096299.1?report=genbank&log$=protalign&blast_rank=9&RID=0) 321 NYCKRTPLYIDFKEIGWDSWIIAPPGYEAYECRGVCNYPLAEHLTPTKHAIIQALVHLKNSQKASKACCVPTKLEPISIL 400

[XP_023107498.1](https://www.ncbi.nlm.nih.gov/protein/XP_023107498.1?report=genbank&log$=protalign&blast_rank=10&RID=0) 321 NYCKRTPLYIDFKEIGWDSWIIAPPGYEAYECRGVCNYPLAEHLTPTKHAIIQALVHLKNSQKASKACCVPTKLEPISIL 400

[XP_005686884.1](https://www.ncbi.nlm.nih.gov/protein/XP_005686884.1?report=genbank&log$=protalign&blast_rank=11&RID=0) 321 NYCKRTPLYIDFKEIGWDSWIIAPPGYEAYECRGVCNYPLAEHLTPTKHAIIQALVHLKNSQKASKACCVPTKLEPISIL 400

[XP_004029400.1](https://www.ncbi.nlm.nih.gov/protein/XP_004029400.1?report=genbank&log$=protalign&blast_rank=12&RID=0) 321 NYCKRTPLYIDFKEIGWDSWIIAPPGYEAYECRGVCNYPLAEHLTPTKHAIIQALVHLKNSQKASKACCVPTKLEPISIL 400

[XP_024098418.1](https://www.ncbi.nlm.nih.gov/protein/XP_024098418.1?report=genbank&log$=protalign&blast_rank=13&RID=0) 321 NYCKRTPLYIDFKEIGWDSWIIAPPGYEAYECRGVCNYPLAEHLTPTKHAIIQALVHLKNSQKASKACCVPTKLEPISIL 400

[XP_032033811.1](https://www.ncbi.nlm.nih.gov/protein/XP_032033811.1?report=genbank&log$=protalign&blast_rank=14&RID=0) 321 NYCKRTPLYIDFKEIGWDSWIIAPPGYEAYECRGVCNYPLAEHLTPTKHAIIQALVHLKNSQKASKACCVPTKLEPISIL 400

[NP_055297.1](https://www.ncbi.nlm.nih.gov/protein/NP_055297.1?report=genbank&log$=protalign&blast_rank=0&RID=0) 401 YLDKGVVTYKFKYEGMAVSECGCR 424

[NP_033886.2](https://www.ncbi.nlm.nih.gov/protein/NP_033886.2?report=genbank&log$=protalign&blast_rank=1&RID=0) 398 YLDKGVVTYKFKYEGMAVSECGCR 421

[NP_001026994.1](https://www.ncbi.nlm.nih.gov/protein/NP_001026994.1?report=genbank&log$=protalign&blast_rank=2&RID=0) 398 YLDKGVVTYKFKYEGMAVSECGCR 421

[NP_001179047.1](https://www.ncbi.nlm.nih.gov/protein/NP_001179047.1?report=genbank&log$=protalign&blast_rank=3&RID=0) 401 YLDKGVVTYKFKYEGMAVSECGCR 424

[XP_003125118.1](https://www.ncbi.nlm.nih.gov/protein/XP_003125118.1?report=genbank&log$=protalign&blast_rank=4&RID=0) 400 YLDKGVVTYKFKYEGMAVSECGCR 423

[XP_538528.2](https://www.ncbi.nlm.nih.gov/protein/XP_538528.2?report=genbank&log$=protalign&blast_rank=5&RID=0) 401 YLDKGVVTYKFKYEGMAVSECGCR 424

[XP_001491666.1](https://www.ncbi.nlm.nih.gov/protein/XP_001491666.1?report=genbank&log$=protalign&blast_rank=6&RID=0) 401 YLDKGVVTYKFKYEGMAVSECGCR 424

[XP_004005868.2](https://www.ncbi.nlm.nih.gov/protein/XP_004005868.2?report=genbank&log$=protalign&blast_rank=7&RID=0) 401 YLDKGVVTYKFKYEGMAVSECGCR 424

[XP_525772.1](https://www.ncbi.nlm.nih.gov/protein/XP_525772.1?report=genbank&log$=protalign&blast_rank=8&RID=0) 401 YLDKGVVTYKFKYEGMAVSECGCR 424

[XP_001096299.1](https://www.ncbi.nlm.nih.gov/protein/XP_001096299.1?report=genbank&log$=protalign&blast_rank=9&RID=0) 401 YLDKGVVTYKFKYEGMAVSECGCR 424

[XP_023107498.1](https://www.ncbi.nlm.nih.gov/protein/XP_023107498.1?report=genbank&log$=protalign&blast_rank=10&RID=0) 401 YLDKGVVTYKFKYEGMAVSECGCR 424

[XP_005686884.1](https://www.ncbi.nlm.nih.gov/protein/XP_005686884.1?report=genbank&log$=protalign&blast_rank=11&RID=0) 401 YLDKGVVTYKFKYEGMAVSECGCR 424

[XP_004029400.1](https://www.ncbi.nlm.nih.gov/protein/XP_004029400.1?report=genbank&log$=protalign&blast_rank=12&RID=0) 401 YLDKGVVTYKFKYEGMAVSECGCR 424

[XP_024098418.1](https://www.ncbi.nlm.nih.gov/protein/XP_024098418.1?report=genbank&log$=protalign&blast_rank=13&RID=0) 401 YLDKGVVTYKFKYEGMAVSECGCR 424

[XP_032033811.1](https://www.ncbi.nlm.nih.gov/protein/XP_032033811.1?report=genbank&log$=protalign&blast_rank=14&RID=0) 401 YLDKGVVTYKFKYEGMAVSECGCR 424

INHBA

NP_002183.1 inhibin beta A chain preproprotein [Homo sapiens]

NP_032406.1 inhibin beta A chain preproprotein [Mus musculus]

NP_058824.1 inhibin beta A chain preproprotein [Rattus norvegicus]

NP_776788.1 inhibin beta A chain precursor [Bos taurus]

NP_999193.1 inhibin beta A chain precursor [Sus scrofa]

XP_540364.2 inhibin beta A chain [Canis lupus familiaris]

NP_001075378.1 inhibin beta A chain precursor [Equus caballus]

NP_001009458.1 inhibin beta A chain precursor [Ovis aries]

XP_519063.2 inhibin beta A chain [Pan troglodytes]

XP_028701686.1 inhibin beta A chain [Macaca mulatta]

NP_001009856.1 inhibin beta A chain precursor [Felis catus]

NP_001272510.1 inhibin beta A chain precursor [Capra hircus]

XP_004045385.1 inhibin beta A chain [Gorilla gorilla gorilla]

XP_002818070.1 inhibin beta A chain [Pongo abelii]

XP_032614156.1 inhibin beta A chain [Hylobates moloch]

[NP_002183.1](https://www.ncbi.nlm.nih.gov/protein/NP_002183.1?report=genbank&log$=protalign&blast_rank=0&RID=0) 1 MPLLWLRGFLLASCWIIVRSSPTPGSEGHSAAPDCPSCALAALPKDVPNSQPEMVEAVKKHILNMLHLKKRPDVTQPVPK 80

[NP_032406.1](https://www.ncbi.nlm.nih.gov/protein/NP_032406.1?report=genbank&log$=protalign&blast_rank=1&RID=0) 1 MPLLWLRGFLLASCWIIVRSSPTPGSEGHGSAPDCPSCALATLPKDGPNSQPEMVEAVKKHILNMLHLKKRPDVTQPVPK 80

[NP_058824.1](https://www.ncbi.nlm.nih.gov/protein/NP_058824.1?report=genbank&log$=protalign&blast_rank=2&RID=0) 1 MPLLWLRGFLLASCWIIVRSSPTPGSEGHGAAPDCPSCALATLPKDGPNSQPEMVEAVKKHILNMLHLKKRPDVTQPVPK 80

[NP_776788.1](https://www.ncbi.nlm.nih.gov/protein/NP_776788.1?report=genbank&log$=protalign&blast_rank=3&RID=0) 1 MPLLWLRGFLLASCWIIVRSSPTPGSEGHSAAPDCPSCALATLPKDVPNSQPEMVEAVKKHILNMLHLKKRPDVTQPVPK 80

[NP_999193.1](https://www.ncbi.nlm.nih.gov/protein/NP_999193.1?report=genbank&log$=protalign&blast_rank=4&RID=0) 1 MPLLWLRGFLLASCWIIVRSSPTPGSGGHSAAPDCPSCALATLPKDVPNSQPEMVEAVKKHILNMLHLKKRPDVTQPVPK 80

[XP_540364.2](https://www.ncbi.nlm.nih.gov/protein/XP_540364.2?report=genbank&log$=protalign&blast_rank=5&RID=0) 1 MPLLWLRGFLVASCWIIVRSSPTPGPEGPGAAPACPACALTALPRDAPNSQPEMVEAVKKHILNMLHLKKRPEVTQPVPK 80

[NP_001075378.1](https://www.ncbi.nlm.nih.gov/protein/NP_001075378.1?report=genbank&log$=protalign&blast_rank=6&RID=0) 1 MPLLWLRGFLLASCWIIVKSSPTPGSEGHSAAPNCPSCALATLPKDVPNAQPEMVEAVKKHILNMLHLKKRPDVTQPVPK 80

[NP_001009458.1](https://www.ncbi.nlm.nih.gov/protein/NP_001009458.1?report=genbank&log$=protalign&blast_rank=7&RID=0) 1 MPLLWLRGFLLASCWIIVRSSPTPGSEGHSAAPDCPSCALATLPKDVPNSQPEMVEAVKKHILNMLHLKKRPDVTQPVPK 80

[XP_519063.2](https://www.ncbi.nlm.nih.gov/protein/XP_519063.2?report=genbank&log$=protalign&blast_rank=8&RID=0) 1 MPLLWLRGFLLASCWIIVRSSPTPGSEGHSAAPDCPSCALAALPKDVPNSQPEMVEAVKKHILNMLHLKKRPDVTQPVPK 80

[XP_028701686.1](https://www.ncbi.nlm.nih.gov/protein/XP_028701686.1?report=genbank&log$=protalign&blast_rank=9&RID=0) 1 MPLLWLRGFLLASCWIIVRSSPTPGSEGHSAAPDCPSCALAALPKDVPNSQPEMVEAVKKHILNMLHLKKRPDVTQPVPK 80

[NP_001009856.1](https://www.ncbi.nlm.nih.gov/protein/NP_001009856.1?report=genbank&log$=protalign&blast_rank=10&RID=0) 1 MPLLWLRGFLLASCWIIVRSSPTPGSEGPGAAPDCPSCALATLPKDVPNSQPEMVEAVKKHILNMLHLKKRPEVTQPVPK 80

[NP_001272510.1](https://www.ncbi.nlm.nih.gov/protein/NP_001272510.1?report=genbank&log$=protalign&blast_rank=11&RID=0) 1 MPLLWLRGFLLASCWIIVRSSPTPGSEGHSAAPDCPSCALATLPKDVPNSQPEMVEAVKKHILNMLHLKKRPDVTQPVPK 80

[XP_004045385.1](https://www.ncbi.nlm.nih.gov/protein/XP_004045385.1?report=genbank&log$=protalign&blast_rank=12&RID=0) 1 MPLLWLRGFLLASCWIIVRSSPTPGSEGHSAAPDCPSCALAALPKDVPNSQPEMVEAVKKHILNMLHLKKRPDVTQPVPK 80

[XP_002818070.1](https://www.ncbi.nlm.nih.gov/protein/XP_002818070.1?report=genbank&log$=protalign&blast_rank=13&RID=0) 1 MPLLWLRGFLLASCWIIVRSSPTPGSEGHSAAPDCPSCALAALPKDVPNSQPEMVEAVKKHILNMLHLKKRPDVTQPVPK 80

[XP_032614156.1](https://www.ncbi.nlm.nih.gov/protein/XP_032614156.1?report=genbank&log$=protalign&blast_rank=14&RID=0) 1 MPLLWLRGFLLASCWIIVRSSPTPGSEGHSAAPDCPSCALAALPKDVPNSQPEMVEAVKKHILNMLHLKKRPDVTQPVPK 80

[NP_002183.1](https://www.ncbi.nlm.nih.gov/protein/NP_002183.1?report=genbank&log$=protalign&blast_rank=0&RID=0) 81 AALLNAIRKLHVGKVGENGYVEIEDDIGRRAEMNELMEQTSEIITFAESGTARKTLHFEISKEGSDLSVVERAEVWLFLK 160

[NP_032406.1](https://www.ncbi.nlm.nih.gov/protein/NP_032406.1?report=genbank&log$=protalign&blast_rank=1&RID=0) 81 AALLNAIRKLHVGKVGENGYVEIEDDIGRRAEMNELMEQTSEIITFAESGTARKTLHFEISKEGSDLSVVERAEVWLFLK 160

[NP_058824.1](https://www.ncbi.nlm.nih.gov/protein/NP_058824.1?report=genbank&log$=protalign&blast_rank=2&RID=0) 81 AALLNAIRKLHVGKVGENGYVEIEDDIGRRAEMNELMEQTSEIITFAESGTARKTLHFEISKEGSDLSVVERAEVWLFLK 160

[NP_776788.1](https://www.ncbi.nlm.nih.gov/protein/NP_776788.1?report=genbank&log$=protalign&blast_rank=3&RID=0) 81 AALLNAIRKLHVGKVGENGYVEIEDDIGRRAEMNELMEQTSEIITFAESGTARKTLHFEISKEGSDLSVVERAEIWLFLK 160

[NP_999193.1](https://www.ncbi.nlm.nih.gov/protein/NP_999193.1?report=genbank&log$=protalign&blast_rank=4&RID=0) 81 AALLNAIRKLHVGKVGENGYVELEDDIGRRAEMNELMEQTSEIITFAEAGTARKTLRFEISKEGSDLSVVERAEIWLFLK 160

[XP_540364.2](https://www.ncbi.nlm.nih.gov/protein/XP_540364.2?report=genbank&log$=protalign&blast_rank=5&RID=0) 81 AALLNAIRKLHVGKVGENGFVEIEDDIGRRAEMNELMEQTSEIITFAESGTARKTLHFEISKEGSDLSVVERAEVWLFLK 160

[NP_001075378.1](https://www.ncbi.nlm.nih.gov/protein/NP_001075378.1?report=genbank&log$=protalign&blast_rank=6&RID=0) 81 AALLNAIRKLHVGKVGENGYVEIEDDIGRRAEMNELMEQTSEIITFAESGTARKTLHFEISKEGSDLSVVERAEVWLFLK 160

[NP_001009458.1](https://www.ncbi.nlm.nih.gov/protein/NP_001009458.1?report=genbank&log$=protalign&blast_rank=7&RID=0) 81 AALLNAIRKLHVGKVGENGYVEIEDDIGRRAEMNELMEQTSEIITFAESGTARKTLHFEISQEGSDLSVVERAEIWLFLK 160

[XP_519063.2](https://www.ncbi.nlm.nih.gov/protein/XP_519063.2?report=genbank&log$=protalign&blast_rank=8&RID=0) 81 AALLNAIRKLHVGKVGENGYVEIEDDIGRRAEMNELMEQTSEIITFAESGTARKTLHFEISKEGSDLSVVERAEVWLFLK 160

[XP_028701686.1](https://www.ncbi.nlm.nih.gov/protein/XP_028701686.1?report=genbank&log$=protalign&blast_rank=9&RID=0) 81 AALLNAIRKLHVGKVGENGYVEIEDDIGRRAEMNELMEQTSEIITFAESGTTRKTLHFEISKEGSDLSVVERAEVWLFLK 160

[NP_001009856.1](https://www.ncbi.nlm.nih.gov/protein/NP_001009856.1?report=genbank&log$=protalign&blast_rank=10&RID=0) 81 AALLNAIRKLHVGKVGENGYVEIEDDIGRRAEMNELMEQTSEIITFAESGTARKTLHFEISKEGSDLSVVERAEVWLFLK 160

[NP_001272510.1](https://www.ncbi.nlm.nih.gov/protein/NP_001272510.1?report=genbank&log$=protalign&blast_rank=11&RID=0) 81 AALLNAIRKLHVGKVGENGYVEIEDDIGRRAEMNELMEQTSEIITFAESGTARKTLHFEISKEGSDLSVVERAEIWLFLK 160

[XP_004045385.1](https://www.ncbi.nlm.nih.gov/protein/XP_004045385.1?report=genbank&log$=protalign&blast_rank=12&RID=0) 81 AALLNAIRKLHVGKVGENGYVEIEDDIGRRAEMNELMEQTSEIITFAESGTARKTLHFEISKEGSDLSVVERAEVWLFLK 160

[XP_002818070.1](https://www.ncbi.nlm.nih.gov/protein/XP_002818070.1?report=genbank&log$=protalign&blast_rank=13&RID=0) 81 AALLNAIRKLHVGKVGENGYVEIEDDIGRRAEMNELMEQTSEIITFAESGTARKTLHFEISKEGSDLSVVERAEVWLFLK 160

[XP_032614156.1](https://www.ncbi.nlm.nih.gov/protein/XP_032614156.1?report=genbank&log$=protalign&blast_rank=14&RID=0) 81 AALLNAIRKLHVGKVGENGYVEIEDDIGRRAEMNELMEQTSEIITFAESGTARKTLHFEISKEGSDLSVVERAEVWLFLK 160

[NP_002183.1](https://www.ncbi.nlm.nih.gov/protein/NP_002183.1?report=genbank&log$=protalign&blast_rank=0&RID=0) 161 VPKANRTRTKVTIRLFQQQKHPQGSLDTGEEAEEVGLKGERSELLLSEKVVDARKSTWHVFPVSSSIQRLLDQGKSSLDV 240

[NP_032406.1](https://www.ncbi.nlm.nih.gov/protein/NP_032406.1?report=genbank&log$=protalign&blast_rank=1&RID=0) 161 VPKANRTRTKVTIRLFQQQKHPQGSLDTGDEAEEMGLKGERSELLLSEKVVDARKSTWHIFPVSSSIQRLLDQGKSSLDV 240

[NP_058824.1](https://www.ncbi.nlm.nih.gov/protein/NP_058824.1?report=genbank&log$=protalign&blast_rank=2&RID=0) 161 VPKANRTRTKVTIRLFQQQKHPQGSLDMGDEAEEMGLKGERSELLLSEKVVDARKSTWHIFPVSSSIQRLLDQGKSSLDV 240

[NP_776788.1](https://www.ncbi.nlm.nih.gov/protein/NP_776788.1?report=genbank&log$=protalign&blast_rank=3&RID=0) 161 VPKANRTRSKVTIRLFQQQKHLQGSLDAGEEAEEVGLKGEKSEMLISEKVVDARKSTWHIFPVSSCIQRLLDQGKSSLDI 240

[NP_999193.1](https://www.ncbi.nlm.nih.gov/protein/NP_999193.1?report=genbank&log$=protalign&blast_rank=4&RID=0) 161 VPKANRTRTKVSIRLFQQQRRPQGSADAGEEAEDVGFPEEKSEVLISEKVVDARKSTWHIFPVSSSIQRLLDQGKSALDI 240

[XP_540364.2](https://www.ncbi.nlm.nih.gov/protein/XP_540364.2?report=genbank&log$=protalign&blast_rank=5&RID=0) 161 VPKANRTRTKVTIRLLQ--KHPQGSLDAGEEAEDMGFPEERNEVLISEKVVDARKSTWHIFPVSSSIQRLLDQGRSSLDV 238

[NP_001075378.1](https://www.ncbi.nlm.nih.gov/protein/NP_001075378.1?report=genbank&log$=protalign&blast_rank=6&RID=0) 161 VPKANRTRSKVTIRLLQQQKHPQGSSDTREEAEEADLMEERSEQLISEKVVDARKSTWHIFPVSSSIQRLLDQGKSSLDI 240

[NP_001009458.1](https://www.ncbi.nlm.nih.gov/protein/NP_001009458.1?report=genbank&log$=protalign&blast_rank=7&RID=0) 161 VPKANRTRSKVTIRLFQQQKHLQGSLDAGEEAEEVGLKGEKSEMLISEKVVDARKSTWHIFPVSSCIQRLLDQGKSSLDI 240

[XP_519063.2](https://www.ncbi.nlm.nih.gov/protein/XP_519063.2?report=genbank&log$=protalign&blast_rank=8&RID=0) 161 VPKANRTRTKVTIRLFQQQKHPQGSLDTGEEAEEVGLKGERSELLLSEKVVDARKSTWHVFPVSSSIQRLLDQGKSSLDV 240

[XP_028701686.1](https://www.ncbi.nlm.nih.gov/protein/XP_028701686.1?report=genbank&log$=protalign&blast_rank=9&RID=0) 161 VPKANRTRTKVTIRLFQQQKHPQGSLDTGEEAEEVGLKGERSELLLSEKVVDARKSTWHVFPVSSSIQRLLDQGKSSLDV 240

[NP_001009856.1](https://www.ncbi.nlm.nih.gov/protein/NP_001009856.1?report=genbank&log$=protalign&blast_rank=10&RID=0) 161 VPKANRTRTKVTIQLLQKQ--PQGGVDAGEEAEEMGLMEERNEVLISEKVVDARKSTWHIFPVSSSIQRLLDQGKSSLDV 238

[NP_001272510.1](https://www.ncbi.nlm.nih.gov/protein/NP_001272510.1?report=genbank&log$=protalign&blast_rank=11&RID=0) 161 VPKANRTRSKVTIRLFQQQKHLQGSLDAGEEAEEVGLKGEKSEMLISEKVVDARKSTWHIFPVSSCIQRLLDQGKSSLDI 240

[XP_004045385.1](https://www.ncbi.nlm.nih.gov/protein/XP_004045385.1?report=genbank&log$=protalign&blast_rank=12&RID=0) 161 VPKANRTRTKVTIRLFQQQKHPQGSLDTGEEAEEVGLKGERSELLLSEKVVDARKSTWHVFPVSSSIQRLLDQGKSSLDV 240

[XP_002818070.1](https://www.ncbi.nlm.nih.gov/protein/XP_002818070.1?report=genbank&log$=protalign&blast_rank=13&RID=0) 161 VPKANRTRTKVTIRLFQQQKHPQGSLDTGEEAEEVGLKGERSELLLSEKVVDARKSTWHVFPVSSSIQRLLDQGKSSLDV 240

[XP_032614156.1](https://www.ncbi.nlm.nih.gov/protein/XP_032614156.1?report=genbank&log$=protalign&blast_rank=14&RID=0) 161 VPKANRTRTKVTIRLFQQQKHPQGSLDTGEEAEEVGLKGERSELLLSEKVVDARKSTWHVFPVSSSIQRLLDQGKSSLDV 240

[NP_002183.1](https://www.ncbi.nlm.nih.gov/protein/NP_002183.1?report=genbank&log$=protalign&blast_rank=0&RID=0) 241 RIACEQCQESGASLVLLGKKKKKEEEGEGKKKGGGEGGAGADEEKEQSHRPFLMLQARQSEDHPHRRRRRGLECDGKVNI 320

[NP_032406.1](https://www.ncbi.nlm.nih.gov/protein/NP_032406.1?report=genbank&log$=protalign&blast_rank=1&RID=0) 241 RIACEQCQESGASLVLLGKKKKKEVDGDGKKKDGSDGGL--EEEKEQSHRPFLMLQARQSEDHPHRRRRRGLECDGKVNI 318

[NP_058824.1](https://www.ncbi.nlm.nih.gov/protein/NP_058824.1?report=genbank&log$=protalign&blast_rank=2&RID=0) 241 RIACEQCQESGASLVLLGKKKKKEVDGDGKKKDGSDGGL--EEEKEQSHRPFLMLQARQSEDHPHRRRRRGLECDGKVNI 318

[NP_776788.1](https://www.ncbi.nlm.nih.gov/protein/NP_776788.1?report=genbank&log$=protalign&blast_rank=3&RID=0) 241 RIACEQCQETGASLVLLGKKKKKEEEGEGKKRD-GEGGAGGDEEKEQSHRPFLMLQARQSEDHPHRRRRRGLECDGKVNI 319

[NP_999193.1](https://www.ncbi.nlm.nih.gov/protein/NP_999193.1?report=genbank&log$=protalign&blast_rank=4&RID=0) 241 RTACEQCHETGASLVLLGKKKKKEEEAEGRKRDG--EGAGVDEEKEQSHRPFLMLQARQSEEHPHRRRRRGLECDGKVNI 318

[XP_540364.2](https://www.ncbi.nlm.nih.gov/protein/XP_540364.2?report=genbank&log$=protalign&blast_rank=5&RID=0) 239 RIACEQCHETGASLVLLGKKKKKEEEGEGKKKDGGDAGAGGDEDKEQSHRPFLMLQARQSEDHPHRRRRRGLECDGKVNI 318

[NP_001075378.1](https://www.ncbi.nlm.nih.gov/protein/NP_001075378.1?report=genbank&log$=protalign&blast_rank=6&RID=0) 241 RIACDQCHETGASLVLLGKKKKKEEEGEGKKKDGGEAGAGVDEEKEQSHRPFLMLQARQSEDHPHRRRRRGLECDGKVNI 320

[NP_001009458.1](https://www.ncbi.nlm.nih.gov/protein/NP_001009458.1?report=genbank&log$=protalign&blast_rank=7&RID=0) 241 RIACEQCQETGASLVLLGKKKRKEEEGEGKKRD-GEGGAGGDEEKEQSHRPFLMLQARQSEDHPHRRRRRGLECDGKVNI 319

[XP_519063.2](https://www.ncbi.nlm.nih.gov/protein/XP_519063.2?report=genbank&log$=protalign&blast_rank=8&RID=0) 241 RIACEQCQESGASLVLLGKKKKKEEEGEGKKKGGGEGGAGADEEKEQSHRPFLMLQARQSEDHPHRRRRRGLECDGKVNI 320

[XP_028701686.1](https://www.ncbi.nlm.nih.gov/protein/XP_028701686.1?report=genbank&log$=protalign&blast_rank=9&RID=0) 241 RIACEQCQESGASLVLLGKKKKKEEEGDGKKKGGGEGGAGADEEKEQSHRPFLMLQARQSEDHPHRRRRRGLECDGKVNI 320

[NP_001009856.1](https://www.ncbi.nlm.nih.gov/protein/NP_001009856.1?report=genbank&log$=protalign&blast_rank=10&RID=0) 239 RIACEQCHETGASLVLLGKKKKKEEEGEGKKKDGGDGGAGADEDKEQSHRPFLMLQARQSEDHPHRRRRRGLECDGKVNI 318

[NP_001272510.1](https://www.ncbi.nlm.nih.gov/protein/NP_001272510.1?report=genbank&log$=protalign&blast_rank=11&RID=0) 241 RIACEQCQETGASLVLLGKKKRKEEEGEGKKRD-GEGGAGGDEEKEQSHRPFLMLQARQSEDHPHRRRRRGLECDGKVNI 319

[XP_004045385.1](https://www.ncbi.nlm.nih.gov/protein/XP_004045385.1?report=genbank&log$=protalign&blast_rank=12&RID=0) 241 RIACEQCQESGASLVLLGKKKKKEEEGEGKKKGGGEGGAGADEEKEQSHRPFLMLQARQSEDHPHRRRRRGLECDGKVNI 320

[XP_002818070.1](https://www.ncbi.nlm.nih.gov/protein/XP_002818070.1?report=genbank&log$=protalign&blast_rank=13&RID=0) 241 RIACEQCQESGASLVLLGKKKKKEEEGEGKKKGGGEGGAGADEEKEQSHRPFLMLQARQSEDHPHRRRRRGLECDGKVNI 320

[XP_032614156.1](https://www.ncbi.nlm.nih.gov/protein/XP_032614156.1?report=genbank&log$=protalign&blast_rank=14&RID=0) 241 RIACEQCQESGASLVLLGKKKKKEEEGEGKKKGGGEGGAGADEEKEQSHRPFLMLQARQSEDHPHRRRRRGLECDGKVNI 320

[NP_002183.1](https://www.ncbi.nlm.nih.gov/protein/NP_002183.1?report=genbank&log$=protalign&blast_rank=0&RID=0) 321 CCKKQFFVSFKDIGWNDWIIAPSGYHANYCEGECPSHIAGTSGSSLSFHSTVINHYRMRGHSPFANLKSCCVPTKLRPMS 400

[NP_032406.1](https://www.ncbi.nlm.nih.gov/protein/NP_032406.1?report=genbank&log$=protalign&blast_rank=1&RID=0) 319 CCKKQFFVSFKDIGWNDWIIAPSGYHANYCEGECPSHIAGTSGSSLSFHSTVINHYRMRGHSPFANLKSCCVPTKLRPMS 398

[NP_058824.1](https://www.ncbi.nlm.nih.gov/protein/NP_058824.1?report=genbank&log$=protalign&blast_rank=2&RID=0) 319 CCKKQFFVSFKDIGWNDWIIAPSGYHANYCEGECPSHIAGTSGSSLSFHSTVINHYRMRGHSPFANLKSCCVPTKLRPMS 398

[NP_776788.1](https://www.ncbi.nlm.nih.gov/protein/NP_776788.1?report=genbank&log$=protalign&blast_rank=3&RID=0) 320 CCKKQFFVSFKDIGWNDWIIAPSGYHANYCEGECPSHIAGTSGSSLSFHSTVINHYRMRGHSPFANLKSCCVPTKLRPMS 399

[NP_999193.1](https://www.ncbi.nlm.nih.gov/protein/NP_999193.1?report=genbank&log$=protalign&blast_rank=4&RID=0) 319 CCKKQFFVSFKDIGWNDWIIAPSGYHANYCEGECPSHIAGTSGSSLSFHSTVINHYRMRGHSPFANLKSCCVPTKLRPMS 398

[XP_540364.2](https://www.ncbi.nlm.nih.gov/protein/XP_540364.2?report=genbank&log$=protalign&blast_rank=5&RID=0) 319 CCKKQFFVSFKDIGWNDWIIAPSGYHANYCEGGCPSHIAGTSGSSLSFHSTVINHYRLRGHSPFTNLKSCCVPTKLRPMS 398

[NP_001075378.1](https://www.ncbi.nlm.nih.gov/protein/NP_001075378.1?report=genbank&log$=protalign&blast_rank=6&RID=0) 321 CCKKQFFVSFKDIGWNDWIIAPSGYHANYCEGECPSHIAGTSGSSLSFHSTVINQYRLRGHNPFANLKSCCVPTKLRPMS 400

[NP_001009458.1](https://www.ncbi.nlm.nih.gov/protein/NP_001009458.1?report=genbank&log$=protalign&blast_rank=7&RID=0) 320 CCKKQFYVSFKDIGWNDWIIAPSGYHANYCEGECPSHIAGTSGSSLSFHSTVINHYRMRGHSPFANLKSCCVPTKLRPMS 399

[XP_519063.2](https://www.ncbi.nlm.nih.gov/protein/XP_519063.2?report=genbank&log$=protalign&blast_rank=8&RID=0) 321 CCKKQFFVSFKDIGWNDWIIAPSGYHANYCEGECPSHIAGTSGSSLSFHSTVINHYRMRGHSPFANLKSCCVPTKLRPMS 400

[XP_028701686.1](https://www.ncbi.nlm.nih.gov/protein/XP_028701686.1?report=genbank&log$=protalign&blast_rank=9&RID=0) 321 CCKKQFFVSFKDIGWNDWIIAPSGYHANYCEGECPSHIAGTSGSSLSFHSTVINHYRMRGHSPFANLKSCCVPTKLRPMS 400

[NP_001009856.1](https://www.ncbi.nlm.nih.gov/protein/NP_001009856.1?report=genbank&log$=protalign&blast_rank=10&RID=0) 319 CCKKQFFVSFKDIGWNDWIIAPSGYHANYCEGECPSHIAGTSGSSLSFHSTVINHYRMRGHSPFANLKSCCVPTKLRPMS 398

[NP_001272510.1](https://www.ncbi.nlm.nih.gov/protein/NP_001272510.1?report=genbank&log$=protalign&blast_rank=11&RID=0) 320 CCKKQFYVSFKDIGWNDWIIAPSGYHANYCEGECPSHIAGTSGSSLSFHSTVINHYRMRGHSPFANLKSCCVPTKLRPMS 399

[XP_004045385.1](https://www.ncbi.nlm.nih.gov/protein/XP_004045385.1?report=genbank&log$=protalign&blast_rank=12&RID=0) 321 CCKKQFFVSFKDIGWNDWIIAPSGYHANYCEGECPSHIAGTSGSSLSFHSTVINHYRMRGHSPFANLKSCCVPTKLRPMS 400

[XP_002818070.1](https://www.ncbi.nlm.nih.gov/protein/XP_002818070.1?report=genbank&log$=protalign&blast_rank=13&RID=0) 321 CCKKQFFVSFKDIGWNDWIIAPSGYHANYCEGECPSHIAGTSGSSLSFHSTVINHYRMRGHSPFANLKSCCVPTKLRPMS 400

[XP_032614156.1](https://www.ncbi.nlm.nih.gov/protein/XP_032614156.1?report=genbank&log$=protalign&blast_rank=14&RID=0) 321 CCKKQFFVSFKDIGWNDWIIAPSGYHANYCEGECPSHIAGTSGSSLSFHSTVINHYRMRGHSPFANLKSCCVPTKLRPMS 400

[NP_002183.1](https://www.ncbi.nlm.nih.gov/protein/NP_002183.1?report=genbank&log$=protalign&blast_rank=0&RID=0) 401 MLYYDDGQNIIKKDIQNMIVEECGCS 426

[NP_032406.1](https://www.ncbi.nlm.nih.gov/protein/NP_032406.1?report=genbank&log$=protalign&blast_rank=1&RID=0) 399 MLYYDDGQNIIKKDIQNMIVEECGCS 424

[NP_058824.1](https://www.ncbi.nlm.nih.gov/protein/NP_058824.1?report=genbank&log$=protalign&blast_rank=2&RID=0) 399 MLYYDDGQNIIKKDIQNMIVEECGCS 424

[NP_776788.1](https://www.ncbi.nlm.nih.gov/protein/NP_776788.1?report=genbank&log$=protalign&blast_rank=3&RID=0) 400 MLYYDDGQNIIKKDIQNMIVEECGCS 425

[NP_999193.1](https://www.ncbi.nlm.nih.gov/protein/NP_999193.1?report=genbank&log$=protalign&blast_rank=4&RID=0) 399 MLYYDDGQNIIKKDIQNMIVEECGCS 424

[XP_540364.2](https://www.ncbi.nlm.nih.gov/protein/XP_540364.2?report=genbank&log$=protalign&blast_rank=5&RID=0) 399 MLYYDDGQNIIKKDIQNMIVEECGCS 424

[NP_001075378.1](https://www.ncbi.nlm.nih.gov/protein/NP_001075378.1?report=genbank&log$=protalign&blast_rank=6&RID=0) 401 MLYYDDGQNIIKKDIQNMIVEECGCS 426

[NP_001009458.1](https://www.ncbi.nlm.nih.gov/protein/NP_001009458.1?report=genbank&log$=protalign&blast_rank=7&RID=0) 400 MLYYDDGQNIIKKDIQNMIVEECGCS 425

[XP_519063.2](https://www.ncbi.nlm.nih.gov/protein/XP_519063.2?report=genbank&log$=protalign&blast_rank=8&RID=0) 401 MLYYDDGQNIIKKDIQNMIVEECGCS 426

[XP_028701686.1](https://www.ncbi.nlm.nih.gov/protein/XP_028701686.1?report=genbank&log$=protalign&blast_rank=9&RID=0) 401 MLYYDDGQNIIKKDIQNMIVEECGCS 426

[NP_001009856.1](https://www.ncbi.nlm.nih.gov/protein/NP_001009856.1?report=genbank&log$=protalign&blast_rank=10&RID=0) 399 MLYYDDGQNIIKKDIQNMIVEECGCS 424

[NP_001272510.1](https://www.ncbi.nlm.nih.gov/protein/NP_001272510.1?report=genbank&log$=protalign&blast_rank=11&RID=0) 400 MLYYDDGQNIIKKDIQNMIVEECGCS 425

[XP_004045385.1](https://www.ncbi.nlm.nih.gov/protein/XP_004045385.1?report=genbank&log$=protalign&blast_rank=12&RID=0) 401 MLYYDDGQNIIKKDIQNMIVEECGCS 426

[XP_002818070.1](https://www.ncbi.nlm.nih.gov/protein/XP_002818070.1?report=genbank&log$=protalign&blast_rank=13&RID=0) 401 MLYYDDGQNIIKKDIQNMIVEECGCS 426

[XP_032614156.1](https://www.ncbi.nlm.nih.gov/protein/XP_032614156.1?report=genbank&log$=protalign&blast_rank=14&RID=0) 401 MLYYDDGQNIIKKDIQNMIVEECGCS 426

INHBB

NP_002184.2 inhibin beta B chain preproprotein [Homo sapiens]

NP_032407.1 inhibin beta B chain preproprotein [Mus musculus]

NP_542949.1 inhibin beta B chain precursor [Rattus norvegicus]

NP_789822.2 inhibin beta B chain precursor [Bos taurus]

NP_001158314.1 inhibin beta B chain precursor [Sus scrofa]

XP_038281949.1 inhibin beta B chain [Canis lupus familiaris]

XP_023478506.1 inhibin beta B chain [Equus caballus]

XP_027820847.1 inhibin beta B chain [Ovis aries]

XP_016805107.1 inhibin beta B chain [Pan troglodytes]

XP_001090729.4 inhibin beta B chain [Macaca mulatta]

XP_003990762.4 inhibin beta B chain [Felis catus]

XP_017917150.1 PREDICTED: inhibin beta B chain [Capra hircus]

XP_004031753.1 inhibin beta B chain [Gorilla gorilla gorilla]

XP_024099269.1 inhibin beta B chain [Pongo abelii]

XP_032001809.1 inhibin beta B chain [Hylobates moloch]

[NP_002184.2](https://www.ncbi.nlm.nih.gov/protein/NP_002184.2?report=genbank&log$=protalign&blast_rank=0&RID=0) 1 MDGLPGRALGAACLLLLAAGWLGPEAWGSPTPPPTPAAP-PPPPPPGSPGGSQDTCTSCGG FRRPEELGRVDGDFLE 76

[NP_032407.1](https://www.ncbi.nlm.nih.gov/protein/NP_032407.1?report=genbank&log$=protalign&blast_rank=1&RID=0) 1 MDGLPGRALGAACLLLLVAGWLGPEAWGSPTPPPSPAAP-PPPPPPGAPGGSQDTCTSCGG[4]FRRPEELGRVDGDFLE 80

[NP_542949.1](https://www.ncbi.nlm.nih.gov/protein/NP_542949.1?report=genbank&log$=protalign&blast_rank=2&RID=0) 1 MDGLPGRALGAACLLLLAAGWLGPEAWGSPTPPPSPAAP-PPPPPPGAPGGSQDTCTSCGG[4]FRRPEELGRVDGDFLE 80

[NP_789822.2](https://www.ncbi.nlm.nih.gov/protein/NP_789822.2?report=genbank&log$=protalign&blast_rank=3&RID=0) 1 MDGLPGRALGAACLLMLAVGSLGPGVWGSPTPPPLPAAPqPPPPPPGAPGGSQDTCTSCGG FRRPEELGRVDGDFLE 77

[NP_001158314.1](https://www.ncbi.nlm.nih.gov/protein/NP_001158314.1?report=genbank&log$=protalign&blast_rank=4&RID=0) 1 MDGLPGRALGAACLLLLAAGWLGPEAWGSPTPPPSPAAP-PPPPPPGALGGSQDTCTSCGG FRRPEELGRLDGDFLE 76

[XP_038281949.1](https://www.ncbi.nlm.nih.gov/protein/XP_038281949.1?report=genbank&log$=protalign&blast_rank=5&RID=0) 1 MDGLPGRALGAACLLLLAAGWLGPEAWGSPTPPPSPAAP-PPPPPPGAPGGSQDTCTSCGG FRRPEELGRVDGDFLE 76

[XP_023478506.1](https://www.ncbi.nlm.nih.gov/protein/XP_023478506.1?report=genbank&log$=protalign&blast_rank=6&RID=0) 1 MDGLPGRALGAACLLLLAAGWLGPEAWGSPTPPPSPAAP-PPPPPPGAPGGSQDTCTSCGG FRRPEELGRVDRDFLE 76

[XP_027820847.1](https://www.ncbi.nlm.nih.gov/protein/XP_027820847.1?report=genbank&log$=protalign&blast_rank=7&RID=0) 1 MDGLPGRALGAACLVMLAVGWLGPGVWGSPTPPPSPAAPqPPPPPPGAPGGAQDTCTSCGG FRRPEELGRVDGDFLE 77

[XP_016805107.1](https://www.ncbi.nlm.nih.gov/protein/XP_016805107.1?report=genbank&log$=protalign&blast_rank=8&RID=0) 1 MDGLPGRALGAACLLLLAAGWLGPEAWGSPTPPPSPAAP-PPPPPPGAPGGSQDTCTSCGG FRRPEELGRVDGDFLE 76

[XP_001090729.4](https://www.ncbi.nlm.nih.gov/protein/XP_001090729.4?report=genbank&log$=protalign&blast_rank=9&RID=0) 1 MDGLPGRALGAACLLLLAAGWLGPEAWGSPTPPPSPAAP-PPPPPPGAPGGSQDTCTSCGG FRRPEELGRVDGDFLE 76

[XP_003990762.4](https://www.ncbi.nlm.nih.gov/protein/XP_003990762.4?report=genbank&log$=protalign&blast_rank=10&RID=0) 1 MDGLPGRALGAACLLLLAAGWLGPEAWGSPTPPPSPAAP-PPPPPPGAPGGSQDTCTSCGG FRRPEELGRVDGDFLE 76

[XP_017917150.1](https://www.ncbi.nlm.nih.gov/protein/XP_017917150.1?report=genbank&log$=protalign&blast_rank=11&RID=0) 1 MDGLPGRALGAACLVMLAVGWLGPGVWGSPTPPPSPAAPqPPPPPPGAPGGAQDTCTSCGG FRRPEELGRVDGDFLE 77

[XP_004031753.1](https://www.ncbi.nlm.nih.gov/protein/XP_004031753.1?report=genbank&log$=protalign&blast_rank=12&RID=0) 1 MDGLPGRALGAACLLLLAAGWLGPEAWGSPTPPPSPAAP-PPPPPPGAPGGSQDTCTSCGG FRRPEELGRVDGDFLE 76

[XP_024099269.1](https://www.ncbi.nlm.nih.gov/protein/XP_024099269.1?report=genbank&log$=protalign&blast_rank=13&RID=0) 1 MDGLPGRALGAACLLLLAAGWLGPEAWGSPTPPPSPAAP-PPPPPPGAPGGSQDTCTSCGG FRRPEELGRVDGDFLE 76

[XP_032001809.1](https://www.ncbi.nlm.nih.gov/protein/XP_032001809.1?report=genbank&log$=protalign&blast_rank=14&RID=0) 1 MDGLPGRALGAACLLLLAAGWLGPEAWGSPTPPPSPAAP-PPPPPPGAPGGSQDTCTSCGG FRRPEELGRVDGDFLE 76

[NP_002184.2](https://www.ncbi.nlm.nih.gov/protein/NP_002184.2?report=genbank&log$=protalign&blast_rank=0&RID=0) 77 AVKRHILSRLQMRGRPNITHAVPKAAMVTALRKLHAGKVREDGRVEIPHLDGHASPGADGQERVSEIISFAETDGLASSR 156

[NP_032407.1](https://www.ncbi.nlm.nih.gov/protein/NP_032407.1?report=genbank&log$=protalign&blast_rank=1&RID=0) 81 AVKRHILSRLQLRGRPNITHAVPKAAMVTALRKLHAGKVREDGRVEIPHLDGHASPGADGQERVSEIISFAETDGLASSR 160

[NP_542949.1](https://www.ncbi.nlm.nih.gov/protein/NP_542949.1?report=genbank&log$=protalign&blast_rank=2&RID=0) 81 AVKRHILSRLQLRGRPNITHAVPKAAMVTALRKLHAGKVREDGRVEIPHLDGHASPGADGQERVSEIISFAETDGLASSR 160

[NP_789822.2](https://www.ncbi.nlm.nih.gov/protein/NP_789822.2?report=genbank&log$=protalign&blast_rank=3&RID=0) 78 AVKRHILNRLQMRGRPNITHAVPKAAMVTALRKLHAGKVREDGRVEIPHLDGHASPGADGQERVSEIISFAETDGLASSR 157

[NP_001158314.1](https://www.ncbi.nlm.nih.gov/protein/NP_001158314.1?report=genbank&log$=protalign&blast_rank=4&RID=0) 77 AVKRHILNRLQMRGRPNITHAVPKAAMVTALRKLHAGKVREDGRVEIPHLDGHASPGADGQERVSEIISFAETDGLASSR 156

[XP_038281949.1](https://www.ncbi.nlm.nih.gov/protein/XP_038281949.1?report=genbank&log$=protalign&blast_rank=5&RID=0) 77 AVKRHILSRLQMRGRPNITHAVPKAAMVTALRKLHAGKVREDGRVEIPHLDGHASPGSDGQERVSEIISFAETDGLASSR 156

[XP_023478506.1](https://www.ncbi.nlm.nih.gov/protein/XP_023478506.1?report=genbank&log$=protalign&blast_rank=6&RID=0) 77 AVKRHILSRLQMRGRPNITHAVPKAAMVTALRKLHAGKVREDGRVEIPHLDGHASPGADGQERVSEIISFAETDGLASSR 156

[XP_027820847.1](https://www.ncbi.nlm.nih.gov/protein/XP_027820847.1?report=genbank&log$=protalign&blast_rank=7&RID=0) 78 AVKRHILSRLQMRGRPNITHAVPKAAMVTALRKLHAGKVREDGRVEIPHLDGHASPGADGPERVSEIISFAETDGLASSR 157

[XP_016805107.1](https://www.ncbi.nlm.nih.gov/protein/XP_016805107.1?report=genbank&log$=protalign&blast_rank=8&RID=0) 77 AVKRHILSRLQMRGRPNITHAVPKAAMVTALRKLHAGKVREDGRVEIPHLDGHASPGADGQERVSEIISFAETDGLASSR 156

[XP_001090729.4](https://www.ncbi.nlm.nih.gov/protein/XP_001090729.4?report=genbank&log$=protalign&blast_rank=9&RID=0) 77 AVKRHILSRLQMRGRPNITHAVPKAAMVTALRKLHAGKVREDGRVEIPHLDGHASPGADGQERVSEIISFAETDGLASSR 156

[XP_003990762.4](https://www.ncbi.nlm.nih.gov/protein/XP_003990762.4?report=genbank&log$=protalign&blast_rank=10&RID=0) 77 AVKRHILSRLQMRGRPNITHAVPKAAMVTALRKLHAGKVREDGRVEIPHLDGHASPGADGQERVSEIISFAETDGLASSR 156

[XP_017917150.1](https://www.ncbi.nlm.nih.gov/protein/XP_017917150.1?report=genbank&log$=protalign&blast_rank=11&RID=0) 78 AVKRHILSRLQMRGRPNITHAVPKAAMVTALRKLHAGKVREDGRVEIPHLDGHASPGADGPERVSEIISFAETDGLASSR 157

[XP_004031753.1](https://www.ncbi.nlm.nih.gov/protein/XP_004031753.1?report=genbank&log$=protalign&blast_rank=12&RID=0) 77 AVKRHILSRLQMRGRPNITHAVPKAAMVTALRKLHAGKVREDGRVEIPHLDGHASPGADGQERVSEIISFAETDGLASSR 156

[XP_024099269.1](https://www.ncbi.nlm.nih.gov/protein/XP_024099269.1?report=genbank&log$=protalign&blast_rank=13&RID=0) 77 AVKRHILSRLQMRGRPNITHAVPKAAMVTALRKLHAGKVREDGRVEIPHLDGHASPGADGQERVSEIISFAETDGLASSR 156

[XP_032001809.1](https://www.ncbi.nlm.nih.gov/protein/XP_032001809.1?report=genbank&log$=protalign&blast_rank=14&RID=0) 77 AVKRHILSRLQMRGRPNITHAVPKAAMVTALRKLHAGKVREDGRVEIPHLDGHASPAADGQERVSEIISFAETDGLASSR 156

[NP_002184.2](https://www.ncbi.nlm.nih.gov/protein/NP_002184.2?report=genbank&log$=protalign&blast_rank=0&RID=0) 157 VRLYFFISNEGNQNLFVVQASLWLYLKLLPYVLEKGSRRKVRVKVYFQEQGHGDRWNMVEKRVDLKRSGWHTFPLTEAIQ 236

[NP_032407.1](https://www.ncbi.nlm.nih.gov/protein/NP_032407.1?report=genbank&log$=protalign&blast_rank=1&RID=0) 161 VRLYFFVSNEGNQNLFVVQASLWLYLKLLPYVLEKGSRRKVRVKVYFQEQGHGDRWNVVEKKVDLKRSGWHTFPITEAIQ 240

[NP_542949.1](https://www.ncbi.nlm.nih.gov/protein/NP_542949.1?report=genbank&log$=protalign&blast_rank=2&RID=0) 161 VRLYFFVSNEGNQNLFVVQASLWLYLKLLPYVLEKGSRRKVRVKVYFQEQGHGDRWNVVEKKVDLKRSGWHTFPITEAIQ 240

[NP_789822.2](https://www.ncbi.nlm.nih.gov/protein/NP_789822.2?report=genbank&log$=protalign&blast_rank=3&RID=0) 158 VRLYFFISNEGNQNLFVVQASLWLYLKLLPYVLEKGGRRKVRVKVYFQEQGPGDRWAAVEKRVDLKRSGWHTFPLTEPIQ 237

[NP_001158314.1](https://www.ncbi.nlm.nih.gov/protein/NP_001158314.1?report=genbank&log$=protalign&blast_rank=4&RID=0) 157 VRLYFFISNEGNQNLFVVQASLWLYLKLLPYVLEKGSRRKVRVKVYFQEPGHGDRWDVVEKRVDLKRSGWHTLPLTEAIQ 236

[XP_038281949.1](https://www.ncbi.nlm.nih.gov/protein/XP_038281949.1?report=genbank&log$=protalign&blast_rank=5&RID=0) 157 VRLYFFVSNEGNQNLFVVQASLWLYLKLLPYVLEKGSRRKVRVKVYFQEQGQGDRWNAVEKKVDLKRSGWHTFPLTEAIQ 236

[XP_023478506.1](https://www.ncbi.nlm.nih.gov/protein/XP_023478506.1?report=genbank&log$=protalign&blast_rank=6&RID=0) 157 VRLYFFISNEGNQNLFVVQASLWLYLKLLPYVLEKGSRRKVRVKVYFQEQGHGDRWNVVEKKVDLKRSGWHTFPLTEAIQ 236

[XP_027820847.1](https://www.ncbi.nlm.nih.gov/protein/XP_027820847.1?report=genbank&log$=protalign&blast_rank=7&RID=0) 158 VRLYFFISNEGNQNLFVVQASLWLYLKLLPYVLEKGGRRKVRVKVYFQEQGPGDRWAAVEKRVDLKRSGWHTFPLTEPIQ 237

[XP_016805107.1](https://www.ncbi.nlm.nih.gov/protein/XP_016805107.1?report=genbank&log$=protalign&blast_rank=8&RID=0) 157 VRLYFFISNEGNQNLFVVQASLWLYLKLLPYVLEKGSRRKVRVKVYFQEQGHGDRWNMVEKRVDLKRSGWHTFPLTEAIQ 236

[XP_001090729.4](https://www.ncbi.nlm.nih.gov/protein/XP_001090729.4?report=genbank&log$=protalign&blast_rank=9&RID=0) 157 VRLYFFISNEGNQNLFVVQASLWLYLKLLPYVLEKGSRRKVRVKVYFQEQGHGDRWNMVEKRVDLKRSGWHTFPLTEAIQ 236

[XP_003990762.4](https://www.ncbi.nlm.nih.gov/protein/XP_003990762.4?report=genbank&log$=protalign&blast_rank=10&RID=0) 157 VRLYFFISNEGNQNLFVVQASLWLYLKLLPYVLEKGSRRKVRVKVYFQEQGHGDRWNVVEKKVDLKRSGWHTFPLTEAIQ 236

[XP_017917150.1](https://www.ncbi.nlm.nih.gov/protein/XP_017917150.1?report=genbank&log$=protalign&blast_rank=11&RID=0) 158 VRLYFFISNEGNQNLFVVQASLWLYLKLLPYVLEKGGRRKVRVKVYFQEQGPGDRWAAVEKRVDLKRSGWHTFPLTEPIQ 237

[XP_004031753.1](https://www.ncbi.nlm.nih.gov/protein/XP_004031753.1?report=genbank&log$=protalign&blast_rank=12&RID=0) 157 VRLYFFISNEGNQNLFVVQASLWLYLKLLPYVLEKGSRRKVRVKVYFQEQGHGDRWNMVEKRVDLKRSGWHTFPLTEAIQ 236

[XP_024099269.1](https://www.ncbi.nlm.nih.gov/protein/XP_024099269.1?report=genbank&log$=protalign&blast_rank=13&RID=0) 157 VRLYFFISNEGNQNLFVVQASLWLYLKLLPYVLEKGSRRKVRVKVYFQEQGHGDRWNMVEKRVDLKRSGWHTFPLTEAIQ 236

[XP_032001809.1](https://www.ncbi.nlm.nih.gov/protein/XP_032001809.1?report=genbank&log$=protalign&blast_rank=14&RID=0) 157 VRLYFFISNEGNQNLFVVQASLWLYLKLLPYVLEKGSRRKVRVKVYFQEQGHGDRWNMVEKRVDLKRSGWHTFPLTEAIQ 236

[NP_002184.2](https://www.ncbi.nlm.nih.gov/protein/NP_002184.2?report=genbank&log$=protalign&blast_rank=0&RID=0) 237 ALFERGERRLNLDVQCDSCQELAVVPVFVDPGEESHRPFVVVQARLGDSRHRIRKRGLECDGRTNLCCRQQFFIDFRLIG 316

[NP_032407.1](https://www.ncbi.nlm.nih.gov/protein/NP_032407.1?report=genbank&log$=protalign&blast_rank=1&RID=0) 241 ALFERGERRLNLDVQCDSCQELAVVPVFVDPGEESHRPFVVVQARLGDSRHRIRKRGLECDGRTSLCCRQQFFIDFRLIG 320

[NP_542949.1](https://www.ncbi.nlm.nih.gov/protein/NP_542949.1?report=genbank&log$=protalign&blast_rank=2&RID=0) 241 ALFERGERRLNLDVQCDSCQELAVVPVFVDPGEESHRPFVVVQARLGDSRHRIRKRGLECDGRTSLCCRQQFFIDFRLIG 320

[NP_789822.2](https://www.ncbi.nlm.nih.gov/protein/NP_789822.2?report=genbank&log$=protalign&blast_rank=3&RID=0) 238 ALFSRGERRLSLDVQCDSCRELAVVPVFVDPGEESHRPFVVVQARLGDSRHRIRKRGLECDGRTNLCCRQQFFIDFRLIG 317

[NP_001158314.1](https://www.ncbi.nlm.nih.gov/protein/NP_001158314.1?report=genbank&log$=protalign&blast_rank=4&RID=0) 237 ALFERGERRLNLDVQCDGCQELAVVPVFVDPGEESHRPFVVVQARLVDSRHRIRKRGLECDGRTNLCCRQQFFIDFRLIG 316

[XP_038281949.1](https://www.ncbi.nlm.nih.gov/protein/XP_038281949.1?report=genbank&log$=protalign&blast_rank=5&RID=0) 237 ALFERGERRLSLDVQCDGCQELAVVPVFVDPGEESHRPFVVVQARLGDSRHRIRKRGLECDGRTNLCCRQQFFIDFRLIG 316

[XP_023478506.1](https://www.ncbi.nlm.nih.gov/protein/XP_023478506.1?report=genbank&log$=protalign&blast_rank=6&RID=0) 237 ALFERGERRLNLDVQCDGCQELAVVPVFVDPGEESHRPFVVVQARLGDSRHRIRKRGLECDGRTNLCCRQQFFIDFRLIG 316

[XP_027820847.1](https://www.ncbi.nlm.nih.gov/protein/XP_027820847.1?report=genbank&log$=protalign&blast_rank=7&RID=0) 238 ALFSRGERRLSLDVQCDGCRELAVVPVFVDPGEESHRPFVVVQARLGDSRHRIRKRGLECDGRTSLCCRQQFFIDFRLIG 317

[XP_016805107.1](https://www.ncbi.nlm.nih.gov/protein/XP_016805107.1?report=genbank&log$=protalign&blast_rank=8&RID=0) 237 ALFERGERRLNLDVQCDSCQELAVVPVFVDPAEESHRPFVVVQARLGDSRHRIRKRGLECDGRTNLCCRQQFFIDFRLIG 316

[XP_001090729.4](https://www.ncbi.nlm.nih.gov/protein/XP_001090729.4?report=genbank&log$=protalign&blast_rank=9&RID=0) 237 ALFERGERRLNLDVQCDSCQELAVVPVFVDPGEESHRPFVVVQARLGDSRHRIRKRGLECDGRTNLCCRQQFFIDFRLIG 316

[XP_003990762.4](https://www.ncbi.nlm.nih.gov/protein/XP_003990762.4?report=genbank&log$=protalign&blast_rank=10&RID=0) 237 SLFERGERRLNLDVQCDGCQELAVVPVFVDPGEESHRPFVVVQARLGDSRHRIRKRGLECDGRTNLCCRQQFFIDFRLIG 316

[XP_017917150.1](https://www.ncbi.nlm.nih.gov/protein/XP_017917150.1?report=genbank&log$=protalign&blast_rank=11&RID=0) 238 ALFSRGERRLSLDVQCDGCRELAVVPVFVDPGEESHRPFVVVQARLGDSRHRIRKRGLECDGRTNLCCRQQFFIDFRLIG 317

[XP_004031753.1](https://www.ncbi.nlm.nih.gov/protein/XP_004031753.1?report=genbank&log$=protalign&blast_rank=12&RID=0) 237 ALFERGERRLNLDVQCDSCQELAVVPVFVDPGEESHRPFVVVQARLGDSRHRIRKRGLECDGRTNLCCRQQFFIDFRLIG 316

[XP_024099269.1](https://www.ncbi.nlm.nih.gov/protein/XP_024099269.1?report=genbank&log$=protalign&blast_rank=13&RID=0) 237 ALFERGERRLNLDVQCDSCQELAVVPVFVDPGEESHRPFVVVQARLGDSRHRIRKRGLECDGRTNLCCRQQFFIDFRLIG 316

[XP_032001809.1](https://www.ncbi.nlm.nih.gov/protein/XP_032001809.1?report=genbank&log$=protalign&blast_rank=14&RID=0) 237 ALFERGERRLNLDVQCDSCQELAVVPVFVDPGEESHRPFVVVQARLGDSRHRIRKRGLECDGRTNLCCRQQFFIDFRLIG 316

[NP_002184.2](https://www.ncbi.nlm.nih.gov/protein/NP_002184.2?report=genbank&log$=protalign&blast_rank=0&RID=0) 317 WNDWIIAPTGYYGNYCEGSCPAYLAGVPGSASSFHTAVVNQYRMRGLNPGTVNSCCIPTKLSTMSMLYFDDEYNIVKRDV 396

[NP_032407.1](https://www.ncbi.nlm.nih.gov/protein/NP_032407.1?report=genbank&log$=protalign&blast_rank=1&RID=0) 321 WNDWIIAPTGYYGNYCEGSCPAYLAGVPGSASSFHTAVVNQYRMRGLNPGPVNSCCIPTKLSSMSMLYFDDEYNIVKRDV 400

[NP_542949.1](https://www.ncbi.nlm.nih.gov/protein/NP_542949.1?report=genbank&log$=protalign&blast_rank=2&RID=0) 321 WNDWIIAPTGYYGNYCEGSCPAYLAGVPGSASSFHTAVVNQYRMRGLNPGPVNSCCIPTKLSSMSMLYFDDEYNIVKRDV 400

[NP_789822.2](https://www.ncbi.nlm.nih.gov/protein/NP_789822.2?report=genbank&log$=protalign&blast_rank=3&RID=0) 318 WNDWIIAPTGYYGNYCEGSCPAYLAGVPGSASSFHTAVVNQYRMRGLNPGTVNSCCIPTKLSTMSMLYFDDEYNIVKRDV 397

[NP_001158314.1](https://www.ncbi.nlm.nih.gov/protein/NP_001158314.1?report=genbank&log$=protalign&blast_rank=4&RID=0) 317 WSDWIIAPTGYYGNYCEGSCPAYLAGVPGSASSFHTAVVNQYRMRGLNPGTVNSCCIPTKLSTMSMLYFDDEYNIVKRDV 396

[XP_038281949.1](https://www.ncbi.nlm.nih.gov/protein/XP_038281949.1?report=genbank&log$=protalign&blast_rank=5&RID=0) 317 WNDWIIAPTGYYGNYCEGSCPAYLAGVPGSASSFHTAVVNQYRMRGLNPGTVNSCCIPTKLSTMSMLYFDDEYNIVKRDV 396

[XP_023478506.1](https://www.ncbi.nlm.nih.gov/protein/XP_023478506.1?report=genbank&log$=protalign&blast_rank=6&RID=0) 317 WNDWIIAPIGYYGNYCEGSCPAYLAGVPGSASSFHTAVVNQYRMRGLNPGTVNSCCIPTKLSTMSMLYFDDEYNIVKRDV 396

[XP_027820847.1](https://www.ncbi.nlm.nih.gov/protein/XP_027820847.1?report=genbank&log$=protalign&blast_rank=7&RID=0) 318 WNDWIIAPTGYYGNYCEGSCPAYLAGVPGSASSFHTAVVNQYRMRGLNPGTVNSCCIPTKLSTMSMLYFDDEYNIVKRDV 397

[XP_016805107.1](https://www.ncbi.nlm.nih.gov/protein/XP_016805107.1?report=genbank&log$=protalign&blast_rank=8&RID=0) 317 WNDWIIAPTGYYGNYCEGSCPAYLAGVPGSASSFHTAVVNQYRMRGLNPGTVNSCCIPTKLSTMSMLYFDDEYNIVKRDV 396

[XP_001090729.4](https://www.ncbi.nlm.nih.gov/protein/XP_001090729.4?report=genbank&log$=protalign&blast_rank=9&RID=0) 317 WNDWIIAPTGYYGNYCEGSCPAYLAGVPGSASSFHTAVVNQYRMRGLNPGAVNSCCIPTKLSTMSMLYFDDEYNIVKRDV 396

[XP_003990762.4](https://www.ncbi.nlm.nih.gov/protein/XP_003990762.4?report=genbank&log$=protalign&blast_rank=10&RID=0) 317 WNDWIIAPTGYYGNYCEGSCPAYLAGVPGSASSFHTAVVNQYRMRGLNPGTVNSCCIPTKLSTMSMLYFDDEYNIVKRDV 396

[XP_017917150.1](https://www.ncbi.nlm.nih.gov/protein/XP_017917150.1?report=genbank&log$=protalign&blast_rank=11&RID=0) 318 WNDWIIAPTGYYGNYCEGSCPAYLAGVPGSASSFHTAVVNQYRMRGLNPGTVNSCCIPTKLSTMSMLYFDDEYNIVKRDV 397

[XP_004031753.1](https://www.ncbi.nlm.nih.gov/protein/XP_004031753.1?report=genbank&log$=protalign&blast_rank=12&RID=0) 317 WNDWIIAPTGYYGNYCEGSCPAYLAGVPGSASSFHTAVVNQYRMRGLNPGTVNSCCIPTKLSTMSMLYFDDEYNIVKRDV 396

[XP_024099269.1](https://www.ncbi.nlm.nih.gov/protein/XP_024099269.1?report=genbank&log$=protalign&blast_rank=13&RID=0) 317 WNDWIIAPTGYYGNYCEGSCPAYLAGVPGSASSFHTAVVNQYRMRGLNPGTVNSCCIPTKLSTMSMLYFDDEYNIVKRDV 396

[XP_032001809.1](https://www.ncbi.nlm.nih.gov/protein/XP_032001809.1?report=genbank&log$=protalign&blast_rank=14&RID=0) 317 WNDWIIAPTGYYGNYCEGSCPAYLAGVPGSASSFHTAVVNQYRMRGLNPGTVNSCCIPTKLSTMSMLYFDDEYNIVKRDV 396

[NP_002184.2](https://www.ncbi.nlm.nih.gov/protein/NP_002184.2?report=genbank&log$=protalign&blast_rank=0&RID=0) 397 PNMIVEECGCA 407

[NP_032407.1](https://www.ncbi.nlm.nih.gov/protein/NP_032407.1?report=genbank&log$=protalign&blast_rank=1&RID=0) 401 PNMIVEECGCA 411

[NP_542949.1](https://www.ncbi.nlm.nih.gov/protein/NP_542949.1?report=genbank&log$=protalign&blast_rank=2&RID=0) 401 PNMIVEECGCA 411

[NP_789822.2](https://www.ncbi.nlm.nih.gov/protein/NP_789822.2?report=genbank&log$=protalign&blast_rank=3&RID=0) 398 PNMIVEECGCA 408

[NP_001158314.1](https://www.ncbi.nlm.nih.gov/protein/NP_001158314.1?report=genbank&log$=protalign&blast_rank=4&RID=0) 397 PNMIVEECGCA 407

[XP_038281949.1](https://www.ncbi.nlm.nih.gov/protein/XP_038281949.1?report=genbank&log$=protalign&blast_rank=5&RID=0) 397 PNMIVEECGCA 407

[XP_023478506.1](https://www.ncbi.nlm.nih.gov/protein/XP_023478506.1?report=genbank&log$=protalign&blast_rank=6&RID=0) 397 PNMIVEECGCA 407

[XP_027820847.1](https://www.ncbi.nlm.nih.gov/protein/XP_027820847.1?report=genbank&log$=protalign&blast_rank=7&RID=0) 398 PNMIVEECGCA 408

[XP_016805107.1](https://www.ncbi.nlm.nih.gov/protein/XP_016805107.1?report=genbank&log$=protalign&blast_rank=8&RID=0) 397 PNMIVEECGCA 407

[XP_001090729.4](https://www.ncbi.nlm.nih.gov/protein/XP_001090729.4?report=genbank&log$=protalign&blast_rank=9&RID=0) 397 PNMIVEECGCA 407

[XP_003990762.4](https://www.ncbi.nlm.nih.gov/protein/XP_003990762.4?report=genbank&log$=protalign&blast_rank=10&RID=0) 397 PNMIVEECGCA 407

[XP_017917150.1](https://www.ncbi.nlm.nih.gov/protein/XP_017917150.1?report=genbank&log$=protalign&blast_rank=11&RID=0) 398 PNMIVEECGCA 408

[XP_004031753.1](https://www.ncbi.nlm.nih.gov/protein/XP_004031753.1?report=genbank&log$=protalign&blast_rank=12&RID=0) 397 PNMIVEECGCA 407

[XP_024099269.1](https://www.ncbi.nlm.nih.gov/protein/XP_024099269.1?report=genbank&log$=protalign&blast_rank=13&RID=0) 397 PNMIVEECGCA 407

[XP_032001809.1](https://www.ncbi.nlm.nih.gov/protein/XP_032001809.1?report=genbank&log$=protalign&blast_rank=14&RID=0) 397 PNMIVEECGCA 407

GDF8

NP_005250.1 growth/differentiation factor 8 preproprotein [Homo sapiens]

NP_034964.1 growth/differentiation factor 8 preproprotein [Mus musculus]

NP_062024.1 growth/differentiation factor 8 precursor [Rattus norvegicus]

NP_001001525.1 growth/differentiation factor 8 precursor [Bos taurus]

NP_999600.2 growth/differentiation factor 8 precursor [Sus scrofa]

NP_001002959.1 growth/differentiation factor 8 precursor [Canis lupus familiaris]

NP_001075286.1 growth/differentiation factor 8 precursor [Equus caballus]

NP_001009428.1 growth/differentiation factor 8 precursor [Ovis aries]

NP_001073388.1 growth/differentiation factor 8 precursor [Pan troglodytes]

NP_001073588.1 growth/differentiation factor 8 precursor [Macaca mulatta]

XP_003991021.1 growth/differentiation factor 8 [Felis catus]

NP_001272666.1 growth/differentiation factor 8 precursor [Capra hircus]

XP_018877573.1 growth/differentiation factor 8 [Gorilla gorilla gorilla]

XP_002812720.1 growth/differentiation factor 8 [Pongo abelii]

XP_032609257.1 growth/differentiation factor 8 [Hylobates moloch]

[NP_005250.1](https://www.ncbi.nlm.nih.gov/protein/NP_005250.1?report=genbank&log$=protalign&blast_rank=0&RID=0) 1 -MQKLQLCVYIYLFMLIVAGPVDLNENSEQKENVEKEGLCNACTWRQNTKSSRIEAIKIQILSKLRLETAPNISKDVIRQ 79

[NP_034964.1](https://www.ncbi.nlm.nih.gov/protein/NP_034964.1?report=genbank&log$=protalign&blast_rank=1&RID=0) 1 mMQKLQMYVYIYLFMLIAAGPVDLNEGSEREENVEKEGLCNACAWRQNTRYSRIEAIKIQILSKLRLETAPNISKDAIRQ 80

[NP_062024.1](https://www.ncbi.nlm.nih.gov/protein/NP_062024.1?report=genbank&log$=protalign&blast_rank=2&RID=0) 1 mIQKPQMYVYIYLFVLIAAGPVDLNEDSEREANVEKEGLCNACAWRQNTRYSRIEAIKIQILSKLRLETAPNISKDAIRQ 80

[NP_001001525.1](https://www.ncbi.nlm.nih.gov/protein/NP_001001525.1?report=genbank&log$=protalign&blast_rank=3&RID=0) 1 -MQKLQISVYIYLFMLIVAGPVDLNENSEQKENVEKEGLCNACLWRENTTSSRLEAIKIQILSKLRLETAPNISKDAIRQ 79

[NP_999600.2](https://www.ncbi.nlm.nih.gov/protein/NP_999600.2?report=genbank&log$=protalign&blast_rank=4&RID=0) 1 -MQKLQIYVYIYLFMLIVAGPVDLNENSEQKENVEKEGLCNACMWRQNTKSSRLEAIKIQILSKLRLETAPNISKDAIRQ 79

[NP_001002959.1](https://www.ncbi.nlm.nih.gov/protein/NP_001002959.1?report=genbank&log$=protalign&blast_rank=5&RID=0) 1 -MQRLQICVYIYLFVLIVAGPVDLSENSEQKENVEKEGLCNACMWRQNTKSSRIEAIKIQILSKLRLETAPNISRDAVRQ 79

[NP_001075286.1](https://www.ncbi.nlm.nih.gov/protein/NP_001075286.1?report=genbank&log$=protalign&blast_rank=6&RID=0) 1 -MQKLQISVYIYLFVLILAGPVDLNENSEQKENVEKEGLCNACTWRQNTKSSRIEAIKIQILSKLRLETAPNISKDAIRQ 79

[NP_001009428.1](https://www.ncbi.nlm.nih.gov/protein/NP_001009428.1?report=genbank&log$=protalign&blast_rank=7&RID=0) 1 -MQKLQIFVYIYLFMLLVAGPVDLNENSEQKENVEKKGLCNACLWRQNNKSSRLEAIKIQILSKLRLETAPNISKDAIRQ 79

[NP_001073388.1](https://www.ncbi.nlm.nih.gov/protein/NP_001073388.1?report=genbank&log$=protalign&blast_rank=8&RID=0) 1 -MQKLQLCVYIYLFMLIVAGPVDLNENSEQKENVEKEGLCNACTWRQNTKSSRIEAIKIQILSKLRLETAPNISKDAIRQ 79

[NP_001073588.1](https://www.ncbi.nlm.nih.gov/protein/NP_001073588.1?report=genbank&log$=protalign&blast_rank=9&RID=0) 1 -MQKLQLCVYIYLFMLIVAGPVDLNENSEQKENVEKEGLCNACTWRQNTKSSRIEAIKIQILSKLRLETAPNISKDAIRQ 79

[XP_003991021.1](https://www.ncbi.nlm.nih.gov/protein/XP_003991021.1?report=genbank&log$=protalign&blast_rank=10&RID=0) 1 -MQKLQIYVYIYLFMLIVAGPVDLNENSEQKENVEKEGLCNACTWRQNTKSSRIEAIKIQILSKLRLETAPNISKDAIRQ 79

[NP_001272666.1](https://www.ncbi.nlm.nih.gov/protein/NP_001272666.1?report=genbank&log$=protalign&blast_rank=11&RID=0) 1 -MQKLQIFVYIYLFMLLVAGPVDLNENSEQKENVEKKGLCNACLWRQNNKSSRLEAIKIQILSKLRLETAPNISKDAIRQ 79

[XP_018877573.1](https://www.ncbi.nlm.nih.gov/protein/XP_018877573.1?report=genbank&log$=protalign&blast_rank=12&RID=0) 1 -MQKLQLCVYIYLFMLIVAGPVDLNENSEQKENVEKEGLCNACTWRQNTKSSRIEAIKIQILSKLRLETAPNISKDAIRQ 79

[XP_002812720.1](https://www.ncbi.nlm.nih.gov/protein/XP_002812720.1?report=genbank&log$=protalign&blast_rank=13&RID=0) 1 -MQKLQLCVYIYLFMLIVAGPVDLNENSEQKENVEKEGLCNACTWRQNTKSSRIEAIKIQILSKLRLETAPNISKDAIRQ 79

[XP_032609257.1](https://www.ncbi.nlm.nih.gov/protein/XP_032609257.1?report=genbank&log$=protalign&blast_rank=14&RID=0) 1 -MQKLQLCVYIYLFMLIVAGPVDLNENSEQKENVEKEGLCNACTWRQNTKSSRIEAIKIQILSKLRLETAPNISKDAIRQ 79

[NP_005250.1](https://www.ncbi.nlm.nih.gov/protein/NP_005250.1?report=genbank&log$=protalign&blast_rank=0&RID=0) 80 LLPKAPPLRELIDQYDVQRDDSSDGSLEDDDYHATTETIITMPTESDFLMQVDGKPKCCFFKFSSKIQYNKVVKAQLWIY 159

[NP_034964.1](https://www.ncbi.nlm.nih.gov/protein/NP_034964.1?report=genbank&log$=protalign&blast_rank=1&RID=0) 81 LLPRAPPLRELIDQYDVQRDDSSDGSLEDDDYHATTETIITMPTESDFLMQADGKPKCCFFKFSSKIQYNKVVKAQLWIY 160

[NP_062024.1](https://www.ncbi.nlm.nih.gov/protein/NP_062024.1?report=genbank&log$=protalign&blast_rank=2&RID=0) 81 LLPRAPPLRELIDQYDVQRDDSSDGSLEDDDYHATTETIITMPTESDFLMQADGKPKCCFFKFSSKIQYNKVVKAQLWIY 160

[NP_001001525.1](https://www.ncbi.nlm.nih.gov/protein/NP_001001525.1?report=genbank&log$=protalign&blast_rank=3&RID=0) 80 LLPKAPPLLELIDQFDVQRDASSDGSLEDDDYHARTETVITMPTESDLLTQVEGKPKCCFFKFSSKIQYNKLVKAQLWIY 159

[NP_999600.2](https://www.ncbi.nlm.nih.gov/protein/NP_999600.2?report=genbank&log$=protalign&blast_rank=4&RID=0) 80 LLPKAPPLRELIDQYDVQRDDSSDGSLEDDDYHATTETIITMPTESDLLMQVEGKPKCCFFKFSSKIQYNKVVKAQLWIY 159

[NP_001002959.1](https://www.ncbi.nlm.nih.gov/protein/NP_001002959.1?report=genbank&log$=protalign&blast_rank=5&RID=0) 80 LLPRAPPLRELIDQYDVQRDDSSDGSLEDDDYHATTETVIAMPAETDLLMQVEGKPKCCFFKFSSKIQYNKVVKAQLWIY 159

[NP_001075286.1](https://www.ncbi.nlm.nih.gov/protein/NP_001075286.1?report=genbank&log$=protalign&blast_rank=6&RID=0) 80 LLPKAPPLRELIDQYDVQRDDSSDGSLEDDDYHATTETIITMPTESDLLMQVEGKPKCCFFKFSSKIQYNKVVKAQLWIY 159

[NP_001009428.1](https://www.ncbi.nlm.nih.gov/protein/NP_001009428.1?report=genbank&log$=protalign&blast_rank=7&RID=0) 80 LLPKAPPLRELIDQYDVQRDDSSDGSLEDDDYHVTTETVITMPTESDLLAEVQEKPKCCFFKFSSKIQHNKVVKAQLWIY 159

[NP_001073388.1](https://www.ncbi.nlm.nih.gov/protein/NP_001073388.1?report=genbank&log$=protalign&blast_rank=8&RID=0) 80 LLPKAPPLRELIDQYDVQRDDSSDGSLEDDDYHATTETIITMPTESDFLMQVDGKPKCCFFKFSSKIQYNKVVKAQLWIY 159

[NP_001073588.1](https://www.ncbi.nlm.nih.gov/protein/NP_001073588.1?report=genbank&log$=protalign&blast_rank=9&RID=0) 80 LLPKAPPLRELIDQYDVQRDDSSDGSLEDDDYHATTETIITMPTESDFLMQVDGKPKCCFFKFSSKIQYNKVVKAQLWIY 159

[XP_003991021.1](https://www.ncbi.nlm.nih.gov/protein/XP_003991021.1?report=genbank&log$=protalign&blast_rank=10&RID=0) 80 LLPKAPPLRELIDQYDVQRDDSSDGSLEDDDYHATTETIITMPTESDLLMQVEGKPKCCFFKFSSKIQYNKVVKAQLWIY 159

[NP_001272666.1](https://www.ncbi.nlm.nih.gov/protein/NP_001272666.1?report=genbank&log$=protalign&blast_rank=11&RID=0) 80 LLPKAPPLRELIDQYDVQRDDSSDGSLEDDDYHVTTETVITMPTESDLLAEVQEKPKCCFFKFSSKIQHNKVVKAQLWIY 159

[XP_018877573.1](https://www.ncbi.nlm.nih.gov/protein/XP_018877573.1?report=genbank&log$=protalign&blast_rank=12&RID=0) 80 LLPKAPPLRELIDQYDVQRDDSSDGSLEDDDYHATTETIITMPTESDFLMQVDGKPKCCFFKFSSKIQYNKVVKAQLWIY 159

[XP_002812720.1](https://www.ncbi.nlm.nih.gov/protein/XP_002812720.1?report=genbank&log$=protalign&blast_rank=13&RID=0) 80 LLPKAPPLRELIDQYDVQRDDSSDGSLEDDDYHATTETIITMPTESDFLMQVDGKPKCCFFKFSSKIQYNKVVKAQLWIY 159

[XP_032609257.1](https://www.ncbi.nlm.nih.gov/protein/XP_032609257.1?report=genbank&log$=protalign&blast_rank=14&RID=0) 80 LLPKAPPLRELIDQYDVQRDDSSDGSLEDDDYHATTETIITMPTESDFLMQVDGKPKCCFFKFSSKIQYNKVVKAQLWIY 159

[NP_005250.1](https://www.ncbi.nlm.nih.gov/protein/NP_005250.1?report=genbank&log$=protalign&blast_rank=0&RID=0) 160 LRPVETPTTVFVQILRLIKPMKDGTRYTGIRSLKLDMNPGTGIWQSIDVKTVLQNWLKQPESNLGIEIKALDENGHDLAV 239

[NP_034964.1](https://www.ncbi.nlm.nih.gov/protein/NP_034964.1?report=genbank&log$=protalign&blast_rank=1&RID=0) 161 LRPVKTPTTVFVQILRLIKPMKDGTRYTGIRSLKLDMSPGTGIWQSIDVKTVLQNWLKQPESNLGIEIKALDENGHDLAV 240

[NP_062024.1](https://www.ncbi.nlm.nih.gov/protein/NP_062024.1?report=genbank&log$=protalign&blast_rank=2&RID=0) 161 LRAVKTPTTVFVQILRLIKPMKDGTRYTGIRSLKLDMSPGTGIWQSIDVKTVLQNWLKQPESNLGIEIKALDENGHDLAV 240

[NP_001001525.1](https://www.ncbi.nlm.nih.gov/protein/NP_001001525.1?report=genbank&log$=protalign&blast_rank=3&RID=0) 160 LRPVKTPATVFVQILRLIKPMKDGTRYTGIRSLKLDMNPGTGIWQSIDVKTVLQNWLKQPESNLGIEIKALDENGHDLAV 239

[NP_999600.2](https://www.ncbi.nlm.nih.gov/protein/NP_999600.2?report=genbank&log$=protalign&blast_rank=4&RID=0) 160 LRPVKTPTTVFVQILRLIKPMKDGTRYTGIRSLKLDMNPGTGIWQSIDVKTVLQNWLKQPESNLGIEIKALDENGHDLAV 239

[NP_001002959.1](https://www.ncbi.nlm.nih.gov/protein/NP_001002959.1?report=genbank&log$=protalign&blast_rank=5&RID=0) 160 LRPVKTPTTVFVQILRLIKPMKDGTRYTGIRSLKLDMNPGTGIWQSIDVKTVLQNWLKQPESNLGIEIKALDENGHDLAV 239

[NP_001075286.1](https://www.ncbi.nlm.nih.gov/protein/NP_001075286.1?report=genbank&log$=protalign&blast_rank=6&RID=0) 160 LRPVKTPTTVFVQILRLIKPMKDGTRYTGIRSLKLDMNPGAGIWQSIDVKTVLQNWLKQPESNLGIEIKALDENGHDLAV 239

[NP_001009428.1](https://www.ncbi.nlm.nih.gov/protein/NP_001009428.1?report=genbank&log$=protalign&blast_rank=7&RID=0) 160 LRPVKTPTTVFVQILRLIKPMKDGTRYTGIRSLKLDMNPGTGIWQSIDVKTVLQNWLKQPESNLGIEIKALDENGHDLAV 239

[NP_001073388.1](https://www.ncbi.nlm.nih.gov/protein/NP_001073388.1?report=genbank&log$=protalign&blast_rank=8&RID=0) 160 LRPVETPTTVFVQILRLIKPMKDGTRYTGIRSLKLDMNPGTGIWQSIDVKTVLQNWLKQPESNLGIEIKALDENGHDLAV 239

[NP_001073588.1](https://www.ncbi.nlm.nih.gov/protein/NP_001073588.1?report=genbank&log$=protalign&blast_rank=9&RID=0) 160 LRPVETPTTVFVQILRLIKPMKDGTRYTGIRSLKLDMNPGTGIWQSIDVKTVLQNWLKQPESNLGIEIKALDENGHDLAV 239

[XP_003991021.1](https://www.ncbi.nlm.nih.gov/protein/XP_003991021.1?report=genbank&log$=protalign&blast_rank=10&RID=0) 160 LRPVKTPTTVFVQILRLIKPMKDGTRYTGIRSLKLDMNPGTGIWQSIDVKTVLQNWLKQPESNLGIEIKALDENGHDLAV 239

[NP_001272666.1](https://www.ncbi.nlm.nih.gov/protein/NP_001272666.1?report=genbank&log$=protalign&blast_rank=11&RID=0) 160 LRPVKTPTTVFVQILRLIKPMKDGTRYTGIRSLKLDMNPGTGIWQSIDVKTVLQNWLKQPESNLGIEIKALDENGHDLAV 239

[XP_018877573.1](https://www.ncbi.nlm.nih.gov/protein/XP_018877573.1?report=genbank&log$=protalign&blast_rank=12&RID=0) 160 LRPVETPTTVFVQILRLIKPMKDGTRYTGIRSLKLDMNPGTGIWQSIDVKTVLQNWLKQPESNLGIEIKALDENGHDLAV 239

[XP_002812720.1](https://www.ncbi.nlm.nih.gov/protein/XP_002812720.1?report=genbank&log$=protalign&blast_rank=13&RID=0) 160 LRPVETPTTVFVQILRLIKPMKDGTRYTGIRSLKLDMNPGTGIWQSIDVKTVLQNWLKQPESNLGIEIKALDENGHDLAV 239

[XP_032609257.1](https://www.ncbi.nlm.nih.gov/protein/XP_032609257.1?report=genbank&log$=protalign&blast_rank=14&RID=0) 160 LRPVETPTTVFVQILRLIKPMKDGTRYTGIRSLKLDMNPGTGIWQSIDVKTVLQNWLKQPESNLGIEIKALDENGHDLAV 239

[NP_005250.1](https://www.ncbi.nlm.nih.gov/protein/NP_005250.1?report=genbank&log$=protalign&blast_rank=0&RID=0) 240 TFPGPGEDGLNPFLEVKVTDTPKRSRRDFGLDCDEHSTESRCCRYPLTVDFEAFGWDWIIAPKRYKANYCSGECEFVFLQ 319

[NP_034964.1](https://www.ncbi.nlm.nih.gov/protein/NP_034964.1?report=genbank&log$=protalign&blast_rank=1&RID=0) 241 TFPGPGEDGLNPFLEVKVTDTPKRSRRDFGLDCDEHSTESRCCRYPLTVDFEAFGWDWIIAPKRYKANYCSGECEFVFLQ 320

[NP_062024.1](https://www.ncbi.nlm.nih.gov/protein/NP_062024.1?report=genbank&log$=protalign&blast_rank=2&RID=0) 241 TFPGPGEDGLNPFLEVKVTDTPKRSRRDFGLDCDEHSTESRCCRYPLTVDFEAFGWDWIIAPKRYKANYCSGECEFVFLQ 320

[NP_001001525.1](https://www.ncbi.nlm.nih.gov/protein/NP_001001525.1?report=genbank&log$=protalign&blast_rank=3&RID=0) 240 TFPEPGEDGLTPFLEVKVTDTPKRSRRDFGLDCDEHSTESRCCRYPLTVDFEAFGWDWIIAPKRYKANYCSGECEFVFLQ 319

[NP_999600.2](https://www.ncbi.nlm.nih.gov/protein/NP_999600.2?report=genbank&log$=protalign&blast_rank=4&RID=0) 240 TFPGPGEDGLNPFLEVKVTDTPKRSRRDFGLDCDEHSTESRCCRYPLTVDFEAFGWDWIIAPKRYKASYCSGECEFVFLQ 319

[NP_001002959.1](https://www.ncbi.nlm.nih.gov/protein/NP_001002959.1?report=genbank&log$=protalign&blast_rank=5&RID=0) 240 TFPGPGEDGLNPFLEVKVTDTPKRSRRDFGLDCDEHSTESRCCRYPLTVDFEAFGWDWIIAPKRYKANYCSGECEFVFLQ 319

[NP_001075286.1](https://www.ncbi.nlm.nih.gov/protein/NP_001075286.1?report=genbank&log$=protalign&blast_rank=6&RID=0) 240 TFPRPGEDGLNPFLEVKVTDTPKRSRRDFGLDCDEHSTESRCCRYPLTVDFEAFGWDWIIAPKRYKANYCSGECEFVFLQ 319

[NP_001009428.1](https://www.ncbi.nlm.nih.gov/protein/NP_001009428.1?report=genbank&log$=protalign&blast_rank=7&RID=0) 240 TFPEPGEEGLNPFLEVKVTDTPKRSRRDFGLDCDEHSTESRCCRYPLTVDFEAFGWDWIIAPKRYKANYCSGECEFLFLQ 319

[NP_001073388.1](https://www.ncbi.nlm.nih.gov/protein/NP_001073388.1?report=genbank&log$=protalign&blast_rank=8&RID=0) 240 TFPGPGEDGLNPFLEVKVTDTPKRSRRDFGLDCDEHSTESRCCRYPLTVDFEAFGWDWIIAPKRYKANYCSGECEFVFLQ 319

[NP_001073588.1](https://www.ncbi.nlm.nih.gov/protein/NP_001073588.1?report=genbank&log$=protalign&blast_rank=9&RID=0) 240 TFPGPGEDGLNPFLEVKVTDTPKRSRRDFGLDCDEHSTESRCCRYPLTVDFEAFGWDWIIAPKRYKANYCSGECEFVFLQ 319

[XP_003991021.1](https://www.ncbi.nlm.nih.gov/protein/XP_003991021.1?report=genbank&log$=protalign&blast_rank=10&RID=0) 240 TFPGPGEDGLNPFLEVKVTDTPKRSRRDFGLDCDEHSTESRCCRYPLTVDFEAFGWDWIIAPKRYKANYCSGECEFVFLQ 319

[NP_001272666.1](https://www.ncbi.nlm.nih.gov/protein/NP_001272666.1?report=genbank&log$=protalign&blast_rank=11&RID=0) 240 TFPEPGEEGLNPFLEVKVTDTPKRSRRDFGLDCDEHSTESRCCRYPLTVDFEAFGWDWIIAPKRYKANYCSGECEFLFLQ 319

[XP_018877573.1](https://www.ncbi.nlm.nih.gov/protein/XP_018877573.1?report=genbank&log$=protalign&blast_rank=12&RID=0) 240 TFPGPGEDGLNPFLEVKVTDTPKRSRRDFGLDCDEHSTESRCCRYPLTVDFEAFGWDWIIAPKRYKANYCSGECEFVFLQ 319

[XP_002812720.1](https://www.ncbi.nlm.nih.gov/protein/XP_002812720.1?report=genbank&log$=protalign&blast_rank=13&RID=0) 240 TFPGPGEDGLNPFLEVKVTDTPKRSRRDFGLDCDEHSTESRCCRYPLTVDFEAFGWDWIIAPKRYKANYCSGECEFVFLQ 319

[XP_032609257.1](https://www.ncbi.nlm.nih.gov/protein/XP_032609257.1?report=genbank&log$=protalign&blast_rank=14&RID=0) 240 TFPGPGEDGLNPFLEVKVTDTPKRSRRDFGLDCDEHSTESRCCRYPLTVDFEAFGWDWIIAPKRYKANYCSGECEFVFLQ 319

[NP_005250.1](https://www.ncbi.nlm.nih.gov/protein/NP_005250.1?report=genbank&log$=protalign&blast_rank=0&RID=0) 320 KYPHTHLVHQANPRGSAGPCCTPTKMSPINMLYFNGKEQIIYGKIPAMVVDRCGCS 375

[NP_034964.1](https://www.ncbi.nlm.nih.gov/protein/NP_034964.1?report=genbank&log$=protalign&blast_rank=1&RID=0) 321 KYPHTHLVHQANPRGSAGPCCTPTKMSPINMLYFNGKEQIIYGKIPAMVVDRCGCS 376

[NP_062024.1](https://www.ncbi.nlm.nih.gov/protein/NP_062024.1?report=genbank&log$=protalign&blast_rank=2&RID=0) 321 KYPHTHLVHQANPRGSAGPCCTPTKMSPINMLYFNGKEQIIYGKIPAMVVDRCGCS 376

[NP_001001525.1](https://www.ncbi.nlm.nih.gov/protein/NP_001001525.1?report=genbank&log$=protalign&blast_rank=3&RID=0) 320 KYPHTHLVHQANPRGSAGPCCTPTKMSPINMLYFNGEGQIIYGKIPAMVVDRCGCS 375

[NP_999600.2](https://www.ncbi.nlm.nih.gov/protein/NP_999600.2?report=genbank&log$=protalign&blast_rank=4&RID=0) 320 KYPHTHLVHQANPRGSAGPCCTPTKMSPINMLYFNGKEQIIYGKIPAMVVDRCGCS 375

[NP_001002959.1](https://www.ncbi.nlm.nih.gov/protein/NP_001002959.1?report=genbank&log$=protalign&blast_rank=5&RID=0) 320 KYPHTHLVHQANPRGSAGPCCTPTKMSPINMLYFNGKEQIIYGKIPAMVVDRCGCS 375

[NP_001075286.1](https://www.ncbi.nlm.nih.gov/protein/NP_001075286.1?report=genbank&log$=protalign&blast_rank=6&RID=0) 320 KYPHTHLVHQANPRGSAGPCCTPTKMSPINMLYFNGKEQIIYGKIPAMVVDRCGCS 375

[NP_001009428.1](https://www.ncbi.nlm.nih.gov/protein/NP_001009428.1?report=genbank&log$=protalign&blast_rank=7&RID=0) 320 KYPHTHLVHQANPKGSAGPCCTPTKMSPINMLYFNGKEQIIYGKIPGMVVDRCGCS 375

[NP_001073388.1](https://www.ncbi.nlm.nih.gov/protein/NP_001073388.1?report=genbank&log$=protalign&blast_rank=8&RID=0) 320 KYPHTHLVHQANPRGSAGPCCTPTKMSPINMLYFNGKEQIIYGKIPAMVVDRCGCS 375

[NP_001073588.1](https://www.ncbi.nlm.nih.gov/protein/NP_001073588.1?report=genbank&log$=protalign&blast_rank=9&RID=0) 320 KYPHTHLVHQANPRGSAGPCCTPTKMSPINMLYFNGKEQIIYGKIPAMVVDRCGCS 375

[XP_003991021.1](https://www.ncbi.nlm.nih.gov/protein/XP_003991021.1?report=genbank&log$=protalign&blast_rank=10&RID=0) 320 KYPHTHLVHQANPRGSAGPCCTPTKMSPINMLYFNGKEQIIYGKIPAMVVDRCGCS 375

[NP_001272666.1](https://www.ncbi.nlm.nih.gov/protein/NP_001272666.1?report=genbank&log$=protalign&blast_rank=11&RID=0) 320 KYPHTHLVHQANPKGSAGPCCTPTKMSPINMLYFNGKEQIIYGKIPGMVVDRCGCS 375

[XP_018877573.1](https://www.ncbi.nlm.nih.gov/protein/XP_018877573.1?report=genbank&log$=protalign&blast_rank=12&RID=0) 320 KYPHTHLVHQANPRGSAGPCCTPTKMSPINMLYFNGKEQIIYGKIPAMVVDRCGCS 375

[XP_002812720.1](https://www.ncbi.nlm.nih.gov/protein/XP_002812720.1?report=genbank&log$=protalign&blast_rank=13&RID=0) 320 KYPHTHLVHQANPRGSAGPCCTPTKMSPINMLYFNGKEQIIYGKIPAMVVDRCGCS 375

[XP_032609257.1](https://www.ncbi.nlm.nih.gov/protein/XP_032609257.1?report=genbank&log$=protalign&blast_rank=14&RID=0) 320 KYPHTHLVHQANPRGSAGPCCTPTKMSPINMLYFNGKEQIIYGKIPAMVVDRCGCS 375

GDF11

NP_005802.1 growth/differentiation factor 11 preproprotein [Homo sapiens]

NP_034402.1 growth/differentiation factor 11 preproprotein [Mus musculus]

NP_058899.1 growth/differentiation factor 11 precursor [Rattus norvegicus]

XP_002687467.2 growth/differentiation factor 11 [Bos taurus]

NP_001231239.1 growth/differentiation factor 11 precursor [Sus scrofa]

XP_038534852.1 growth/differentiation factor 11 [Canis lupus familiaris]

XP_023499520.1 growth/differentiation factor 11 [Equus caballus]

XP_012031460.3 growth/differentiation factor 11 [Ovis aries]

XP_016778746.2 growth/differentiation factor 11 [Pan troglodytes]

XP_015007410.2 growth/differentiation factor 11 [Macaca mulatta]

XP_003988905.1 growth/differentiation factor 11 [Felis catus]

XP_017903600.1 PREDICTED: growth/differentiation factor 11 [Capra hircus]

XP_018894140.2 growth/differentiation factor 11 [Gorilla gorilla gorilla]

XP_024112308.1 growth/differentiation factor 11 [Pongo abelii]

XP_032029354.1 growth/differentiation factor 11 [Hylobates moloch]

[NP_005802.1](https://www.ncbi.nlm.nih.gov/protein/NP_005802.1?report=genbank&log$=protalign&blast_rank=0&RID=0) 1 MVLAAPLLLGFLLLALELRPRGEAAEGP-AAAAAAAAAAAAa-GVGGERSSRPAPSVAPEPDGCPVCVWRQHSRELRLES 78

[NP_034402.1](https://www.ncbi.nlm.nih.gov/protein/NP_034402.1?report=genbank&log$=protalign&blast_rank=1&RID=0) 1 MVLAAPLLLGFLLLALELRPRGEAAEGP-AAAAAAAAAAA---GVGGERSSRPAPSAPPEPDGCPVCVWRQHSRELRLES 76

[NP_058899.1](https://www.ncbi.nlm.nih.gov/protein/NP_058899.1?report=genbank&log$=protalign&blast_rank=2&RID=0) 1 MVLAAPLLLGFLLLALELRPRGEAAEGP-AAAAAAAAAAA---GVGGERSSRPAPSAAPEPDGCPVCVWRQHSRELRLES 76

[XP_002687467.2](https://www.ncbi.nlm.nih.gov/protein/XP_002687467.2?report=genbank&log$=protalign&blast_rank=3&RID=0) 1 MVLAAPLLLGFLLLALELRPRGEAAEGP-AAAAAAAAAAAA--GAGGERSSRPAPSVAPEPDGCPVCVWRQHSRELRLES 77

[NP_001231239.1](https://www.ncbi.nlm.nih.gov/protein/NP_001231239.1?report=genbank&log$=protalign&blast_rank=4&RID=0) 1 MVLAAPLLLGFLLLALELRPRGEAAEGP-AAAAAAAAAA----GAGGERSSRPAASVAPEPDGCPVCLWRQHSRELRLES 75

[XP_038534852.1](https://www.ncbi.nlm.nih.gov/protein/XP_038534852.1?report=genbank&log$=protalign&blast_rank=5&RID=0) 1 MVLAAPLLLGFLLLALELRPRGEAAEGP-AAAAAAAAAAA---GAGGERSSRPAPSAAPEPDGCPVCVWRQHSRELRLES 76

[XP_023499520.1](https://www.ncbi.nlm.nih.gov/protein/XP_023499520.1?report=genbank&log$=protalign&blast_rank=6&RID=0) 1 MVLAAPLLLGFLLLALELRPRGEAAEGP-AAAAAAAAAA----GAGGERSSRPAPSVAPEPDGCPVCVWRQHSRELRLES 75

[XP_012031460.3](https://www.ncbi.nlm.nih.gov/protein/XP_012031460.3?report=genbank&log$=protalign&blast_rank=7&RID=0) 1 MVLAAPLLLGFLLLALELRPRGEAAEGP-AAAAAAAAAAAAa-GAGGERSSRPAPSVAPEPDGCPVCVWRQHSRELRLES 78

[XP_016778746.2](https://www.ncbi.nlm.nih.gov/protein/XP_016778746.2?report=genbank&log$=protalign&blast_rank=8&RID=0) 1 MVLAAPLLLGFLLLALELRPRGEAAEGP-AAAAAAAAAAA---GVGGERSSRPAPSVAPEPDGCPVCVWRQHSRELRLES 76

[XP_015007410.2](https://www.ncbi.nlm.nih.gov/protein/XP_015007410.2?report=genbank&log$=protalign&blast_rank=9&RID=0) 1 MVLAAPLLLGFLLLALELRPRGEAAEGP-AAAAAAAAAAAAaaGVGGERSSRPAPSVAPEPDGCPVCVWRQHSRELRLES 79

[XP_003988905.1](https://www.ncbi.nlm.nih.gov/protein/XP_003988905.1?report=genbank&log$=protalign&blast_rank=10&RID=0) 1 MVLAAPLLLGFLLLALELRPRGEAAEGP-AAAAAAAAAA----GAGGERSSRPAPSVAPEPDGCPVCVWRQHSRELRLES 75

[XP_017903600.1](https://www.ncbi.nlm.nih.gov/protein/XP_017903600.1?report=genbank&log$=protalign&blast_rank=11&RID=0) 1 MVLAAPLLLGFLLLALELRPRGEAAEGP-AAAAAAAAAAAA--GAGGERSSRPAPSVAPEPDGCPVCVWRQHSRELRLES 77

[XP_018894140.2](https://www.ncbi.nlm.nih.gov/protein/XP_018894140.2?report=genbank&log$=protalign&blast_rank=12&RID=0) 1 MVLAAPLLLGFLLLALELRPRGEAAEGP-AAAAAAAAAAAAa-GVGGERSSRPAPSVAPEPDGCPVCVWRQHSRELRLES 78

[XP_024112308.1](https://www.ncbi.nlm.nih.gov/protein/XP_024112308.1?report=genbank&log$=protalign&blast_rank=13&RID=0) 1 MVLAAPLLLGFLLLALELRPRGEAAEGPvAAAAAAAAAAAAaaGVGGERSSRPAPSVAPEPDGCPVCVWRQHSRELRLES 80

[XP_032029354.1](https://www.ncbi.nlm.nih.gov/protein/XP_032029354.1?report=genbank&log$=protalign&blast_rank=14&RID=0) 1 MVLAAPLLLGFLLLALELRPRGEAAEGP-AAAAAAAAAAAA--GVGGERSSRPAPSVAPEPDGCPVCVWRQHSRELRLES 77

[NP_005802.1](https://www.ncbi.nlm.nih.gov/protein/NP_005802.1?report=genbank&log$=protalign&blast_rank=0&RID=0) 79 IKSQILSKLRLKEAPNISREVVKQLLPKAPPLQQILDLHDFQGDALQPEDFLEEDEYHATTETVISMAQETDPAVQTDGS 158

[NP_034402.1](https://www.ncbi.nlm.nih.gov/protein/NP_034402.1?report=genbank&log$=protalign&blast_rank=1&RID=0) 77 IKSQILSKLRLKEAPNISREVVKQLLPKAPPLQQILDLHDFQGDALQPEDFLEEDEYHATTETVISMAQETDPAVQTDGS 156

[NP_058899.1](https://www.ncbi.nlm.nih.gov/protein/NP_058899.1?report=genbank&log$=protalign&blast_rank=2&RID=0) 77 IKSQILSKLRLKEAPNISREVVKQLLPKAPPLQQILDLHDFQGDALQPEDFLEEDEYHATTETVISMAQETDPAVQTDGS 156

[XP_002687467.2](https://www.ncbi.nlm.nih.gov/protein/XP_002687467.2?report=genbank&log$=protalign&blast_rank=3&RID=0) 78 IKSQILSKLRLKEAPNISREVVKQLLPKAPPLQQILDLHDFQGDALQPEDFLEEDEYHATTETVISMAQETDPAVQTDGS 157

[NP_001231239.1](https://www.ncbi.nlm.nih.gov/protein/NP_001231239.1?report=genbank&log$=protalign&blast_rank=4&RID=0) 76 VKSQILSKLRLKEAPNISREVVKQLLPKAPPLQQILDLHDFQGDALQPEDFLEEDEYHATTETVISMAQETDPAVQTDGS 155

[XP_038534852.1](https://www.ncbi.nlm.nih.gov/protein/XP_038534852.1?report=genbank&log$=protalign&blast_rank=5&RID=0) 77 IKSQILSKLRLKEAPNISREVVKQLLPKAPPLQQILDLHDFQGDALQPEDFLEEDEYHATTETVISMAQETDPAVQTDGS 156

[XP_023499520.1](https://www.ncbi.nlm.nih.gov/protein/XP_023499520.1?report=genbank&log$=protalign&blast_rank=6&RID=0) 76 IKSQILSKLRLKEAPNISREVVKQLLPKAPPLQQILDLHDFQGDALQPEDFLEEDEYHATTETVISMAQETDPAVQTDGS 155

[XP_012031460.3](https://www.ncbi.nlm.nih.gov/protein/XP_012031460.3?report=genbank&log$=protalign&blast_rank=7&RID=0) 79 IKSQILSKLRLKEAPNISREVVKQLLPKAPPLQQILDLHDFQGDALQPEDFLEEDEYHATTETVISMAQETDPAVQTDGS 158

[XP_016778746.2](https://www.ncbi.nlm.nih.gov/protein/XP_016778746.2?report=genbank&log$=protalign&blast_rank=8&RID=0) 77 IKSQILSKLRLKEAPNISREVVKQLLPKAPPLQQILDLHDFQGDALQPEDFLEEDEYHATTETVISMAQETDPAVQTDGS 156

[XP_015007410.2](https://www.ncbi.nlm.nih.gov/protein/XP_015007410.2?report=genbank&log$=protalign&blast_rank=9&RID=0) 80 IKSQILSKLRLKEAPNISREVVKQLLPKAPPLQQILDLHDFQGDALQPEDFLEEDEYHATTETVISMAQETDPAVQTDGS 159

[XP_003988905.1](https://www.ncbi.nlm.nih.gov/protein/XP_003988905.1?report=genbank&log$=protalign&blast_rank=10&RID=0) 76 IKSQILSKLRLKEAPNISREVVKQLLPKAPPLQQILDLHDFQGDALQPEDFLEEDEYHATTETVISMAQETDPAVQTDGS 155

[XP_017903600.1](https://www.ncbi.nlm.nih.gov/protein/XP_017903600.1?report=genbank&log$=protalign&blast_rank=11&RID=0) 78 IKSQILSKLRLKEAPNISREVVKQLLPKAPPLQQILDLHDFQGDALQPEDFLEEDEYHATTETVISMAQETDPAVQTDGS 157

[XP_018894140.2](https://www.ncbi.nlm.nih.gov/protein/XP_018894140.2?report=genbank&log$=protalign&blast_rank=12&RID=0) 79 IKSQILSKLRLKEAPNISREVVKQLLPKAPPLQQILDLHDFQGDALQPEDFLEEDEYHATTETVISMAQETDPAVQTDGS 158

[XP_024112308.1](https://www.ncbi.nlm.nih.gov/protein/XP_024112308.1?report=genbank&log$=protalign&blast_rank=13&RID=0) 81 IKSQILSKLRLKEAPNISREVVKQLLPKAPPLQQILDLHDFQGDALQPEDFLEEDEYHATTETVISMAQETDPAVQTDGS 160

[XP_032029354.1](https://www.ncbi.nlm.nih.gov/protein/XP_032029354.1?report=genbank&log$=protalign&blast_rank=14&RID=0) 78 IKSQILSKLRLKEAPNISREVVKQLLPKAPPLQQILDLHDFQGDALQPEDFLEEDEYHATTETVISMAQETDPAVQTDGS 157

[NP_005802.1](https://www.ncbi.nlm.nih.gov/protein/NP_005802.1?report=genbank&log$=protalign&blast_rank=0&RID=0) 159 PLCCHFHFSPKVMFTKVLKAQLWVYLRPVPRPATVYLQILRLKPLTGEGTAGGGGGGRRHIRIRSLKIELHSRSGHWQSI 238

[NP_034402.1](https://www.ncbi.nlm.nih.gov/protein/NP_034402.1?report=genbank&log$=protalign&blast_rank=1&RID=0) 157 PLCCHFHFSPKVMFTKVLKAQLWVYLRPVPRPATVYLQILRLKPLTGEGTAGGGGGGRRHIRIRSLKIELHSRSGHWQSI 236

[NP_058899.1](https://www.ncbi.nlm.nih.gov/protein/NP_058899.1?report=genbank&log$=protalign&blast_rank=2&RID=0) 157 PLCCHFHFSPKVMFTKVLKAQLWVYLRPVPRPATVYLQILRLKPLTGEGTAGGGGGGRRHIRIRSLKIELHSRSGHWQSI 236

[XP_002687467.2](https://www.ncbi.nlm.nih.gov/protein/XP_002687467.2?report=genbank&log$=protalign&blast_rank=3&RID=0) 158 PLCCHFHFSPKVMFTKVLKAQLWVYLRPVPRPATVYLQILRLKPLTGEGTAGGGGGGRRHIRIRSLKIDLHSRSGHWQSI 237

[NP_001231239.1](https://www.ncbi.nlm.nih.gov/protein/NP_001231239.1?report=genbank&log$=protalign&blast_rank=4&RID=0) 156 PLCCHFHFSPKVMFTKVLKAQLWVYLRPVPRPATVYLQILRLKPLTGEGTAGGGGGGRRHIRIRSLKIDLHSRSGHWQSI 235

[XP_038534852.1](https://www.ncbi.nlm.nih.gov/protein/XP_038534852.1?report=genbank&log$=protalign&blast_rank=5&RID=0) 157 PLCCHFHFSPKVMFTKVLKAQLWVYLRPVPRPATVYLQILRLKPLTGEGTAGGGGGGRRHIRIRSLKIELHSRSGHWQSI 236

[XP_023499520.1](https://www.ncbi.nlm.nih.gov/protein/XP_023499520.1?report=genbank&log$=protalign&blast_rank=6&RID=0) 156 PLCCHFHFSPKVMFTKVLKAQLWVYLRPVPRPATVYLQILRLKPLTGEGTAGGGGGGRRHIRIRSLKIELHSRSGHWQSI 235

[XP_012031460.3](https://www.ncbi.nlm.nih.gov/protein/XP_012031460.3?report=genbank&log$=protalign&blast_rank=7&RID=0) 159 PLCCHFHFSPKVMFTKVLKAQLWVYLRPVPRPATVYLQILRLKPLTGEGTAGGGGGGRRHIRIRSLKIDLHSRSGHWQSI 238

[XP_016778746.2](https://www.ncbi.nlm.nih.gov/protein/XP_016778746.2?report=genbank&log$=protalign&blast_rank=8&RID=0) 157 PLCCHFHFSPKVMFTKVLKAQLWVYLRPVPRPATVYLQILRLKPLTGEGTAGGGGGGRRHIRIRSLKIELHSRSGHWQSI 236

[XP_015007410.2](https://www.ncbi.nlm.nih.gov/protein/XP_015007410.2?report=genbank&log$=protalign&blast_rank=9&RID=0) 160 PLCCHFHFSPKVMFTKVLKAQLWVYLRPVPRPATVYLQILRLKPLTGEGTAGGGGGGRRHIRIRSLKIELHSRSGHWQSI 239

[XP_003988905.1](https://www.ncbi.nlm.nih.gov/protein/XP_003988905.1?report=genbank&log$=protalign&blast_rank=10&RID=0) 156 PLCCHFHFSPKVMFTKVLKAQLWVYLRPVPRPATVYLQILRLKPLTGEGTAGGGGGGRRHIRIRSLKIELHSRSGHWQSI 235

[XP_017903600.1](https://www.ncbi.nlm.nih.gov/protein/XP_017903600.1?report=genbank&log$=protalign&blast_rank=11&RID=0) 158 PLCCHFHFSPKVMFTKVLKAQLWVYLRPVPRPATVYLQILRLKPLTGEGTAGGGGGGRRHIRIRSLKIDLHSRSGHWQSI 237

[XP_018894140.2](https://www.ncbi.nlm.nih.gov/protein/XP_018894140.2?report=genbank&log$=protalign&blast_rank=12&RID=0) 159 PLCCHFHFSPKVMFTKVLKAQLWVYLRPVPRPATVYLQILRLKPLTGEGTAGGGGGGRRHIRIRSLKIELHSRSGHWQSI 238

[XP_024112308.1](https://www.ncbi.nlm.nih.gov/protein/XP_024112308.1?report=genbank&log$=protalign&blast_rank=13&RID=0) 161 PLCCHFHFSPKVMFTKVLKAQLWVYLRPVPRPATVYLQILRLKPLTGEGTAGGGGGGRRHIRIRSLKIELHSRSGHWQSI 240

[XP_032029354.1](https://www.ncbi.nlm.nih.gov/protein/XP_032029354.1?report=genbank&log$=protalign&blast_rank=14&RID=0) 158 PLCCHFHFSPKVMFTKVLKAQLWVYLRPVPRPATVYLQILRLKPLTGEGTAGGGGGGRRHIRIRSLKIELHSRSGHWQSI 237

[NP_005802.1](https://www.ncbi.nlm.nih.gov/protein/NP_005802.1?report=genbank&log$=protalign&blast_rank=0&RID=0) 239 DFKQVLHSWFRQPQSNWGIEINAFDPSGTDLAVTSLGPGAEGLHPFMELRVLENTKRSRRNLGLDCDEHSSESRCCRYPL 318

[NP_034402.1](https://www.ncbi.nlm.nih.gov/protein/NP_034402.1?report=genbank&log$=protalign&blast_rank=1&RID=0) 237 DFKQVLHSWFRQPQSNWGIEINAFDPSGTDLAVTSLGPGAEGLHPFMELRVLENTKRSRRNLGLDCDEHSSESRCCRYPL 316

[NP_058899.1](https://www.ncbi.nlm.nih.gov/protein/NP_058899.1?report=genbank&log$=protalign&blast_rank=2&RID=0) 237 DFKQVLHSWFRQPQSNWGIEINAFDPSGTDLAVTSLGPGAEGLHPFMELRVLENTKRSRRNLGLDCDEHSSESRCCRYPL 316

[XP_002687467.2](https://www.ncbi.nlm.nih.gov/protein/XP_002687467.2?report=genbank&log$=protalign&blast_rank=3&RID=0) 238 DFKQVLHSWFRQPQSNWGIEINAFDPSGTDLAVTSLGPGAEGLHPFMELRVLENTKRSRRNLGLDCDEHSSESRCCRYPL 317

[NP_001231239.1](https://www.ncbi.nlm.nih.gov/protein/NP_001231239.1?report=genbank&log$=protalign&blast_rank=4&RID=0) 236 DFKQVLHSWFRQPQSNWGIEINAFDPSGTDLAVTSLGPGAEGLHPFMELRVLENTKRSRRNLGLDCDEHSSESRCCRYPL 315

[XP_038534852.1](https://www.ncbi.nlm.nih.gov/protein/XP_038534852.1?report=genbank&log$=protalign&blast_rank=5&RID=0) 237 DFKQVLHSWFRQPQSNWGIEINAFDPSGTDLAVTSLGPGAEGLHPFMELRVLENTKRSRRNLGLDCDEHSSESRCCRYPL 316

[XP_023499520.1](https://www.ncbi.nlm.nih.gov/protein/XP_023499520.1?report=genbank&log$=protalign&blast_rank=6&RID=0) 236 DFKQVLHSWFRQPQSNWGIEINAFDPSGTDLAVTSLGPGAEGLHPFMELRVLENTKRSRRNLGLDCDEHSSESRCCRYPL 315

[XP_012031460.3](https://www.ncbi.nlm.nih.gov/protein/XP_012031460.3?report=genbank&log$=protalign&blast_rank=7&RID=0) 239 DFKQVLHSWFRQPQSNWGIEINAFDPSGTDLAVTSLGPGAEGLHPFMELRVLENTKRSRRNLGLDCDEHSSESRCCRYPL 318

[XP_016778746.2](https://www.ncbi.nlm.nih.gov/protein/XP_016778746.2?report=genbank&log$=protalign&blast_rank=8&RID=0) 237 DFKQVLHSWFRQPQSNWGIEINAFDPSGTDLAVTSLGPGAEGLHPFMELRVLENTKRSRRNLGLDCDEHSSESRCCRYPL 316

[XP_015007410.2](https://www.ncbi.nlm.nih.gov/protein/XP_015007410.2?report=genbank&log$=protalign&blast_rank=9&RID=0) 240 DFKQVLHSWFRQPQSNWGIEINAFDPSGTDLAVTSLGPGAEGLHPFMELRVLENTKRSRRNLGLDCDEHSSESRCCRYPL 319

[XP_003988905.1](https://www.ncbi.nlm.nih.gov/protein/XP_003988905.1?report=genbank&log$=protalign&blast_rank=10&RID=0) 236 DFKQVLHSWFRQPQSNWGIEINAFDPSGTDLAVTSLGPGAEGLHPFMELRVLENTKRSRRNLGLDCDEHSSESRCCRYPL 315

[XP_017903600.1](https://www.ncbi.nlm.nih.gov/protein/XP_017903600.1?report=genbank&log$=protalign&blast_rank=11&RID=0) 238 DFKQVLHSWFRQPQSNWGIEINAFDPSGTDLAVTSLGPGAEGLHPFMELRVLENTKRSRRNLGLDCDEHSSESRCCRYPL 317

[XP_018894140.2](https://www.ncbi.nlm.nih.gov/protein/XP_018894140.2?report=genbank&log$=protalign&blast_rank=12&RID=0) 239 DFKQVLHSWFRQPQSNWGIEINAFDPSGTDLAVTSLGPGAEGLHPFMELRVLENTKRSRRNLGLDCDEHSSESRCCRYPL 318

[XP_024112308.1](https://www.ncbi.nlm.nih.gov/protein/XP_024112308.1?report=genbank&log$=protalign&blast_rank=13&RID=0) 241 DFKQVLHSWFRQPQSNWGIEINAFDPSGTDLAVTSLGPGAEGLHPFMELRVLENTKRSRRNLGLDCDEHSSESRCCRYPL 320

[XP_032029354.1](https://www.ncbi.nlm.nih.gov/protein/XP_032029354.1?report=genbank&log$=protalign&blast_rank=14&RID=0) 238 DFKQVLHSWFRQPQSNWGIEINAFDPSGTDLAVTSLGPGAEGLHPFMELRVLENTKRSRRNLGLDCDEHSSESRCCRYPL 317

[NP_005802.1](https://www.ncbi.nlm.nih.gov/protein/NP_005802.1?report=genbank&log$=protalign&blast_rank=0&RID=0) 319 TVDFEAFGWDWIIAPKRYKANYCSGQCEYMFMQKYPHTHLVQQANPRGSAGPCCTPTKMSPINMLYFNDKQQIIYGKIPG 398

[NP_034402.1](https://www.ncbi.nlm.nih.gov/protein/NP_034402.1?report=genbank&log$=protalign&blast_rank=1&RID=0) 317 TVDFEAFGWDWIIAPKRYKANYCSGQCEYMFMQKYPHTHLVQQANPRGSAGPCCTPTKMSPINMLYFNDKQQIIYGKIPG 396

[NP_058899.1](https://www.ncbi.nlm.nih.gov/protein/NP_058899.1?report=genbank&log$=protalign&blast_rank=2&RID=0) 317 TVDFEAFGWDWIIAPKRYKANYCSGQCEYMFMQKYPHTHLVQQANPRGSAGPCCTPTKMSPINMLYFNDKQQIIYGKIPG 396

[XP_002687467.2](https://www.ncbi.nlm.nih.gov/protein/XP_002687467.2?report=genbank&log$=protalign&blast_rank=3&RID=0) 318 TVDFEAFGWDWIIAPKRYKANYCSGQCEYMFMQKYPHTHLVQQANPRGSAGPCCTPTKMSPINMLYFNDKQQIIYGKIPG 397

[NP_001231239.1](https://www.ncbi.nlm.nih.gov/protein/NP_001231239.1?report=genbank&log$=protalign&blast_rank=4&RID=0) 316 TVDFEAFGWDWIIAPKRYKANYCSGQCEYMFMQKYPHTHLVQQANPRGSAGPCCTPTKMSPINMLYFNDKQQIIYGKIPG 395

[XP_038534852.1](https://www.ncbi.nlm.nih.gov/protein/XP_038534852.1?report=genbank&log$=protalign&blast_rank=5&RID=0) 317 TVDFEAFGWDWIIAPKRYKANYCSGQCEYMFMQKYPHTHLVQQANPRGSAGPCCTPTKMSPINMLYFNDKQQIIYGKIPG 396

[XP_023499520.1](https://www.ncbi.nlm.nih.gov/protein/XP_023499520.1?report=genbank&log$=protalign&blast_rank=6&RID=0) 316 TVDFEAFGWDWIIAPKRYKANYCSGQCEYMFMQKYPHTHLVQQANPRGSAGPCCTPTKMSPINMLYFNDKQQIIYGKIPG 395

[XP_012031460.3](https://www.ncbi.nlm.nih.gov/protein/XP_012031460.3?report=genbank&log$=protalign&blast_rank=7&RID=0) 319 TVDFEAFGWDWIIAPKRYKANYCSGQCEYMFMQKYPHTHLVQQANPRGSAGPCCTPTKMSPINMLYFNDKQQIIYGKIPG 398

[XP_016778746.2](https://www.ncbi.nlm.nih.gov/protein/XP_016778746.2?report=genbank&log$=protalign&blast_rank=8&RID=0) 317 TVDFEAFGWDWIIAPKRYKANYCSGQCEYMFMQKYPHTHLVQQANPRGSAGPCCTPTKMSPINMLYFNDKQQIIYGKIPG 396

[XP_015007410.2](https://www.ncbi.nlm.nih.gov/protein/XP_015007410.2?report=genbank&log$=protalign&blast_rank=9&RID=0) 320 TVDFEAFGWDWIIAPKRYKANYCSGQCEYMFMQKYPHTHLVQQANPRGSAGPCCTPTKMSPINMLYFNDKQQIIYGKIPG 399

[XP_003988905.1](https://www.ncbi.nlm.nih.gov/protein/XP_003988905.1?report=genbank&log$=protalign&blast_rank=10&RID=0) 316 TVDFEAFGWDWIIAPKRYKANYCSGQCEYMFMQKYPHTHLVQQANPRGSAGPCCTPTKMSPINMLYFNDKQQIIYGKIPG 395

[XP_017903600.1](https://www.ncbi.nlm.nih.gov/protein/XP_017903600.1?report=genbank&log$=protalign&blast_rank=11&RID=0) 318 TVDFEAFGWDWIIAPKRYKANYCSGQCEYMFMQKYPHTHLVQQANPRGSAGPCCTPTKMSPINMLYFNDKQQIIYGKIPG 397

[XP_018894140.2](https://www.ncbi.nlm.nih.gov/protein/XP_018894140.2?report=genbank&log$=protalign&blast_rank=12&RID=0) 319 TVDFEAFGWDWIIAPKRYKANYCSGQCEYMFMQKYPHTHLVQQANPRGSAGPCCTPTKMSPINMLYFNDKQQIIYGKIPG 398

[XP_024112308.1](https://www.ncbi.nlm.nih.gov/protein/XP_024112308.1?report=genbank&log$=protalign&blast_rank=13&RID=0) 321 TVDFEAFGWDWIIAPKRYKANYCSGQCEYMFMQKYPHTHLVQQANPRGSAGPCCTPTKMSPINMLYFNDKQQIIYGKIPG 400

[XP_032029354.1](https://www.ncbi.nlm.nih.gov/protein/XP_032029354.1?report=genbank&log$=protalign&blast_rank=14&RID=0) 318 TVDFEAFGWDWIIAPKRYKANYCSGQCEYMFMQKYPHTHLVQQANPRGSAGPCCTPTKMSPINMLYFNDKQQIIYGKIPG 397

[NP_005802.1](https://www.ncbi.nlm.nih.gov/protein/NP_005802.1?report=genbank&log$=protalign&blast_rank=0&RID=0) 399 MVVDRCGCS 407

[NP_034402.1](https://www.ncbi.nlm.nih.gov/protein/NP_034402.1?report=genbank&log$=protalign&blast_rank=1&RID=0) 397 MVVDRCGCS 405

[NP_058899.1](https://www.ncbi.nlm.nih.gov/protein/NP_058899.1?report=genbank&log$=protalign&blast_rank=2&RID=0) 397 MVVDRCGCS 405

[XP_002687467.2](https://www.ncbi.nlm.nih.gov/protein/XP_002687467.2?report=genbank&log$=protalign&blast_rank=3&RID=0) 398 MVVDRCGCS 406

[NP_001231239.1](https://www.ncbi.nlm.nih.gov/protein/NP_001231239.1?report=genbank&log$=protalign&blast_rank=4&RID=0) 396 MVVDRCGCS 404

[XP_038534852.1](https://www.ncbi.nlm.nih.gov/protein/XP_038534852.1?report=genbank&log$=protalign&blast_rank=5&RID=0) 397 MVVDRCGCS 405

[XP_023499520.1](https://www.ncbi.nlm.nih.gov/protein/XP_023499520.1?report=genbank&log$=protalign&blast_rank=6&RID=0) 396 MVVDRCGCS 404

[XP_012031460.3](https://www.ncbi.nlm.nih.gov/protein/XP_012031460.3?report=genbank&log$=protalign&blast_rank=7&RID=0) 399 MVVDRCGCS 407

[XP_016778746.2](https://www.ncbi.nlm.nih.gov/protein/XP_016778746.2?report=genbank&log$=protalign&blast_rank=8&RID=0) 397 MVVDRCGCS 405

[XP_015007410.2](https://www.ncbi.nlm.nih.gov/protein/XP_015007410.2?report=genbank&log$=protalign&blast_rank=9&RID=0) 400 MVVDRCGCS 408

[XP_003988905.1](https://www.ncbi.nlm.nih.gov/protein/XP_003988905.1?report=genbank&log$=protalign&blast_rank=10&RID=0) 396 MVVDRCGCS 404

[XP_017903600.1](https://www.ncbi.nlm.nih.gov/protein/XP_017903600.1?report=genbank&log$=protalign&blast_rank=11&RID=0) 398 MVVDRCGCS 406

[XP_018894140.2](https://www.ncbi.nlm.nih.gov/protein/XP_018894140.2?report=genbank&log$=protalign&blast_rank=12&RID=0) 399 MVVDRCGCS 407

[XP_024112308.1](https://www.ncbi.nlm.nih.gov/protein/XP_024112308.1?report=genbank&log$=protalign&blast_rank=13&RID=0) 401 MVVDRCGCS 409

[XP_032029354.1](https://www.ncbi.nlm.nih.gov/protein/XP_032029354.1?report=genbank&log$=protalign&blast_rank=14&RID=0) 398 MVVDRCGCS 406

INHBC

NP_005529.1 inhibin beta C chain preproprotein [Homo sapiens]

NP_034695.1 inhibin beta C chain preproprotein [Mus musculus]

NP_072136.1 inhibin beta C chain precursor [Rattus norvegicus]

NP_001192912.1 inhibin beta C chain precursor [Bos taurus]

XP_003355541.3 inhibin beta C chain [Sus scrofa]

XP_849169.2 inhibin beta C chain [Canis lupus familiaris]

XP_001488633.1 inhibin beta C chain [Equus caballus]

XP_004006594.3 inhibin beta C chain [Ovis aries]

XP_522443.1 inhibin beta C chain [Pan troglodytes]

XP_001115940.1 inhibin beta C chain [Macaca mulatta]

XP_023112929.1 inhibin beta C chain [Felis catus]

XP_005680345.1 PREDICTED: inhibin beta C chain [Capra hircus]

XP_004053482.1 inhibin beta C chain [Gorilla gorilla gorilla]

XP_002823468.3 inhibin beta C chain [Pongo abelii]

XP_032028142.1 inhibin beta C chain [Hylobates moloch]

[NP_005529.1](https://www.ncbi.nlm.nih.gov/protein/NP_005529.1?report=genbank&log$=protalign&blast_rank=0&RID=0) 1 --MTSSLLLAFLLLAPTTVATPRAGGQCPACGGPTLELESQRELLLDLAKRSILDKLHLTQRPTLNRPVSRAALRTALQH 78

[NP_034695.1](https://www.ncbi.nlm.nih.gov/protein/NP_034695.1?report=genbank&log$=protalign&blast_rank=1&RID=0) 1 --MASSLLLALLFLTPTTVVNPKTEGPCPACWGAIFDLESQRELLLDLAKKSILDKLHLSQRPILSRPVSRGALKTALQR 78

[NP_072136.1](https://www.ncbi.nlm.nih.gov/protein/NP_072136.1?report=genbank&log$=protalign&blast_rank=2&RID=0) 1 --MASSLLLALLFLTLATVVNLKTDGPCPACWGATFDLESHRELLLDLAKKSILDKLHLSQRPILSRPVSREALKTALRR 78

[NP_001192912.1](https://www.ncbi.nlm.nih.gov/protein/NP_001192912.1?report=genbank&log$=protalign&blast_rank=3&RID=0) 1 --MICSLFLAFLVLAAAMVATPRADRQCPACGEPALDVESHRELLLNLAKRSILDKLHLSQRPTLGRPVSGVALRAALHR 78

[XP_003355541.3](https://www.ncbi.nlm.nih.gov/protein/XP_003355541.3?report=genbank&log$=protalign&blast_rank=4&RID=0) 1 maMISSLLLAFLFLAPATVATPQADSQCLACGGPTLDLESQRDLLLNLAKRSILDKLHLTQRPTLSRPVSRAALRTALQG 80

[XP_849169.2](https://www.ncbi.nlm.nih.gov/protein/XP_849169.2?report=genbank&log$=protalign&blast_rank=5&RID=0) 1 --MISPLLLAFLFLAPATVAIPRADSQCLACGGPAVDVERQRELLLDLAKRSILEKLHLSQRPTLSRPVSGAALRAALQR 78

[XP_001488633.1](https://www.ncbi.nlm.nih.gov/protein/XP_001488633.1?report=genbank&log$=protalign&blast_rank=6&RID=0) 1 --MISSMLLAFLFLAPATVATSRADGQCLACAGPTLDLESQRELLLDLAKRNILDKLHLSQRPTLSRPVSRAALRTVLQR 78

[XP_004006594.3](https://www.ncbi.nlm.nih.gov/protein/XP_004006594.3?report=genbank&log$=protalign&blast_rank=7&RID=0) 1 --MICSLFLAFLVLPAAMVATPRADRQCPACGEPALDVESHRELLLNLAKRSILDKLHLSQRPTLGRPVSRVALRAALQR 78

[XP_522443.1](https://www.ncbi.nlm.nih.gov/protein/XP_522443.1?report=genbank&log$=protalign&blast_rank=8&RID=0) 1 --MTSSLLLAFLLLAPTTVATPRSGGQCPACGGPTLELESQRELLLDLAKRSILDKLHLTQRPTLNRPVSRAALRTALQH 78

[XP_001115940.1](https://www.ncbi.nlm.nih.gov/protein/XP_001115940.1?report=genbank&log$=protalign&blast_rank=9&RID=0) 1 --MTSSLLLAFFLLAPTTVATPRAGGQCPACGGPTLELESQRELLLHLAKRSILDKLHLSQRPTLNRPVSRAALRTALQR 78

[XP_023112929.1](https://www.ncbi.nlm.nih.gov/protein/XP_023112929.1?report=genbank&log$=protalign&blast_rank=10&RID=0) 1 --MISSLLLAFLLLAPAPAAIPRADSQCLACGGPALDLDRQRELLLDLAKRSILDKLRLSQRPTLSRPVSRAALRTVLQH 78

[XP_005680345.1](https://www.ncbi.nlm.nih.gov/protein/XP_005680345.1?report=genbank&log$=protalign&blast_rank=11&RID=0) 1 --MICSLFLAFLVLPAAMVATPRADRQCPACGEPALDVESHRELLLNLAKRSILDKLHLSQRPTLGRPVSRVALRAALQR 78

[XP_004053482.1](https://www.ncbi.nlm.nih.gov/protein/XP_004053482.1?report=genbank&log$=protalign&blast_rank=12&RID=0) 1 --MTSSLLLAFLLLAPTTVATPRAGGQCPACGGPTLELESQRELLLDLAKRSILDKLHLTQRPTLNRPVSRAALRTALQH 78

[XP_002823468.3](https://www.ncbi.nlm.nih.gov/protein/XP_002823468.3?report=genbank&log$=protalign&blast_rank=13&RID=0) 1 --MTTSLLLAFLLLAPTTVATPRAGGQCPACGGPTLELESQRELLLDLAKRSILDKLHLTQRPTLNRPVSRAALRTALQR 78

[XP_032028142.1](https://www.ncbi.nlm.nih.gov/protein/XP_032028142.1?report=genbank&log$=protalign&blast_rank=14&RID=0) 1 --MTSSLLLAFLLLAPTTVATPRAGGQCPACGGPTLELESQRELLLDLAKRSILDKLHLTQRPTLNRPVSRAALRTALQR 78

[NP_005529.1](https://www.ncbi.nlm.nih.gov/protein/NP_005529.1?report=genbank&log$=protalign&blast_rank=0&RID=0) 79 LHGVPQGALLEDNREQE-CEIISFAETGLSTINQTRLDFHFSSDRTAGDREVQQASLMFFVQLPSNT-TWTLKVRVLVLG 156

[NP_034695.1](https://www.ncbi.nlm.nih.gov/protein/NP_034695.1?report=genbank&log$=protalign&blast_rank=1&RID=0) 79 LRGPRRETLLEHDQRQEeYEIISFADTDLSSINQTRLEFHF-SGRMASGMEVRQTRFMFFVQFPHNA-TQTMNIRVLVLR 156

[NP_072136.1](https://www.ncbi.nlm.nih.gov/protein/NP_072136.1?report=genbank&log$=protalign&blast_rank=2&RID=0) 79 LRGTRAETLLEHDQRQE-YEIISFADTGLSNINQTRLEFHF-SDRTTGGVEVLQTRFMFFMQLPPNT-TQTMNIRVLVLR 155

[NP_001192912.1](https://www.ncbi.nlm.nih.gov/protein/NP_001192912.1?report=genbank&log$=protalign&blast_rank=3&RID=0) 79 LHGPPQGALPEADEGQE-YEIISFAETGLCNTTQTRLDFHFSSDSSAGGLEVQQASLMFFVQLPPNT-TCPLKVRVLELS 156

[XP_003355541.3](https://www.ncbi.nlm.nih.gov/protein/XP_003355541.3?report=genbank&log$=protalign&blast_rank=4&RID=0) 81 LHGPPQGVLPEADRAQE-YEIISFAETGLSNIDQTRLDFHFPSDRTSGSLEVQQASLMFFVQLPPNA-TWTLKVRVLELG 158

[XP_849169.2](https://www.ncbi.nlm.nih.gov/protein/XP_849169.2?report=genbank&log$=protalign&blast_rank=5&RID=0) 79 LHGPPQGMLPEADGEQE-YEIISFADTGLSDINQTRLDFHFS-DRTASGMEIQQASLMFFVQIPPNT-TQTLKLKILVLS 155

[XP_001488633.1](https://www.ncbi.nlm.nih.gov/protein/XP_001488633.1?report=genbank&log$=protalign&blast_rank=6&RID=0) 79 LHGPPQGVLPEADRGQE-YEIISFAQTGLSNVNQTRLDFYF-SDRTAGGMEIQQASLMFFVQLPPNT-TQTMKVRILVPG 155

[XP_004006594.3](https://www.ncbi.nlm.nih.gov/protein/XP_004006594.3?report=genbank&log$=protalign&blast_rank=7&RID=0) 79 LHGPPQGVLPEADGGQE-YEIITFAETGLCNTTQTRLDFHFSSDSSAGGLEVQQASLMFFVQLPPNT-TCPLKVRVLELS 156

[XP_522443.1](https://www.ncbi.nlm.nih.gov/protein/XP_522443.1?report=genbank&log$=protalign&blast_rank=8&RID=0) 79 LHGVPQGALLEDNREQE-CEIISFAETGLSTINQTRLDFHFSSNRTAGDREVQQASLMFFVQLSSNT-TWTLKVRVLVLG 156

[XP_001115940.1](https://www.ncbi.nlm.nih.gov/protein/XP_001115940.1?report=genbank&log$=protalign&blast_rank=9&RID=0) 79 LHGVPQGALPEDNREQE-CEIISFAETGLSTINKTRLDFHFSSDRTAGDREVQQASLMFFVQLPSNT-TWTLKVRVLVLG 156

[XP_023112929.1](https://www.ncbi.nlm.nih.gov/protein/XP_023112929.1?report=genbank&log$=protalign&blast_rank=10&RID=0) 79 LHGPPQGTLLEADREQE-YEIISFADTGFSNINQTRLDFHFSSDRTASAMEIQQASVMFFVQLPPNTiTLPLKLRILVPG 157

[XP_005680345.1](https://www.ncbi.nlm.nih.gov/protein/XP_005680345.1?report=genbank&log$=protalign&blast_rank=11&RID=0) 79 LHGPPQGVLPEADGGQE-YEIITFAETGLCNTTQTRLDFHFSSDSSASGLEVQQASLMFFVQLPPNT-TCPLKVRVLELS 156

[XP_004053482.1](https://www.ncbi.nlm.nih.gov/protein/XP_004053482.1?report=genbank&log$=protalign&blast_rank=12&RID=0) 79 LHGVPQGALLEDNREQE-CEIISFAETGLSTINQTRLDFHFSSDRTAGDREVQQASLMFFVQLPSNT-TWTLKVRVLVLG 156

[XP_002823468.3](https://www.ncbi.nlm.nih.gov/protein/XP_002823468.3?report=genbank&log$=protalign&blast_rank=13&RID=0) 79 LHGVPQGALLEDNREQE-CEIISFAETGLSTINQTRLDFHFSSDRTAGDREVQQASLMFFVQLPSNT-TWTLKMRVLVLG 156

[XP_032028142.1](https://www.ncbi.nlm.nih.gov/protein/XP_032028142.1?report=genbank&log$=protalign&blast_rank=14&RID=0) 79 LRGVPQGALPEDNGEQE-CEIISFAETGLSTINQTRLDFHFSSDRTAGDREVQQASLVFFVQLPSNT-TWTLKVRVLVLG 156

[NP_005529.1](https://www.ncbi.nlm.nih.gov/protein/NP_005529.1?report=genbank&log$=protalign&blast_rank=0&RID=0) 157 PHNTNLTLATQYLLEVDASGWHQLPLGPEAQAACSQGHLTLELVLEGQVAQSSVILGGAAHRPFVAARVRVGGKHQIHRR 236

[NP_034695.1](https://www.ncbi.nlm.nih.gov/protein/NP_034695.1?report=genbank&log$=protalign&blast_rank=1&RID=0) 157 PYDTNLTLTSQYVVQVNASGWYQLLLGPEAQAACSQGHLTLELVPESQVAHSSLILGWFSHRPFVAAQVRVEGKHRVRRR 236

[NP_072136.1](https://www.ncbi.nlm.nih.gov/protein/NP_072136.1?report=genbank&log$=protalign&blast_rank=2&RID=0) 156 PYDTNLTLTSQYMLQVDASGWYQLLLGPEAQAACSQGHLTLELVPESQLAHSSLILDGVSHRPFVAAQVRVEGKHRVRRR 235

[NP_001192912.1](https://www.ncbi.nlm.nih.gov/protein/NP_001192912.1?report=genbank&log$=protalign&blast_rank=3&RID=0) 157 PRDTNLTSATQHLLQVDDTGWHQLLLGPEAQTAYSQGHLALELAPEEQVDWSPVVLARAAHRPFVTARVRVGGQHRVRRR 236

[XP_003355541.3](https://www.ncbi.nlm.nih.gov/protein/XP_003355541.3?report=genbank&log$=protalign&blast_rank=4&RID=0) 159 PHGTNLTLATQHPLEVNNSGWHQLLLGPEAEAAYSQGHLILELVPEGQVAWNSVILDGAAHRPFVTARVRVGGKHRVRRR 238

[XP_849169.2](https://www.ncbi.nlm.nih.gov/protein/XP_849169.2?report=genbank&log$=protalign&blast_rank=5&RID=0) 156 SRDTNLTSATQHLLDVDASGWHQLFLGREAQAACNQGHLTLELVPEGQGAWSSVILGGAAHRPFVSAKVKAGGKHRVHRR 235

[XP_001488633.1](https://www.ncbi.nlm.nih.gov/protein/XP_001488633.1?report=genbank&log$=protalign&blast_rank=6&RID=0) 156 PHDTNLTLATQHLLEVDASGWHQLLLGPEAQAACSQRHLTLELVPEGQVAQSSVILGGAAHRPFVAARVRVGGKHRVRRR 235

[XP_004006594.3](https://www.ncbi.nlm.nih.gov/protein/XP_004006594.3?report=genbank&log$=protalign&blast_rank=7&RID=0) 157 PRDTNLTSATQHLLEVNDSGWHQLLLGPEAQTAYSQGHLTLELAPEGQVAWSPVILAGAAHRPFVTARVRVGGKHRLRRR 236

[XP_522443.1](https://www.ncbi.nlm.nih.gov/protein/XP_522443.1?report=genbank&log$=protalign&blast_rank=8&RID=0) 157 PHNTNLTLATQYLLEVDASGWHQLLLGPEAQAACSQGHLTLELVLEGQVAQSSVILGGAAHRPFVAARVRVGGKHRIHRR 236

[XP_001115940.1](https://www.ncbi.nlm.nih.gov/protein/XP_001115940.1?report=genbank&log$=protalign&blast_rank=9&RID=0) 157 PHNTNLTLATQYLLEVDASGWHQLLLGPEAQAAYSQGHLTLELVPEGQVAQSSVILGGAAHRPFVAARVRVGGKHRIHRR 236

[XP_023112929.1](https://www.ncbi.nlm.nih.gov/protein/XP_023112929.1?report=genbank&log$=protalign&blast_rank=10&RID=0) 158 SHDTNLTWATQQLLEVDASGWHRLFLGPEAQAAFSQGHLTLELVLEGQGAQSSVILGGAAHRPFVAAKVKVGGKHRLHRR 237

[XP_005680345.1](https://www.ncbi.nlm.nih.gov/protein/XP_005680345.1?report=genbank&log$=protalign&blast_rank=11&RID=0) 157 PRDTNLTSATQHLLEVNDSGWHQLLLGPEAQTAYSQGHLALELAPEGQEAWSPVILAGAAHRPFVTARVRVGGKHRLRRR 236

[XP_004053482.1](https://www.ncbi.nlm.nih.gov/protein/XP_004053482.1?report=genbank&log$=protalign&blast_rank=12&RID=0) 157 PHNTNLTLATQYLLEVDASGWHQLLLGPEAQAACSQGHLTLELVLEGQVAQSSVILGGAAHRPFVAARVRVGGKHRIHRR 236

[XP_002823468.3](https://www.ncbi.nlm.nih.gov/protein/XP_002823468.3?report=genbank&log$=protalign&blast_rank=13&RID=0) 157 PHNTNLTLATQYLLEVDASGWHQLLLGPEAQAACSQGHLTLELVLEGQVAQSSVILGGAAHRPFVAARVRVGGKHRIHRR 236

[XP_032028142.1](https://www.ncbi.nlm.nih.gov/protein/XP_032028142.1?report=genbank&log$=protalign&blast_rank=14&RID=0) 157 PHNTNLSLATQYLLEVDASGWHQLLLGPEAQAACSQGHLTLELVLEGQVAQSSVILGGAAHRPFVAARVRVGGKHRIHRR 236

[NP_005529.1](https://www.ncbi.nlm.nih.gov/protein/NP_005529.1?report=genbank&log$=protalign&blast_rank=0&RID=0) 237 GIDCQGGSRMCCRQEFFVDFREIGWHDWIIQPEGYAMNFCIGQCPLHIAGMPGIAASFHTAVLNLLKANTAAGTTGGGSC 316

[NP_034695.1](https://www.ncbi.nlm.nih.gov/protein/NP_034695.1?report=genbank&log$=protalign&blast_rank=1&RID=0) 237 GIDCQGASRMCCRQEFFVDFREIGWNDWIIQPEGYAMNFCTGQCPLHVAGMPGISASFHTAVLNLLKANAAAGTTGRGSC 316

[NP_072136.1](https://www.ncbi.nlm.nih.gov/protein/NP_072136.1?report=genbank&log$=protalign&blast_rank=2&RID=0) 236 GINCQGLSRMCCRQEFFVDFREIGWHDWIIQPEGYAMNFCTGQCPLHVAGMPGISASFHTAVLNLLKANTDAGTARRGSC 315

[NP_001192912.1](https://www.ncbi.nlm.nih.gov/protein/NP_001192912.1?report=genbank&log$=protalign&blast_rank=3&RID=0) 237 GIDCQGRSKMCCRQEFFVDFREIGWHDWIIQPEGYAMNFCTGQCPLHVAGMPGIAASFYTSVLNLLKVNTAAGTTRGSSC 316

[XP_003355541.3](https://www.ncbi.nlm.nih.gov/protein/XP_003355541.3?report=genbank&log$=protalign&blast_rank=4&RID=0) 239 GIDCQGRSRMCCRQEFFVDFREIGWHDWIIQPEGYAMNFCTGHCPLHVAGVPGIAASFHTAVLNLLKANTAAGTTGGGSC 318

[XP_849169.2](https://www.ncbi.nlm.nih.gov/protein/XP_849169.2?report=genbank&log$=protalign&blast_rank=5&RID=0) 236 GINCQGGSRMCCRQEFFVDFRDIGWHDWIIQPEGYAMNFCTGQCPLHVAGMPGIAASFHTAVLNLLKANTAAGTAGGGSC 315

[XP_001488633.1](https://www.ncbi.nlm.nih.gov/protein/XP_001488633.1?report=genbank&log$=protalign&blast_rank=6&RID=0) 236 GINCQGGSRMCCRQEFFVDFREIGWHDWIIQPEGYAMNFCTGQCPLHVAGMPGIAASFHTTVFNLLKANTAAGATGGGSC 315

[XP_004006594.3](https://www.ncbi.nlm.nih.gov/protein/XP_004006594.3?report=genbank&log$=protalign&blast_rank=7&RID=0) 237 GIDCQGRSRMCCRQEFFVDFREIGWHDWIIQPEGYAMNFCTGQCPLHVAGMPGIAASFYTSVLNLLKVNTAAGTTRGSSC 316

[XP_522443.1](https://www.ncbi.nlm.nih.gov/protein/XP_522443.1?report=genbank&log$=protalign&blast_rank=8&RID=0) 237 GIDCQGGSRMCCRQEFFVDFREIGWHDWIIQPEGYAMNFCIGQCPLHIAGMPGIAASFHTAVLNLLKANTAAGTTGGGSC 316

[XP_001115940.1](https://www.ncbi.nlm.nih.gov/protein/XP_001115940.1?report=genbank&log$=protalign&blast_rank=9&RID=0) 237 GIDCQEGSRMCCRQEFFVDFREIGWHDWIIQPEGYAMNFCIGQCPLHVAGMPGIAASFHTAVLNLLKANTAAGTTGGGSC 316

[XP_023112929.1](https://www.ncbi.nlm.nih.gov/protein/XP_023112929.1?report=genbank&log$=protalign&blast_rank=10&RID=0) 238 GIDCQGGSRMCCRREFFVDFREIGWHDWIIQPEGYAMNFCTGQCPLHVAGMPGIAASFHTAVLNLLKANTAAGTAGGGSC 317

[XP_005680345.1](https://www.ncbi.nlm.nih.gov/protein/XP_005680345.1?report=genbank&log$=protalign&blast_rank=11&RID=0) 237 GIDCQGRSRMCCRQEFFVDFREIGWHDWIIQPEGYAMNFCTGQCPLHVAGMPGIAASFYTSVLNLLKVNTAAGTTRGSSC 316

[XP_004053482.1](https://www.ncbi.nlm.nih.gov/protein/XP_004053482.1?report=genbank&log$=protalign&blast_rank=12&RID=0) 237 GIDCQGGSRMCCRQEFFVDFREIGWHDWIIQPEGYAMNFCIGQCPLHIAGMPGIAASFHTAVLNLLKANTAAGTTGGGSC 316

[XP_002823468.3](https://www.ncbi.nlm.nih.gov/protein/XP_002823468.3?report=genbank&log$=protalign&blast_rank=13&RID=0) 237 GIDCQGGSRMCCRQEFFVDFREIGWHDWIIQPEGYAMNFCIGQCPLHIAGMPGIAASFHTAVLNLLKANTAAGTTGGGSC 316

[XP_032028142.1](https://www.ncbi.nlm.nih.gov/protein/XP_032028142.1?report=genbank&log$=protalign&blast_rank=14&RID=0) 237 GIDCQGGSRMCCRQEFFVDFREIGWHDWIIQPEGYAMNFCIGQCPLHIAGMPGIAASFHTAVLNLLKANTAAGTTGGGSC 316

[NP_005529.1](https://www.ncbi.nlm.nih.gov/protein/NP_005529.1?report=genbank&log$=protalign&blast_rank=0&RID=0) 317 CVPTARRPLSLLYYDRDSNIVKTDIPDMVVEACGCS 352

[NP_034695.1](https://www.ncbi.nlm.nih.gov/protein/NP_034695.1?report=genbank&log$=protalign&blast_rank=1&RID=0) 317 CVPTSRRPLSLLYYDRDSNIVKTDIPDMVVEACGCS 352

[NP_072136.1](https://www.ncbi.nlm.nih.gov/protein/NP_072136.1?report=genbank&log$=protalign&blast_rank=2&RID=0) 316 CVPTSRRPLSLLYYDRDSNIVKTDIPDMVVEACGCS 351

[NP_001192912.1](https://www.ncbi.nlm.nih.gov/protein/NP_001192912.1?report=genbank&log$=protalign&blast_rank=3&RID=0) 317 CVPTVRRPLSLLYYDRDSNIVKTDIPDMVVEACGCS 352

[XP_003355541.3](https://www.ncbi.nlm.nih.gov/protein/XP_003355541.3?report=genbank&log$=protalign&blast_rank=4&RID=0) 319 CVPTARRPLSLLYYDKDSNIVKTDIPDMVVEACGCS 354

[XP_849169.2](https://www.ncbi.nlm.nih.gov/protein/XP_849169.2?report=genbank&log$=protalign&blast_rank=5&RID=0) 316 CVPTARRPLSLLYYDRDSNVVKTDIPDMVVEACGCS 351

[XP_001488633.1](https://www.ncbi.nlm.nih.gov/protein/XP_001488633.1?report=genbank&log$=protalign&blast_rank=6&RID=0) 316 CVPTTRRPLSLLYYDRDSNIVKTDIPDMVVETCGCS 351

[XP_004006594.3](https://www.ncbi.nlm.nih.gov/protein/XP_004006594.3?report=genbank&log$=protalign&blast_rank=7&RID=0) 317 CVPTIRRPLSLLYYDRDSNIVKTDIPDMVVEACGCS 352

[XP_522443.1](https://www.ncbi.nlm.nih.gov/protein/XP_522443.1?report=genbank&log$=protalign&blast_rank=8&RID=0) 317 CVPTARRPLSLLYYDRDSNIVKTDIPDMVVEACGCS 352

[XP_001115940.1](https://www.ncbi.nlm.nih.gov/protein/XP_001115940.1?report=genbank&log$=protalign&blast_rank=9&RID=0) 317 CVPTARRPLSLLYYDRDSNIVKTDIPDMVVEACGCS 352

[XP_023112929.1](https://www.ncbi.nlm.nih.gov/protein/XP_023112929.1?report=genbank&log$=protalign&blast_rank=10&RID=0) 318 CVPTARRPLSLLYYDRDSNIVKTDIPDMVVEACGCS 353

[XP_005680345.1](https://www.ncbi.nlm.nih.gov/protein/XP_005680345.1?report=genbank&log$=protalign&blast_rank=11&RID=0) 317 CVPTIRRPLSLLYYDRDSNIVKTDIPDMVVEACGCS 352

[XP_004053482.1](https://www.ncbi.nlm.nih.gov/protein/XP_004053482.1?report=genbank&log$=protalign&blast_rank=12&RID=0) 317 CVPTARRPLSLLYYDRDSNIVKTDIPDMVVEACGCS 352

[XP_002823468.3](https://www.ncbi.nlm.nih.gov/protein/XP_002823468.3?report=genbank&log$=protalign&blast_rank=13&RID=0) 317 CVPTARRPLSLLYYDRDSNIVKTDIPDMVVEACGCS 352

[XP_032028142.1](https://www.ncbi.nlm.nih.gov/protein/XP_032028142.1?report=genbank&log$=protalign&blast_rank=14&RID=0) 317 CVPTARRPLSLLYYDRDSNIVKTDIPDMVVEACGCS 352

INHBE

NP_113667.1 inhibin beta E chain preproprotein [Homo sapiens]

NP_032408.2 inhibin beta E chain preproprotein [Mus musculus]

NP_114003.2 inhibin beta E chain precursor [Rattus norvegicus]

NP_001192771.1 inhibin beta E chain precursor [Bos taurus]

XP_003126368.2 LOW QUALITY PROTEIN: inhibin beta E chain [Sus scrofa]

XP_005625596.1 inhibin beta E chain isoform X1 [Canis lupus familiaris]

XP_001488840.1 inhibin beta E chain [Equus caballus]

XP_004006593.1 inhibin beta E chain [Ovis aries]

XP_509161.2 inhibin beta E chain [Pan troglodytes]

XP_001115958.2 inhibin beta E chain [Macaca mulatta]

XP_003988987.1 inhibin beta E chain [Felis catus]

XP_005680344.1 PREDICTED: inhibin beta E chain [Capra hircus]

XP_004053483.1 inhibin beta E chain [Gorilla gorilla gorilla]

XP_002823469.1 inhibin beta E chain [Pongo abelii]

XP_032029168.1 inhibin beta E chain [Hylobates moloch]

[NP_113667.1](https://www.ncbi.nlm.nih.gov/protein/NP_113667.1?report=genbank&log$=protalign&blast_rank=0&RID=0) 1 MRLPDVQLWLVLLWALVRAQGTGSVCPSCGGSKLAPQAERALVLELAKQQILDGLHLTSRPRITHPPPQAALTRAL 76

[NP_032408.2](https://www.ncbi.nlm.nih.gov/protein/NP_032408.2?report=genbank&log$=protalign&blast_rank=1&RID=0) 1 MKLPKAQLWLILLWALVWVQSTRSACPSCGGPTLAPQGERALVLELAKQQILEGLHLTSRPRITRPLPQAALTRAL 76

[NP_114003.2](https://www.ncbi.nlm.nih.gov/protein/NP_114003.2?report=genbank&log$=protalign&blast_rank=2&RID=0) 1 MGLSNVQLWTILLWALAWVQSTRSACPSCGAPTLTPQGERALVLELAKQQILEGLHLTSRPRITRPLPQAALTRAL 76

[NP_001192771.1](https://www.ncbi.nlm.nih.gov/protein/NP_001192771.1?report=genbank&log$=protalign&blast_rank=3&RID=0) 1 MGLLKVQLQLVLLWALVWAQVAGSACPSCGGPTLAPQAERALVLKLAKQQILEGLQLTSRPRLTHPPPKAVLTRAL 76

[XP_003126368.2](https://www.ncbi.nlm.nih.gov/protein/XP_003126368.2?report=genbank&log$=protalign&blast_rank=4&RID=0) 1 MGLPDVQLWLVLLWALVWAQGEGSVCPSCGGPTLAPQAERALVLELAKQQILQGLHLSSRPRITHPPPQAALTRAL 76

[XP_005625596.1](https://www.ncbi.nlm.nih.gov/protein/XP_005625596.1?report=genbank&log$=protalign&blast_rank=5&RID=0) 1 MGLPDVQPQLVLLWALVWAQGAGSGCPSCGGPTLAPQAERALVLELAKQQILEGLHLTSRPRITHPPPQAALARAL 76

[XP_001488840.1](https://www.ncbi.nlm.nih.gov/protein/XP_001488840.1?report=genbank&log$=protalign&blast_rank=6&RID=0) 1 MELPDVQLQLVLLWALVWAQGAGSVCPSCGGPTLAPQAERALVLELAKQQILEGLHLTSRPRITHPPSQAALTRAL 76

[XP_004006593.1](https://www.ncbi.nlm.nih.gov/protein/XP_004006593.1?report=genbank&log$=protalign&blast_rank=7&RID=0) 1 MGLHKVQLQLVLLWALVWAQVAGSACPSCGGPTLAPQAERALVLKLAKQQILEGLQLTSRPRMTHPPPPAVLTRAL 76

[XP_509161.2](https://www.ncbi.nlm.nih.gov/protein/XP_509161.2?report=genbank&log$=protalign&blast_rank=8&RID=0) 1 MRLPDVQLWLVLLWALVRAQGTGSVCPSCGGSKLAPQAERALVLELAKQQILDGLHLTSRPRITHPPPQAALTRAL 76

[XP_001115958.2](https://www.ncbi.nlm.nih.gov/protein/XP_001115958.2?report=genbank&log$=protalign&blast_rank=9&RID=0) 1 MGLPVVQLWLVLLWTLVRAQGTGSVCPSCGDSKLAPQAERALVLELAKQQILEGLHLTSRPRITHPPPQAALTRAL 76

[XP_003988987.1](https://www.ncbi.nlm.nih.gov/protein/XP_003988987.1?report=genbank&log$=protalign&blast_rank=10&RID=0) 1 MGLPDGQRQLVLLWALVWAQGAGSVCPSCGGPTLEPQAERALVLELAKQQILEGLHLTSRPRITHPPPQAALTRAL 76

[XP_005680344.1](https://www.ncbi.nlm.nih.gov/protein/XP_005680344.1?report=genbank&log$=protalign&blast_rank=11&RID=0) 1 [32]MGLHKVQLQLVLLWALVWAQVAGSACPSCGGPTLAPQAERALVLKLAKQQILEGLQLTSRPRMTHPPPQAVLTRAL 108

[XP_004053483.1](https://www.ncbi.nlm.nih.gov/protein/XP_004053483.1?report=genbank&log$=protalign&blast_rank=12&RID=0) 1 MQLPDVQLWLVLLWALVRAQGTGSVCPSCGGSKLAPQAERALVLELAKQQILDGLHLTSRPRITHPPPQAALTRAL 76

[XP_002823469.1](https://www.ncbi.nlm.nih.gov/protein/XP_002823469.1?report=genbank&log$=protalign&blast_rank=13&RID=0) 1 MGLPDVQLWLVLLWALVRAQGTGSVCPSCGGSKLAPQAERALVLELAKQQILDGLHLTSRPRITHPPPQAALTRAL 76

[XP_032029168.1](https://www.ncbi.nlm.nih.gov/protein/XP_032029168.1?report=genbank&log$=protalign&blast_rank=14&RID=0) 1 MGLPDVQLWLVLLWALVRAQGTGSVCPSCGGSKLTPQAERALVLELAKQQILDGLHLTSRPRITHPPPQAALTRAL 76

[NP_113667.1](https://www.ncbi.nlm.nih.gov/protein/NP_113667.1?report=genbank&log$=protalign&blast_rank=0&RID=0) 77 RRLQPGSVAPGNGEEVISFATVTD-STSAYSSLLTFHLSTPRSHHLYHARLWLHVLPTLPGTLCLRIFRWGPRRRRQGSR 155

[NP_032408.2](https://www.ncbi.nlm.nih.gov/protein/NP_032408.2?report=genbank&log$=protalign&blast_rank=1&RID=0) 77 RRLQPKSMVPGNREKVISFATIIDKSTSTYRSMLTFQLSPLWSHHLYHARLWLHVPPSFPGTLYLRIFRCGTTRCR-GFR 155

[NP_114003.2](https://www.ncbi.nlm.nih.gov/protein/NP_114003.2?report=genbank&log$=protalign&blast_rank=2&RID=0) 77 RRLQPRSMVPGNREKVISFATSIDKSTSTYRSVLTFQLSPLWSHHLYHARLWLHVPPSFPATLYLRIFGCGTTRCR-GSR 155

[NP_001192771.1](https://www.ncbi.nlm.nih.gov/protein/NP_001192771.1?report=genbank&log$=protalign&blast_rank=3&RID=0) 77 RRLQRGRVVPANGEQVISFAVLTDSSTATCSSTLTFHLSTPRSHHLYHARLWLQVLPTLPGPLSLRIFRWGGRRRGRGSR 156

[XP_003126368.2](https://www.ncbi.nlm.nih.gov/protein/XP_003126368.2?report=genbank&log$=protalign&blast_rank=4&RID=0) 77 RRLQRGSVGPAHGEEVISFAAITDSSTSTCGSTLTFHLSTPRSHHLYHARLWVHALPTLPGTLSLRIFRRGPRRRRRGSR 156

[XP_005625596.1](https://www.ncbi.nlm.nih.gov/protein/XP_005625596.1?report=genbank&log$=protalign&blast_rank=5&RID=0) 77 RRLQPRSMPIADGDEVISFATVAD-STSACSSVLTFDLSTAQPHQLSRARLWLHARPAPPGSLYLRVFGRRPGGGQRGAR 155

[XP_001488840.1](https://www.ncbi.nlm.nih.gov/protein/XP_001488840.1?report=genbank&log$=protalign&blast_rank=6&RID=0) 77 RRLQPGSAAPANGEEVISFATITDSSTSTCSSVLTFHLSAPRSHHLEHARLWLHALPTLPGALYLRIFQCGPRRRRQGSR 156

[XP_004006593.1](https://www.ncbi.nlm.nih.gov/protein/XP_004006593.1?report=genbank&log$=protalign&blast_rank=7&RID=0) 77 RRLQRGRVAPANGEQVISFAVLTDSSTATCSSTLTFHLSTPRSHHLYHARLWLHVLPTLPGPLSLRIFRWGGRRRGRGSR 156

[XP_509161.2](https://www.ncbi.nlm.nih.gov/protein/XP_509161.2?report=genbank&log$=protalign&blast_rank=8&RID=0) 77 RRLQPGSVAPGNGEEVISFATVTD-STSAYSSLLTFHLSTPRSHHLYHARLWLHVLPTLPGTLCLRIFRWGPRRRRQGSR 155

[XP_001115958.2](https://www.ncbi.nlm.nih.gov/protein/XP_001115958.2?report=genbank&log$=protalign&blast_rank=9&RID=0) 77 RRLQPGSVAPGNGEEVISFATVTD-STSAYSSLLTFHLSTPRFHHLYHARLWLHMLPTLPGTLCLRIFRWGPRRRHQRSR 155

[XP_003988987.1](https://www.ncbi.nlm.nih.gov/protein/XP_003988987.1?report=genbank&log$=protalign&blast_rank=10&RID=0) 77 RRLQPGSVAPVNGEEVISFATITEPSTSACSSVLTFHLSTAQSRHLYHARLWLRVLPTFPGTLSLRTFRWNPGRRRRESR 156

[XP_005680344.1](https://www.ncbi.nlm.nih.gov/protein/XP_005680344.1?report=genbank&log$=protalign&blast_rank=11&RID=0) 109 RRLQRGRAAPANGEQVISFAVLTDSSTATCSSTLTFHLSTPRSHHLYHARLWLHVLPTLPGPLSLRIFRWGGRRRGRGSR 188

[XP_004053483.1](https://www.ncbi.nlm.nih.gov/protein/XP_004053483.1?report=genbank&log$=protalign&blast_rank=12&RID=0) 77 RRLQPGSVAPGNGEEVISFATVTD-STSVHSSLLTFHLSTPRSHHLYHARLWLHVLPTLPGTLCLRIFRWGPRRRRQGSR 155

[XP_002823469.1](https://www.ncbi.nlm.nih.gov/protein/XP_002823469.1?report=genbank&log$=protalign&blast_rank=13&RID=0) 77 RRLQPGSVAPGNGEEVISFATVTD-STSAYSSLLTFHLSTPRSHHLYHARLWLHVLPTLPGTLCLRIFRWGPRRRRPGSR 155

[XP_032029168.1](https://www.ncbi.nlm.nih.gov/protein/XP_032029168.1?report=genbank&log$=protalign&blast_rank=14&RID=0) 77 RRLQPGSVASGNGEEVISFATVTD-STSAYSSLLTFHLSTPRSHHLYHARLWLHVLPTLPGTLCLRIFRWGPRRRRQGSR 155

[NP_113667.1](https://www.ncbi.nlm.nih.gov/protein/NP_113667.1?report=genbank&log$=protalign&blast_rank=0&RID=0) 156 TLLAEHHITNLGWHTLTLPSSGLRGEKSGVLKLQLDCRPLEG-NSTVTGQ-PRRLLDTAGHQQPFLELKIRANEPGAGRA 233

[NP_032408.2](https://www.ncbi.nlm.nih.gov/protein/NP_032408.2?report=genbank&log$=protalign&blast_rank=1&RID=0) 156 TFLAEHQTTSSGWHALTLPSSGLRSEDSGVVKLQLEFRPLDL-NSTAAGL-PRLLLDTAGQQRPFLELKIRANEPGAGRA 233

[NP_114003.2](https://www.ncbi.nlm.nih.gov/protein/NP_114003.2?report=genbank&log$=protalign&blast_rank=2&RID=0) 156 TFLADYQTTSSGWHALTLPSSGLRSEESGVTKLQLEFRPLDL-NSTTARL-PRLLLDTAGQQRPFLELKIRANEPGAGRA 233

[NP_001192771.1](https://www.ncbi.nlm.nih.gov/protein/NP_001192771.1?report=genbank&log$=protalign&blast_rank=3&RID=0) 157 VFLAEHQLTTPGWHALTLPSSGLKREESGVLKLRLDCSPLEG-NRTVTPQ----LLDTAGEQRPFLELKTRPKLPGAGRA 231

[XP_003126368.2](https://www.ncbi.nlm.nih.gov/protein/XP_003126368.2?report=genbank&log$=protalign&blast_rank=4&RID=0) 157 VLLAEHQMTTPGWHALTLPSSGLRREESGVLKLQLDCRALEG-NGTAALQ-PCQLLDTAGEQRPFLELKTRPKEPGAGRA 234

[XP_005625596.1](https://www.ncbi.nlm.nih.gov/protein/XP_005625596.1?report=genbank&log$=protalign&blast_rank=5&RID=0) 156 TLLAEQHLPAAGWHALALPSGGLRAEESAVLQLQLKCRLLPG-NRTSAQQlGRRLLDTAGDRRPFLQLQIWPREPGAGRA 234

[XP_001488840.1](https://www.ncbi.nlm.nih.gov/protein/XP_001488840.1?report=genbank&log$=protalign&blast_rank=6&RID=0) 157 ALLAEHQMKTPGWHALTLPSSGLRGEESGVLKLQLDCRPLED-NSTAARQ-PRQLLDMVGDRRPFLELKIRPSEPGAGRA 234

[XP_004006593.1](https://www.ncbi.nlm.nih.gov/protein/XP_004006593.1?report=genbank&log$=protalign&blast_rank=7&RID=0) 157 VFLAEHQLTTPGWHALTLPSSGLKREESGVLKLRLDCSSLEG-NRTVAPQ----LLDSAGEQRPFLELKTRPKWPGAARA 231

[XP_509161.2](https://www.ncbi.nlm.nih.gov/protein/XP_509161.2?report=genbank&log$=protalign&blast_rank=8&RID=0) 156 TLLAEHHITNLGWHALTLPSSGLRGEKSGVLKLQLDCRPLEG-NSTITGQ-PRRLLDTAGHQHPFLELKIRANEPGAGRA 233

[XP_001115958.2](https://www.ncbi.nlm.nih.gov/protein/XP_001115958.2?report=genbank&log$=protalign&blast_rank=9&RID=0) 156 TLLAEHHITNLGWHALTLPSSGLRGEKSGVLKLQLDCRPLEGnNSTVTGQ-PRRLLDTAGHQQPFLELKIRANEPGAGRA 234

[XP_003988987.1](https://www.ncbi.nlm.nih.gov/protein/XP_003988987.1?report=genbank&log$=protalign&blast_rank=10&RID=0) 157 TLLAEHQMTTPGWHALTLPSSGLRGEASAVLKLQLDCRLPGG-NATAA---PQWLVDTAGDERPFLELKIWPKGPGAGRT 232

[XP_005680344.1](https://www.ncbi.nlm.nih.gov/protein/XP_005680344.1?report=genbank&log$=protalign&blast_rank=11&RID=0) 189 VFLAEHQLTTPGWHALTLPSSGLKREESGVLKLRLDCSSLEG-NRTVAPQ----LLDSAGEQRPFLELKTRPKWPGAARA 263

[XP_004053483.1](https://www.ncbi.nlm.nih.gov/protein/XP_004053483.1?report=genbank&log$=protalign&blast_rank=12&RID=0) 156 TLLAEHHITNLGWHALTLPSSGLRAEKSGVLKLQLDCRPLEG-NSTVTGQ-PRQLLDTAGHQQPFLELKIQANEPGAGRA 233

[XP_002823469.1](https://www.ncbi.nlm.nih.gov/protein/XP_002823469.1?report=genbank&log$=protalign&blast_rank=13&RID=0) 156 TLLAEHHITNLGWHALTLPSSGLRGEKSGVLKLQLDCRPLEG-NSTVTGQ-PRRLVDTAGHQQPFLELKIRANEPGAGRA 233

[XP_032029168.1](https://www.ncbi.nlm.nih.gov/protein/XP_032029168.1?report=genbank&log$=protalign&blast_rank=14&RID=0) 156 TLLAEHHITNLGWHALTLPSSGLRGEKSGVLKLQLDCRPLEG-NSTVTGQ-PRRLLDTAGHQQPFLELKIRANEPGAGRA 233

[NP_113667.1](https://www.ncbi.nlm.nih.gov/protein/NP_113667.1?report=genbank&log$=protalign&blast_rank=0&RID=0) 234 RRRTPTCEPATPLCCRRDHYVDFQELGWRDWILQPEGYQLNYCSGQCPPHLAGSPGIAASFHSAVFSLLKANNPWPASTS 313

[NP_032408.2](https://www.ncbi.nlm.nih.gov/protein/NP_032408.2?report=genbank&log$=protalign&blast_rank=1&RID=0) 234 RRRTPTCEPETPLCCRRDHYVDFQELGWRDWILQPEGYQLNYCSGQCPPHLAGSPGIAASFHSAVFSLLKANNPWPAGSS 313

[NP_114003.2](https://www.ncbi.nlm.nih.gov/protein/NP_114003.2?report=genbank&log$=protalign&blast_rank=2&RID=0) 234 RRRTPTCESETPLCCRRDHYVDFQELGWRDWILQPEGYQLNYCSGQCPPHLAGSPGIAASFHSAVFSLLKANNPWPAGSS 313

[NP_001192771.1](https://www.ncbi.nlm.nih.gov/protein/NP_001192771.1?report=genbank&log$=protalign&blast_rank=3&RID=0) 232 RRRTPSCEPATPLCCRRDHYVDFQELGWRDWILQPEGYRLNYCSGQCPPHLAGSPGIAASFHSAVFSLLKANNPWPLGTS 311

[XP_003126368.2](https://www.ncbi.nlm.nih.gov/protein/XP_003126368.2?report=genbank&log$=protalign&blast_rank=4&RID=0) 235 RRRTPTCEPETPXCCRRDHYVDFQELGWRDWILQPEGYQLNYCSGQCPPHLAGSPGIAASFHSAVLSLLKANNPWPLGTS 314

[XP_005625596.1](https://www.ncbi.nlm.nih.gov/protein/XP_005625596.1?report=genbank&log$=protalign&blast_rank=5&RID=0) 235 RRRTPTCEPETPLCCRRDHYVDFQELGWRDWILQPEGYQLNYCSGQCPPHLAGSPGIAASFHSAVFSLLKANNPWPLGTS 314

[XP_001488840.1](https://www.ncbi.nlm.nih.gov/protein/XP_001488840.1?report=genbank&log$=protalign&blast_rank=6&RID=0) 235 RRRTPTCEPETPLCCRRDHYVDFQELGWRDWILQPEGYQLNYCSGQCPPHLAGSPGIAASFHSAVFSLLKANNPWPLGNS 314

[XP_004006593.1](https://www.ncbi.nlm.nih.gov/protein/XP_004006593.1?report=genbank&log$=protalign&blast_rank=7&RID=0) 232 RRRTPSCEPATPLCCRRDHYVDFQELGWRDWILQPEGYRLNFCSGQCPPHLAGSPGIAASFHSAVFSLLKANNPWPLGTS 311

[XP_509161.2](https://www.ncbi.nlm.nih.gov/protein/XP_509161.2?report=genbank&log$=protalign&blast_rank=8&RID=0) 234 RRRTPTCEPATPLCCRRDHYVDFQELGWRDWILQPEGYQLNYCSGQCPPHLAGSPGIAASFHSAVFSLLKANNPWPASTS 313

[XP_001115958.2](https://www.ncbi.nlm.nih.gov/protein/XP_001115958.2?report=genbank&log$=protalign&blast_rank=9&RID=0) 235 RRRTPTCEPATPLCCRRDHYVDFQELGWQDWILQPEGYQLNYCSGQCPPHLAGSPGIAASFHSAVFSLLKANNPWPASTS 314

[XP_003988987.1](https://www.ncbi.nlm.nih.gov/protein/XP_003988987.1?report=genbank&log$=protalign&blast_rank=10&RID=0) 233 RRRTPTCEPETPLCCRRDHYVDFRELGWRDWILQPEGYQLNYCSGQCPPHLAGSPGIAASFHSAVFSLLKANNPWPLGTS 312

[XP_005680344.1](https://www.ncbi.nlm.nih.gov/protein/XP_005680344.1?report=genbank&log$=protalign&blast_rank=11&RID=0) 264 RRRTPSCEPATPLCCRRDHYVDFQELGWRDWILQPEGYRLNFCSGQCPPHLAGSPGIAASFHSAVFSLLKANNPWPLGTS 343

[XP_004053483.1](https://www.ncbi.nlm.nih.gov/protein/XP_004053483.1?report=genbank&log$=protalign&blast_rank=12&RID=0) 234 RRRTPTCEPATPLCCRRDHYVDFQELGWRDWILQPEGYQLNYCSGQCPPHLAGSPGIAASFHSAVFSLLKANNPWPASTS 313

[XP_002823469.1](https://www.ncbi.nlm.nih.gov/protein/XP_002823469.1?report=genbank&log$=protalign&blast_rank=13&RID=0) 234 RRRTPTCEPATPLCCRRDHYVDFQELGWRDWILQPEGYQLNYCSGQCPPHLAGSPGIAASFHSAVFSLLKANNPWPASTS 313

[XP_032029168.1](https://www.ncbi.nlm.nih.gov/protein/XP_032029168.1?report=genbank&log$=protalign&blast_rank=14&RID=0) 234 RRRTPTCEPATPLCCRRDRYVDFQELGWQDWILQPEGYQLNYCSGQCPPHLAGSPGIAASFHSAVFSLLKANNPWPASTS 313

[NP_113667.1](https://www.ncbi.nlm.nih.gov/protein/NP_113667.1?report=genbank&log$=protalign&blast_rank=0&RID=0) 314 CCVPTARRPLSLLYLDHNGNVVKTDVPDMVVEACGCS 350

[NP_032408.2](https://www.ncbi.nlm.nih.gov/protein/NP_032408.2?report=genbank&log$=protalign&blast_rank=1&RID=0) 314 CCVPTARRPLSLLYLDHNGNVVKTDVPDMVVEACGCS 350

[NP_114003.2](https://www.ncbi.nlm.nih.gov/protein/NP_114003.2?report=genbank&log$=protalign&blast_rank=2&RID=0) 314 CCVPTARRPLSLLYLDHNGNVVKTDVPDMVVEACGCS 350

[NP_001192771.1](https://www.ncbi.nlm.nih.gov/protein/NP_001192771.1?report=genbank&log$=protalign&blast_rank=3&RID=0) 312 CCVPTARRPLSLLYLDRDGNVVKTDVPDMVVEACGCS 348

[XP_003126368.2](https://www.ncbi.nlm.nih.gov/protein/XP_003126368.2?report=genbank&log$=protalign&blast_rank=4&RID=0) 315 CCVPTARRSLSLLYLDRDGNVVKTDVPDMVVEACGCS 351

[XP_005625596.1](https://www.ncbi.nlm.nih.gov/protein/XP_005625596.1?report=genbank&log$=protalign&blast_rank=5&RID=0) 315 CCVPTARRPLSLLYLDRDGNVVKTDVPDMVVEACGCS 351

[XP_001488840.1](https://www.ncbi.nlm.nih.gov/protein/XP_001488840.1?report=genbank&log$=protalign&blast_rank=6&RID=0) 315 CCVPTARRPLSLLYLDRDGNVVKTDVPDMVVEACGCS 351

[XP_004006593.1](https://www.ncbi.nlm.nih.gov/protein/XP_004006593.1?report=genbank&log$=protalign&blast_rank=7&RID=0) 312 CCVPTARRPLSLLYLDRDGNVVKTDVPDMVVEACGCS 348

[XP_509161.2](https://www.ncbi.nlm.nih.gov/protein/XP_509161.2?report=genbank&log$=protalign&blast_rank=8&RID=0) 314 CCVPTARRPLSLLYLDHNGNVVKTDVPDMVVEACGCS 350

[XP_001115958.2](https://www.ncbi.nlm.nih.gov/protein/XP_001115958.2?report=genbank&log$=protalign&blast_rank=9&RID=0) 315 CCVPTARRPLSLLYLDHNGNVVKTDVPDMVVEACGCS 351

[XP_003988987.1](https://www.ncbi.nlm.nih.gov/protein/XP_003988987.1?report=genbank&log$=protalign&blast_rank=10&RID=0) 313 CCVPTARRPLSLLYLDRDGNVVKTDVPDMVVEACGCS 349

[XP_005680344.1](https://www.ncbi.nlm.nih.gov/protein/XP_005680344.1?report=genbank&log$=protalign&blast_rank=11&RID=0) 344 CCVPTARRPLSLLYLDRDGNVVKTDVPDMVVEACGCS 380

[XP_004053483.1](https://www.ncbi.nlm.nih.gov/protein/XP_004053483.1?report=genbank&log$=protalign&blast_rank=12&RID=0) 314 CCVPTARRPLSLLYLDHNGNVVKTDVPDMVVEACGCS 350

[XP_002823469.1](https://www.ncbi.nlm.nih.gov/protein/XP_002823469.1?report=genbank&log$=protalign&blast_rank=13&RID=0) 314 CCVPTARRPLSLLYLDHNGNVVKTDVPDMVVEACGCS 350

[XP_032029168.1](https://www.ncbi.nlm.nih.gov/protein/XP_032029168.1?report=genbank&log$=protalign&blast_rank=14&RID=0) 314 CCVPTARRPLSLLYLDHNGNVVKTDVPDMVVEACGCS 350

TGFB1

NP_000651.3 transforming growth factor beta-1 proprotein preproprotein [Homo sapiens]

NP_035707.1 transforming growth factor beta-1 proprotein preproprotein [Mus musculus]

NP_067589.1 transforming growth factor beta-1 proprotein precursor [Rattus norvegicus]

NP_001159540.1 transforming growth factor beta-1 proprotein precursor [Bos taurus]

XP_020949162.1 transforming growth factor beta-1 isoform X1 [Sus scrofa]

XP_038300925.1 transforming growth factor beta-1 proprotein isoform X1 [Canis lupus familiaris]

XP_005596143.1 transforming growth factor beta-1 isoform X1 [Equus caballus]

XP_027833173.1 transforming growth factor beta-1 proprotein isoform X3 [Ovis aries]

XP_009433930.1 transforming growth factor beta-1 isoform X2 [Pan troglodytes]

XP_028695614.1 transforming growth factor beta-1 proprotein isoform X1 [Macaca mulatta]

XP_006941294.1 transforming growth factor beta-1 proprotein isoform X1 [Felis catus]

XP_017917139.1 PREDICTED: transforming growth factor beta-1 isoform X1 [Capra hircus]

XP_018870688.1 transforming growth factor beta-1 proprotein isoform X1 [Gorilla gorilla gorilla]

XP_009230906.2 transforming growth factor beta-1 [Pongo abelii]

XP_032028718.1 transforming growth factor beta-1 proprotein [Hylobates moloch]

[NP_000651.3](https://www.ncbi.nlm.nih.gov/protein/NP_000651.3?report=genbank&log$=protalign&blast_rank=0&RID=0) 1 MPPSGLRLLPLLLPLLWLLVLTPGRPAAGLSTCKTIDMELVKRKRIEAIRGQILSKLRLASPPSQGEVPPGPLPEAVLAL 80

[NP_035707.1](https://www.ncbi.nlm.nih.gov/protein/NP_035707.1?report=genbank&log$=protalign&blast_rank=1&RID=0) 1 MPPSGLRLLPLLLPLPWLLVLTPGRPAAGLSTCKTIDMELVKRKRIEAIRGQILSKLRLASPPSQGEVPPGPLPEAVLAL 80

[NP_067589.1](https://www.ncbi.nlm.nih.gov/protein/NP_067589.1?report=genbank&log$=protalign&blast_rank=2&RID=0) 1 MPPSGLRLLPLLLPLPWLLVLTPGRPAAGLSTCKTIDMELVKRKRIEAIRGQILSKLRLASPPSQGEVPPGPLPEAVLAL 80

[NP_001159540.1](https://www.ncbi.nlm.nih.gov/protein/NP_001159540.1?report=genbank&log$=protalign&blast_rank=3&RID=0) 1 MPPSGLRLLPLLLPLLWLLMLTPGRPVAGLSTCKTIDMELVKRKRIEAIRGQILSKLRLASPPSQGDVPPGPLPEAILAL 80

[XP_020949162.1](https://www.ncbi.nlm.nih.gov/protein/XP_020949162.1?report=genbank&log$=protalign&blast_rank=4&RID=0) 1 MPPSGLRLLPLLLPLLWLLVLTPGRPAAGLSTCKTIDMELVKRKRIEAIRGQILSKLRLASPPSQGDVPPGPLPEAVLAL 80

[XP_038300925.1](https://www.ncbi.nlm.nih.gov/protein/XP_038300925.1?report=genbank&log$=protalign&blast_rank=5&RID=0) 1 MPPSGLRLLPLLLPLLRLLVLTPGRPAAGLSTCKTIDMELVKRKRIEAIRGQILSKLRLASPPSQGEVPPGPLPEAVLAL 80

[XP_005596143.1](https://www.ncbi.nlm.nih.gov/protein/XP_005596143.1?report=genbank&log$=protalign&blast_rank=6&RID=0) 1 MPPSGLRLLPLLLPLLWLLVLTPGRPAAGLSTCKTIDMELVKRKRIEAIRGQILSKLRLASPPSQGEVPPGPLPEAVLAL 80

[XP_027833173.1](https://www.ncbi.nlm.nih.gov/protein/XP_027833173.1?report=genbank&log$=protalign&blast_rank=7&RID=0) 1 MPPSGLRLLPLLLPLLWLLMLTPGRPVAGLSTCKTIDMELVKRKRIEAIRGQILSKLRLASPPSQGDVPPGPLPEAILAL 80

[XP_009433930.1](https://www.ncbi.nlm.nih.gov/protein/XP_009433930.1?report=genbank&log$=protalign&blast_rank=8&RID=0) 1 MPPSGLRLLPLLLPLLWLLVLTPGRPAAGLSTCKTIDMELVKRKRIEAIRGQILSKLRLASPPSQGEVPPGPLPEAVLAL 80

[XP_028695614.1](https://www.ncbi.nlm.nih.gov/protein/XP_028695614.1?report=genbank&log$=protalign&blast_rank=9&RID=0) 1 MPPSGLRLLPLLLPLLWLLVLTPGRPAAGLSTCKTIDMELVKRKRIEAIRGQILSKLRLASPPSQGEVPPGPLPEAVLAL 80

[XP_006941294.1](https://www.ncbi.nlm.nih.gov/protein/XP_006941294.1?report=genbank&log$=protalign&blast_rank=10&RID=0) 1 MPPSGLRLLPLLLPLLWLLVLTPGRPAAGLSTCKTIDMELVKRKRIEAIRGQILSKLRLASPPSQGEVPPGPLPEAVLAL 80

[XP_017917139.1](https://www.ncbi.nlm.nih.gov/protein/XP_017917139.1?report=genbank&log$=protalign&blast_rank=11&RID=0) 1 MPPSGLRLLPLLLPLLWLLMLTPGRPVAGLSTCKTIDMELVKRKRIEAIRGQILSKLRLASPPSQGDVPPGPLPEAILAL 80

[XP_018870688.1](https://www.ncbi.nlm.nih.gov/protein/XP_018870688.1?report=genbank&log$=protalign&blast_rank=12&RID=0) 1 MPPSGLRLLPLLLPLLWLLVLTPGRPAAGLSTCKTIDMELVKRKRIEAIRGQILSKLRLASPPSQGEVPPGPLPEAVLAL 80

[XP_009230906.2](https://www.ncbi.nlm.nih.gov/protein/XP_009230906.2?report=genbank&log$=protalign&blast_rank=13&RID=0) 1 MPPSGLRLLPLLLPLLWLLVLTPGRPAAGLSTCKTIDMELVKRKRIEAIRGQILSKLRLASPPSQGEVPPGPLPEAVLAL 80

[XP_032028718.1](https://www.ncbi.nlm.nih.gov/protein/XP_032028718.1?report=genbank&log$=protalign&blast_rank=14&RID=0) 1 MPPSGLRLLPLLLPLLWLLVLTLGRPAAGLSTCKTIDMELVKRKRIEAIRGQILSKLRLASPPSQGEVPPGPLPEAVLAL 80

[NP_000651.3](https://www.ncbi.nlm.nih.gov/protein/NP_000651.3?report=genbank&log$=protalign&blast_rank=0&RID=0) 81 YNSTRDRVAGESAEPEPEPEADYYAKEVTRVLMVETHNEIYDKFKQSTHSIYMFFNTSELREAVPEPVLLSRAELRLLRL 160

[NP_035707.1](https://www.ncbi.nlm.nih.gov/protein/NP_035707.1?report=genbank&log$=protalign&blast_rank=1&RID=0) 81 YNSTRDRVAGESADPEPEPEADYYAKEVTRVLMVDRNNAIYEKTKDISHSIYMFFNTSDIREAVPEPPLLSRAELRLQRL 160

[NP_067589.1](https://www.ncbi.nlm.nih.gov/protein/NP_067589.1?report=genbank&log$=protalign&blast_rank=2&RID=0) 81 YNSTRDRVAGESADPEPEPEADYYAKEVTRVLMVDRNNAIYDKTKDITHSIYMFFNTSDIREAVPEPPLLSRAELRLQRF 160

[NP_001159540.1](https://www.ncbi.nlm.nih.gov/protein/NP_001159540.1?report=genbank&log$=protalign&blast_rank=3&RID=0) 81 YNSTRDRVAGESAETEPEPEADYYAKEVTRVLMVEYGNKIYDKMKSSSHSIYMFFNTSELREAVPEPVLLSRADVRLLRL 160

[XP_020949162.1](https://www.ncbi.nlm.nih.gov/protein/XP_020949162.1?report=genbank&log$=protalign&blast_rank=4&RID=0) 81 YNSTRDRVAGESVEPEPEPEADYYAKEVTRVLMVESGNQIYDKFKGTPHSLYMLFNTSELREAVPEPVLLSRAELRLLRL 160

[XP_038300925.1](https://www.ncbi.nlm.nih.gov/protein/XP_038300925.1?report=genbank&log$=protalign&blast_rank=5&RID=0) 81 YNSTRDRVAGESAEPEPEPEADYYAKEVTRVLMVENTNKIYEKVKKSPHSIYMLFNTSELREAVPEPVLLSRAELRLLRL 160

[XP_005596143.1](https://www.ncbi.nlm.nih.gov/protein/XP_005596143.1?report=genbank&log$=protalign&blast_rank=6&RID=0) 81 YNSTRAQVAGESAETEPEPEADYYAKEVTRVLMVEKENEIYKTVETGSHSIYMFFNTSELRAAVPDPMLLSRAELRLLRL 160

[XP_027833173.1](https://www.ncbi.nlm.nih.gov/protein/XP_027833173.1?report=genbank&log$=protalign&blast_rank=7&RID=0) 81 YNSTRDRVAGESAETEPEPEADYYAKEVTRVLMVEYGNKIYDKMKSSSHSIYMFFNTSELREAVPEPVLLSRAELRLLRL 160

[XP_009433930.1](https://www.ncbi.nlm.nih.gov/protein/XP_009433930.1?report=genbank&log$=protalign&blast_rank=8&RID=0) 81 YNSTRDRVAGESAEPEPEPEADYYAKEVTRVLMVETHNEIYDKFKQSTHSIYMFFNTSELREAVPEPVLLSRAELRLLRL 160

[XP_028695614.1](https://www.ncbi.nlm.nih.gov/protein/XP_028695614.1?report=genbank&log$=protalign&blast_rank=9&RID=0) 81 YNSTRDRVAGESAEPEPEPEADYYAKEVTRVLMVETHNEIYDKFKQSTHSIYMFFNTSELREAVPEPVLLSRAELRLLRL 160

[XP_006941294.1](https://www.ncbi.nlm.nih.gov/protein/XP_006941294.1?report=genbank&log$=protalign&blast_rank=10&RID=0) 81 YNSTRDRVAGESAEPEPEPEADYYAKEVTRVLMVENTNKIYEKVQRTPHSIYMLFNTSELREAVPEPVLLSRAELRLLRL 160

[XP_017917139.1](https://www.ncbi.nlm.nih.gov/protein/XP_017917139.1?report=genbank&log$=protalign&blast_rank=11&RID=0) 81 YNSTRDRVAGESAETEPEPEADYYAKEVTRVLMVEYGNKIYDKMKSSSHSIYMFFNTSELREAVPEPVLLSRAELRLLRL 160

[XP_018870688.1](https://www.ncbi.nlm.nih.gov/protein/XP_018870688.1?report=genbank&log$=protalign&blast_rank=12&RID=0) 81 YNSTRDRVAGESAEPEPEPEADYYAKEVTRVLMVETHNEIYDKFKQSTHSIYMFFNTSELREAVPEPVLLSRAELRLLRL 160

[XP_009230906.2](https://www.ncbi.nlm.nih.gov/protein/XP_009230906.2?report=genbank&log$=protalign&blast_rank=13&RID=0) 81 YNSTRDRVAGESAEPEPEPEADYYAKEVTRVLMVETHNEIYDKFKQSTHSIYMFFNTSELREAVPEPVLLSRAELRLLRL 160

[XP_032028718.1](https://www.ncbi.nlm.nih.gov/protein/XP_032028718.1?report=genbank&log$=protalign&blast_rank=14&RID=0) 81 YNSTRDRVAGESAEPEPEPEADYYAKEVTRVLMVETHNEIYDKFKQSTHSIYMFFNTSELREAVPEPVLLSRAELRLLRL 160

[NP_000651.3](https://www.ncbi.nlm.nih.gov/protein/NP_000651.3?report=genbank&log$=protalign&blast_rank=0&RID=0) 161 KLKVEQHVELYQKYSNNSWRYLSNRLLAPSDSPEWLSFDVTGVVRQWLSRGGEIEGFRLSAHCSCDSRDNTLQVDIN-GF 239

[NP_035707.1](https://www.ncbi.nlm.nih.gov/protein/NP_035707.1?report=genbank&log$=protalign&blast_rank=1&RID=0) 161 KSSVEQHVELYQKYSNNSWRYLGNRLLTPTDTPEWLSFDVTGVVRQWLNQGDGIQGFRFSAHCSCDSKDNKLHVEIN-GI 239

[NP_067589.1](https://www.ncbi.nlm.nih.gov/protein/NP_067589.1?report=genbank&log$=protalign&blast_rank=2&RID=0) 161 KSTVEQHVELYQKYSNNSWRYLGNRLLTPTDTPEWLSFDVTGVVRQWLNQGDGIQGFRFSAHCSCDSKDNVLHVEIN-GI 239

[NP_001159540.1](https://www.ncbi.nlm.nih.gov/protein/NP_001159540.1?report=genbank&log$=protalign&blast_rank=3&RID=0) 161 KLKVEQHVELYQKYSNNSWRYLSNRLLAPSDSPEWLSFDVTGVVRQWLTRREEIEGFRLSAHCSCDSKDNTLQVDIN-GF 239

[XP_020949162.1](https://www.ncbi.nlm.nih.gov/protein/XP_020949162.1?report=genbank&log$=protalign&blast_rank=4&RID=0) 161 KLKVEQHVELYQKYSNDSWRYLSNRLLAPSDSPEWLSFDVTGVVRQWLTRREAIEGFRLSAHCSCDSKDNTLHVEINAGF 240

[XP_038300925.1](https://www.ncbi.nlm.nih.gov/protein/XP_038300925.1?report=genbank&log$=protalign&blast_rank=5&RID=0) 161 KLKAEQHVELYQKYSNDSWRYLSNRLLAPSDTPEWLSFDVTGVVRQWLSHGGEVEGFRLSAHCSCDSKDNTLQVDINAGF 240

[XP_005596143.1](https://www.ncbi.nlm.nih.gov/protein/XP_005596143.1?report=genbank&log$=protalign&blast_rank=6&RID=0) 161 KLSVEQHVELYQKYSNNSWRYLSNRLLTPSDSPEWLSFDVTGVVRQWLSQGGAMEGFRLSAHCSCDSKDNTLRVGINVGF 240

[XP_027833173.1](https://www.ncbi.nlm.nih.gov/protein/XP_027833173.1?report=genbank&log$=protalign&blast_rank=7&RID=0) 161 KLKVEQHVELYQKYSNNSWRYLSNRLLAPSDSPEWLSFDVTGVVRQWLTHREEIEGFRLSAHCSCDSKDNTLQVDINAGF 240

[XP_009433930.1](https://www.ncbi.nlm.nih.gov/protein/XP_009433930.1?report=genbank&log$=protalign&blast_rank=8&RID=0) 161 KLKVEQHVELYQKYSNNSWRYLSNRLLAPSDSPEWLSFDVTGVVRQWLSRGGEIEGFRLSAHCSCDSRDNTLQVDINAGF 240

[XP_028695614.1](https://www.ncbi.nlm.nih.gov/protein/XP_028695614.1?report=genbank&log$=protalign&blast_rank=9&RID=0) 161 KLKVEQHVELYQKYSNNSWRYLSNRLLAPSDSPEWLSFDVTGVVRQWLSRGGEIEGFRLSAHCSCDSKDNTLQVDINAGF 240

[XP_006941294.1](https://www.ncbi.nlm.nih.gov/protein/XP_006941294.1?report=genbank&log$=protalign&blast_rank=10&RID=0) 161 KLKAEQHVELYQKYSNNSWRYLSNRLLAPSDTPEWLSFDVTGVVRQWLSHGGEVEGFRLSAHCSCDSKDNTLQVDINAGF 240

[XP_017917139.1](https://www.ncbi.nlm.nih.gov/protein/XP_017917139.1?report=genbank&log$=protalign&blast_rank=11&RID=0) 161 KLKVEQHVELYQKYSNNSWRYLSNRLLAPSDSPEWLSFDVTGVVRQWLTHREEIEGFRLSAHCSCDSKDNTLQVDINAGF 240

[XP_018870688.1](https://www.ncbi.nlm.nih.gov/protein/XP_018870688.1?report=genbank&log$=protalign&blast_rank=12&RID=0) 161 KLKVEQHVELYQKYSNNSWRYLSNRLLAPSDSPEWLSFDVTGVVRQWLSRGGEIEGFRLSAHCSCDSRDNTLQVDINAGF 240

[XP_009230906.2](https://www.ncbi.nlm.nih.gov/protein/XP_009230906.2?report=genbank&log$=protalign&blast_rank=13&RID=0) 161 KLKVEQHVELYQKYSNNSWRYLSNRLLAPSDSPEWLSFDVTGVVRQWLSRGGEIEGFRLSAHCSCDSKDNTLQVDIN-GF 239

[XP_032028718.1](https://www.ncbi.nlm.nih.gov/protein/XP_032028718.1?report=genbank&log$=protalign&blast_rank=14&RID=0) 161 KLKVEQHVELYQKYSNNSWRYLSNRLLAPSDSPEWLSFDVTGVVRQWLSHGGEIEGFRLSAHCSCDSKDNTLQVDIN-GF 239

[NP_000651.3](https://www.ncbi.nlm.nih.gov/protein/NP_000651.3?report=genbank&log$=protalign&blast_rank=0&RID=0) 240 TTGRRGDLATIHGMNRPFLLLMATPLERAQHLQSSRHRRALDTNYCFSSTEKNCCVRQLYIDFRKDLGWKWIHEPKGYHA 319

[NP_035707.1](https://www.ncbi.nlm.nih.gov/protein/NP_035707.1?report=genbank&log$=protalign&blast_rank=1&RID=0) 240 SPKRRGDLGTIHDMNRPFLLLMATPLERAQHLHSSRHRRALDTNYCFSSTEKNCCVRQLYIDFRKDLGWKWIHEPKGYHA 319

[NP_067589.1](https://www.ncbi.nlm.nih.gov/protein/NP_067589.1?report=genbank&log$=protalign&blast_rank=2&RID=0) 240 SPKRRGDLGTIHDMNRPFLLLMATPLERAQHLHSSRHRRALDTNYCFSSTEKNCCVRQLYIDFRKDLGWKWIHEPKGYHA 319

[NP_001159540.1](https://www.ncbi.nlm.nih.gov/protein/NP_001159540.1?report=genbank&log$=protalign&blast_rank=3&RID=0) 240 SSGRRGDLATIHGMNRPFLLLMATPLERAQHLHSSRHRRALDTNYCFSSTEKNCCVRQLYIDFRKDLGWKWIHEPKGYHA 319

[XP_020949162.1](https://www.ncbi.nlm.nih.gov/protein/XP_020949162.1?report=genbank&log$=protalign&blast_rank=4&RID=0) 241 NSGRRGDLATIHGMNRPFLLLMATPLERAQHLHSSRHRRALDTNYCFSSTEKNCCVRQLYIDFRKDLGWKWIHEPKGYHA 320

[XP_038300925.1](https://www.ncbi.nlm.nih.gov/protein/XP_038300925.1?report=genbank&log$=protalign&blast_rank=5&RID=0) 241 SSSRRGDLATIHGMNRPFLLLMATPLERAQHLHSSRQRRALDTNYCFSSTEKNCCVRQLYIDFRKDLGWKWIHEPKGYHA 320

[XP_005596143.1](https://www.ncbi.nlm.nih.gov/protein/XP_005596143.1?report=genbank&log$=protalign&blast_rank=6&RID=0) 241 SSSRRGDLATIDGMNRPFLLLMATPLERAQQLHSSRHRRALDTNYCFSSTEKNCCVRQLYIDFRKDLGWKWIHEPKGYHA 320

[XP_027833173.1](https://www.ncbi.nlm.nih.gov/protein/XP_027833173.1?report=genbank&log$=protalign&blast_rank=7&RID=0) 241 SSGRRGDLATIHGMNRPFLLLMATPLERAQHLHSSRHRRALDTNYCFSSTEKNCCVRQLYIDFRKDLGWKWIHEPKGYHA 320

[XP_009433930.1](https://www.ncbi.nlm.nih.gov/protein/XP_009433930.1?report=genbank&log$=protalign&blast_rank=8&RID=0) 241 TTGRRGDLATIHGMNRPFLLLMATPLERAQHLQSSRHRRALDTNYCFSSTEKNCCVRQLYIDFRKDLGWKWIHEPKGYHA 320

[XP_028695614.1](https://www.ncbi.nlm.nih.gov/protein/XP_028695614.1?report=genbank&log$=protalign&blast_rank=9&RID=0) 241 TTGRRGDLATIHGMNRPFLLLMATPLERAQHLQSSRHRRALDTNYCFSSTEKNCCVRQLYIDFRKDLGWKWIHEPKGYHA 320

[XP_006941294.1](https://www.ncbi.nlm.nih.gov/protein/XP_006941294.1?report=genbank&log$=protalign&blast_rank=10&RID=0) 241 SSSRRGDLATIHGMNRPFLLLMATPLERAQHLHSSRHRRALDTNYCFSSTEKNCCVRQLYIDFRKDLGWKWIHEPKGYHA 320

[XP_017917139.1](https://www.ncbi.nlm.nih.gov/protein/XP_017917139.1?report=genbank&log$=protalign&blast_rank=11&RID=0) 241 SSGRRGDLATIHGMNRPFLLLMATPLERAQHLHSSRHRRALDTNYCFSSTEKNCCVRQLYIDFRKDLGWKWIHEPKGYHA 320

[XP_018870688.1](https://www.ncbi.nlm.nih.gov/protein/XP_018870688.1?report=genbank&log$=protalign&blast_rank=12&RID=0) 241 TTGRRGDLATIHGMNRPFLLLMATPLERAQHLQSSRHRRALDTNYCFSSTEKNCCVRQLYIDFRKDLGWKWIHEPKGYHA 320

[XP_009230906.2](https://www.ncbi.nlm.nih.gov/protein/XP_009230906.2?report=genbank&log$=protalign&blast_rank=13&RID=0) 240 TTGRRGDLATIHGMNRPFLLLMATPLERAQHLQSSRHRRALDTNYCFSSTEKNCCVRQLYIDFRKDLGWKWIHEPKGYHA 319

[XP_032028718.1](https://www.ncbi.nlm.nih.gov/protein/XP_032028718.1?report=genbank&log$=protalign&blast_rank=14&RID=0) 240 TTGRRGDLATIHGMNRPFLLLMATPLERAQHLQSSRHRRALDTNYCFSSTEKNCCVRQLYIDFRKDLGWKWIHEPKGYHA 319

[NP_000651.3](https://www.ncbi.nlm.nih.gov/protein/NP_000651.3?report=genbank&log$=protalign&blast_rank=0&RID=0) 320 NFCLGPCPYIWSLDTQYSKVLALYNQHNPGASAAPCCVPQALEPLPIVYYVGRKPKVEQLSNMIVRSCKCS 390

[NP_035707.1](https://www.ncbi.nlm.nih.gov/protein/NP_035707.1?report=genbank&log$=protalign&blast_rank=1&RID=0) 320 NFCLGPCPYIWSLDTQYSKVLALYNQHNPGASASPCCVPQALEPLPIVYYVGRKPKVEQLSNMIVRSCKCS 390

[NP_067589.1](https://www.ncbi.nlm.nih.gov/protein/NP_067589.1?report=genbank&log$=protalign&blast_rank=2&RID=0) 320 NFCLGPCPYIWSLDTQYSKVLALYNQHNPGASASPCCVPQALEPLPIVYYVGRKPKVEQLSNMIVRSCKCS 390

[NP_001159540.1](https://www.ncbi.nlm.nih.gov/protein/NP_001159540.1?report=genbank&log$=protalign&blast_rank=3&RID=0) 320 NFCLGPCPYIWSLDTQYSKVLALYNQHNPGASAAPCCVPQALEPLPIVYYVGRKPKVEQLSNMIVRSCKCS 390

[XP_020949162.1](https://www.ncbi.nlm.nih.gov/protein/XP_020949162.1?report=genbank&log$=protalign&blast_rank=4&RID=0) 321 NFCLGPCPYIWSLDTQYSKVLALYNQHNPGASAAPCCVPQALEPLPIVYYVGRKPKVEQLSNMIVRSCKCS 391

[XP_038300925.1](https://www.ncbi.nlm.nih.gov/protein/XP_038300925.1?report=genbank&log$=protalign&blast_rank=5&RID=0) 321 NFCLGPCPYIWSLDTQYSKVLALYNQHNPGASAAPCCVPQALEPLPIVYYVGRKPKVEQLSNMIVRSCKCS 391

[XP_005596143.1](https://www.ncbi.nlm.nih.gov/protein/XP_005596143.1?report=genbank&log$=protalign&blast_rank=6&RID=0) 321 NFCLGPCPYIWSLDTQYSKVLALYNQHNPGASAAPCCVPQVLEPLPIVYYVGRKPKVEQLSNMIVRSCKCS 391

[XP_027833173.1](https://www.ncbi.nlm.nih.gov/protein/XP_027833173.1?report=genbank&log$=protalign&blast_rank=7&RID=0) 321 NFCLGPCPYIWSLDTQYSKVLALYNQHNPGASAAPCCVPQALEPLPIVYYVGRKPKVEQLSNMIVRSCKCS 391

[XP_009433930.1](https://www.ncbi.nlm.nih.gov/protein/XP_009433930.1?report=genbank&log$=protalign&blast_rank=8&RID=0) 321 NFCLGPCPYIWSLDTQYSKVLALYNQHNPGASAAPCCVPQALEPLPIVYYVGRKPKVEQLSNMIVRSCKCS 391

[XP_028695614.1](https://www.ncbi.nlm.nih.gov/protein/XP_028695614.1?report=genbank&log$=protalign&blast_rank=9&RID=0) 321 NFCLGPCPYIWSLDTQYSKVLALYNQHNPGASAAPCCVPQALEPLPIVYYVGRKPKVEQLSNMIVRSCKCS 391

[XP_006941294.1](https://www.ncbi.nlm.nih.gov/protein/XP_006941294.1?report=genbank&log$=protalign&blast_rank=10&RID=0) 321 NFCLGPCPYIWSLDTQYSKVLALYNQHNPGASAAPCCVPQALEPLPIVYYVGRKPKVEQLSNMIVRSCKCS 391

[XP_017917139.1](https://www.ncbi.nlm.nih.gov/protein/XP_017917139.1?report=genbank&log$=protalign&blast_rank=11&RID=0) 321 NFCLGPCPYIWSLDTQYSKVLALYNQHNPGASAAPCCVPQALEPLPIVYYVGRKPKVEQLSNMIVRSCKCS 391

[XP_018870688.1](https://www.ncbi.nlm.nih.gov/protein/XP_018870688.1?report=genbank&log$=protalign&blast_rank=12&RID=0) 321 NFCLGPCPYIWSLDTQYSKVLALYNQHNPGASAAPCCVPQALEPLPIVYYVGRKPKVEQLSNMIVRSCKCS 391

[XP_009230906.2](https://www.ncbi.nlm.nih.gov/protein/XP_009230906.2?report=genbank&log$=protalign&blast_rank=13&RID=0) 320 NFCLGPCPYIWSLDTQYSKVLALYNQHNPGASAAPCCVPQALEPLPIVYYVGRKPKVEQLSNMIVRSCKCS 390

[XP_032028718.1](https://www.ncbi.nlm.nih.gov/protein/XP_032028718.1?report=genbank&log$=protalign&blast_rank=14&RID=0) 320 NFCLGPCPYIWSLDTQYSKVLALYNQHNPGASAAPCCVPQALEPLPIVYYVGRKPKVEQLSNMIVRSCKCS 390

TGFB2

NP_003229.1 transforming growth factor beta-2 proprotein isoform 2 preproprotein [Homo sapiens]

NP_033393.2 transforming growth factor beta-2 proprotein isoform 1 preproprotein [Mus musculus]

NP_112393.2 transforming growth factor beta-2 proprotein precursor [Rattus norvegicus]

XP_005216841.1 transforming growth factor beta-2 isoform X1 [Bos taurus]

XP_020919952.1 transforming growth factor beta-2 isoform X1 [Sus scrofa]

XP_545713.2 transforming growth factor beta-2 proprotein isoform X1 [Canis lupus familiaris]

XP_014970470.1 transforming growth factor beta-2 proprotein isoform X1 [Macaca mulatta]

XP_001172158.1 transforming growth factor beta-2 isoform X1 [Pan troglodytes]

XP_004013651.1 transforming growth factor beta-2 proprotein isoform X1 [Ovis aries]

XP_003364612.1 transforming growth factor beta-2 isoform X1 [Equus caballus]

XP_003999556.1 transforming growth factor beta-2 proprotein isoform X1 [Felis catus]

XP_017915648.1 PREDICTED: transforming growth factor beta-2 isoform X1 [Capra hircus]

XP_004028464.2 transforming growth factor beta-2 proprotein isoform X1 [Gorilla gorilla gorilla]

XP_002809490.1 transforming growth factor beta-2 isoform X1 [Pongo abelii]

XP_031992675.1 transforming growth factor beta-2 proprotein isoform X2 [Hylobates moloch]

[NP_003229.1](https://www.ncbi.nlm.nih.gov/protein/NP_003229.1?report=genbank&log$=protalign&blast_rank=0&RID=0) 1 MHYCVLSAFLILHLVTVALSLSTCSTLDMDQFMRKRIEAIRGQILSKLKLTSPPEDYPEPEEVPPEVISIYNSTRDLLQE 80

[NP_033393.2](https://www.ncbi.nlm.nih.gov/protein/NP_033393.2?report=genbank&log$=protalign&blast_rank=1&RID=0) 1 MHYCVLSTFLLLHLVPVALSLSTCSTLDMDQFMRKRIEAIRGQILSKLKLTSPPEDYPEPDEVPPEVISIYNSTRDLLQE 80

[NP_112393.2](https://www.ncbi.nlm.nih.gov/protein/NP_112393.2?report=genbank&log$=protalign&blast_rank=2&RID=0) 1 MHYCVLRTFLLLHLVPVALSLSTCSTLDMDQFMRKRIEAIRGQILSKLKLTSPPEDYPEPDEVPPEVISIYNSTRDLLQE 80

[XP_005216841.1](https://www.ncbi.nlm.nih.gov/protein/XP_005216841.1?report=genbank&log$=protalign&blast_rank=3&RID=0) 1 MHYCVLSAFLLLHLVTVALSLSTCSTLDMDQFMRKRIEAIRGQILSKLKLTSPPEDYPEPEEVPPEVISIYNSTRDLLQE 80

[XP_020919952.1](https://www.ncbi.nlm.nih.gov/protein/XP_020919952.1?report=genbank&log$=protalign&blast_rank=4&RID=0) 1 MHYCVLSAFLLLHLVTVALSLSTCSTLDMDQFMRKRIEAIRGQILSKLKLTSPPEDYPEPEEVPPEVISIYNSTRDLLQE 80

[XP_545713.2](https://www.ncbi.nlm.nih.gov/protein/XP_545713.2?report=genbank&log$=protalign&blast_rank=5&RID=0) 1 MHYCVLSAFLILHLVTAALSLSTCSTLDMDQFMRKRIEAIRGQILSKLKLTSPPEDYPEPEEVPPEVISIYNSTRDLLQE 80

[XP_014970470.1](https://www.ncbi.nlm.nih.gov/protein/XP_014970470.1?report=genbank&log$=protalign&blast_rank=6&RID=0) 1 MHYCVLSAFLILHLVTVALSLSTCSTLDMDQFMRKRIEAIRGQILSKLKLTSPPEDYPEPEEVPPEVISIYNSTRDLLQE 80

[XP_001172158.1](https://www.ncbi.nlm.nih.gov/protein/XP_001172158.1?report=genbank&log$=protalign&blast_rank=7&RID=0) 1 MHYCVLSAFLILHLVTVALSLSTCSTLDMDQFMRKRIEAIRGQILSKLKLTSPPEDYPEPEEVPPEVISIYNSTRDLLQE 80

[XP_004013651.1](https://www.ncbi.nlm.nih.gov/protein/XP_004013651.1?report=genbank&log$=protalign&blast_rank=8&RID=0) 1 MHYCVLSAFLLLHLVTVALSLSTCSTLDMDQFMRKRIEAIRGQILSKLKLTSPPEDYPEPEEVPPEVISIYNSTRDLLQE 80

[XP_003364612.1](https://www.ncbi.nlm.nih.gov/protein/XP_003364612.1?report=genbank&log$=protalign&blast_rank=9&RID=0) 1 MHYCVLSAFLLLHLVAVALSLSTCSTLDMDQFMRKRIEAIRGQILSKLKLTSPPEDYPEPEEVPPEVISIYNSTRDLLQE 80

[XP_003999556.1](https://www.ncbi.nlm.nih.gov/protein/XP_003999556.1?report=genbank&log$=protalign&blast_rank=10&RID=0) 1 MHYCVLSAFLLLHLVTAALSLSTCSTLDMDQFMRKRIEAIRGQILSKLKLTSPPEDYPEPEEVPPEVISIYNSTRDLLQE 80

[XP_017915648.1](https://www.ncbi.nlm.nih.gov/protein/XP_017915648.1?report=genbank&log$=protalign&blast_rank=11&RID=0) 1 MHYCVLSAFLLLHLVTVALSLSTCSTLDMDQFMRKRIEAIRGQILSKLKLTSPPEDYPEPEEVPPEVISIYNSTRDLLQE 80

[XP_004028464.2](https://www.ncbi.nlm.nih.gov/protein/XP_004028464.2?report=genbank&log$=protalign&blast_rank=12&RID=0) 1 MHYCVLSAFLILHLVTVALSLSTCSTLDMDQFMRKRIEAIRGQILSKLKLTSPPEDYPEPEEVPPEVISIYNSTRDLLQE 80

[XP_002809490.1](https://www.ncbi.nlm.nih.gov/protein/XP_002809490.1?report=genbank&log$=protalign&blast_rank=13&RID=0) 1 MHYCVLSAFLILHLVTVALSLSTCSTLDMDQFMRKRIEAIRGQILSKLKLNSPPEDYPEPEEVPPEVISIYNSTRDLLQE 80

[XP_031992675.1](https://www.ncbi.nlm.nih.gov/protein/XP_031992675.1?report=genbank&log$=protalign&blast_rank=14&RID=0) 1 MHYCVLSAFLILHLVTVALSLSTCSTLDMDQFMRKRIEAIRGQILSKLKLTSPPEDYPEPEEVPPEVISIYNSTRDLLQE 80

[NP_003229.1](https://www.ncbi.nlm.nih.gov/protein/NP_003229.1?report=genbank&log$=protalign&blast_rank=0&RID=0) 81 KASRRAAACERERSDEEYYAKEVYKIDMPPFFPSE----------------------------NAIPPTFYRPYFRIVRF 132

[NP_033393.2](https://www.ncbi.nlm.nih.gov/protein/NP_033393.2?report=genbank&log$=protalign&blast_rank=1&RID=0) 81 KASRRAAACERERSDEEYYAKEVYKIDMPSHLPSE----------------------------NAIPPTFYRPYFRIVRF 132

[NP_112393.2](https://www.ncbi.nlm.nih.gov/protein/NP_112393.2?report=genbank&log$=protalign&blast_rank=2&RID=0) 81 KASRRAAACERERSDEEYYAKEVYKIDMPSHFPSETVCPVVTTSSGSVGSFCSRQSQVLCGYLDAIPPTFYRPYFRIVRF 160

[XP_005216841.1](https://www.ncbi.nlm.nih.gov/protein/XP_005216841.1?report=genbank&log$=protalign&blast_rank=3&RID=0) 81 KASRRAAACERERSDEEYYAKEVYKIDMPSFLPSETVCPVVTTPSGSVGSLCSRQSQVFCGYLDAIPPTFYRPYFRIVRF 160

[XP_020919952.1](https://www.ncbi.nlm.nih.gov/protein/XP_020919952.1?report=genbank&log$=protalign&blast_rank=4&RID=0) 81 KASRRAAACERERSDEEYYAKEVYKIDMPPFFPSETVCPVVTTPSGSVGSLCSRQSQVLCGYLDAIPPTFYRPYFRIVRF 160

[XP_545713.2](https://www.ncbi.nlm.nih.gov/protein/XP_545713.2?report=genbank&log$=protalign&blast_rank=5&RID=0) 81 KASRRAAACERERSDEEYYAKEVYKIDMPPFFPSETVCPVVTTPSGSVGSFCSRQSQVLCGYLDAIPPTFYRPYFRIVRF 160

[XP_014970470.1](https://www.ncbi.nlm.nih.gov/protein/XP_014970470.1?report=genbank&log$=protalign&blast_rank=6&RID=0) 81 KASRRAAACERERSDEEYYAKEVYKIDMPPFFPSETVCPVVTTPSGSVGSLCSRQSQVLCGYLDAIPPTFYRPYFRIVRF 160

[XP_001172158.1](https://www.ncbi.nlm.nih.gov/protein/XP_001172158.1?report=genbank&log$=protalign&blast_rank=7&RID=0) 81 KASRRAAACERERSDEEYYAKEVYKIDMPPFFPSETVCPVVTTPSGSVGSLCSRQSQVLCGYLDAIPPTFYRPYFRIVRF 160

[XP_004013651.1](https://www.ncbi.nlm.nih.gov/protein/XP_004013651.1?report=genbank&log$=protalign&blast_rank=8&RID=0) 81 KASRRAAACERERSDEEYYAKEVYKIDMPSFLPSETVCPVVTTPSGSVGSLCSRQSQVFCGYLDAIPPTFYRPYFRIVRF 160

[XP_003364612.1](https://www.ncbi.nlm.nih.gov/protein/XP_003364612.1?report=genbank&log$=protalign&blast_rank=9&RID=0) 81 KASRRAAACERERSDEEYYAKEVYKIDMPPFFPSETVCPVVTTPSGSVGSLCSRQSQVLCGYLDAIPPTFYRPYFRIVRF 160

[XP_003999556.1](https://www.ncbi.nlm.nih.gov/protein/XP_003999556.1?report=genbank&log$=protalign&blast_rank=10&RID=0) 81 KASRRAAACERERSDEEYYAKEVYKIDMPPFFPSETVCPVVTTPSGSVGSLCSRHSQVLCGYLDAIPPTFYRPYFRIVRF 160

[XP_017915648.1](https://www.ncbi.nlm.nih.gov/protein/XP_017915648.1?report=genbank&log$=protalign&blast_rank=11&RID=0) 81 KASRRAAACERERSDEEYYAKEVYKIDMPSFLPSETVCPVVTTPSGSVGSLCSRQSQVFCGYLDAIPPTFYRPYFRIVRF 160

[XP_004028464.2](https://www.ncbi.nlm.nih.gov/protein/XP_004028464.2?report=genbank&log$=protalign&blast_rank=12&RID=0) 81 KASRRAAACERERSDEEYYAKEVYKIDMPPFFPSETVCPVVTTPSGSVGSLCSRQSQVLCGYLDAIPPTFYRPYFRIVRF 160

[XP_002809490.1](https://www.ncbi.nlm.nih.gov/protein/XP_002809490.1?report=genbank&log$=protalign&blast_rank=13&RID=0) 81 KASRRAAACERERSDEEYYAKEVYKIDMPPFFPSETVCPVVTTPSGSVGSLCSRQSQVLCGYLDAIPPTFYRPYFRIVRF 160

[XP_031992675.1](https://www.ncbi.nlm.nih.gov/protein/XP_031992675.1?report=genbank&log$=protalign&blast_rank=14&RID=0) 81 KASRRAAACERERSDEEYYAKEVYKIDMPPFFPSETVCPVVTTPSGSVGSLCSRQSQVLCGYLDAIPPTFYRPYFRIVRF 160

[NP_003229.1](https://www.ncbi.nlm.nih.gov/protein/NP_003229.1?report=genbank&log$=protalign&blast_rank=0&RID=0) 133 DVSAMEKNASNLVKAEFRVFRLQNPKARVPEQRIELYQILKSKDLTSPTQRYIDSKVVKTRAEGEWLSFDVTDAVHEWLH 212

[NP_033393.2](https://www.ncbi.nlm.nih.gov/protein/NP_033393.2?report=genbank&log$=protalign&blast_rank=1&RID=0) 133 DVSTMEKNASNLVKAEFRVFRLQNPKARVAEQRIELYQILKSKDLTSPTQRYIDSKVVKTRAEGEWLSFDVTDAVQEWLH 212

[NP_112393.2](https://www.ncbi.nlm.nih.gov/protein/NP_112393.2?report=genbank&log$=protalign&blast_rank=2&RID=0) 161 DVSTMEKNASNLVKAEFRVFRLQNPKARVAEQRIELYQILKSKDLTSPTQRYIDSKVVKTRAEGEWLSFDVTDAVHEWLH 240

[XP_005216841.1](https://www.ncbi.nlm.nih.gov/protein/XP_005216841.1?report=genbank&log$=protalign&blast_rank=3&RID=0) 161 DVSSMEKNASNLVKAEFRVFRLQNPKARVPEQRIELYQILKSKDLTSPTQRYIDSKVVKTRAEGEWLSFDVTDAVHEWLH 240

[XP_020919952.1](https://www.ncbi.nlm.nih.gov/protein/XP_020919952.1?report=genbank&log$=protalign&blast_rank=4&RID=0) 161 DVSAMEKNASNLVKAEFRVFRLQNPKARVAEQRIELYQILKSKDLTSPTQRYIDSKVVKTRAEGEWLSFDVTDAVHEWLH 240

[XP_545713.2](https://www.ncbi.nlm.nih.gov/protein/XP_545713.2?report=genbank&log$=protalign&blast_rank=5&RID=0) 161 DVSAMEKNASNLVKAEFRVFRLQNPKARVPEQRIELYQILKSKDLTSPTQRYIDSKVVKTRAEGEWLSFDVTDAVHEWLH 240

[XP_014970470.1](https://www.ncbi.nlm.nih.gov/protein/XP_014970470.1?report=genbank&log$=protalign&blast_rank=6&RID=0) 161 DVSAMEKNASNLVKAEFRVFRLQNPKARVPEQRIELYQILKSKDLTSPTQRYIDSKVVKTRAEGEWLSFDVTDAVHEWLH 240

[XP_001172158.1](https://www.ncbi.nlm.nih.gov/protein/XP_001172158.1?report=genbank&log$=protalign&blast_rank=7&RID=0) 161 DVSAMEKNASNLVKAEFRVFRLQNPKARVPEQRIELYQILKSKDLTSPTQRYIDSKVVKTRAEGEWLSFDVTDAVHEWLH 240

[XP_004013651.1](https://www.ncbi.nlm.nih.gov/protein/XP_004013651.1?report=genbank&log$=protalign&blast_rank=8&RID=0) 161 DVSSMEKNASNLVKAEFRVFRLQNPKARVPEQRIELYQILKSKDLTSPTQRYIDSKVVKTRAEGEWLSFDVTDAVHEWLH 240

[XP_003364612.1](https://www.ncbi.nlm.nih.gov/protein/XP_003364612.1?report=genbank&log$=protalign&blast_rank=9&RID=0) 161 DVSAMEKNASNLVKAEFRVFRLQNPKARVPEQRIELYQILKSKDLTSPTQRYIDSKVVKTRAEGEWLSFDVTDAVHEWLH 240

[XP_003999556.1](https://www.ncbi.nlm.nih.gov/protein/XP_003999556.1?report=genbank&log$=protalign&blast_rank=10&RID=0) 161 DVSAMEKNASNLVKAEFRVFRLQNPKARVPEQRIELYQILKSKDLTSPTQRYIDSKVVKTRAEGEWLSFDVTDAVHEWLH 240

[XP_017915648.1](https://www.ncbi.nlm.nih.gov/protein/XP_017915648.1?report=genbank&log$=protalign&blast_rank=11&RID=0) 161 DVSSMEKNASNLVKAEFRVFRLQNPKARVPEQRIELYQILKSKDLTSPTQRYIDSKVVKTRAEGEWLSFDVTDAVHEWLH 240

[XP_004028464.2](https://www.ncbi.nlm.nih.gov/protein/XP_004028464.2?report=genbank&log$=protalign&blast_rank=12&RID=0) 161 DVSAMEKNASNLVKAEFRVFRLQNPKARVPEQRIELYQILKSKDLTSPTQRYIDSKVVKTRAEGEWLSFDVTDAVHEWLH 240

[XP_002809490.1](https://www.ncbi.nlm.nih.gov/protein/XP_002809490.1?report=genbank&log$=protalign&blast_rank=13&RID=0) 161 DVSAMEKNASNLVKAEFRVFRLQNPKARVPEQRIELYQILKSKDLTSPTQRYIDSKVVKTRAEGEWLSFDVTDAVHEWLH 240

[XP_031992675.1](https://www.ncbi.nlm.nih.gov/protein/XP_031992675.1?report=genbank&log$=protalign&blast_rank=14&RID=0) 161 DVSAMEKNASNLVKAEFRVFRLQNPKARVPEQRIELYQILKSKDLTSPTQRYIDSKVVKTRAEGEWLSFDVTDAVHEWLH 240

[NP_003229.1](https://www.ncbi.nlm.nih.gov/protein/NP_003229.1?report=genbank&log$=protalign&blast_rank=0&RID=0) 213 HKDRNLGFKISLHCPCCTFVPSNNYIIPNKSEELEARFAGIDGTSTYTSGDQKTIKSTRKKNSGKTPHLLLMLLPSYRLE 292

[NP_033393.2](https://www.ncbi.nlm.nih.gov/protein/NP_033393.2?report=genbank&log$=protalign&blast_rank=1&RID=0) 213 HKDRNLGFKISLHCPCCTFVPSNNYIIPNKSEELEARFAGIDGTSTYASGDQKTIKSTRKKTSGKTPHLLLMLLPSYRLE 292

[NP_112393.2](https://www.ncbi.nlm.nih.gov/protein/NP_112393.2?report=genbank&log$=protalign&blast_rank=2&RID=0) 241 HKDRNLGFKISLHCPCCTFIPSNNYIIPNKSQELEARFAGIDGTSTYASGDQKTIKSTRKKSSGKTPHLLLMLLPSYRLE 320

[XP_005216841.1](https://www.ncbi.nlm.nih.gov/protein/XP_005216841.1?report=genbank&log$=protalign&blast_rank=3&RID=0) 241 HKDRNLGFKISLHCPCCTFVPSNNYIIPNKSEELEARFAGIDGTSTYTSGDQKTIKSTRKKNSGKSPHLLLMLLPSYRLE 320

[XP_020919952.1](https://www.ncbi.nlm.nih.gov/protein/XP_020919952.1?report=genbank&log$=protalign&blast_rank=4&RID=0) 241 HKDRNLGFKISLHCPCCTFVPSNNYIIPNKSEELEARFAGIDGTSTYTSGDQKTIKSTRKKNSGKTPHLLLMLLPSYRLE 320

[XP_545713.2](https://www.ncbi.nlm.nih.gov/protein/XP_545713.2?report=genbank&log$=protalign&blast_rank=5&RID=0) 241 HKDRNLGFKISLHCPCCTFVPSNNYIIPNKSEELEARFAGIDGTSTYTSGDQKTIKSTRKKNSGKTPHLLLMLLPSYRLE 320

[XP_014970470.1](https://www.ncbi.nlm.nih.gov/protein/XP_014970470.1?report=genbank&log$=protalign&blast_rank=6&RID=0) 241 HKDRNLGFKISLHCPCCTFVPSNNYIIPNKSEELEARFAGIDGTSTYTSGDQKTIKSTRKKNSGKTPHLLLMLLPSYRLE 320

[XP_001172158.1](https://www.ncbi.nlm.nih.gov/protein/XP_001172158.1?report=genbank&log$=protalign&blast_rank=7&RID=0) 241 HKDRNLGFKISLHCPCCTFVPSNNYIIPNKSEELEARFAGIDGTSTYTSGDQKTIKSTRKKNSGKTPHLLLMLLPSYRLE 320

[XP_004013651.1](https://www.ncbi.nlm.nih.gov/protein/XP_004013651.1?report=genbank&log$=protalign&blast_rank=8&RID=0) 241 HKDRNLGFKISLHCPCCTFVPSNNYIIPNKSEELEARFAGIDGTSTYTSGDQKTIKSTRKKNSGKSPHLLLMLLPSYRLE 320

[XP_003364612.1](https://www.ncbi.nlm.nih.gov/protein/XP_003364612.1?report=genbank&log$=protalign&blast_rank=9&RID=0) 241 HKDRNLGFKISLHCPCCTFVPSNNYIIPNKSEELEARFAGIDGTSTYTSGDQKTIKSTRKKNSGKTPHLLLMLLPSYRLE 320

[XP_003999556.1](https://www.ncbi.nlm.nih.gov/protein/XP_003999556.1?report=genbank&log$=protalign&blast_rank=10&RID=0) 241 HKDRNLGFKISLHCPCCTFVPSNNYIIPNKSEELEARFAGIDGTSTYTSGDQKTIKSTRKKNSGKTPHLLLMLLPSYRLE 320

[XP_017915648.1](https://www.ncbi.nlm.nih.gov/protein/XP_017915648.1?report=genbank&log$=protalign&blast_rank=11&RID=0) 241 HKDRNLGFKISLHCPCCTFVPSNNYIIPNKSEELEARFAGIDGTSTYTSGDQKTIKSTRKKNSGKSPHLLLMLLPSYRLE 320

[XP_004028464.2](https://www.ncbi.nlm.nih.gov/protein/XP_004028464.2?report=genbank&log$=protalign&blast_rank=12&RID=0) 241 HKDRNLGFKISLHCPCCTFVPSNNYIIPNKSEELEARFAGIDGTSTYTSGDQKTIKSTRKKNSGKTPHLLLMLLPSYRLE 320

[XP_002809490.1](https://www.ncbi.nlm.nih.gov/protein/XP_002809490.1?report=genbank&log$=protalign&blast_rank=13&RID=0) 241 HKDRNLGFKISLHCPCCTFVPSNNYIIPNKSEELEARFAGIDGTSTYTSGDQKTIKSTRKKNSGKTPHLLLMLLPSYRLE 320

[XP_031992675.1](https://www.ncbi.nlm.nih.gov/protein/XP_031992675.1?report=genbank&log$=protalign&blast_rank=14&RID=0) 241 HKDRNLGFKISLHCPCCTFVPSNNYIIPNKSEELEARFAGIDGTSTYTSGDQKTIKSTRKKNSGKTPHLLLMLLPSYRLE 320

[NP_003229.1](https://www.ncbi.nlm.nih.gov/protein/NP_003229.1?report=genbank&log$=protalign&blast_rank=0&RID=0) 293 SQQTNRRKKRALDAAYCFRNVQDNCCLRPLYIDFKRDLGWKWIHEPKGYNANFCAGACPYLWSSDTQHSRVLSLYNTINP 372

[NP_033393.2](https://www.ncbi.nlm.nih.gov/protein/NP_033393.2?report=genbank&log$=protalign&blast_rank=1&RID=0) 293 SQQSSRRKKRALDAAYCFRNVQDNCCLRPLYIDFKRDLGWKWIHEPKGYNANFCAGACPYLWSSDTQHTKVLSLYNTINP 372

[NP_112393.2](https://www.ncbi.nlm.nih.gov/protein/NP_112393.2?report=genbank&log$=protalign&blast_rank=2&RID=0) 321 SQQSSRRRKRALDAAYCFRNVQDNCCLRPLYIDFKRDLGWKWIHEPKGYNANFCAGACPYLWSSDTQHTKVLSLYNTINP 400

[XP_005216841.1](https://www.ncbi.nlm.nih.gov/protein/XP_005216841.1?report=genbank&log$=protalign&blast_rank=3&RID=0) 321 SQQSNRRKKRALDAAYCFRNVQDNCCLRPLYIDFKRDLGWKWIHEPKGYNANFCAGACPYLWSSDTQHSRVLSLYNTINP 400

[XP_020919952.1](https://www.ncbi.nlm.nih.gov/protein/XP_020919952.1?report=genbank&log$=protalign&blast_rank=4&RID=0) 321 SQQSNRRKKRALDAAYCFRNVQDNCCLRPLYIDFKRDLGWKWIHEPKGYNANFCAGACPYLWSSDTQHSRVLSLYNTINP 400

[XP_545713.2](https://www.ncbi.nlm.nih.gov/protein/XP_545713.2?report=genbank&log$=protalign&blast_rank=5&RID=0) 321 SQQSNRRKKRALDAAYCFRNVQDNCCLRPLYIDFKRDLGWKWIHEPKGYNANFCAGACPYLWSSDTQHSRVLSLYNTINP 400

[XP_014970470.1](https://www.ncbi.nlm.nih.gov/protein/XP_014970470.1?report=genbank&log$=protalign&blast_rank=6&RID=0) 321 SQQTNRRKKRALDAAYCFRNVQDNCCLRPLYIDFKRDLGWKWIHEPKGYNANFCAGACPYLWSSDTQHSRVLSLYNTINP 400

[XP_001172158.1](https://www.ncbi.nlm.nih.gov/protein/XP_001172158.1?report=genbank&log$=protalign&blast_rank=7&RID=0) 321 SQQTNRRKKRALDAAYCFRNVQDNCCLRPLYIDFKRDLGWKWIHEPKGYNANFCAGACPYLWSSDTQHSRVLSLYNTINP 400

[XP_004013651.1](https://www.ncbi.nlm.nih.gov/protein/XP_004013651.1?report=genbank&log$=protalign&blast_rank=8&RID=0) 321 SQQSNRRKKRALDAAYCFRNVQDNCCLRPLYIDFKRDLGWKWIHEPKGYNANFCAGACPYLWSSDTQHSRVLSLYNTINP 400

[XP_003364612.1](https://www.ncbi.nlm.nih.gov/protein/XP_003364612.1?report=genbank&log$=protalign&blast_rank=9&RID=0) 321 SQQSNRRKKRALDAAYCFRNVQDNCCLRPLYIDFKRDLGWKWIHEPKGYNANFCAGACPYLWSSDTQHSRVLSLYNTINP 400

[XP_003999556.1](https://www.ncbi.nlm.nih.gov/protein/XP_003999556.1?report=genbank&log$=protalign&blast_rank=10&RID=0) 321 SQQSNRRKKRALDAAYCFRNVQDNCCLRPLYIDFKRDLGWKWIHEPKGYNANFCAGACPYLWSSDTQHSRVLSLYNTINP 400

[XP_017915648.1](https://www.ncbi.nlm.nih.gov/protein/XP_017915648.1?report=genbank&log$=protalign&blast_rank=11&RID=0) 321 SQQSNRRKKRALDAAYCFRNVQDNCCLRPLYIDFKRDLGWKWIHEPKGYNANFCAGACPYLWSADTQHSRVLSLYNTINP 400

[XP_004028464.2](https://www.ncbi.nlm.nih.gov/protein/XP_004028464.2?report=genbank&log$=protalign&blast_rank=12&RID=0) 321 SQQTNRRKKRALDAAYCFRNVQDNCCLRPLYIDFKRDLGWKWIHEPKGYNANFCAGACPYLWSSDTQHSRVLSLYNTINP 400

[XP_002809490.1](https://www.ncbi.nlm.nih.gov/protein/XP_002809490.1?report=genbank&log$=protalign&blast_rank=13&RID=0) 321 SQQTNRRKKRALDAAYCFRNVQDNCCLRPLYIDFKRDLGWKWIHEPKGYNANFCAGACPYLWSSDTQHSRVLSLYNTINP 400

[XP_031992675.1](https://www.ncbi.nlm.nih.gov/protein/XP_031992675.1?report=genbank&log$=protalign&blast_rank=14&RID=0) 321 SQQTNRRKKRALDAAYCFRNVQDNCCLRPLYIDFKRDLGWKWIHEPKGYNANFCAGACPYLWSSDTQHSRVLSLYNTINP 400

[NP_003229.1](https://www.ncbi.nlm.nih.gov/protein/NP_003229.1?report=genbank&log$=protalign&blast_rank=0&RID=0) 373 EASASPCCVSQDLEPLTILYYIGKTPKIEQLSNMIVKSCKCS 414

[NP_033393.2](https://www.ncbi.nlm.nih.gov/protein/NP_033393.2?report=genbank&log$=protalign&blast_rank=1&RID=0) 373 EASASPCCVSQDLEPLTILYYIGNTPKIEQLSNMIVKSCKCS 414

[NP_112393.2](https://www.ncbi.nlm.nih.gov/protein/NP_112393.2?report=genbank&log$=protalign&blast_rank=2&RID=0) 401 EASASPCCVSQDLEPLTILYYIGNTPKIEQLSNMIVKSCKCS 442

[XP_005216841.1](https://www.ncbi.nlm.nih.gov/protein/XP_005216841.1?report=genbank&log$=protalign&blast_rank=3&RID=0) 401 EASASPCCVSQDLEPLTILYYIGKTPKIEQLSNMIVKSCKCS 442

[XP_020919952.1](https://www.ncbi.nlm.nih.gov/protein/XP_020919952.1?report=genbank&log$=protalign&blast_rank=4&RID=0) 401 EASASPCCVSQDLEPLTILYYIGKTPKIEQLSNMIVKSCKCS 442

[XP_545713.2](https://www.ncbi.nlm.nih.gov/protein/XP_545713.2?report=genbank&log$=protalign&blast_rank=5&RID=0) 401 EASASPCCVSQDLEPLTILYYIGKTPKIEQLSNMIVKSCKCS 442

[XP_014970470.1](https://www.ncbi.nlm.nih.gov/protein/XP_014970470.1?report=genbank&log$=protalign&blast_rank=6&RID=0) 401 EASASPCCVSQDLEPLTILYYIGKTPKIEQLSNMIVKSCKCS 442

[XP_001172158.1](https://www.ncbi.nlm.nih.gov/protein/XP_001172158.1?report=genbank&log$=protalign&blast_rank=7&RID=0) 401 EASASPCCVSQDLEPLTILYYIGKTPKIEQLSNMIVKSCKCS 442

[XP_004013651.1](https://www.ncbi.nlm.nih.gov/protein/XP_004013651.1?report=genbank&log$=protalign&blast_rank=8&RID=0) 401 EASASPCCVSQDLEPLTILYYIGKTPKIEQLSNMIVKSCKCS 442

[XP_003364612.1](https://www.ncbi.nlm.nih.gov/protein/XP_003364612.1?report=genbank&log$=protalign&blast_rank=9&RID=0) 401 EASASPCCVSQDLEPLTILYYIGKTPKIEQLSNMIVKSCKCS 442

[XP_003999556.1](https://www.ncbi.nlm.nih.gov/protein/XP_003999556.1?report=genbank&log$=protalign&blast_rank=10&RID=0) 401 EASASPCCVSQDLEPLTILYYIGKTPKIEQLSNMIVKSCKCS 442

[XP_017915648.1](https://www.ncbi.nlm.nih.gov/protein/XP_017915648.1?report=genbank&log$=protalign&blast_rank=11&RID=0) 401 EASASPCCVSQDLEPLTILYYIGKTPKIEQLSNMIVKSCKCS 442

[XP_004028464.2](https://www.ncbi.nlm.nih.gov/protein/XP_004028464.2?report=genbank&log$=protalign&blast_rank=12&RID=0) 401 EASASPCCVSQDLEPLTILYYIGKTPKIEQLSNMIVKSCKCS 442

[XP_002809490.1](https://www.ncbi.nlm.nih.gov/protein/XP_002809490.1?report=genbank&log$=protalign&blast_rank=13&RID=0) 401 EASASPCCVSQDLEPLTILYYIGKTPKIEQLSNMIVKSCKCS 442

[XP_031992675.1](https://www.ncbi.nlm.nih.gov/protein/XP_031992675.1?report=genbank&log$=protalign&blast_rank=14&RID=0) 401 EASASPCCVSQDLEPLTILYYIGKTPKIEQLSNMIVKSCKCS 442

TGFB3

NP_003230.1 transforming growth factor beta-3 proprotein isoform 1 preproprotein [Homo sapiens]

NP_033394.2 transforming growth factor beta-3 proprotein precursor [Mus musculus]

NP_037306.1 transforming growth factor beta-3 proprotein preproprotein [Rattus norvegicus]

NP_001094653.1 transforming growth factor beta-3 proprotein [Bos taurus]

XP_005666412.1 transforming growth factor beta-3 isoform X1 [Sus scrofa]

XP_854119.2 transforming growth factor beta-3 proprotein [Canis lupus familiaris]

XP_001492737.1 transforming growth factor beta-3 [Equus caballus]

XP_004010851.1 transforming growth factor beta-3 proprotein [Ovis aries]

XP_001161669.1 transforming growth factor beta-3 isoform X1 [Pan troglodytes]

NP_001244404.1 transforming growth factor beta-3 proprotein precursor [Macaca mulatta]

XP_003987900.1 transforming growth factor beta-3 proprotein [Felis catus]

XP_005686198.1 PREDICTED: transforming growth factor beta-3 [Capra hircus]

XP_004055526.1 transforming growth factor beta-3 proprotein [Gorilla gorilla gorilla]

XP_002825003.1 transforming growth factor beta-3 [Pongo abelii]

XP_031990693.1 transforming growth factor beta-3 proprotein [Hylobates moloch]

[NP_003230.1](https://www.ncbi.nlm.nih.gov/protein/NP_003230.1?report=genbank&log$=protalign&blast_rank=0&RID=0) 1 MKMHLQRALVVLALLNFATVSLSLSTCTTLDFGHIKKKRVEAIRGQILSKLRLTSPPEPTVMTHVPYQVLALYNST 76

[NP_033394.2](https://www.ncbi.nlm.nih.gov/protein/NP_033394.2?report=genbank&log$=protalign&blast_rank=1&RID=0) 1 MKMHLQRALVVLALLNLATISLSLSTCTTLDFGHIKKKRVEAIRGQILSKLRLTSPPEPSVMTHVPYQVLALYNST 76

[NP_037306.1](https://www.ncbi.nlm.nih.gov/protein/NP_037306.1?report=genbank&log$=protalign&blast_rank=2&RID=0) 1 MKMHLQRALVVLALLNLATVSLSLSTCTTLDFGHIKKKRVEAIRGQILSKLRLTSPPEPSVMTHVPYQVLALYNST 76

[NP_001094653.1](https://www.ncbi.nlm.nih.gov/protein/NP_001094653.1?report=genbank&log$=protalign&blast_rank=3&RID=0) 1 [42]LKMHLQRALVVLALLNFATVSLSMSTCTTLDFNHIKRKRVEAIRGQILSKLRLTSPPDPSGLASVPIQVLDLYNST 118

[XP_005666412.1](https://www.ncbi.nlm.nih.gov/protein/XP_005666412.1?report=genbank&log$=protalign&blast_rank=4&RID=0) 1 [45]MKMHLQRALVVLALLNFATVSLSMSTCTTLDFDHIKRKRVEAIRGQILSKLRLTSPPDPSMLANIPTQVLDLYNST 121

[XP_854119.2](https://www.ncbi.nlm.nih.gov/protein/XP_854119.2?report=genbank&log$=protalign&blast_rank=5&RID=0) 1 [45]MKMHLQRALVVLALLNFATVSLSLSTCTTLDFGHIKKKRVEAIRGQILSKLRLTSPPEPSVMTHVPYQVLALYNST 121

[XP_001492737.1](https://www.ncbi.nlm.nih.gov/protein/XP_001492737.1?report=genbank&log$=protalign&blast_rank=6&RID=0) 1 MKMHLQRALVVLALLNFATVSLSLSTCTTLDFGHIKKKRVEAIRGQILSKLRLTSPPEPSVMTHVPYQVLALYNST 76

[XP_004010851.1](https://www.ncbi.nlm.nih.gov/protein/XP_004010851.1?report=genbank&log$=protalign&blast_rank=7&RID=0) 1 [42]LKMHLQRALVVLALLNFATVSLSMSTCTTLDFNHIKRKRVEAIRGQILSKLRLTSPPDPSGLASIPIQVLDLYNST 118

[XP_001161669.1](https://www.ncbi.nlm.nih.gov/protein/XP_001161669.1?report=genbank&log$=protalign&blast_rank=8&RID=0) 1 MKMHLQRALVVLALLNFATVSLSLSTCTTLDFGHIKKKRVEAIRGQILSKLRLTSPPEPTVMTHVPYQVLALYNST 76

[NP_001244404.1](https://www.ncbi.nlm.nih.gov/protein/NP_001244404.1?report=genbank&log$=protalign&blast_rank=9&RID=0) 1 MKMHLQRALVVLALLNFASVSLSLSTCTTLDFGHIKKKRVEAIRGQILSKLRLTSPPEPTVMTHVPYQVLALYNST 76

[XP_003987900.1](https://www.ncbi.nlm.nih.gov/protein/XP_003987900.1?report=genbank&log$=protalign&blast_rank=10&RID=0) 1 MKMHLHRALVVLALLNFATVSLSLSTCTTLDFGHIKKKRVEAIRGQILSKLRLTSPPEPSVMTHVPYQVLALYNST 76

[XP_005686198.1](https://www.ncbi.nlm.nih.gov/protein/XP_005686198.1?report=genbank&log$=protalign&blast_rank=11&RID=0) 1 [42]LKMHLQRALVVLALLNFATVSLSMSTCTTLDFNHIKRKRVEAIRGQILSKLRLTSPPDPSGLASIPIQVLDLYNST 118

[XP_004055526.1](https://www.ncbi.nlm.nih.gov/protein/XP_004055526.1?report=genbank&log$=protalign&blast_rank=12&RID=0) 1 MKMHLQRALVVLALLNFATVSLSLSTCTTLDFGHIKKKRVEAIRGQILSKLRLTSPPEPTVMTHVPYQVLALYNST 76

[XP_002825003.1](https://www.ncbi.nlm.nih.gov/protein/XP_002825003.1?report=genbank&log$=protalign&blast_rank=13&RID=0) 1 MKMHLQRALVVLALLNFATVSLSLSTCTTLDFGHIKKKRVEAIRGQILSKLRLTSPPEPTVMTHVPYQVLALYNST 76

[XP_031990693.1](https://www.ncbi.nlm.nih.gov/protein/XP_031990693.1?report=genbank&log$=protalign&blast_rank=14&RID=0) 1 MKMHLQRALVVLALLNFATVSLSLSTCTTLDFGHIKKKRVEAIRGQILSKLRLTSPPEPTVMTHVPYQVLALYNST 76

[NP_003230.1](https://www.ncbi.nlm.nih.gov/protein/NP_003230.1?report=genbank&log$=protalign&blast_rank=0&RID=0) 77 RELLEEMHGEREEGCTQENTESEYYAKEIHKFDMIQGLAEHNELAVCPKGITSKVFRFNVSSVEKNRTNLFRAEFRVLRV 156

[NP_033394.2](https://www.ncbi.nlm.nih.gov/protein/NP_033394.2?report=genbank&log$=protalign&blast_rank=1&RID=0) 77 RELLEEMHGEREEGCTQETSESEYYAKEIHKFDMIQGLAEHNELAVCPKGITSKVFRFNVSSVEKNGTNLFRAEFRVLRV 156

[NP_037306.1](https://www.ncbi.nlm.nih.gov/protein/NP_037306.1?report=genbank&log$=protalign&blast_rank=2&RID=0) 77 RELLEEMHGEREEGCTQETSESEYYAKEIHKFDMIQGLAEHNELAVCPKGITSKVFRFNVSSVEKNGTNLFRAEFRVLRV 156

[NP_001094653.1](https://www.ncbi.nlm.nih.gov/protein/NP_001094653.1?report=genbank&log$=protalign&blast_rank=3&RID=0) 119 RELLEEVHGERGDVCTQANTESEYYAKEIYKFDMIQGLEEHNDLTVCPKGITSKIFRFNVSSVEKNETNLFRAEFRVFRM 198

[XP_005666412.1](https://www.ncbi.nlm.nih.gov/protein/XP_005666412.1?report=genbank&log$=protalign&blast_rank=4&RID=0) 122 RELLEEVHGERGDDCTQENTESEYYAKEIYKFDMIQGLEEHNDLAVCPKGITSKIFRFNVSSVEKNETNLFRAEFRVLRM 201

[XP_854119.2](https://www.ncbi.nlm.nih.gov/protein/XP_854119.2?report=genbank&log$=protalign&blast_rank=5&RID=0) 122 RELLEEMQGEREDSCTQENTESEYYAKEIHKFDMIQGLAEHNELAVCPKGITSKVFRFNVSSVEKNGTNLFRAEFRVLRV 201

[XP_001492737.1](https://www.ncbi.nlm.nih.gov/protein/XP_001492737.1?report=genbank&log$=protalign&blast_rank=6&RID=0) 77 RELLEEMHGEREDGCTQENTESEYYAKEIHKFDMIQGLAEHNELAVCPKGITSKVFRFNVSSVEKNGTNLFRAEFRVLRV 156

[XP_004010851.1](https://www.ncbi.nlm.nih.gov/protein/XP_004010851.1?report=genbank&log$=protalign&blast_rank=7&RID=0) 119 RELLEEVHGERGDVCTQANTESEYYAKEIYKFDMIQGLEEHNDLTVCPKGITSKIFRFNVSSVEKNETNLFRAEFRVFRM 198

[XP_001161669.1](https://www.ncbi.nlm.nih.gov/protein/XP_001161669.1?report=genbank&log$=protalign&blast_rank=8&RID=0) 77 RELLEEMHGEREEGCTQENTESEYYAKEIHKFDMIQGLAEHNELAVCPKGITSKVFRFNVSSVEKNRTNLFRAEFRVLRV 156

[NP_001244404.1](https://www.ncbi.nlm.nih.gov/protein/NP_001244404.1?report=genbank&log$=protalign&blast_rank=9&RID=0) 77 RELLEEMHGEREEGCTQENTESEYYAKEIHKFDMIQGLAEHNELAVCPKGITSKVFRFNVSSVEKNRTNLFRAEFRVLRV 156

[XP_003987900.1](https://www.ncbi.nlm.nih.gov/protein/XP_003987900.1?report=genbank&log$=protalign&blast_rank=10&RID=0) 77 RELLEEMQGEREDSCTQENTESEYYAKEIHKFDMIQGLAEHNELAVCPKGITSKVFRFNVSSVEKNGTNLFRAEFRVLRV 156

[XP_005686198.1](https://www.ncbi.nlm.nih.gov/protein/XP_005686198.1?report=genbank&log$=protalign&blast_rank=11&RID=0) 119 RELLEEVHGERGDVCTQANTESEYYAKEIYKFDMIQGLEEHNDLTVCPKGITSKIFRFNVSSVEKNETNLFRAEFRVFRM 198

[XP_004055526.1](https://www.ncbi.nlm.nih.gov/protein/XP_004055526.1?report=genbank&log$=protalign&blast_rank=12&RID=0) 77 RELLEEMHGEREEGCTQENTESEYYAKEIHKFDMIQGLAEHNELAVCPKGITSKVFRFNVSSVEKNRTNLFRAEFRVLRV 156

[XP_002825003.1](https://www.ncbi.nlm.nih.gov/protein/XP_002825003.1?report=genbank&log$=protalign&blast_rank=13&RID=0) 77 RELLEEMHGEREEGCTQENTESEYYAKEIHKFDMIQGLAEHNELAVCPKGITSKVFRFNVSSVEKNRTNLFRAEFRVLRV 156

[XP_031990693.1](https://www.ncbi.nlm.nih.gov/protein/XP_031990693.1?report=genbank&log$=protalign&blast_rank=14&RID=0) 77 RELLEEMHGEREEGCTQENTESEYYAKEIHKFDMIQGLAEHNELAVCPKGITSKVFRFNVSSVEKNRTNLFRAEFRVLRV 156

[NP_003230.1](https://www.ncbi.nlm.nih.gov/protein/NP_003230.1?report=genbank&log$=protalign&blast_rank=0&RID=0) 157 PNPSSKRNEQRIELFQILRPDEHIAKQRYIGGKNLPTRGTAEWLSFDVTDTVREWLLRRESNLGLEISIHCPCHTFQPNG 236

[NP_033394.2](https://www.ncbi.nlm.nih.gov/protein/NP_033394.2?report=genbank&log$=protalign&blast_rank=1&RID=0) 157 PNPSSKRTEQRIELFQILRPDEHIAKQRYIGGKNLPTRGTAEWLSFDVTDTVREWLLRRESNLGLEISIHCPCHTFQPNG 236

[NP_037306.1](https://www.ncbi.nlm.nih.gov/protein/NP_037306.1?report=genbank&log$=protalign&blast_rank=2&RID=0) 157 PNPSSKRTEQRIELFQILRPDEHIAKQRYIGGKNLPTRGTAEWLSFDVTDTVREWLLRRESNLGLEISIHCPCHTFQPNG 236

[NP_001094653.1](https://www.ncbi.nlm.nih.gov/protein/NP_001094653.1?report=genbank&log$=protalign&blast_rank=3&RID=0) 199 PNPASKRSEQRIELFQILQPGEHIAKQRYIDGKNLPTRGTGEWLSFDVTDTVREWLLRRESNLGLEISIHCPCHTFQPNG 278

[XP_005666412.1](https://www.ncbi.nlm.nih.gov/protein/XP_005666412.1?report=genbank&log$=protalign&blast_rank=4&RID=0) 202 PNPSSKRSEQRIELFQILQPDEHIAKQRYIDGKNLPTRGAAEWLSFDVTDTVREWLLRRESNLGLEISIHCPCHTFQPNG 281

[XP_854119.2](https://www.ncbi.nlm.nih.gov/protein/XP_854119.2?report=genbank&log$=protalign&blast_rank=5&RID=0) 202 PNPSSKRSEQRIELFQILRPDEHIAKQRYIGGKNLPTRGTAEWLSFDVTDTVREWLLRRESNLGLEISIHCPCHTFQPNG 281

[XP_001492737.1](https://www.ncbi.nlm.nih.gov/protein/XP_001492737.1?report=genbank&log$=protalign&blast_rank=6&RID=0) 157 PNPSSKRNEQRIELFQILRPDEHIAKQRYIGGKNLPTRGTAEWLSFDVTDTVREWLLRRESNLGLEISIHCPCHTFQPNG 236

[XP_004010851.1](https://www.ncbi.nlm.nih.gov/protein/XP_004010851.1?report=genbank&log$=protalign&blast_rank=7&RID=0) 199 PNPASKRSEQRIELFQILQPGEHIAKQRYIDGKNLPTRGTGEWLSFDVTDTVREWLLRRESNLGLEISIHCPCHTFQPNG 278

[XP_001161669.1](https://www.ncbi.nlm.nih.gov/protein/XP_001161669.1?report=genbank&log$=protalign&blast_rank=8&RID=0) 157 PNPSSKRNEQRIELFQILRPDEHIAKQRYIGGKNLPTRGTAEWLSFDVTDTVREWLLRRESNLGLEISIHCPCHTFQPNG 236

[NP_001244404.1](https://www.ncbi.nlm.nih.gov/protein/NP_001244404.1?report=genbank&log$=protalign&blast_rank=9&RID=0) 157 PNPSSKRNEQRIELFQILRPDEHIAKQRYIGGKNLPTRGTAEWLSFDVTDTVREWLLRRESNLGLEISIHCPCHTFQPNG 236

[XP_003987900.1](https://www.ncbi.nlm.nih.gov/protein/XP_003987900.1?report=genbank&log$=protalign&blast_rank=10&RID=0) 157 PNPSSKRSEQRIELFQILRPDEHIAKQRYIGGKNLPTRGTAEWLSFDVTDTVREWLLRRESNLGLEISIHCPCHTFQPNG 236

[XP_005686198.1](https://www.ncbi.nlm.nih.gov/protein/XP_005686198.1?report=genbank&log$=protalign&blast_rank=11&RID=0) 199 PNPASKRSEQRIELFQILQPGEHIAKQRYIDGKNLPTRGTGEWLSFDVTDTVREWLLRRESNLGLEISIHCPCHTFQPNG 278

[XP_004055526.1](https://www.ncbi.nlm.nih.gov/protein/XP_004055526.1?report=genbank&log$=protalign&blast_rank=12&RID=0) 157 PNPSSKRNEQRIELFQILRPDEHIAKQRYIGGKNLPTRGTAEWLSFDVTDTVREWLLRRESNLGLEISIHCPCHTFQPNG 236

[XP_002825003.1](https://www.ncbi.nlm.nih.gov/protein/XP_002825003.1?report=genbank&log$=protalign&blast_rank=13&RID=0) 157 PNPSSKRNEQRIELFQILRPDEHIAKQRYIGGKNLPTRGTAEWLSFDVTDTVREWLLRRESNLGLEISIHCPCHTFQPNG 236

[XP_031990693.1](https://www.ncbi.nlm.nih.gov/protein/XP_031990693.1?report=genbank&log$=protalign&blast_rank=14&RID=0) 157 PNPSSKRNEQRIELFQILRPDEHIAKQRYIGGKNLPTRGTAEWLSFDVTDTVREWLLRRESNLGLEISIHCPCHTFQPNG 236

[NP_003230.1](https://www.ncbi.nlm.nih.gov/protein/NP_003230.1?report=genbank&log$=protalign&blast_rank=0&RID=0) 237 DILENIHEVMEIKFKGVDNEDDHGRGDLGRLKKQKDHHNPHLILMMIPPHRLDNPGQGGQRKKRALDTNYCFRNLEENCC 316

[NP_033394.2](https://www.ncbi.nlm.nih.gov/protein/NP_033394.2?report=genbank&log$=protalign&blast_rank=1&RID=0) 237 DILENVHEVMEIKFKGVDNEDDHGRGDLGRLKKQKDHHNPHLILMMIPPHRLDSPGQGSQRKKRALDTNYCFRNLEENCC 316

[NP_037306.1](https://www.ncbi.nlm.nih.gov/protein/NP_037306.1?report=genbank&log$=protalign&blast_rank=2&RID=0) 237 DILENVHEVMEIKFKGVDNEDDHGRGDLGRLKKQKDHHNPHLILMMIPPHRLDSPGQGGQRKKRALDTNYCFRNLEENCC 316

[NP_001094653.1](https://www.ncbi.nlm.nih.gov/protein/NP_001094653.1?report=genbank&log$=protalign&blast_rank=3&RID=0) 279 DILENIQELMEIKFKGVDSDDDPGRGDLGRLKKKKE-HIPHLILMMIPPNRLDSPG-HSQRKKRALDTNYCFRNLEENCC 356

[XP_005666412.1](https://www.ncbi.nlm.nih.gov/protein/XP_005666412.1?report=genbank&log$=protalign&blast_rank=4&RID=0) 282 DILENIQEVMEIKFKGVDSEDDPGRGDLGRLKKKKE-HSPHLILMMIPPDRLDNPGLGAQRKKRALDTNYCFRNLEENCC 360

[XP_854119.2](https://www.ncbi.nlm.nih.gov/protein/XP_854119.2?report=genbank&log$=protalign&blast_rank=5&RID=0) 282 DILENIHEVMEIKFKGVDSEEDHGRGDLGRLKKQKDHHNPHLILMMIPPHRLDNPGQGGQRKKRALDTNYCFRNLEENCC 361

[XP_001492737.1](https://www.ncbi.nlm.nih.gov/protein/XP_001492737.1?report=genbank&log$=protalign&blast_rank=6&RID=0) 237 DILENIHEVMEIKFKGVDSEDDHGRGDLGRLKKQKDHHNPHLILMMIPPHRLDNPGQGGQRKKRALDTNYCFRNLEENCC 316

[XP_004010851.1](https://www.ncbi.nlm.nih.gov/protein/XP_004010851.1?report=genbank&log$=protalign&blast_rank=7&RID=0) 279 DILENIQELMEIKFKGVDSDDDPGRGDLGRLKKKKE-HIPHLILMMIPPNRLDSPG-HSQRKKRALDTNYCFRNLEENCC 356

[XP_001161669.1](https://www.ncbi.nlm.nih.gov/protein/XP_001161669.1?report=genbank&log$=protalign&blast_rank=8&RID=0) 237 DILENIHEVMEIKFKGVDNEDDHGRGDLGRLKKQKDHHNPHLILMMIPPHRLDNPGQGGQRKKRALDTNYCFRNLEENCC 316

[NP_001244404.1](https://www.ncbi.nlm.nih.gov/protein/NP_001244404.1?report=genbank&log$=protalign&blast_rank=9&RID=0) 237 DILENIHEVMEIKFKGVDNEDDHGRGDLGRLKKQKDHHNPHLILMMIPPHRLDNPGQGGQRKKRALDTNYCFRNLEENCC 316

[XP_003987900.1](https://www.ncbi.nlm.nih.gov/protein/XP_003987900.1?report=genbank&log$=protalign&blast_rank=10&RID=0) 237 DILENIHEVMEIKFKGVDSEDDHGRGDLGRLKKQKDHHNPHLILMMIPPHRLDNPGQGGQRKKRALDTNYCFRNLEENCC 316

[XP_005686198.1](https://www.ncbi.nlm.nih.gov/protein/XP_005686198.1?report=genbank&log$=protalign&blast_rank=11&RID=0) 279 DILENIQELMEIKFKGVDSDDDPGRGDLGRLKKKKE-HIPHLILMMIPPNRLDSPG-HSQRKKRALDTNYCFRNLEENCC 356

[XP_004055526.1](https://www.ncbi.nlm.nih.gov/protein/XP_004055526.1?report=genbank&log$=protalign&blast_rank=12&RID=0) 237 DILENIHEVMEIKFKGVDNEDDHGRGDLGRLKKQKDHHNPHLILMMIPPHRLDNPGQGGQRKKRALDTNYCFRNLEENCC 316

[XP_002825003.1](https://www.ncbi.nlm.nih.gov/protein/XP_002825003.1?report=genbank&log$=protalign&blast_rank=13&RID=0) 237 DILENIHEVMEIKFKGVDNEDDHGRGDLGRLKKQKDHHNPHLILMMIPPHRLDNPGQGGQRKKRALDTNYCFRNLEENCC 316

[XP_031990693.1](https://www.ncbi.nlm.nih.gov/protein/XP_031990693.1?report=genbank&log$=protalign&blast_rank=14&RID=0) 237 DILENIHEVMEIKFKGVDNEDDHGRGDLGRLKKQKDHHNPHLILMMIPPHRLDNPGQGGQRKKRALDTNYCFRNLEENCC 316

[NP_003230.1](https://www.ncbi.nlm.nih.gov/protein/NP_003230.1?report=genbank&log$=protalign&blast_rank=0&RID=0) 317 VRPLYIDFRQDLGWKWVHEPKGYYANFCSGPCPYLRSADTTHSTVLGLYNTLNPEASASPCCVPQDLEPLTILYYVGRTP 396

[NP_033394.2](https://www.ncbi.nlm.nih.gov/protein/NP_033394.2?report=genbank&log$=protalign&blast_rank=1&RID=0) 317 VRPLYIDFRQDLGWKWVHEPKGYYANFCSGPCPYLRSADTTHSTVLGLYNTLNPEASASPCCVPQDLEPLTILYYVGRTP 396

[NP_037306.1](https://www.ncbi.nlm.nih.gov/protein/NP_037306.1?report=genbank&log$=protalign&blast_rank=2&RID=0) 317 VRPLYIDFRQDLGWKWVHEPKGYYANFCSGPCPYLRSSDTTHSTVLGLYNTLNPEASASPCCVPQDLEPLTILYYVGRTP 396

[NP_001094653.1](https://www.ncbi.nlm.nih.gov/protein/NP_001094653.1?report=genbank&log$=protalign&blast_rank=3&RID=0) 357 VRPLYIDFRQDLGWKWVHEPKGYYANFCSGPCPYLRSSDTTHSTVLGLYNTLNPEASASPCCVPQDLEPLTILYYVGRTP 436

[XP_005666412.1](https://www.ncbi.nlm.nih.gov/protein/XP_005666412.1?report=genbank&log$=protalign&blast_rank=4&RID=0) 361 VRPLYIDFRQDLGWKWVHEPKGYYANFCSGPCPYLRSADTTHSSVLGLYNTLNPEASASPCCVPQDLEPLTILYYVGRTA 440

[XP_854119.2](https://www.ncbi.nlm.nih.gov/protein/XP_854119.2?report=genbank&log$=protalign&blast_rank=5&RID=0) 362 VRPLYIDFRQDLGWKWVHEPKGYYANFCSGPCPYLRSADTTHSTVLGLYNTLNPEASASPCCVPQDLEPLTILYYVGRTP 441

[XP_001492737.1](https://www.ncbi.nlm.nih.gov/protein/XP_001492737.1?report=genbank&log$=protalign&blast_rank=6&RID=0) 317 VRPLYIDFRQDLGWKWVHEPKGYYANFCSGPCPYLRSADTTHSTVLGLYNTLNPEASASPCCVPQDLEPLTILYYVGRTP 396

[XP_004010851.1](https://www.ncbi.nlm.nih.gov/protein/XP_004010851.1?report=genbank&log$=protalign&blast_rank=7&RID=0) 357 VRPLYIDFRQDLGWKWVHEPKGYYANFCSGPCPYLRSSDTTHSTVLGLYNTLNPEASASPCCVPQDLEPLTILYYVGRTP 436

[XP_001161669.1](https://www.ncbi.nlm.nih.gov/protein/XP_001161669.1?report=genbank&log$=protalign&blast_rank=8&RID=0) 317 VRPLYIDFRQDLGWKWVHEPKGYYANFCSGPCPYLRSADTTHSTVLGLYNTLNPEASASPCCVPQDLEPLTILYYVGRTP 396

[NP_001244404.1](https://www.ncbi.nlm.nih.gov/protein/NP_001244404.1?report=genbank&log$=protalign&blast_rank=9&RID=0) 317 VRPLYIDFRQDLGWKWVHEPKGYYANFCSGPCPYLRSADTTHSTVLGLYNTLNPEASASPCCVPQDLEPLTILYYVGRTP 396

[XP_003987900.1](https://www.ncbi.nlm.nih.gov/protein/XP_003987900.1?report=genbank&log$=protalign&blast_rank=10&RID=0) 317 VRPLYIDFRQDLGWKWVHEPKGYYANFCSGPCPYLRSADTTHSTVLGLYNTLNPEASASPCCVPQDLEPLTILYYVGRTP 396

[XP_005686198.1](https://www.ncbi.nlm.nih.gov/protein/XP_005686198.1?report=genbank&log$=protalign&blast_rank=11&RID=0) 357 VRPLYIDFRQDLGWKWVHEPKGYYANFCSGPCPYLRSSDTTHSTVLGLYNTLNPEASASPCCVPQDLEPLTILYYVGRTP 436

[XP_004055526.1](https://www.ncbi.nlm.nih.gov/protein/XP_004055526.1?report=genbank&log$=protalign&blast_rank=12&RID=0) 317 VRPLYIDFRQDLGWKWVHEPKGYYANFCSGPCPYLRSADTTHSTVLGLYNTLNPEASASPCCVPQDLEPLTILYYVGRTP 396

[XP_002825003.1](https://www.ncbi.nlm.nih.gov/protein/XP_002825003.1?report=genbank&log$=protalign&blast_rank=13&RID=0) 317 VRPLYIDFRQDLGWKWVHEPKGYYANFCSGPCPYLRSADTTHSTVLGLYNTLNPEASASPCCVPQDLEPLTILYYVGRTP 396

[XP_031990693.1](https://www.ncbi.nlm.nih.gov/protein/XP_031990693.1?report=genbank&log$=protalign&blast_rank=14&RID=0) 317 VRPLYIDFRQDLGWKWVHEPKGYYANFCSGPCPYLRSADTTHSTVLGLYNTLNPEASASPCCVPQDLEPLTILYYVGRTP 396

[NP_003230.1](https://www.ncbi.nlm.nih.gov/protein/NP_003230.1?report=genbank&log$=protalign&blast_rank=0&RID=0) 397 KVEQLSNMVVKSCKCS 412

[NP_033394.2](https://www.ncbi.nlm.nih.gov/protein/NP_033394.2?report=genbank&log$=protalign&blast_rank=1&RID=0) 397 KVEQLSNMVVKSCKCS 412

[NP_037306.1](https://www.ncbi.nlm.nih.gov/protein/NP_037306.1?report=genbank&log$=protalign&blast_rank=2&RID=0) 397 KVEQLSNMVVKSCKCS 412

[NP_001094653.1](https://www.ncbi.nlm.nih.gov/protein/NP_001094653.1?report=genbank&log$=protalign&blast_rank=3&RID=0) 437 KVEQLSNMVVKSCKCS 452

[XP_005666412.1](https://www.ncbi.nlm.nih.gov/protein/XP_005666412.1?report=genbank&log$=protalign&blast_rank=4&RID=0) 441 KVEQLSNMVVKSCKCS 456

[XP_854119.2](https://www.ncbi.nlm.nih.gov/protein/XP_854119.2?report=genbank&log$=protalign&blast_rank=5&RID=0) 442 KVEQLSNMVVKSCKCS 457

[XP_001492737.1](https://www.ncbi.nlm.nih.gov/protein/XP_001492737.1?report=genbank&log$=protalign&blast_rank=6&RID=0) 397 KVEQLSNMVVKSCKCS 412

[XP_004010851.1](https://www.ncbi.nlm.nih.gov/protein/XP_004010851.1?report=genbank&log$=protalign&blast_rank=7&RID=0) 437 KVEQLSNMVVKSCKCS 452

[XP_001161669.1](https://www.ncbi.nlm.nih.gov/protein/XP_001161669.1?report=genbank&log$=protalign&blast_rank=8&RID=0) 397 KVEQLSNMVVKSCKCS 412

[NP_001244404.1](https://www.ncbi.nlm.nih.gov/protein/NP_001244404.1?report=genbank&log$=protalign&blast_rank=9&RID=0) 397 KVEQLSNMVVKSCKCS 412

[XP_003987900.1](https://www.ncbi.nlm.nih.gov/protein/XP_003987900.1?report=genbank&log$=protalign&blast_rank=10&RID=0) 397 KVEQLSNMVVKSCKCS 412

[XP_005686198.1](https://www.ncbi.nlm.nih.gov/protein/XP_005686198.1?report=genbank&log$=protalign&blast_rank=11&RID=0) 437 KVEQLSNMVVKSCKCS 452

[XP_004055526.1](https://www.ncbi.nlm.nih.gov/protein/XP_004055526.1?report=genbank&log$=protalign&blast_rank=12&RID=0) 397 KVEQLSNMVVKSCKCS 412

[XP_002825003.1](https://www.ncbi.nlm.nih.gov/protein/XP_002825003.1?report=genbank&log$=protalign&blast_rank=13&RID=0) 397 KVEQLSNMVVKSCKCS 412

[XP_031990693.1](https://www.ncbi.nlm.nih.gov/protein/XP_031990693.1?report=genbank&log$=protalign&blast_rank=14&RID=0) 397 KVEQLSNMVVKSCKCS 412

BMP8A/B

NP_861525.2 bone morphogenetic protein 8A preproprotein [Homo sapiens]

NP_001711.2 bone morphogenetic protein 8B preproprotein [Homo sapiens]

NP_031584.1 bone morphogenetic protein 8A isoform 2 preproprotein [Mus musculus]

NP_031585.2 bone morphogenetic protein 8B preproprotein [Mus musculus]

NP_001102902.1 bone morphogenetic protein 8A precursor [Rattus norvegicus]

XP_002729572.1 bone morphogenetic protein 8B isoform X1 [Rattus norvegicus]

XP_024845991.1 bone morphogenetic protein 8A [Bos taurus]

XP_020953264.1 LOW QUALITY PROTEIN: bone morphogenetic protein 8A [Sus scrofa]

XP_003356374.4 LOW QUALITY PROTEIN: bone morphogenetic protein 8B [Sus scrofa]

XP_038477617.1 LOW QUALITY PROTEIN: bone morphogenetic protein 8B isoform X1 [Canis lupus familiaris]

XP_038543221.1 LOW QUALITY PROTEIN: bone morphogenetic protein 8B isoform X1 [Canis lupus familiaris]

XP_023488611.1 bone morphogenetic protein 8B [Equus caballus]

XP_023483599.1 bone morphogenetic protein 8B [Equus caballus]

XP_014947141.3 bone morphogenetic protein 8B [Ovis aries]

XP_027824135.1 bone morphogenetic protein 8B [Ovis aries]

XP_024213824.1 bone morphogenetic protein 8A [Pan troglodytes]

XP_016815266.2 bone morphogenetic protein 8B [Pan troglodytes]

XP_028691411.1 bone morphogenetic protein 8B isoform X2 [Macaca mulatta]

XP_028691408.1 bone morphogenetic protein 8B isoform X1 [Macaca mulatta]

XP_014991435.2 bone morphogenetic protein 8B [Macaca mulatta]

XP_023113593.1 LOW QUALITY PROTEIN: bone morphogenetic protein 8A [Felis catus]

XP_017897638.1 PREDICTED: bone morphogenetic protein 8A [Capra hircus]

XP_017897686.1 PREDICTED: bone morphogenetic protein 8B [Capra hircus]

XP_004025569.3 bone morphogenetic protein 8A [Gorilla gorilla gorilla]

XP_030864436.1 bone morphogenetic protein 8B [Gorilla gorilla gorilla]

XP_024103873.1 bone morphogenetic protein 8B-like [Pongo abelii]

XP_009250410.2 bone morphogenetic protein 8B [Pongo abelii]

XP_032016255.1 bone morphogenetic protein 8A [Hylobates moloch]

[NP_861525.2](https://www.ncbi.nlm.nih.gov/protein/NP_861525.2?report=genbank&log$=protalign&blast_rank=0&RID=0) 1 MA AR PGPLWLLGLT LCALGGGG-PGLRPPPGCPQRRLGARERRDVQREILAVLGLPGRPRPR A 62

[NP_001711.2](https://www.ncbi.nlm.nih.gov/protein/NP_001711.2?report=genbank&log$=protalign&blast_rank=1&RID=0) 1 MT AL PGPLWLLGLA LCALGGGG-PGLRPPPGCPQRRLGARERRDVQREILAVLGLPGRPRPR A 62

[NP_031584.1](https://www.ncbi.nlm.nih.gov/protein/NP_031584.1?report=genbank&log$=protalign&blast_rank=2&RID=0) 1 MA MR PGPLWLLGLA LCALGGGH--GPRPPHTCPQRRLGARERRDMQREILAVLGLPGRPRPR A 61

[NP_031585.2](https://www.ncbi.nlm.nih.gov/protein/NP_031585.2?report=genbank&log$=protalign&blast_rank=3&RID=0) 1 MA AR PGLLWLLGLA LCVLGGGH--LSHPPHVFPQRRLGVREPRDMQREIREVLGLPGRPRSR A 61

[NP_001102902.1](https://www.ncbi.nlm.nih.gov/protein/NP_001102902.1?report=genbank&log$=protalign&blast_rank=4&RID=0) 1 MA VR PGPLWLLGLA LCALGGGH--GPRPPHTCPQRRLGARERRDMQREILAVLGLPGRPRPR A 61

[XP_002729572.1](https://www.ncbi.nlm.nih.gov/protein/XP_002729572.1?report=genbank&log$=protalign&blast_rank=5&RID=0) 1 MA AG PGLLWLLGLA LCVLGSSH--LPRSPHVFPQHRLGVREPRDMQREIREVLGLPGRPRSR A 61

[XP_024845991.1](https://www.ncbi.nlm.nih.gov/protein/XP_024845991.1?report=genbank&log$=protalign&blast_rank=6&RID=0) 1 MA AR PGPLWLLGLA LCALSGGGvPGPRPPLGCPQRRLGPRERRDLQREILAVLGLPGRPRPR A 63

[XP_020953264.1](https://www.ncbi.nlm.nih.gov/protein/XP_020953264.1?report=genbank&log$=protalign&blast_rank=7&RID=0) 1 MA TC PAAVRLAT-- --------------PPGCPQSRLGPRERRDLQREILAVLGLPGRPXPR A 47

[XP_003356374.4](https://www.ncbi.nlm.nih.gov/protein/XP_003356374.4?report=genbank&log$=protalign&blast_rank=8&RID=0) 1 MA AR PGTLWLLGLV LCTLSGCG-PGPRPPPGCPQSRLGPRERRDLQREILAVLGLPGRPRPR A 62

[XP_038477617.1](https://www.ncbi.nlm.nih.gov/protein/XP_038477617.1?report=genbank&log$=protalign&blast_rank=9&RID=0) 1 MA AR PGPLWLVGLA LCALSGGG-PGPRPPAGCPARRLGPRERRDMQREILAVLGLPGRPRPR A 62

[XP_038543221.1](https://www.ncbi.nlm.nih.gov/protein/XP_038543221.1?report=genbank&log$=protalign&blast_rank=10&RID=0) 1 MA[14]AR[27]PGPLWLVGLA LCALSGGG-PGPRPRPGRPPPWAAARERRDMQREILAVLGLPGRPRPR A 103

[XP_023488611.1](https://www.ncbi.nlm.nih.gov/protein/XP_023488611.1?report=genbank&log$=protalign&blast_rank=11&RID=0) 1 MA AR PSPLWLLGLA LCALGGGG-PGPRPPPGCPPRRLGPRERRDLQREILAVLGLPGRPRPR T 62

[XP_023483599.1](https://www.ncbi.nlm.nih.gov/protein/XP_023483599.1?report=genbank&log$=protalign&blast_rank=12&RID=0) 1 MA AR PSPLWLLGLA LCALGGGG-PGPRPPPGCPPRRLGPRERRDLQREILEVLGLPGRPRPR T 62

[XP_014947141.3](https://www.ncbi.nlm.nih.gov/protein/XP_014947141.3?report=genbank&log$=protalign&blast_rank=13&RID=0) 1 MA AR PGPLWLLGLA LCALSGGGvPGPRPPLGCPQRRLGPRERRDLQREILAVLGLPGRPRPR A 63

[XP_027824135.1](https://www.ncbi.nlm.nih.gov/protein/XP_027824135.1?report=genbank&log$=protalign&blast_rank=14&RID=0) 1 MA AR PGPLWLLGLA LCALSGGGvPGPRPPLGCPQRRLGPRERRDLQREILAVLGLPGRPRPR A 63

[XP_024213824.1](https://www.ncbi.nlm.nih.gov/protein/XP_024213824.1?report=genbank&log$=protalign&blast_rank=15&RID=0) 1 MA AR PGPLWLLGLT LCALGGGG-PGLRPQPGCPQRRLGARERRDVQREILAVLGLPGRPRPR A 62
[truncated: 158,797 more chars]
