# Supplementary material for: Interleukin-34 orchestrates bone formation through its binding to bone morphogenic proteins
Source: Theranostics. 2025 Feb 11;15(7):3185–202. doi: 10.7150/thno.107340 (PMC11898274; doi:10.7150/thno.107340)
Supplement: Supplementary file 3 — Supplementary tables. [file thnov15p3185s3.pdf]

Table S1 Qiagen q-PCR murine primers

**Official full name; Alias**	**Official symbol**	**Gene Globe Id**
Alkaline phosphatase, liver/bone/kidney	ALPL	QT00012957
Osteocalcin or bone gamma-carboxyglutamate protein	BGLAP	QT00232771
Runt-related transcription factor 2; *CBFA1*	RUNX2	QT00020517
Tumor necrosis factor receptor superfamily, member 11b	TNFRSF11B	QT00014294
Macrophage Colony Stimulating Factor	MCSF	QT00035224
Macrophage Colony Stimulating Factor Receptor	MCSFR	QT00073276
Bone Morphogenetic Protein Receptor 1A	BMPR1A	QT00085358
Bone Morphogenetic Protein Receptor 2	BMPR2	QT00226065
Activin Receptor 2A	ACVR2A	QT00077749

Table S2: Eurogentec q-PCR murine primers

**Official full name; Alias**	**Official symbol**	**Gene Globe Id**
Interleukin-34	IL-34	Fwd 5’-GGA CAC ACT TCT GGG GAC A-3’<br>Rev 5’-CCA AAG CCA CGT CAA GTA GG-3’
Langerin	CD207	Fwd 5’-TCA CCT CCA TTG TGC TTC AG-3’<br>Rev 5’-ATC GTC CAC ACG ACC TCT TT-3’
Glyceraldehyde-3-phosphate dehydrogenase	GAPDH	Fwd 5’-TGC GAC TTC AAC AGC AAC TC-3’<br>Rev 5’-CTT GCT CAG TGT CCT TGC TG-3’
